# Physicochemical Properties of 20 Ionic Liquids Prepared by the Carbonate-Based IL (CBILS) Process

**DOI:** 10.1021/acs.jced.3c00687

**Published:** 2024-04-15

**Authors:** Lukas Pachernegg, Janine Maier, Reyhan Yagmur, Markus Damm, Roland Kalb, Anna Maria Coclite, Stefan Spirk

**Affiliations:** † Institute of Bioproducts and Paper Technology, Graz University of Technology, Graz 8010, Austria; ‡ Ecolyte GmbH, Inffeldgasse 21, Graz 8010, Austria; § Proionic GmbH, Grambach A-8074, Austria; ∥ Institite of Solid State Physics, Graz University of Technology, Graz 8010, Austria

## Abstract

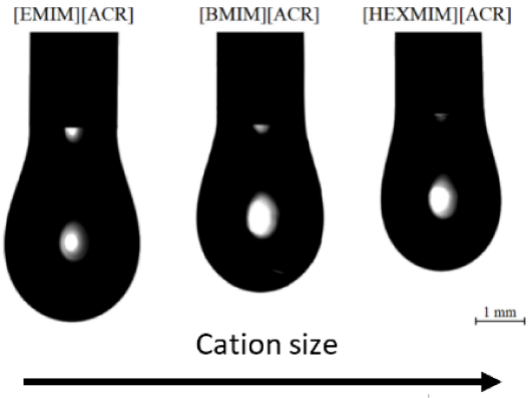

Ionic liquids (ILs) are an emerging materials’ class with applications in areas such as energy storage, catalysis, and biomass dissolution and processing. Their physicochemical properties including surface tension, viscosity, density and their interplay between cation and anion chemistry are decisive in these applications. For many commercially available ILs, a full set of physicochemical data is not available. Here, we extend the knowledge base by providing physicochemical properties such as density (20 and 25 °C), refractive index (20 and 25 °C), surface tension (23 °C, including polar and dispersive components), and shear viscosity (ambient atmosphere, shear rate 1–200 s^–1^), for 20 commercial ILs. A correlation between the crystal volume, dispersive surface tension, and shear viscosity is introduced as a predictive tool, allowing for viscosity estimation. Systematic exploration of cation/anion alkyl side chain lengths reveals the impact on the IL’s physicochemical attributes. Increasing the anion’s headgroup decreases surface tension up to 35.7% and consequently shear viscosity. We further demonstrate that the dispersive part of the surface tension linearly correlates with the refractive index of the ionic liquid. While we provide additional physicochemical data, the screening and modeling efforts will contribute to better structure property predictions enabling faster progress in design and applications of ILs.

## Introduction

Ionic liquids are a class of organic salts with melting points below 100 °C or even below room temperature, so-called room temperature ionic liquids. In recent years, they have attracted more and more attention as they offer unique properties, which clearly show potential for applications in catalysis, electrochemistry, energy storage, and as solvents for biopolymers.^1,2^ Ionic liquids have been proven to be capable to dissolve cellulose, lignin, starch, and chitin.^3^ In particular, the dissolution of cellulose remains a complex topic for various applications, including fiber production. In the case of cellulose, strong intramolecular and intermolecular interactions, as well as the corresponding hierarchical structure, hinder the dissolution process.^4^ Therefore, it either involves toxic chemicals, e.g., Na_2_S, or complex and expensive processing.^5,6^ Ionic liquids offer an alternative pathway for the dissolution and purification of cellulose. This dissolution process proceeds via the swelling of the cellulose fiber in the ionic liquid, resulting in the disruption of the crystalline regions. Consequently, the ions of the ionic liquid form a strong hydrogen bond complex with the individual cellulose chains (C3 and C6 hydroxyl groups), which entangles the individual chains from the bulk material.^4,7−9^ These steps are governed by the physicochemical properties of the ionic liquids, including viscosity, surface tension, impurities, and the molecular structure of the individual ions.^7,10,11^ Ohno et al., for example, were able to show that an increase in cation chain length increases the solubilization temperature of cellulose.^12^ For example, an increase in viscosity increases the penetration time of the ionic liquid into the cellulosic materials and therefore decreases its suitability for high throughput industrial processes in lignocellulosic industries. However, many of these properties are not available for numerous ionic liquids, or in some cases, they feature variations, potentially caused by impurities, including halogenides and water. The measured density at 298 K of [EMIM][OAc] for example varies in literature between 1.09778 g·cm^–3^,^13^ 1.102 g·cm^–3^,^14^ and 1.10269 g·cm^–3^,^15^ based on varying water contents and measurement methods. It should be noted here that depending on the used ILs, selective dissolution of biomass components can also be achieved, which is increasingly attracting attention in pulping processes. For example, lignin and cellulose can be separately dissolved by using different ionic liquids, providing the advantage to the paper industry to avoid extensive chemistry during pulping (e.g., via Kraft process).^16^

**Figure 1:**
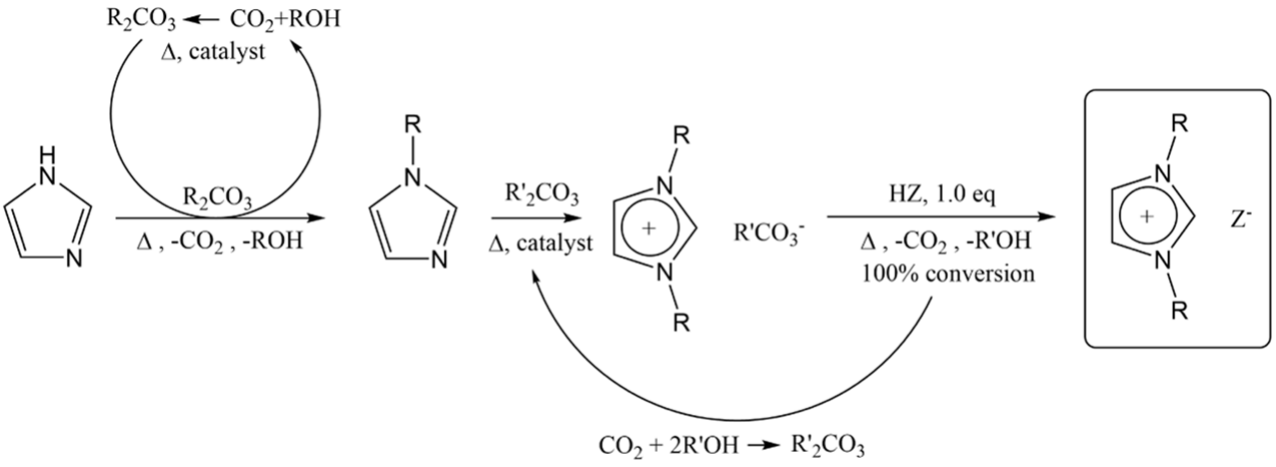
General route for carbonate-based ionic liquid synthesis (CBILS). Reproduced from ref.^17^ with permission from the PCCP Owner Societies.^17^

The well-known, halide free carbonate based ionic liquid synthesis (CBILS) process enables to prevent halide contaminations in the synthesis of ionic liquids (see Figure 1), minimizing the influence of halides.^17^ By measuring the water content of our samples, we can ensure further the reproducibility of the measurements. In this study, we investigated the physicochemical properties of a series of ILs using various techniques, with a focus on NMR spectra, refractive index, density, viscosity, water content, and the OWRK parameters. We further tried to correlate these physical properties with each other to reduce measurement time and for quality control. By understanding the properties of these ILs, we may improve our insights into their potential uses and improve their performance in various applications by better understanding the interconnections of the properties. We selected a set of 20 ionic liquids that are commercially available and can sustainably be produced using the CBILS process.

## Experimental Section

### Materials

1-Butyl-3-methylimidazolium acrylate [BMIM][ACR], 1-butyl-3-methylimidazolium acetate [BMIM][OAc], 1-butyl-1-methylpyrrolidinium bis(fluorosulfonyl)imide [BMPyrr][FSI], cholinium l-lysinate [Chol][Lys], 1,8-diazabicyclo[5.4.0]undec-7-enium acetate [DBUH][OAc], 1-ethyl-3-methylimidazolium acrylate [EMIM][ACR], 1-ethyl-3-methylimidazolium diethylphosphate [EMIM][DEP], 1-ethyl-3-methylimidazolium bis(fluorosulfonyl)imide [EMIM][FSI], 1-ethyl-3-methylimidazolium methanesulfonate [EMIM][MeSO_3_], 1-ethyl-3-methylimidazolium acetate [EMIM][OAc], 1-ethyl-3-methylimidazolium octanoate [EMIM][OOc], 1-ethyl-3-methylimidazolium propionate [EMIM][OPr], 1-ethyl-3-methylimidazolium trifluoromethanesulfonate [EMIM][OTf], 1-ethyl-3-methylimidazolium thiocyanate [EMIM][SCN], 1-ethyl-3-methylimidazolium bis(trifluoromethylsulfonyl)imide [EMIM][TFSI], 1-hexyl-3-methylimidazolium acrylate [HEXMIM][ACR], pyrrolidinium acetate [Pyrr][OAc], pyrrolidinium formate [Pyrr][OFm], and triethylammonium methanesulfonate [TEAH][MeSO_3_] were supplied by proionic GmbH (Grambach, Austria). 1-Ethyl-3-methylimidazolium dicyanamide [EMIM][DCA] was purchased from Sigma-Aldrich (Germany). All ionic liquids (Table 1) were used as received and not purified or dried before use. DMSO-d_6_ (99.9 atom % D) containing 0.03% (v/v) TMS and D_2_O was purchased from Sigma-Aldrich (Germany). The Karl Fischer titration used a two-component HYDRANAL – Titrant 5 (Honeywell Fluka, Germany) solution. *n*-Heptane (ROTIPURAN 99%) for surface tension measurements was obtained from Carl-Roth (Karlsruhe, Germany). Aquastar water standard 1% was purchased from Sigma-Aldrich (Germany). The chemicals used are listed in Table 2.

**Table 1:** Properties of the Ionic Liquids Used in This Study^a^

cation	anion	abbreviation	*M*_w_/g·mol^–1^	molecular formula	CAS-No.	supplier	H_2_O content (w/w)
1-butyl-3-methylimidazolium	acrylate	[BMIM][ACR]	210.28	C_11_H_18_N_2_O_2_	-	proionic Gmbh	0.838 ± 0.040
1-butyl-3-methylimidazolium	acetate	[BMIM][OAc]	198.26	C_10_H_18_N_2_O_2_	284049-75-8	proionic Gmbh	0.721 ± 0.097
1-butyl-1-methylpyrrolidinium	bis(fluorosulfonyl)-imide	[BMPyrr][FSI]	322.39	C_8_H_21_F_2_N_2_O_4_S_2_	1057745-51-3	proionic Gmbh	0.042 ± 0.028
cholinium	l-lysinate	[Chol][Lys]	249.35	C_11_H_28_N_3_O_3_	1361335-94-5	proionic Gmbh	1.760 ± 0.006
1,8-diazabicyclo[5.4.0] undec-7-enium	acetate	[DBUH][OAc]	212.29	C_11_H_20_N_2_O_2_	36443-65-9	proionic Gmbh	0.453 0.012
1-ethyl-3-methylimidazolium	acrylate	[EMIM][ACR]	182.23	C_9_H_14_N_2_O_2_	-	proionic Gmbh	1.216 ± 0.044
1-ethyl-3-methylimidazolium	dicyanamide	[EMIM][DCA]	177.21	C_8_H_11_N_5_	370865-89-7	Sigma-Aldrich	0.704 ± 0.052
1-ethyl-3-methylimidazolium	diethylphosphate	[EMIM][DEP]	264.26	C_10_H_21_N_2_O_4_P	848641-69-0	proionic Gmbh	1.188 ± 0.010
1-ethyl-3-methylimidazolium	bis(fluorosulfonyl)-imide	[EMIM][FSI]	206.26	C_6_H_11_F_2_N_3_O_4_	235789-75-0	proionic Gmbh	0.592 ± 0.092
1-ethyl-3-methylimidazolium	methanesulfonate	[EMIM][MeSO_3_]	170.21	C_7_H_14_N_2_O_3_S	145022-45-3	proionic Gmbh	0.308 ± 0.035
1-ethyl-3-methylimidazolium	acetate	[EMIM][OAc]	271.21	C_8_H_14_N_2_O_2_	143314-17-4	proionic Gmbh	0.200 ± 0.022
1-ethyl-3-methylimidazolium	octanoate	[EMIM][OOc]	184.24	C_14_H_26_N_2_O_2_	1154003-55-0	proionic Gmbh	0.791 ± 0.052
1-ethyl-3-methylimidazolium	propionate	[EMIM][OPr]	260.23	C_9_H_16_N_2_O_2_	865627-64-1	proionic Gmbh	0.787 ± 0.091
1-ethyl-3-methylimidazolium	trifluoromethane sulfonate	[EMIM][OTf]	169.25	C_7_H_11_F_3_N_2_O_3_S	145022-44-2	proionic Gmbh	0.047 ± 0.002
1-ethyl-3-methylimidazolium	thiocyanate	[EMIM][SCN]	291.29	C_7_H_11_N_3_S	331717-63-6	proionic Gmbh	0.320 ± 0.081
1-ethyl-3-methylimidazolium	bis(trifluoro-methylsulfonyl)imide	[EMIM][TFSI]	391.31	C_8_H_11_F_6_N_3_O_6_S_2_	174899-82-2	proionic Gmbh	0.034 ± 0.001
1-hexyl-3-methylimidazolium	acrylate	[HEXMIM][ACR]	238.34	C_13_H_22_N_2_O_2_	-	proionic Gmbh	0.627 ± 0.069
pyrrolidinium	acetate	[Pyrr][OAc]	131.17	C_6_H_13_NO_2_	35574-23-3	proionic Gmbh	2.900 ± 0.082
pyrrolidinium	formate	[Pyrr][OFm]	117.15	C_4_H_11_NO_2_	444810-12-2	proionic Gmbh	12.422 ± 0.674
triethylammonium	methanesulfonate	[TEAH][MeSO_3_]	197.29	C_7_H_19_NO_3_S	93638-15-4	proionic Gmbh	0.551 ± 0.0321

a The water content (w/w) of the ILs has been determined using Karl-Fischer titration. Purities have been provided by the suppliers and were >98% for all ILs. *M*_w_ denotes the molecular weight.

**Table 2:** Chemicals Used in This Study

component	CAS-No.	purity	supplier
DMSO-d_6_	2206-27-1	99.9 atom % D	Sigma-Aldrich
D_2_O	7789-20-0	99.9%	Eurisotop SAS
HYDRANAL Titrant 5	67-56-1	-	Honeywell Fluka
*n*-heptane	142-82-5	Rotipuran 99%	Carl Roth
Aquastar water standard 1%	-	certified reference material	Merck

### Methods

#### Density

The density was measured with a density meter DMA 4500 M (Anton Paar, Graz, Austria) at 20 and 25 °C. Between measurements, the measuring cell was cleaned with isopropanol and deionized water and dried. To minimize the risk of solvent contamination, the density of air was measured as a reference between the measurements of the ionic liquids and compared to the values provided in the manual by Anton Paar. The accepted deviation from the standard value was ±0.0001 g·cm^–3^. At least three measurements were performed per sample. The measurement repeatability according to the manufacturer is 0.00001 g·cm^–3^.

#### Karl Fischer Titration

For water content measurements, a SI Analytics Titroline 7500-05 KF (Xylem Analytics, Mainz, Germany) with the two-component HYDRANAL – Titrant 5 (Honeywell Fluka, Germany) titration solution was used. Reference measurements have been conducted with a Karl Fischer Aquastar water standard of 1%.

#### NMR Measurements

^1^H NMR spectra were obtained using a Bruker NMR with an autosampler at 300 MHz with a 4 s delay time. The results were averaged over 8 scans. For the measurements, 0.1 mL of the samples was dissolved in 0.8 mL of DMSO-d_6_ or D_2_O. The signals of the solvents at 2.50 ppm for DMSO and 4.79 ppm for water in D_2_O were used as reference.^18^ For the analysis, the MNova software from Mestrelab Research was utilized. ^13^C NMR spectra were obtained using a Bruker NMR instrument at 76 MHz. The results were averaged over 8 scans. For the measurements, ca. 0.1 mL of the samples was dissolved in 0.8 mL of DMSO-d_6_ or D_2_O. For the analysis, the MNova software from Mestrelab Research was utilized.

#### Refractive Index

The refractive index was measured with a Abbemat 550 refractometer (Anton Paar, Graz, Austria) with a wavelength of 569 nm. The temperature in the measuring cell was varied between 20 and 25 °C. At least three measurements were performed per sample. As a reference, the refractive index of deionized water was measured and compared to the values provided in the manual by Anton Paar. The deviation was between the limits provided by the supplier for water. The tolerance of the device is ±0.00002.

#### Rheology

The rheology of the ionic liquids was measured with a modular compact rheometer MCR 502 (Anton Paar, Graz, Austria) between a cone–plate (MS-CP50-1)/plate. Sample temperature was controlled with a Peltier element. Measurement procedure was as follows: 0.5–0.6 mL of ionic liquid was placed between cone–plate and plate. The excess material was removed before the measurement. The sample was equilibrated for 4 min at 20 °C, 22.5 °C, and 25 °C. Measurements were done with a shear rate (γ) of 1–200 s^–1^ with linear steps starting with 10 s hold at γ = 1 s^–1^ and 1 s at γ = 200 s^–1^ for each temperature. In the last step, the sample was equilibrated for 4 min of equilibration at 15 °C prior to the measurement. The sample was analyzed at a constant shear rate γ = 50 s^–1^ and constant temperature increase between 15 and 30 °C. Calculation was performed with the RheoCompass software package from Anton Paar and Matlab R2020b (MathWorks Inc., USA). The measurement was repeated at least three times per ionic liquid under an ambient (laboratory) atmosphere (23 °C). The device was calibrated and tested by Anton Paar in advance of the measurements. Test protocols are provided upon request.

#### Surface Tension Measurement

Surface tension was obtained with a Dataphysics model OCA200 (Dataphysics, Filderstadt, Germany). The pendant drop method was employed with a 1.83 mm diameter tip (Dataphysics, Filderstadt, Germany) on a medical syringe. The liquid was dosed with an automated liquid dosing unit. Drop size was measured in air (climatized laboratory according to ISO 187, 23 °C, 50% r.h.) and *n*-heptane as the surrounding medium in a homemade setup. The drop size was determined via an ellipse fitting algorithm. At least five measurements per ionic liquid and surrounding medium were performed and averaged. The polar and dispersive ratios were calculated by using the Owens-Wendt-Rabel and Kaelble (OWRK) theory. To ensure a viable measurement, the Dataphysics OCA200 device and the homemade setup were tested with deionized water in advance of the measurements. Details on the calibration can be obtained in the Supporting Information.^19^ Surface tension of *n*-heptane was taken from Jasper^20^ at 20.14 mN·m^–1^.

## Results and Discussion

### NMR Spectroscopy

Depending on the solubility of the ionic liquids, the ^1^H and ^13^C NMR spectra were acquired either in D_2_O ([BMIM][OAc], [BMIM][ACR], [Chol][Lys], [DBUH][OAC], [EMIM][ACR], [EMIM][DEP], [EMIM][MeSO_3_], [EMIM][OAc], [EMIM][OOc], [EMIM][OPr], [EMIM][SCN], [HEXMIM][ACR], [Pyrr][OAc], [Pyrr][OFm], and [TEAH][MeSO_3_]) or in d_6_-DMSO ([BMPyrr][FSI], [EMIM][DCA], [EMIM][FSI], [EMIM][OAc], [EMIM][OTf], and [EMIM][TFSI]). While the ^13^C NMR spectra clearly indicate the purity of the samples, a peculiarity in the ^1^H NMR spectra was observed as the integrals of the protons at the anions and cations do not match. Imidazolium-based ILs feature a masked carbene, whose protons are very acidic and therefore can easily undergo H/D exchange, resulting in a weaker signal for the protonated form (Table S3). Depending on the ratio of D_2_O and [EMIM][OAc], the exchange reaction rate of the C2–H (Figure S36) is between 10^–4^ and 10^–1^ s^–1^,^21^ which translates to a detectable change in the experimental time frame. Similar behavior can be observed for the acrylate anion (e.g., Figure S24), which is consistent with the literature.^22^ There are several publications about this topic, and we refer the interested reader to more specific literature as this was not the focus of this study. The ^13^C spectra of [EMIM][OTf] (Figures S43 and S44) and [EMIM][TFSI] (Figures S48 and S49) show a coupling of the carbon atoms C_13_ and C_15_/C_18_ with the fluor atoms.^23,24^

All NMR spectra of the ionic liquids are presented in the Supporting Information.

### Density and Refractive Index

**Figure 2:**
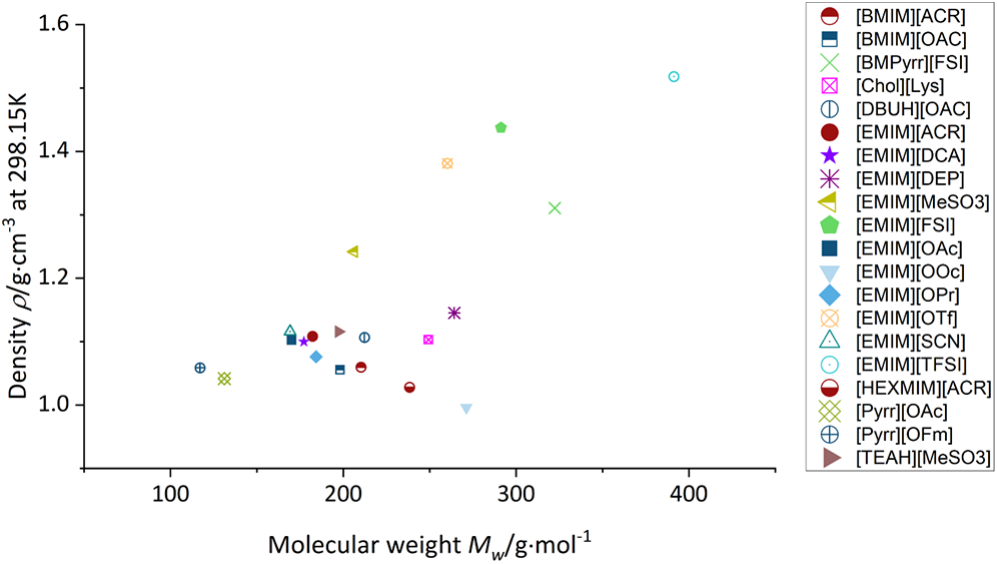
Density at 20 °C as a function of the molecular weight. Ionic liquids with the same anion are of the same color. Measurement uncertainty of the density measurements is ±0.0005 g·cm^–3^.

The density of the tested ionic liquids is generally above the density of water (exception [EMIM][OOc]) and ranges from 0.9960 g·cm^–3^ ([EMIM][OOc]) to 1.5179 g·cm^–3^ ([EMIM][TFSI]) (Table 3). In literature, the density values (in g·cm^–3^, 25 °C) for [EMIM][TFSI] are in the range of 1.5147,^25^ 1.5118^26^ to 1.5187^27^ and correlate well with our data. Similarly, the density (in g·cm^–3^, 25 °C) for [EMIM][OAc] in the literature ranges from 1.09778,^13^ 1.09944,^28^ and 1.102^14^ to 1.1088^29^ and deviates only slightly from our value (1.10231 g·cm^–3^). A detailed list of literature values is in the Supporting Information (Tables S4 and S5). These variations are the result of impurities from the production process, water contaminant, and measurement uncertainties. The water contents of the ionic liquids are listed in the chemicals table (Table 1 and Figure S2). In general, halogenides are a major impurity influencing the physicochemical properties of ionic liquids.^30^ The synthesis route using the CBILS process leads to negligible level of halogenides in the ILs.^17^ NMR spectroscopy was further used to validate the purity of the ILs (see the Supporting Information). Increasing the chain length of the anion ([OAc], [OPr], [OOc]) decreases the density of the ionic liquids as well as their refractive index (Figure 2). The same trend can be observed when increasing the side chain length of the cation. This behavior supports already existing data in literature, which was observed with various cation/anion combinations.^31−33^ Fluorine containing ionic liquids, namely, [BMPyrr][FSI] [EMIM][FSI], [EMIM][OTf], and [EMIM][TFSI], show the highest densities of all ionic liquids in this study. This contradicts the assumption that these anions are less coordinating and show weaker interactions with their cation.^34^ The higher mass of fluorine compared to hydrogen (approximately 19-fold), paired with a similar van der Waals radius (fluorine: 1.47 Å; hydrogen: 1.10 Å), leads to an increase in molar mass, with a comparable low increase of molar volume. This leads ultimately to the strong increase of density, despite weaker interactions.^35,36^

**Table 3:** Density (ρ) and Refractive Index (*n*_D_) of the Ionic Liquids^a^

ionic liquid	ρ_20 °C_^b^/g·cm^–3^	ρ_25 °C_^b^/g·cm^–3^	*n*_D_ 20 °C^c^	*n*_D_ 25 °C^c^
[BMIM][ACR]	1.0623	1.0597	1.5077	1.5062
[BMIM][OAC]	1.0586	1.0555	1.4933	1.4917
[BMPyrr][FSI]	1.3105	1.3066	1.4462	1.4448
[Chol][Lys]	1.1061	1.1030	1.5074	1.5060
[DBUH][OAC]	1.1101	1.1065	1.5208	1.5191
[EMIM][ACR]	1.1114	1.1083	1.5155	1.5135
[EMIM][DCA]^d^	1.1032	1.0999	1.5143	1.5127
[EMIM][DEP]	1.1484	1.1451	1.4742	1.4728
[EMIM][FSI]	1.4442	1.4373	1.4484	1.4470
[EMIM][MeSO_3_]	1.2452	1.2418	1.4970	1.4957
[EMIM][OAc]	1.1053	1.1023	1.5000	1.4982
[EMIM][OOc]	0.9992	0.9960	1.4859	1.4847
[EMIM][OPr]	1.0792	1.0761	1.4962	1.4951
[EMIM][OTf]	1.3852	1.3810	1.4362	1.4349
[EMIM][SCN]	1.1189	1.1159	1.5527	1.5511
[EMIM][TFSI]	1.5230	1.5179	1.4244	1.4229
[HEXMIM][ACR]	1.0310	1.0280	1.5017	1.5001
[Pyrr][OAc]^d^	1.0452	1.0417	1.4643	1.4627
[Pyrr][OFm]^d^	1.0627	1.0586	1.4639	1.4621
[TEAH][MeSO_3_]^d^	1.1191	1.1157	1.4624	1.4609

a Temperature accuracy of the densiometer ±0.02 °C and of the refractometer ±0.03 °C. All measurements were conducted in ambient atmosphere at 101.3 kPa.

b Measurement uncertainty below ±0.0005 g·cm^–^³.

c Measurement uncertainty below ±0.0002.

d Non-CBILS.

### Surface Tension

The Owens–Wendt–Rabel–Kaelble theory (OWRK) separates the surface tension (σ_L_) on the interface into two fractions, the so-called polar (σ_L,p_) and dispersive (σ_L,d_) interactions (eq 1):^37−40^
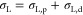
1

The total surface tension of the liquid (σ_L_) is calculated by analyzing the maximum drop size in air as surrounding medium.^41^ For determination of the dispersive interaction (σ_L,d_), a known liquid showing only a dispersive ratio is used as the surrounding medium. As surrounding liquid, we chose *n*-heptane, which is known to have solely dispersive contributions to SFT.^20^ The following equation therefore holds true (eq 2):
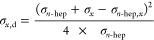
2

where σ_*x*_,d is the dispersive ratio of the tested liquid, σ_*n*-hep_ is the surface tension of *n*-heptane in air, σ_*x*_ is the surface tension of the tested liquid in air, and σ_*n*-hep_,*_x_* is the interfacial tension between the tested liquid and *n*-heptane. The polar ratio can then be calculated via rearranging eq 1 (eq 3):
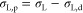
3

**Figure 3:**
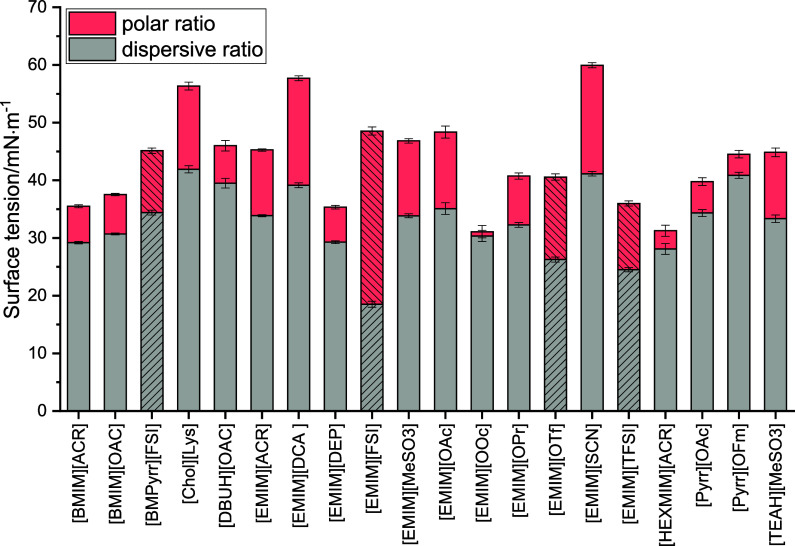
Surface tension of the tested ionic liquids including the polar (red) and dispersive (gray) ratios of the surface tension. Fluorine containing ionic liquids are marked with black cross lines. The results of these fluorine ionic liquids need to be treated with caution since additional interactions, which cannot be quantified with the method used, may influence the surface tension.

Figure 3 shows the surface tension of the ILs. The tested liquids show a broad range of surface tensions, reaching from 31.09 mN·m^–1^ ([EMIM][OOc]) to 59.94 mN·m^–1^ ([EMIM][SCN]) (see Figure 3). The surface tension of the ionic liquids is generally below the value of water (σ_water_: 72.8 mN·m^–1^)^38^ but higher than that for most organic solvents such as benzene, ethanol, methanol, toluene, or chloroform.^20,42,43^ Water has strong hydrogen bonds per unit volume, which is exemplified by the high polar ratio of 51.00 mN·m^–1^.^43^ The weaker coulomb interactions of ILs^44^ per unit volume, which are the main constituent to the polar ratio of the surface tension, lead therefore to a lower polar ratio. An overview of literature values can be seen in the (Table S6). Measured values can be taken from the (Table S7) Different measurement techniques in the literature may lead to some deviations in the results and need to be taken into account when comparing values.

The surface tension (SFT) of the tested ionic liquids is governed by the dispersive part. The dispersive part on the overall surface tension is between 64.8% for [EMIM][OTf] and 97.6% for [EMIM][OOc]. An increase in the side chain length of the cation (ethyl–butyl–hexyl) has a significant influence on the polar contribution. The polar contribution decreases by around 70% with an increase in chain length, whereas the dispersive contribution decreases by only approximately 17%. This shifts the overall SFT toward a more nonpolar interaction. The lowered overall surface tension may be caused by the increase in molecular area.^45−47^ As discussed before, the polar contribution of a molecule is related to its ability to form dipole–dipole interactions with other molecules.^43^ The longer the alkyl side chain, the weaker the dipole–dipole interactions are in relation to the molecule size. This behavior is evident for example in alcohols when increasing from methanol to propanol.^48^ Similarly, the polar contribution in ionic liquids decreases as the chain length increases.^49^ With an increasing chain length, charge density decreases. This leads to a decrease in the dispersive contributions. In ionic liquids, additional interactions can come from liquid crystalline structures,^50,51^ which is favored with increasing chain length. From these structures, an additional interaction can arise, leading to an increase in the dispersive ratio. This partially compensates for the decrease of the dispersive ratio caused by the lower charge density. Overall, this results in a strong decrease of the polar ratio but a comparable small change of the dispersive ratio. A change from acetate ([OAc]) to propanoate ([OPr]) decreases the polar contribution from 13.29 mN·m^–1^ to 8.23 mN·m^–1^, respectively. The further chain length increase from propanoate ([OPr]) toward the octanoate ([OOc]) decreases the contribution of polar interactions again. The influence on the dispersive interactions is again relatively low. This behavior further strengthens the aforementioned hypothesis.

The influence of the Δp*K*_a_-value (difference in p*K*_a_ of the educts) of protic ionic liquids on the surface tension can be illustrated by the formulas [EMIM][MeSO_3_] and [TEAH][MeSO_3_]. The Δp*K*_a_-value shows a strong correlation with the proton transfer ability between the educts in protic ionic liquids.^52^ The ions present show permanent dipoles, which effect the polar ratio of the surface tension by forming coulomb and hydrogen bonds.^53,54^ [EMIM] cations have a p*K*_a_ value, depending on their anionic counterpart, of around 28.^55^ In contrast, the p*K*_a_ value of triethylammonium [TEAH], the protonated form of triethylamine, is around 8–10.^56,57^ Having the same anion, the difference of the Δp*K*_a_-value therefore significantly influences the polar ratio of the surface tension in these ILs. The dispersive parts are essentially identical (33.86 and 33.35 mN·m^–1^ for [EMIM][MeSO_3_] and [TEAH][MeSO_3_], respectively). Therefore, we assume that the influence of the Δp*K*_a_-value is important in the context of the polar ratio but has a rather minor influence on the dispersive ratio.

[EMIM][DCA] and [EMIM][SCN] exhibit the highest surface tensions among all ILs in this study. The dicyanamide [DCA] and the thiocyanate [SCN] anions represent both pseudohalides.^58^ These pseudohalides show strong dipoles, caused by their highly delocalized charges.^59^ These permanent dipoles enable Keesom forces, which are the main contributions to the polar interactions.^40^ This results in the highest polar ratio of all of the tested ionic liquids. [EMIM][DCA] and [EMIM][SCN] show additionally a high dispersive ratio. These dispersive forces predominantly stem from London dispersion interactions, which are caused by the interaction of induced dipoles with fluctuations in charge density.^37^ These dipoles can easily be induced in halides,^60^ and it is assumed that the behavior is similar in pseudohalides. This behavior results in a highly dispersive contribution to the surface tension of the ionic liquids (39.15 mN·m^–1^ and 41.14 mN·m^–1^).

[Chol][Lys] shows almost as high surface tension as [EMIM][DCA] and [EMIM][SCN]. The l-lysinate anion can form strong hydrogen bonds with itself and the cholinium cation. These strong interactions are reflected in high polar contributions to the SFT. The high dispersive ratio, when compared to many [EMIM]-based ionic liquids in this study, is not fully understood. We would expect a lower polarizability of the cholinium cation, compared to the aromatic [EMIM] cation. Ionic liquids containing fluorine, namely, [BMPyrr][FSI], [EMIM][FSI], [EMIM][OTf], and [EMIM][TFSI], may show only parts of the interaction. Fluorine is known to show additional fluorophilic interactions,^61^ which are not sufficiently considered in the OWRK model. This additional interaction parameter is of interest if the ionic liquids containing those fluorine components interact with solids containing fluorine by themselves. This includes a variety of PTFE polymers and needs to be taken into account if such systems are under investigation. Therefore, these data need to be treated with caution.

**Figure 4:**
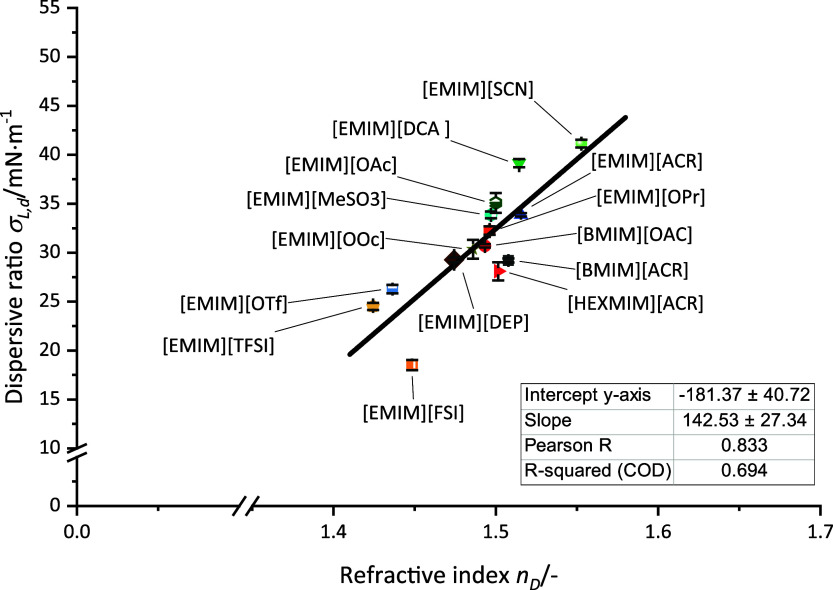
Dispersive ratio of the surface tension vs refractive index and its correlation for imidazolium-based ionic liquids. Measurement uncertainty of the refractive index ±0.0002.

The polarizability of a liquid is connected to its refractive index.^62^ Polarizability is the underlying property, which is necessary for dispersive interactions within the system.^37,40,63^ The tested imidazolium-based ionic liquids show this correlation (Figure 4) between the dispersive ratio of the surface tension and the refractive index. As discussed earlier, the additional interactions of fluorine-based anions may lead to challenges in the data acquisition but surprisingly does not negatively influence the relationship between refractive index and dispersive contributions.

### Rheology

**Figure 5:**
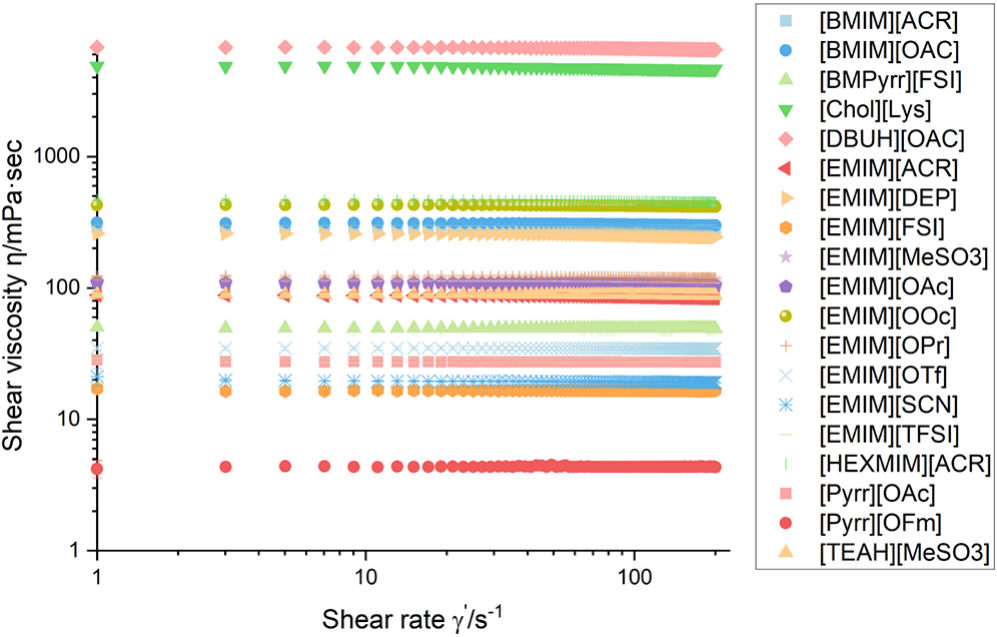
Shear viscosity η in mPa·s measured at 25 °C under an ambient atmosphere. Deviations are the difference between three independent measurements. Data are available in Table S9.

The viscosity of the analyzed ionic liquids is almost independent of the shear rate in the tested regime (Figure 5). MD simulations made by Blanco-Díaz et al. suggested that at low shear rates (<1 s^–1^), ionic liquids show a strong hydrogen bond network. This network translates into a shear thinning behavior of the ionic liquids under investigation.^64^ Ionic liquids with a high polar ratio of the surface tension should therefore show shear thinning behavior. Although [EMIM][SCN] and [EMIM][DCA] showed a high polar ratio, no shear thinning behavior was visible in the tested range. To validate the MD simulation predictions, we recommend conducting additional investigations on these ionic liquids under conditions of low shear and dry atmosphere.

Slattery and coworkers^65^ were able to show a strong correlation between the molecular volume of the ion pair and the viscosity of the ionic liquid in question. The molecular volume of the ion pair (*V*_Ion Pair_) is defined as the sum of the molecular volumes of each ion (*V*_ion_*C*^+^, *V*_ion_*A*^–^) (eq 4).
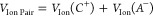
4

These volumes can be estimated from crystal structures (e.g., the CCDC or COD database) containing the ions of interest (more information on how to derive these volumes as well as the used volumes in this study are in the Supporting Information and in references^65−68^). The strong correlation in the original publication is only valid for one type of anion combined with various cations, e.g., [TFSI] with [EMIM], [BMIM], [HEXMIM], [SEt_3_], [BmPyrr], and others.^65^ This approach still lacks an additional parameter to account for the interactions in the bulk between the ions.

We propose to combine the volume of the ion pair with the dispersive ratio of the surface tension, which are independent factors (see Figure S3). The new factor *C* (eq 5) strongly correlates with the viscosity, independent of anion, and cation type. *C* can be calculated by multiplying the volume of the ion pair (*V*_Ion Pair_) with the dispersive ratio of the surface tension (σ_L,d_).
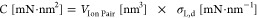
5

**Figure 6:**
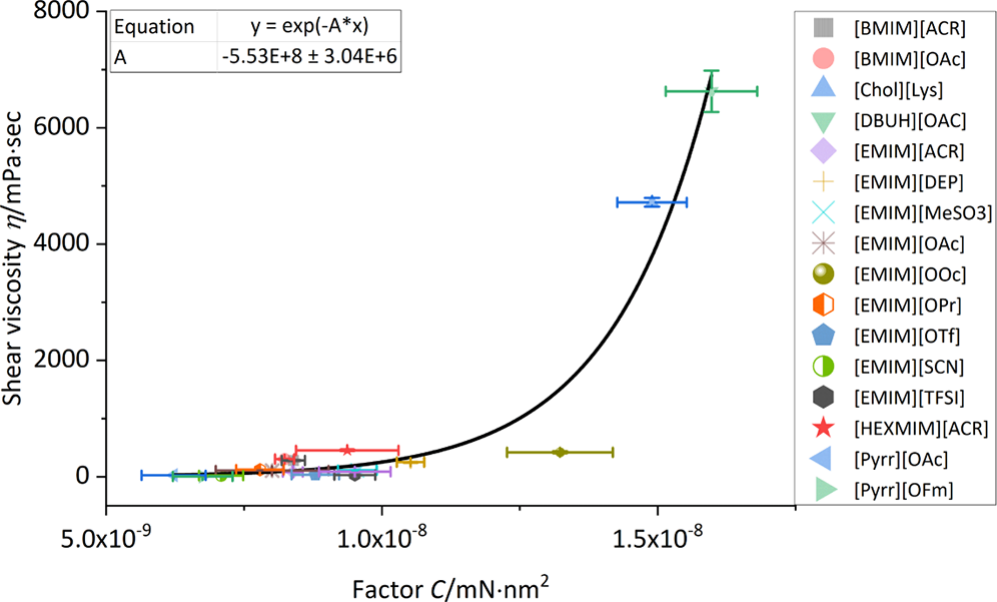
Correlation between the average shear viscosity at 25 °C and the introduced factor *C*. [BMPyrr][FSI] is missing, caused by the lack of crystal data. Deviations of the *x* values represent the measurement uncertainties of the surface tension measurements. Shear viscosity deviations represent the maximum deviation that occurred during rheology measurements between three individual measurements. Data available in Table S11.

With this relationship, it is possible to predict the (shear) viscosity or dispersive ratio of the surface tension of ionic liquids with an exponential equation (eq 6):
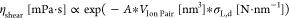
6

where η_shear_ is the averaged shear rate (shear rate 1–200 s^–1^) at 25 °C, and *A* the scaling factor of the equation. The scaling factor *A* was fitted to the data of 16 ionic liquids in this study, since the crystal data were not available for every IL in question. This equation represents an Arrhenius type equation, with the usually used pre-exponential factor being set to 1. This factor was set to a value of 1 to minimize the number of constants, which are needed for the fit. Additionally, this improved the fit quality. The fit suggests that there is an exponential increase of the shear viscosity when the factor *C* exceeds 1.25 × 10^–8^. This translates to bulky molecules with a high dispersive ratio (polarizability). Unfortunately, the number of samples in this area is limited, and further testing needs to be done to further validate the proposed correlation. This problem is shown by [EMIM][OOc], the only notable outliner in the calculation. Below the *C*-value of 1.25 × 10^–8^, 13 of the ionic liquids show a good correlation with the proposed model (Figure 6).

A more sophisticated approach would be the use of van der Waals volumes, as suggested in a later publication of Krossing et al.^69^ This approach would further minimize variations in the data and enhance the quality of the prediction. Although a database was already established by Zhu et al.,^70^ it still lacks data for ionic liquids used in this study. The deviation of van der Waals volumes, which were found in the database, is less than 11% compared to the volumes calculated from crystal structures (*V*_Ion Pair_). Since the volumes from crystal structures (*V*_Ion Pair_) are more easily available, we used these data in the study, despite its higher uncertainty.

## Conclusion

In summary, this study characterized 20 ionic liquids prepared by the CBILS process, which encompassed their density, refractive index, surface tension, OWRK parameters, and shear viscosity. Our investigation showed a notable influence of the anion (chain length) on the density, surface tension, and shear viscosity, which dictate the physicochemical properties of these compounds. In contrast, the impact of cation side chain length was of comparably modest effect.

Our findings are in line with the existing literature on other ionic liquids, which supports the reliability of our results. Additionally, we successfully established a correlation between the refractive index and the dispersive ratio of nonfluorinated imidazolium-based ionic liquids. This supports the earlier findings of Tariq et al. and Iglesias-Otero et al. on a different data set.^25,62^ Refractive index measurements are therefore a simple, reliable, and straightforward measurement technique to estimate the dispersive parts of the surface of imidazolium-based ionic liquids.

We propose a new possibility of combining the crystal volume and dispersive parts of the surface tension to estimate shear viscosity. This approach, in combination with the aforementioned relationship of the dispersive part of the surface tension with the refractive index, enables a simple and fast possibility for shear viscosity estimation.

## Supporting Information Available

A detailed list on literature values of densities, refractive index, surface tension, and viscosity; a calculation procedure for the ion pair volumes, the dispersive ratio versus the ion volumes as well as ^1^H and ^13^C NMR spectra for all ionic liquids used in the study; the experimental data, shear viscosity under constant shear (50 s^–1^) rate as well as the data of the shear viscosity experiment under constant temperature (25 °C); the detailed calibration procedure for the surface tension measurements (PDF)
