# Supplementary material for: Physicochemical Properties of 20 Ionic Liquids Prepared by the Carbonate-Based IL (CBILS) Process
Source: J Chem Eng Data. 2024 Apr 15;69(5):1814–23. doi: 10.1021/acs.jced.3c00687 (PMC11090035; doi:10.1021/acs.jced.3c00687)
Supplement: Supplementary file 1 — je3c00687_si_002.pdf [file je3c00687_si_002.pdf]

Supporting information:

Physicochemical properties of 20 ionic liquids prepared by the carbonate-based IL (CBILS®) process

Lukas Pacheregg[†,‡], Janine Maier[†,‡], Reyhan Yagmur[†,‡], Markus Damm[§], Roland Kalb[§], Anna Maria Coclite[‖], Stefan Spirk[†,‡]*

[†]Institute of Bioproducts and Paper Technology, Graz University of Technology, 8010 Graz, Austria.

[‡]Ecolyte GmbH, 8010 Graz, Austria.

[§]Proionic GmbH, 8074 Grambach, Austria.

[c]Institute of Solid State Physics, Graz University of Technology, 8010 Graz, Austria.

*stefan.spirk@tugraz.at

Table of contents

Validation of surface tension measurements 3

Ionic liquids used in the study 6

Density 7

Refractive index 8

Surface tension 9

Water content 11

Relationship ion pair volume and dispersive ratio of ionic liquids 11

Rheology temperature vs. shear rate 12

Rheology literature review 12

Rheology data 18

NMR spectra 67

[BMIM][ACR] 67

[BMIM][OAc]: 68

[BMPyrr][FSI] 69

[Chol][Lys] 70

[DBUH][OAc] 71

[EMIM][ACR] 72

[EMIM][DCA] 73

[EMIM][DEP] 74

[EMIM][FSI] 75

[EMIM][$MeSO_3$] 76
[EMIM][OAc] 77
[EMIM][OOc] 80
[EMIM][OPr] 81
[EMIM][OTf] 82
[EMIM][SCN] 84
[EMIM][TFSI] 85
[HEXMIM][OAc] 87
[Pyrr][OAc] 88
[Pyrr][OFm] 89
[TEAH][$MeSO_3$] 90
References 91

Validation of surface tension measurements

The validation of the measurement setup for surface tension determination was already published by our colleges elsewhere.[1] In brief the validation was done as followed: For validation of the measurement setup used four different liquids with known surface tension components were used without further purification.

Table S1. Chemicals used for surface tension validation and their polar ($\sigma_{L,p}$) and dispersive ($\sigma_{L,d}$) contributions to the surface tension.

Chemical	CAS-NR	Chemical structure	Supplier	$\sigma_{L,p}$/mN·m^{-1}	$\sigma_{L,d}$/mN·m^{-1}
Dimethyl sulfoxide (DMSO)	67-68-5	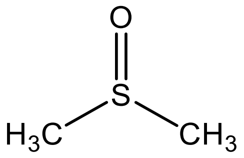	Sigma Aldrich (Germany) Purity: 99%	8.00[2]; 8.68[3]	36.00[2]; 34.86[3]
Ethylene glycol	107-21-1	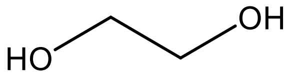	VWR (Germany) Purity: AnalaR®NORMAPUR®	19.00[2]; 21.30[4]; 18.91[5]; 21.40[6]; 16.80[7]	29.00[2]; 26.40[4]; 29.29[5]; 26.30[6]; 30.90[7]
Glycerol	56-81-5	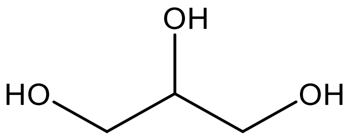	Carl Roth (Germany) Purity: 99%	41.50[8]; 25.00[9]; 26.40[10]; 43.08[5]; 36.90[11]; 34.40[6]; 26.40[7]; 30.00[12]	21.20[8]; 37.40[9]; 37.00[10]; 20.22[5]; 28.30[11]; 29.00[6]; 37.00[7]; 34.00[12]
MilliQ® water	7732-18-5	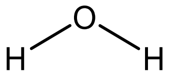	-	52.20[8]; 43.70[9]; 46.80[4]; 53.60[11] ; 50.65[6]; 51.00[7]	19.90[8]; 29.10[9]; 26.00[4]; 18.70[11]; 22.10[6]; 21.80[7]
n-hexane	110-54-3	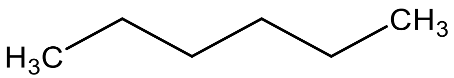	Merck KGaA (Germany) Purity: 99%	0[13, 14]	18.43[13, 14]
n-heptane	142-82-5	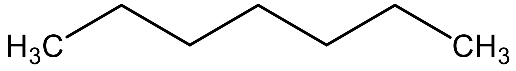	Carl Roth (Germany) Purity: Rotipuran® 99%	0[14]	20.14[14]

The validation of the measurements was done by comparing the measurement values with literature data (**Table S1)** and by calculating the dimensionless Worthington number *Wo* (*Eq S1*).[15]

$$Wo = \frac{\Delta\rho\, g\, V_d}{\pi\, \gamma\, D_n} \qquad \text{Eq S1}$$

$\Delta\rho$ is the density difference between the surrounding media and the liquid in question, g is the gravity constant, V_d is the drop volume, γ is the surface tension of the liquid in question and D_n is the drop diameter. The Worthington number gives one information about the measurement quality. If the number is >0.6 the measurement can be considered as accurate. .[15] Values are overall relatively high, with the exceptions being glycerol in n-hexane and water in n-heptane, both are below 0.6. Nevertheless, we can still anticipate a satisfactory level of accuracy, as the value of 0.56 is only slightly below the 0.6 threshold.

Table S2. Measured values for the surface tensions and interfacial tensions of the test liquids as well as the calculated Worthington numbers. Data taken from Waldner et. al.[1] All measurements have been conducted in a climatized room according to ISO 183 (23°C, 50% r.h., p = 101.3 kPa)

	**Liquid in air**			**Liquid in n-hexane**			**Liquid in n-heptane**		
**Test liquid**	**SFT[a]/ mN·m^{-1}**	**Drop volume/µL**	**Worthington number/-**	**IFT[b]/ mN·m^{-1}**	**Drop volume/µL**	**Worthington number/-**	**IFT[b]/ mN·m^{-1}**	**Drop volume/µL**	**Worthington number/-**
**DMSO**	44.00	16.34	0.70	10.60	9.62	0.69	11.10	9.93	0.64
**Ethylene glycol**	47.40	18.56	0.74	15.80	14.86	0.73	17.20	16.15	0.68
**Glycerol**	64.40	21.45	0.72	28.50	15.54	0.56	28.00	20.13	0.71
**Water**	71.90	30.92	0.73	46.40	51.46	0.65	48.90	50.78	0.56

[a]Surface tension, [b] Interfacial tension

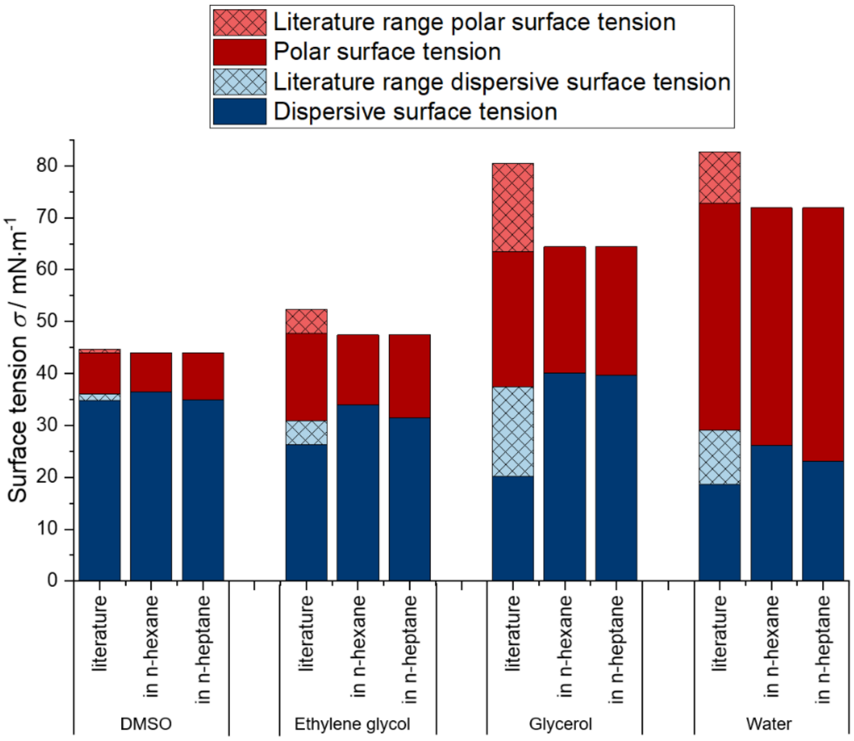

Figure S1. Measured dispersive and polar surface tension components of pure reference liquids in n-hexane and n-heptane were compared to the range of literature values accessible. The shaded region represents the literature spectrum from the minimum to maximum reported components. In general, the measured values fell towards the lower end of the spectrum in terms of polar contributions when compared to the literature values and to the higher end for dispersive contributions. Both the measurements in n-hexane and n-heptane exhibited trends consistent with those reported in the literature.

In general measurements in n-hexane and n-heptane yielded similar results. Especially the values for water and DMSO closely align with literature values. However, the measured values of the polar contributions of ethylene glycol and glycerol are lower than those reported in literature. In contrast the dispersive contribution of ethylene glycol and glycerol are slightly higher than those reported in literature. Notably, for these two component literature values vary significantly. One possible explanation could be that both glycerol and ethylene glycol are hygroscopic substances, and their polarity may increase with higher water content.

Additionally, the setup was tested at the start of each measurement day with deionized water. The measured values were in good agreement (±0.1) with the data published by Waldner et. al.[1]

Ionic liquids used in the study

Table S3. Ionic liquids used in the study

Cation	Anion	Abbreviation	Molecular weight/g·mol^{-1}	Molecular formular	CAS-No.
1-butyl-3-methylimidazolium	acrylate	[BMIM][ACR]	210.28	$C_{11}H_{18}N_2O_2$	-
1-butyl-3-methylimidazolium	acetate	[BMIM][OAc]	198.26	$C_{10}H_{18}N_2O_2$	284049-75-8
1-butyl-1-methylpyrrolidinium	bis(fluorosulfonyl)imide	[BMPyrr][FSI]	322.39	$C_8H_{21}F_2N_2O_4S_2$	1057745-51-3
cholinium	L-lysinate	[Chol][Lys]	249.35	$C_{11}H_{28}N_3O_3$	1361335-94-5
1,8-diazabicyclo[5.4.0]undec-7-enium	acetate	[DBUH][OAc]	212.29	$C_{11}H_{20}N_2O_2$	36443-65-9
1-ethyl-3-methylimidazolium	acrylate	[EMIM][ACR]	182.23	$C_9H_{14}N_2O_2$	-
1-ethyl-3-methylimidazolium	dicyanamide	[EMIM][DCA]	177.21	$C_8H_{11}N_5$	370865-89-7
1-ethyl-3-methylimidazolium	diethylphosphate	[EMIM][DEP]	264.26	$C_{10}H_{21}N_2O_4P$	848641-69-0
1-ethyl-3-methylimidazolium	bis(fluorosulfonyl)imide	[EMIM][FSI]	206.26	$C_6H_{11}F_2N_3O_4$	235789-75-0
1-ethyl-3-methylimidazolium	methanesulfonate	[EMIM][MeSO3]	170.21	$C_7H_{14}N_2O_3S$	145022-45-3
1-ethyl-3-methylimidazolium	acetate	[EMIM][OAc]	271.21	$C_8H_{14}N_2O_2$	143314-17-4
1-ethyl-3-methylimidazolium	octanoate	[EMIM][OOc]	184.24	$C_{14}H_{26}N_2O_2$	1154003-55-0
1-ethyl-3-methylimidazolium	propionate	[EMIM][OPr]	260.23	$C_9H_{16}N_2O_2$	865627-64-1
1-ethyl-3-methylimidazolium	trifluoromethanesulfonate	[EMIM][OTf]	169.25	$C_7H_{11}F_3N_2O_3S$	145022-44-2
1-ethyl-3-methylimidazolium	thiocyanat	[EMIM][SCN]	291.29	$C_7H_{11}N_3S$	331717-63-6
1-ethyl-3-methylimidazolium	bis(trifluoromethylsulfonyl)imide	[EMIM][TFSI]	391.31	$C_8H_{11}F_6N_3O_6S_2$	174899-82-2
1-hexyl-3-methylimidazolium	acrylate	[HEXMIM][ACR]	238.34	$C_{13}H_{22}N_2O_2$	-
pyrrolidinium	acetate	[Pyrr][OAc]	131.17	$C_6H_{13}NO_2$	35574-23-3
pyrrolidinium	formiate	[Pyrr][OFm]	117.15	$C_5H_{11}NO_2$	444810-12-2
triethylammonium	methanesulfonate	[TEAH][MeSO3]	197.29	$C_7H_{19}NO_3S$	93638-15-4

Density

Table S4. Density (ρ) at 25°C (±0.02°C) of ionic liquids in this study (±0.0005 g·cm^{-3}) measured at 101.3 kPa compared with literature values. The temperature of 25°C is only valid for the experimental data gathered in this study and is marked in light green. The experimental temperature for literature values is mentioned next to the data. M: method; VTD: vibrating-tube densiometer, RSD: rotational Stabinger viscometer-densimeter; nm = not mentioned; WB = Westphal balance; PM = pycnometer; CV = capillary viscometer; DM = density meter; OS = own made setup; BM = buoyancy method; DTV = double tube viscosmeter

	$\rho_{25°C}$ g·cm^{-3}	ρ g·cm^{-3}	Temp. K	M	Ref	ρ g·cm^{-3}	Temp. K	M	Ref
[BMIM][ACR]	1.0597								
[BMIM][OAC]	1.0555	1.0523	298.15	RSD	16	1.05263	298.15	VTD	17
		1.05270	298.15	VTD	18	1.0968	298.15	WB	19
		1.0532	298.15	VTD	20	1.05259	298.15	VTD	21
		1.0559	298.15	VTD	22	1.0538	293.15	RSD	23
		1.05223	298.15	VTD	24	1.0406	293.15	VTD	25
		1.053	298.15	PM	26	1.053	298.15	CV	27
		1.243	298.15	DM	28				
[BMpyrr][FSI]	1.3066								
[Chol][Lys]	1.1030								
[DBUH][OAC]	1.1065	1.0760	293.15	RSD	29				
[EMIM][ACR]	1.1083								
[EMIM][DCA]	1.0999	1.1040	298.15	RSD	30	1.101	298.15	RSD	31
		1.1046	293.15	VTD	32	1.1075	293.2	VTD	33
		1.10198	298.15	VTD	34	1.1008	298.15	VTD	35
		1.1058	303.2	RSD	36	1.10187	298.15	VTD	37
		1.10835	298.157	OS	38				
[EMIM][DEP]	1.1451	1.14840	298.15	VTD	39	1.1482	293.15	RSD	23
		1.1400	298.15	DM	40	1.146	298.15	VTD	41
		1.14892	298.15	VTD	42	1.1400	298.15	DM	40
		1.1464	298.15	PM	43	1.1386	313.15	PM	44
		1.1461	298.15	nm	45				
[EMIM][FSI]	1.4373	1.441	298.15	RSD	31				
[EMIM][$MeSO_3$]	1.2418	1.2424	298.15	RSD	30	1.2345	298.15	VTD	46
		1.2409	298.15	VTD	47	1.241	298.15	RSD	31
		1.24731	298.15	VTD	42	1.24152	298.15	VTD	48
		1.2345	298.15	VTD	49	1.2470	303.0	PM	50
		1.23996	298.15	VTD	51				
[EMIM][OAc]	1.1023	1.09778	298.15	VTD	34	1.0944	298.15	VTD	52
		1.102	298.15	nm	53	1.1088	298.15	VTD	22
		1.09826	298.15	VTD	17	1.09903	298.15	VTD	54
		1.09968	298.15	VTD	55	1.0983	298.15	RSD	56
		1.0993	298.15	RSD	30	1.0270	298.15	VTD	46
		1.09966	298.15	VTD	57	1.09902	298.15	VTD	18
		1.09902	298.15	VTD	39	1.097827	298.15	VTD	58
		1.09904	298.15	VTD	59	1.1008	298.15	RSD	60
		1.0983	298.15	VTD	47	1.1437	298.15	WB	19
[EMIM][OOc]	0.9960								
[EMIM][OPr]	1.0761								

[EMIM][OTf]	1.3810	1.3859	298.15	RSD	30	1.3773	303.2	RSD	36
		1.38358	298.15	VTD	61	1.38836	293.15	VTD	62
		1.3796	293.15	VTD	63	1.38593	297.94	BM	64
		1.38360	298.15	VTD	65	1.3818	303.15	VTD	66
		1.3829	293.15	VTD	67	1.38405	298.15	VTD	68
		1.3853	298.15	VTD	69				
[EMIM][SCN]	1.1159	1.1170	298.15	RSD	30	1.1161	298.15	VTD	47
		1.067	298.15	CV	27	1.118	298.15	DM	28
		1.115	298.15	RSD	31	1.11902	298.15	VTD	42
		1.11697	298.15	VTD	70	1.11556	298.155	BM	71
		1.11669	298.15	VTD	72				
[EMIM][TFSI]	1.5179	1.5147	298.15	VTD	20	1.5118	298.15		73
		1.5187	298.15		74	1.4409	293.15	RSD	23
		1.517	298.15	CV	27	1.5193	298.15	VTD	35
		1.52383	293.15	VTD	62	1.51838	298.15	VTD	68
		1.5168	298.15	DTV	75	1.51874	298.15	VTD	76
		1.5147	298.15	VTD	20	1.516391	298.15	VTD	77
		1.51874	298.15	VTD	78	1.51886	298.15	VTD	79
		1.51874	298.15	VTD	80	1.51891	298.15	VTD	81
		1.5250	293.15	VTD	82	1.5223	293.49	VTD	83
		1.51952	298.13	BM	84	1.5235	293.79	VTD	85
		1.526	293.15	VTD	86	1.45402	298.89	VTD	87
		1.5191	298.14	VTD	88	1.5192	298.15	VTD	89
		1.51776	298.15	VTD	90	1.5213	296.15	PM	91
		1.518251	298.15	VTD	92	1.51	303.15	nm	93
[HEXMIM][ACR]	1.0280								
[Pyrr][OAc]	1.0417	1.067	-	VTD	94				
[Pyrr][OFm]	1.0586	1.050	-	VTD	94				
[TEAH][MeSO₃]	1.1157	1.135	-	VTD	94	1.12	303.15	nm	95

Refractive index

Table S5. Refractive index (n_D) at 25°C (±0.03°C) of ionic liquids in this study (±0.0002) measured at 101.3 kPa compared with literature values. M: method

	n_D	n_D	T	Ref	n_D		Ref
[BMIM][ACR]	1.5062						
[BMIM][OAC]	1.4917	1.49488	298.15	17	1.49372	298.15	18
		1.48890	298.15	16	1.49381	298.15	20
[BMpyrr][FSI]	1.4448						
[Chol][Lys]	1.5060						
[DBUH][OAC]	1.5191						
[EMIM][ACR]	1.5135						
[EMIM][DCA]	1.5127	1.51428	298.15	30	1.5127	298.15	31
[EMIM][DEP]	1.4728	1.47304	298.15	39	1.4733	298.2	96
[EMIM][FSI]	1.4470	1.4474	298.15	31			
[EMIM][MeSO₃]	1.4957	1.49542	298.15	30	1.4771	298.15	46
		1.4958	298.15	31	1.4996	293.15	48

		1.4771	298.15	49			
[EMIM][OAc]	1.4982	1.49992	298.15	16	1.50001	298.15	17
		1.50069	298.15	97	1.5002	293.15	55
		1.49997	298.15	56	1.50091	298.15	30
		1.4771	298.15	46	1.50097	298.15	57
		1.50091	298.15	18	1.50069	298.15	39
[EMIM][OOc]	1.4847						
[EMIM][OPr]	1.4951						
[EMIM][OTf]	1.4349	1.43296	298.15	30	1.43322	298.15	61
		1.4330	293.15	62			
[EMIM][SCN]	1.5511	1.5514	298.15	31	1.53909	332.55	30
[EMIM][TFSI]	1.4229	1.4235	293.15	62	1.4220	298.15	75
		1.42307	298.15	76	1.42251	298.15	20
		1.42307	298.15	78	1.422860	298.15	77
		1.4219	297.256	98	1.42293	298.15	79
		1.42307	298.15	80	1.42298	298.15	81
[HEXMIM][ACR]	1.5001						
[Pyrr][OAc]	1.4627						
[Pyrr][OFm]	1.4621						
[TEAH][MeSO₃]	1.4609						

Surface tension

Table S6. Surface tension (σ) of ionic liquids in this study compared with literature values. Measurements have been conducted in a climatized room according to ISO 183 (23°C, 50% r.h., p = 101.3 kPa) M: method; PD: pendant drop method; DNR: Du Noüy ring method; PP: platinum plate method; FB: forced bubble method; CR: capillary rise method, FBP = falling ball principle.

	σ ***mN·m^{-1}***	**±**	***σ*** ***mN·m^{-1}***	***Temp.*** ***K***	***M***	***Ref***	***σ*** ***mN·m^{-1}***	***Temp.*** ***K***	***M***	***Ref***
[BMIM][ACR]	35.52	0.21								
[BMIM][OAC]	37.54	0.17	36.4	298.0	PD	16	39.2	298.15	PP	24
[BMpyrr][FSI]	45.13	0.43								
[Chol][Lys]	56.34	0.63								
[DBUH][OAC]	46.01	0.83	36.88	298.15	PD	29				
[EMIM][ACR]	45.27	0.18								
[EMIM][DCA]	57.70	0.42	56.4	298.2	PD	99	64.0	293	DNR	100
			60.0	298.31	PP	64	42.6	295	CR	101
			60.62	298.30	DNR	64	40.3	298.15	DNR	34
[EMIM][DEP]	35.35	0.25	37.1	298.15	PP	102	35.29	293.15	DNR	103
			35.88	298.15	PP	40	34.46	298.15	DNR	41
[EMIM][FSI]	48.55	0.52								
[EMIM][MeSO₃]	46.83	0.35	50.72	293.15	PD	48	41.3	298.2	PD	104
			47.8	303	PD	50	45.1	298.15	PP	49
			45.1	298.15	PP	46				
[EMIM][OAc]	48.37	1.02	42.9	298.15	DNR	34	47.1	298.15	PP	97
			47.1	298.1	PD	104	45.72	298.15	PD	105
[EMIM][OOc]	31.09	0.96								

[EMIM][OPr]	40.74	0.43	39.6	298.15	FB	[106]				
[EMIM][OTf]	40.57	0.43	39.2	298	CR	[101]	44.46	296	DNR	[107]
			41.3	298.15	PD	[104]	40.43	298.01	PP	[108]
[EMIM][SCN]	59.94	0.40	49.0	295	CR	[101]	57.8	298	DNR	[109]
			53.3	298.2	PD	[104]	55.3	298.15	FB	[110]
			53.1	298.15	PP	[111]	54.1	298.15	FBP	[70]
[EMIM][TFSI]	35.98	0.37	35.2	295	CR	[101]	35.71	298.15	PD	[74]
			35.6	298.15	PD	[112]	35.59	293.15	PD	[73]
			36.94	298.15	DNR	[113]	36.12	297.953	DNR	[114]
			41.6	298	DNR	[107]	38.0	297	DNR	[115]
			39.4	298.15	DNR	[75]	36.45	298.06	PP	[116]
			35.90	298.15	PD	[117]				
[HEXMIM][ACR]	31.25	0.93								
[Pyrr][OAc]	44.53	0.58								
[Pyrr][OFm]	44.53	0.54								
[TEAH][MeSO3]	44.85	0.65								

Table S7. Measured surface tension values and their deviations. Measurements have been conducted in a climatized room according to ISO 183 (23°C, 50% r.h., p = 101.3 kPa)

	$\sigma_{L,tot}$[a] *mN·m^{-1}*		$\sigma_{L,d}$[b] *mN·m^{-1}*		$\sigma_{L,p}$[c] *mN·m^{-1}*	
[BMIM][ACR]	35.52	±0.47	29.20	±0.21	6.32	±0.26
[BMIM][OAC]	37.54	±0.38	30.69	±0.17	6.85	±0.21
[BMPyrr][FSI]	45.13	±0.89	34.40	±0.43	10.73	±0.46
[Chol][Lys]	56.34	±1.33	41.92	±0.63	14.42	±0.70
[DBUH][OAC]	46.01	±1.74	39.50	±0.83	6.51	±0.91
[EMIM][ACR]	45.27	±0.39	33.87	±0.18	11.40	±0.21
[EMIM][DCA][c]	57.70	±0.85	39.15	±0.42	18.55	±0.43
[EMIM][DEP]	35.35	±0.56	29.27	±0.25	6.08	±0.31
[EMIM][MeSO3]	48.55	±1.23	18.52	±0.52	30.03	±0.71
[EMIM][FSI]	46.83	±0.72	33.86	±0.35	12.97	±0.37
[EMIM][OAc]	48.37	±2.07	35.08	±1.02	13.29	±1.05
[EMIM][OOc]	31.09	±2.01	30.35	±0.96	0.74	±1.05
[EMIM][OPr]	40.74	±0.97	32.26	±0.43	8.48	±0.54
[EMIM][OTf]	40.57	±0.98	26.27	±0.43	14.30	±0.55
[EMIM][SCN]	59.94	±0.84	41.14	±0.40	18.80	±0.44
[EMIM][TFSI]	35.98	±0.82	24.51	±0.37	11.47	±0.45
[HEXMIM][ACR]	31.25	±1.91	28.10	±0.93	3.15	±0.98
[Pyrr][OAc][c]	39.77	±1.26	34.34	±0.58	5.43	±0.68
[Pyrr][OFm][c]	44.53	±1.18	40.86	±0.54	3.67	±0.64
[TEAH][MeSO3][c]	44.85	±1.40	33.35	±0.65	11.5	±0.75

[a]Total surface tension, [b]dispersive contribution of the surface tension, [c]polar contribution of the surface tension

Water content

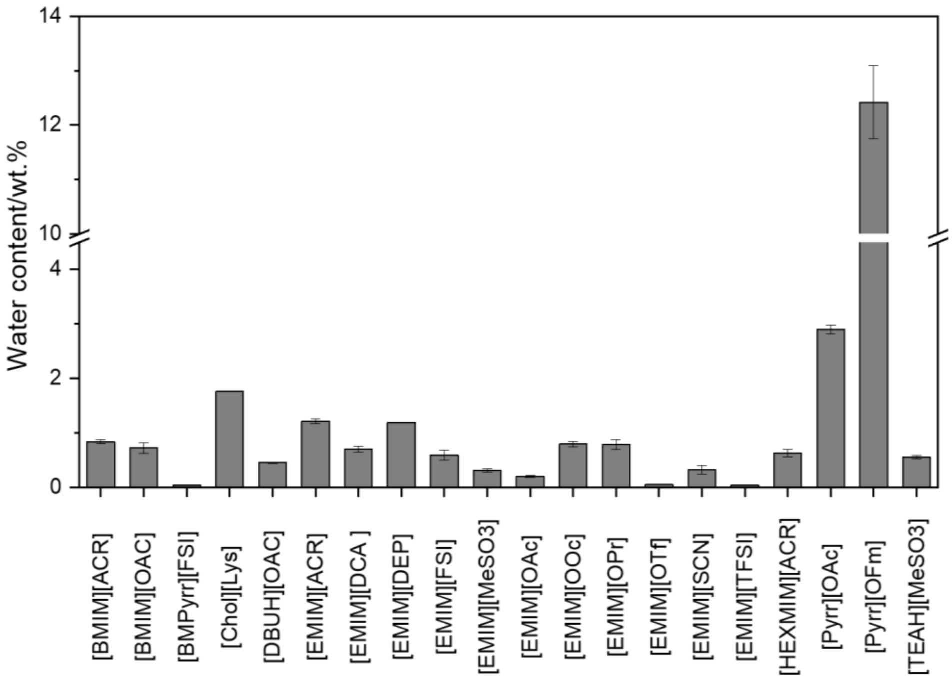

Figure S2. water content of the ionic liquids, directly from the CBILs® process measured by Karl-Fischer titration.

Relationship ion pair volume and dispersive ratio of ionic liquids

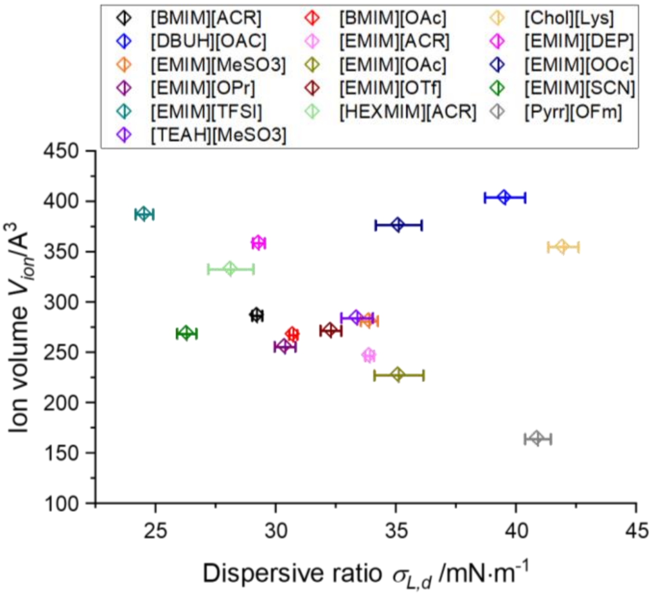

Figure S3. Ion Volume vs. dispersive ratio of the ionic liquids in question. No clear trend can be observed between the two variables.

Rheology temperature vs. shear rate

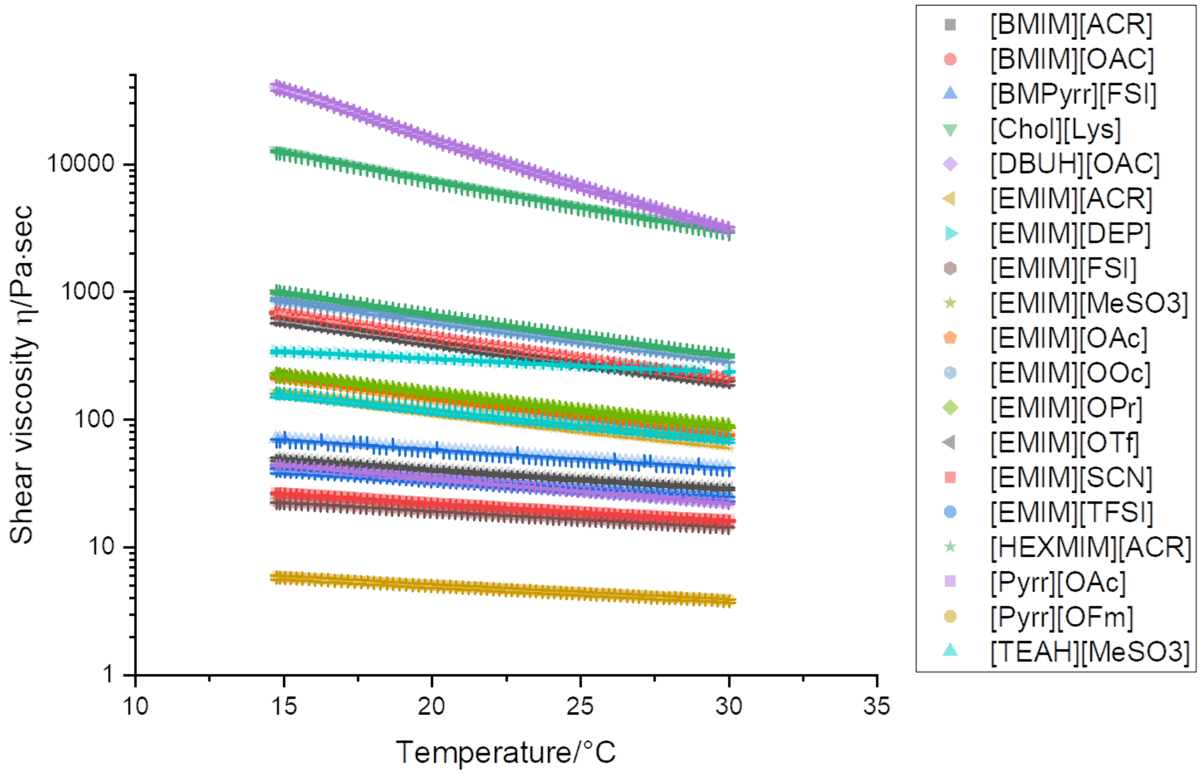

Figure S4. Temperature dependent shear viscosity η at a constant shear rate of 50 s^{-1} ranging from 15°C to 30°C. Displayed values are the average of three independent measurements and their deviations.

Measurement data of figure S3 and figure 5 can be taken from table S8 and table S9.

Rheology literature review

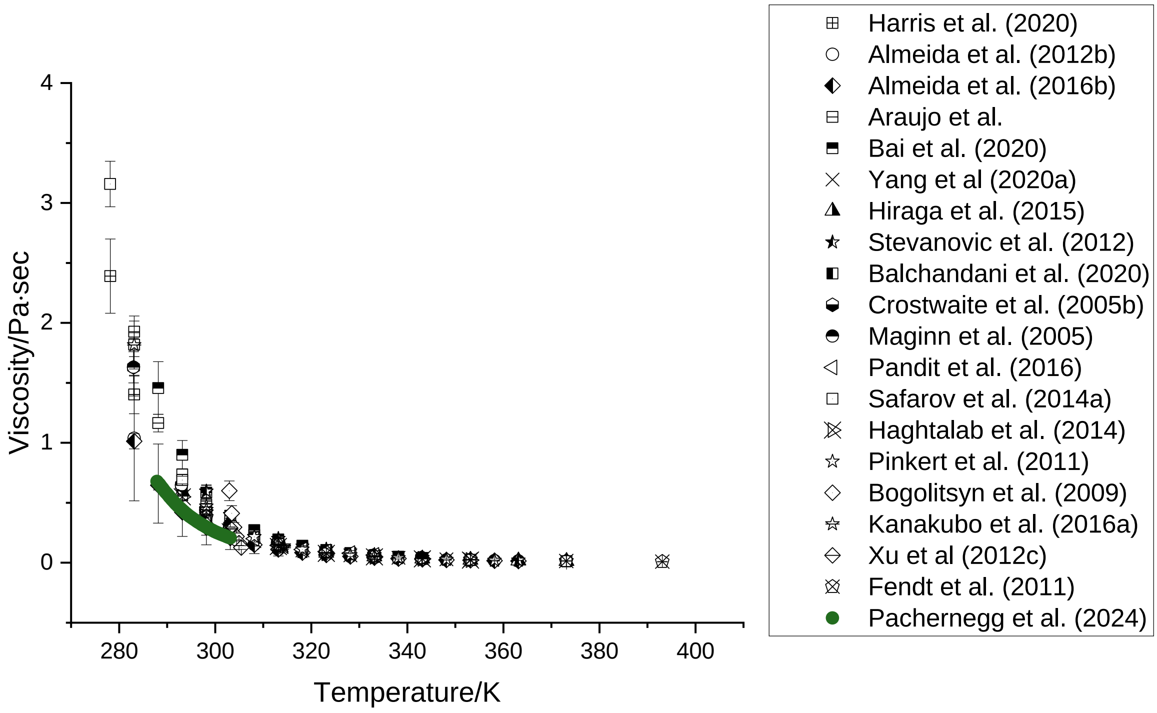

Figure S5. Literature review on the viscosity of [BMIM][OAc]. [16, 17, 21-25, 118-129]

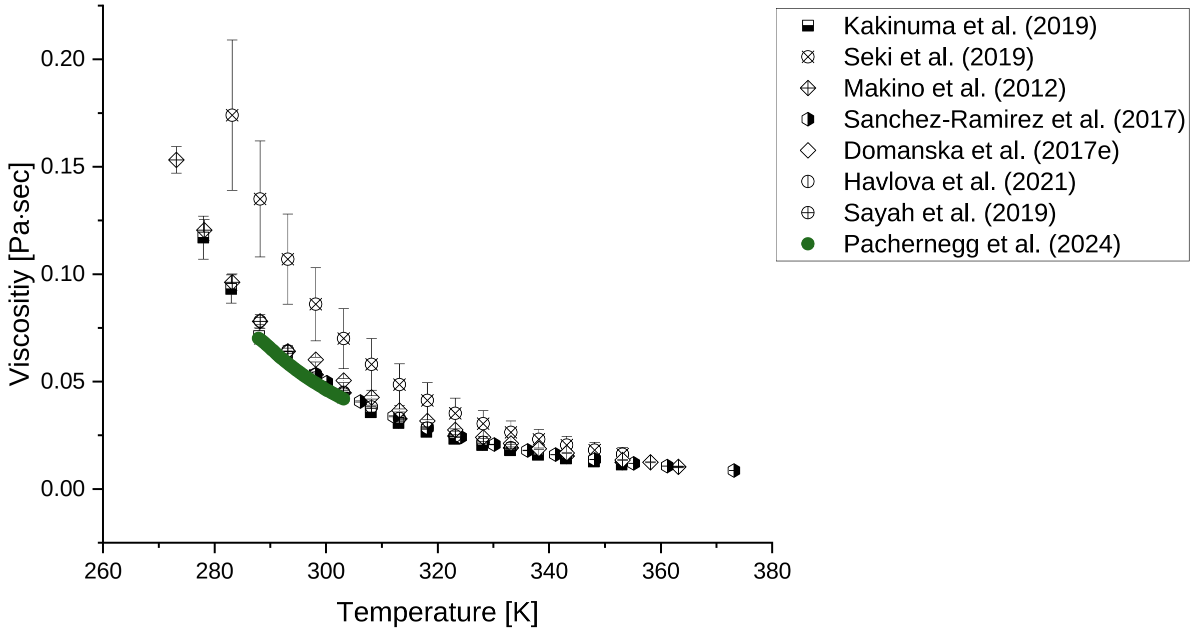

Figure S6. Literature review on the viscosity of [BMPyrr][FSI]. [130-136]

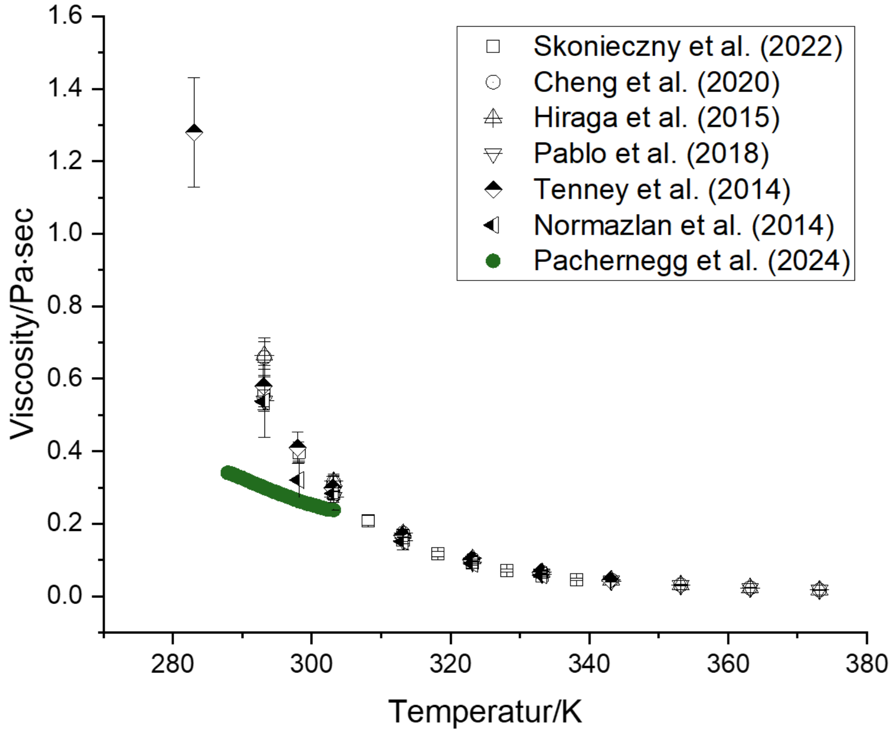

Figure S7. Literature review on the viscosity of [EMIM][DEP]. [23, 41, 137-140]

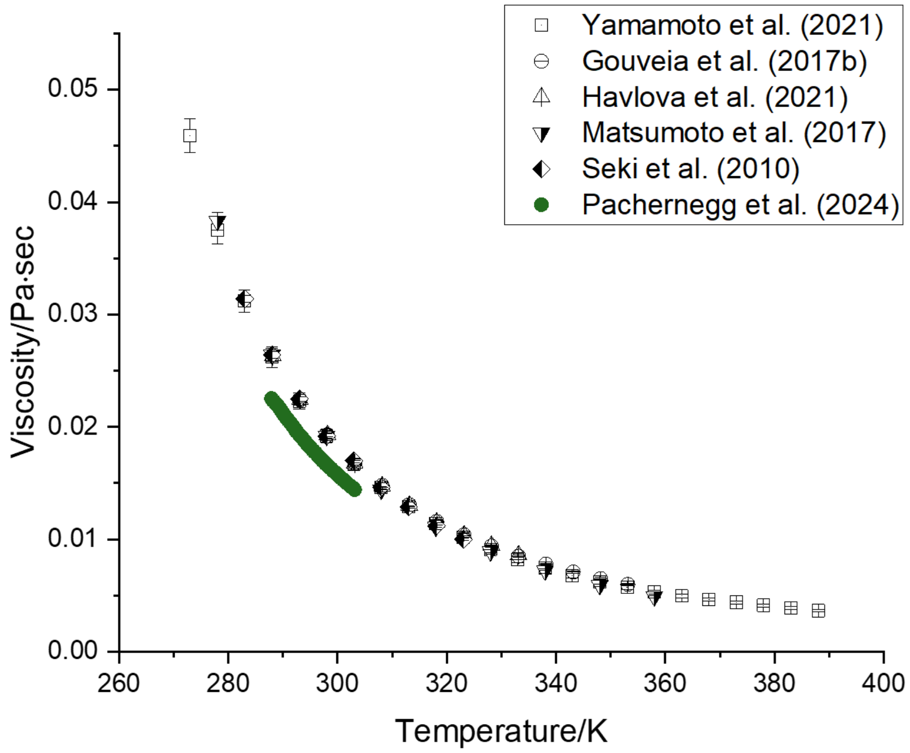

Figure S8. Literature review on the viscosity of [EMIM][FSI].[141-144]

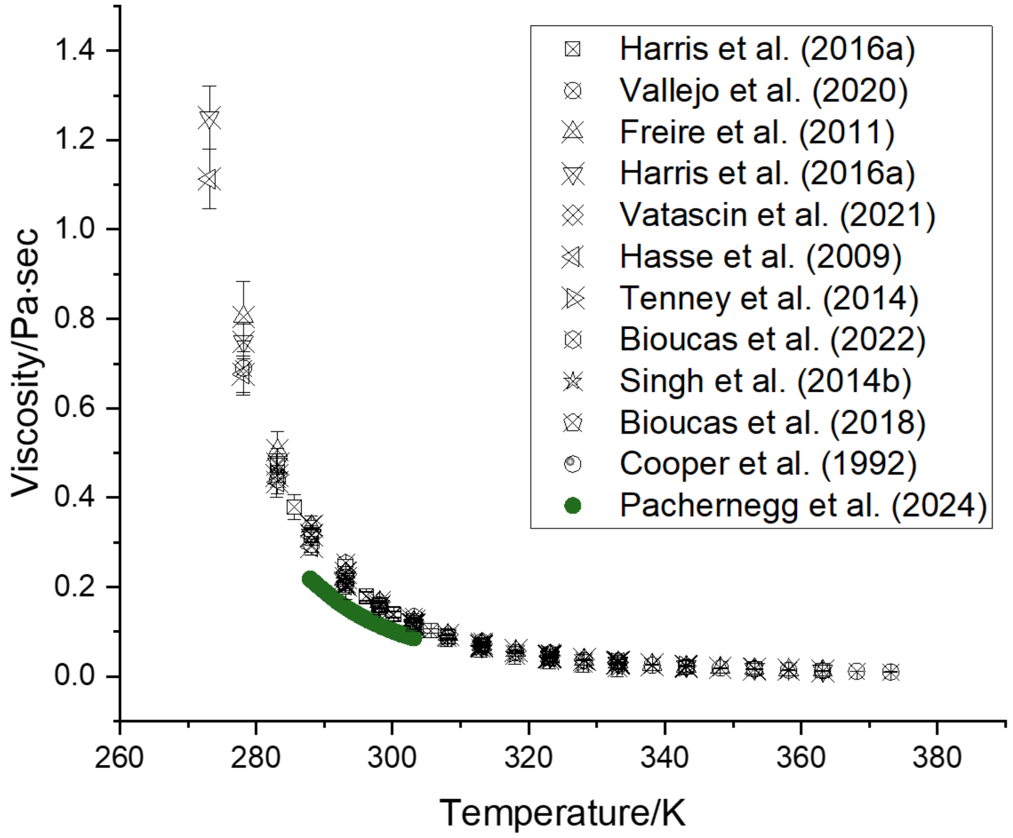

Figure S9. Literature review on the viscosity of [EMIM][MeSO3].[30, 48, 50, 140, 145-150]

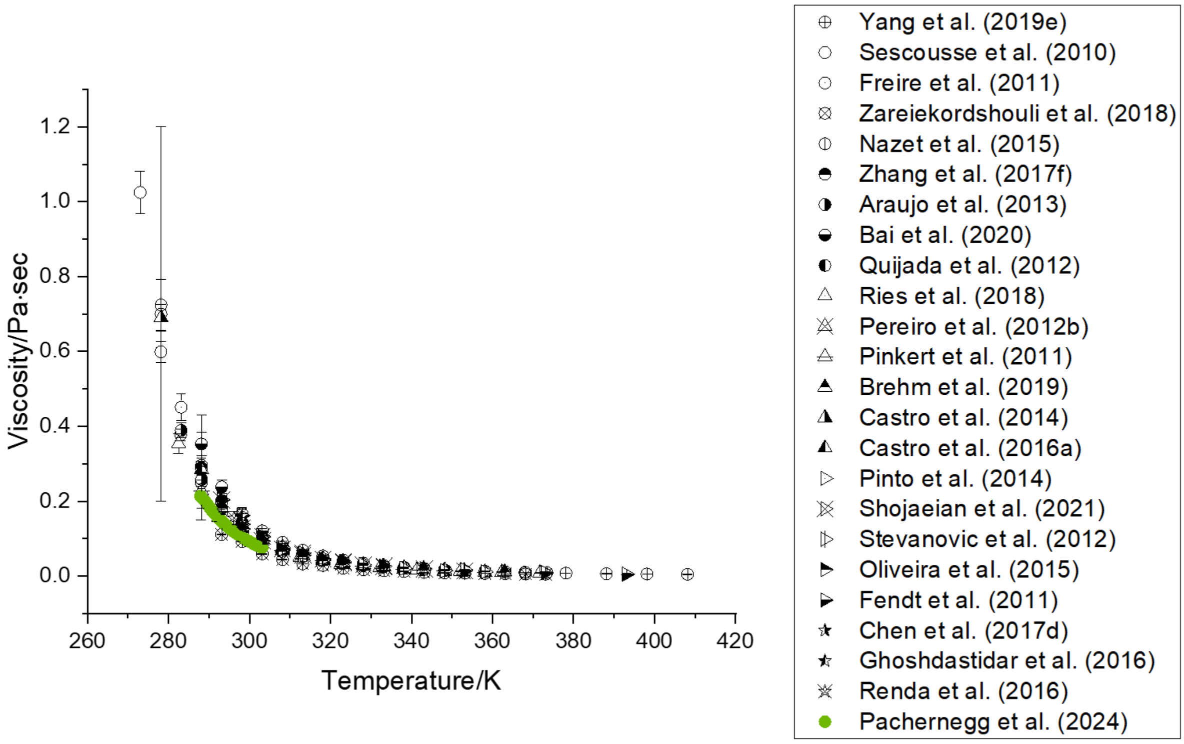

Figure S10. Literature review on the viscosity of [EMIM][OAc].[17, 22, 30, 34, 52, 54, 56, 59, 60, 97, 120, 122, 129, 151-160]

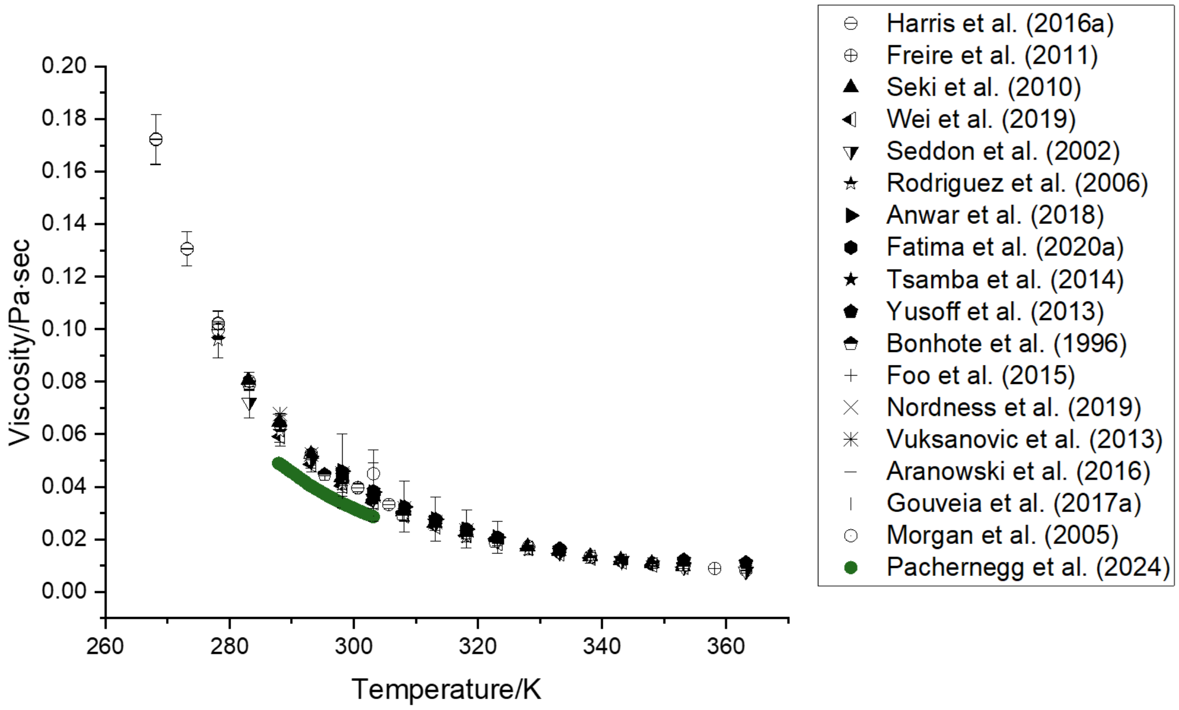

Figure S11. Literature review on the viscosity of [EMIM][OTf]. [30, 65-68, 144, 145, 161-170]

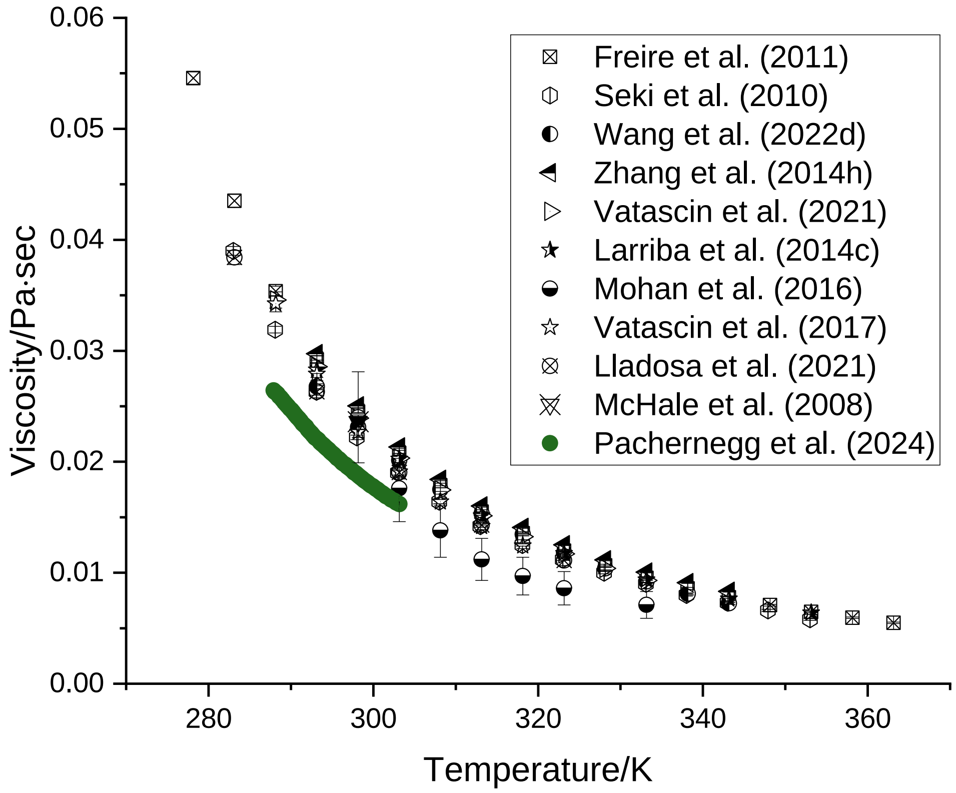

Figure S12. Literature review on the viscosity of [EMIM][SCN].[28, 30, 70, 110, 144, 147, 171-174]

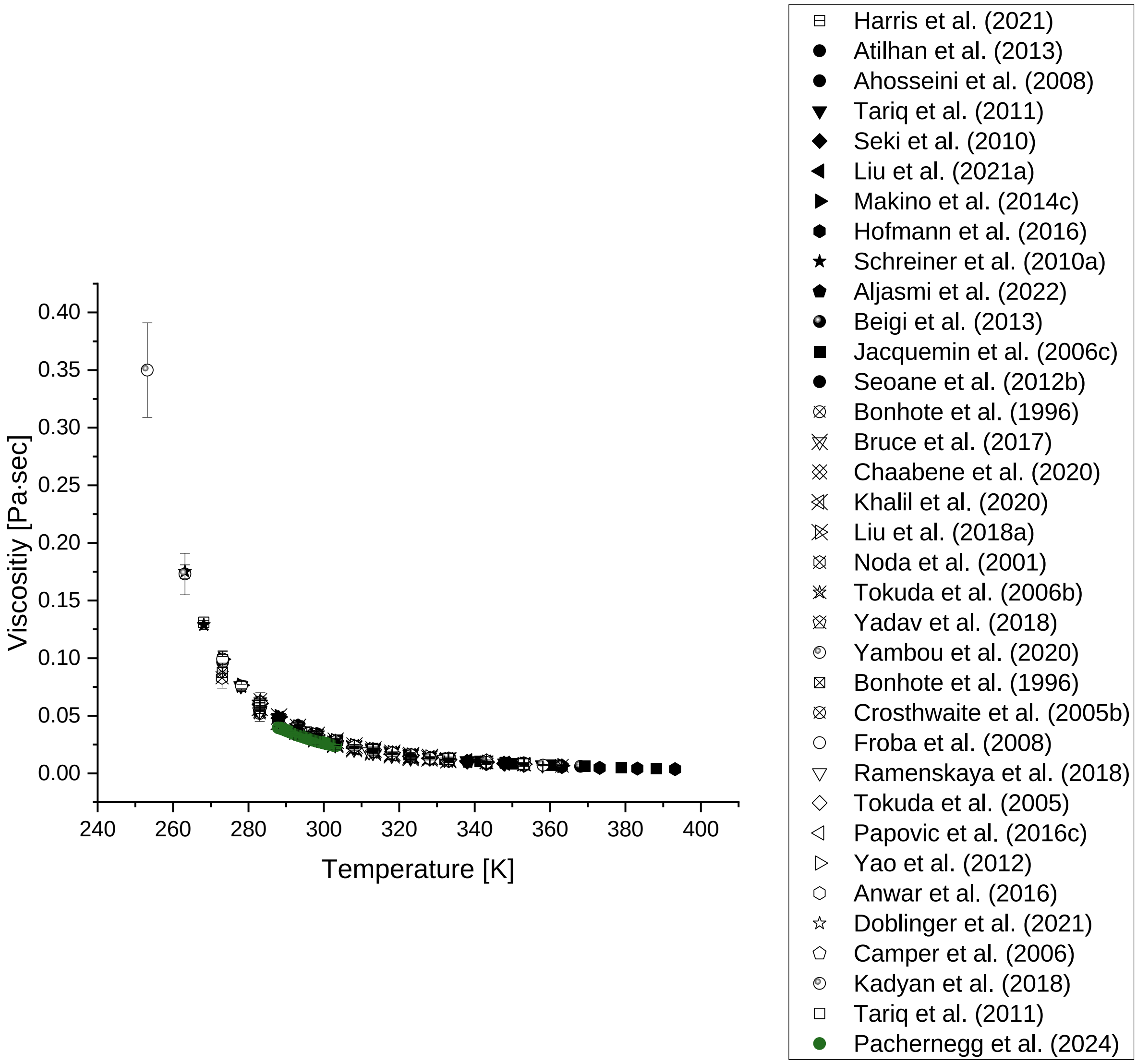

Figure S13. Literature review on the viscosity of [EMIM][TFSI]. Only literature data with more than five datapoints is shown. 35, 73, 75, 78, 85, 92, 93, 124, 144, 165, 175-196

Rheology data

Table S8. Rheology measurement data at constant shear rate measurements (η = 50s^{-1}) and the deviations of the three individual measurements (±). Measurements were conducted in an ambient atmosphere at 101.3 kPa. u_{95} – standard deviation with a confidence interval of 0.95.

[BMIM][ACR]				[BMIM][OAC]				[BMPyrr][FSI]			
T/°C	Shear viscosity η/mPa·sec	Deviation between measure-ments ±	u_{95}	*T*/°C	Shear viscosity η/mPa·sec	Deviation between measure-ments ±	u_{95}	*T*/°C	Shear viscosity η/mPa·sec	Deviation between measure-ments ±	u_{95}
14.75	595.1267	1.4175	28.3486	14.75	678.7800	0.6305	12.6093	14.75	70.1127	0.0069	0.1372
14.87	589.3333	1.4454	28.9143	14.87	670.9233	0.6258	12.5170	14.87	69.9223	0.0032	0.0638
15.03	582.7200	1.3933	27.8689	15.04	663.2933	0.5912	11.8241	15.03	69.6120	0.0028	0.0558
15.21	577.0333	1.3742	27.4818	15.22	655.9833	0.5687	11.3738	15.21	69.2310	0.0028	0.0564
15.41	569.7467	1.3976	27.9571	15.42	646.1900	0.5739	11.4662	15.41	68.8223	0.0029	0.0580
15.62	560.0167	1.3429	26.8608	15.62	634.6867	0.5398	10.7965	15.61	68.4223	0.0029	0.0587
15.83	550.8800	1.3194	26.3942	15.83	624.2200	0.5011	10.0286	15.83	67.9497	0.0025	0.0490
16.04	543.2167	1.3408	26.8146	16.05	612.8967	0.5017	10.0343	16.04	67.4383	0.0031	0.0626
16.26	533.2733	1.2757	25.5193	16.26	600.5633	0.5075	10.1614	16.26	66.9033	0.0035	0.0688
16.48	523.4333	1.2405	24.8066	16.49	589.1333	0.4601	9.2087	16.48	66.4367	0.0023	0.0460
16.70	515.0200	1.2517	25.0290	16.71	579.5833	0.4977	9.9535	16.70	65.8967	0.0024	0.0473
16.93	507.1133	1.2158	24.3217	16.93	569.5533	0.5133	10.2653	16.92	65.3463	0.0029	0.0577
17.15	497.3933	1.1636	23.2655	17.15	558.5333	0.4324	8.6545	17.14	64.7903	0.0026	0.0512
17.37	489.1933	1.1761	23.5317	17.37	548.7200	0.4238	8.4697	17.37	64.4227	0.0036	0.0700
17.59	480.8733	1.1441	22.8814	17.59	538.7833	0.4832	9.6591	17.59	63.8770	0.0030	0.0597
17.81	471.6733	1.0843	21.6776	17.81	527.8933	0.4099	8.2041	17.80	63.3067	0.0024	0.0480
18.03	463.6100	1.0967	21.9316	18.03	518.4533	0.3903	7.8058	18.03	62.7747	0.0019	0.0376
18.25	455.8367	1.0811	21.6194	18.25	509.1900	0.4209	8.4178	18.24	62.2373	0.0018	0.0355
18.46	447.5100	1.0316	20.6293	18.47	499.0500	0.4024	8.0367	18.46	61.7147	0.0023	0.0458

18.68	439.9867	1.0240	20.4860	18.68	490.0233	0.3672	7.3381	18.67	61.2007	0.0020	0.0413
18.89	432.9767	1.0205	20.4148	18.90	481.6867	0.3862	7.7307	18.89	60.7870	0.0014	0.0288
19.11	424.8400	0.9683	19.3687	19.11	472.2633	0.3868	7.7365	19.10	60.4230	0.0011	0.0211
19.32	417.6200	0.9403	18.8075	19.33	464.0633	0.3556	7.1130	19.32	59.9240	0.0016	0.0308
19.54	409.9600	0.9516	19.0345	19.54	456.7367	0.3487	6.9686	19.53	59.4460	0.0011	0.0217
19.75	402.7800	0.9131	18.2638	19.75	448.3700	0.3632	7.2573	19.74	59.0133	0.0005	0.0101
19.96	395.5067	0.8826	17.6544	19.97	439.9933	0.3297	6.5933	19.95	58.5490	0.0016	0.0312
20.17	390.5733	0.9049	18.1024	20.18	433.1567	0.3222	6.4490	20.17	58.0957	0.0012	0.0225
20.38	384.9600	0.8733	17.4565	20.39	425.7600	0.3447	6.8936	20.37	57.6577	0.0011	0.0222
20.59	378.0867	0.8330	16.6699	20.60	417.8667	0.3227	6.4490	20.59	57.2783	0.0001	0.0015
20.80	372.2467	0.8346	16.6827	20.81	411.1167	0.3020	6.0506	20.79	56.8837	0.0012	0.0245
21.01	367.3700	0.8214	16.4306	21.01	404.3367	0.3002	5.9987	21.00	56.4627	0.0005	0.0086
21.22	360.7533	0.7887	15.7747	21.22	396.8567	0.3118	6.2238	21.21	56.0380	0.0007	0.0131
21.43	354.9700	0.7797	15.5923	21.43	390.3067	0.2858	5.7215	21.42	55.6310	0.0014	0.0287
21.63	349.8767	0.7784	15.5665	21.64	384.3933	0.2916	5.8312	21.63	55.2030	0.0008	0.0165
21.84	344.5867	0.7523	15.0383	21.85	378.9200	0.3320	6.6338	21.83	54.8027	0.0005	0.0101
22.05	338.6667	0.7293	14.5880	22.05	372.8567	0.2777	5.5483	22.04	54.4030	0.0011	0.0214
22.25	333.8267	0.7315	14.6294	22.26	367.2600	0.2719	5.4386	22.25	54.0140	0.0010	0.0200
22.46	328.6600	0.7112	14.2243	22.47	361.2733	0.2875	5.7446	22.46	53.6220	0.0005	0.0108
22.67	323.5733	0.6760	13.5203	22.67	355.3833	0.2737	5.4848	22.66	53.2287	0.0009	0.0180
22.87	318.4333	0.6800	13.6000	22.88	349.9867	0.2575	5.1500	22.87	52.8427	0.0012	0.0235
23.08	313.7800	0.6690	13.3716	23.08	344.5967	0.2708	5.4271	23.07	52.4993	0.0004	0.0075
23.28	308.6100	0.6356	12.7180	23.29	338.6933	0.2627	5.2597	23.28	52.1630	0.0005	0.0090
23.49	304.4167	0.6430	12.8562	23.49	333.7333	0.2558	5.1211	23.48	51.7830	0.0013	0.0263
23.69	300.1433	0.6436	12.8764	23.70	328.9033	0.2494	4.9825	23.69	51.4197	0.0006	0.0133
23.90	295.3300	0.6182	12.3519	23.90	323.3467	0.2529	5.0634	23.89	51.0620	0.0003	0.0046
24.10	290.7100	0.6043	12.0929	24.11	318.1200	0.2338	4.6765	24.10	50.7080	0.0010	0.0185
24.31	286.7467	0.6047	12.0851	24.31	313.8100	0.2419	4.8324	24.30	50.3520	0.0009	0.0190
24.51	282.0800	0.5856	11.7122	24.52	308.4000	0.2344	4.6939	24.51	49.9980	0.0001	0.0017

24.71	277.5533	0.5662	11.3280	24.72	303.2333	0.2252	4.4976	24.71	49.6583	0.0007	0.0127
24.92	273.6967	0.5698	11.3894	24.92	298.8000	0.2165	4.3301	24.92	49.4333	0.0012	0.0250
25.12	270.2033	0.5683	11.3657	25.13	294.5733	0.2413	4.8266	25.12	49.0910	0.0003	0.0060
25.33	266.4633	0.5379	10.7640	25.33	289.3633	0.2148	4.2897	25.32	48.7427	0.0006	0.0117
25.53	262.6267	0.5377	10.7533	25.54	285.0300	0.2038	4.0703	25.53	48.4030	0.0011	0.0210
25.73	259.0367	0.5347	10.6986	25.74	280.9200	0.2113	4.2262	25.73	48.1263	0.0008	0.0170
25.94	255.1633	0.5132	10.2623	25.94	276.8200	0.2298	4.5899	25.93	47.7903	0.0004	0.0081
26.14	251.4867	0.5044	10.0830	26.15	272.6800	0.1969	3.9318	26.14	47.4607	0.0010	0.0195
26.34	248.1600	0.5054	10.0995	26.35	268.8433	0.1957	3.9087	26.34	47.1373	0.0007	0.0150
26.55	244.4900	0.4873	9.7415	26.55	264.6833	0.2009	4.0126	26.54	46.7163	0.0004	0.0070
26.75	240.8867	0.4761	9.5265	26.75	260.6833	0.1911	3.8221	26.75	46.3873	0.0009	0.0163
26.95	237.9967	0.4803	9.6006	26.96	257.0833	0.1801	3.5969	26.95	46.0687	0.0010	0.0202
27.16	234.6333	0.4688	9.3786	27.16	253.2600	0.1917	3.8278	27.15	45.7633	0.0006	0.0124
27.36	231.0967	0.4486	8.9764	27.37	249.2500	0.1824	3.6373	27.36	45.6190	0.0010	0.0204
27.57	228.0133	0.4580	9.1610	27.57	245.9833	0.1842	3.6835	27.56	45.3137	0.0015	0.0291
27.77	224.9633	0.4559	9.1211	27.77	242.9833	0.1894	3.7874	27.76	45.0153	0.0013	0.0270
27.97	221.5800	0.4382	8.7642	27.98	239.2533	0.1801	3.5969	27.97	44.7177	0.0013	0.0261
28.17	218.4933	0.4353	8.7099	28.18	235.7833	0.1680	3.3544	28.17	44.4490	0.0019	0.0383
28.38	215.6933	0.4348	8.7053	28.38	232.7933	0.1801	3.5969	28.37	44.1553	0.0018	0.0350
28.58	212.7033	0.4121	8.2393	28.59	229.3867	0.1767	3.5392	28.58	43.8673	0.0012	0.0250
28.78	209.6133	0.4066	8.1310	28.79	225.9800	0.1640	3.2736	28.78	43.5767	0.0016	0.0329
28.99	206.9833	0.4086	8.1681	29.00	222.9800	0.1628	3.2563	28.98	43.3097	0.0021	0.0418
29.19	204.1467	0.3974	7.9426	29.20	219.8633	0.1732	3.4583	29.19	43.0140	0.0014	0.0284
29.39	201.3633	0.3769	7.5416	29.40	216.0633	0.1432	2.8521	29.39	42.7310	0.0017	0.0330
29.60	198.7433	0.3766	7.5388	29.60	212.9833	0.1513	3.0253	29.59	42.4553	0.0019	0.0382
29.80	196.1233	0.3731	7.4610	29.81	210.0633	0.1593	3.1812	29.80	42.1883	0.0014	0.0281
30.00	193.3033	0.3580	7.1598	30.01	206.9233	0.1542	3.0773	30.00	41.9080	0.0015	0.0295

**[Chol][Lys]**				**[DBUH][OAC]**				**[EMIM][ACR]**			

T/°C	Shear viscosity η/mPa·sec	Deviation between measure-ments ±	u_{95}	*T*/°C	Shear viscosity η/mPa·sec	Deviation between measure-ments ±	u_{95}	*T*/°C	Shear viscosity η/mPa·sec	Deviation between measure-ments ±	u_{95}
14.75	12654.3333	10.0677	201.3761	14.75	39907.6667	122.6249	2451.9364	14.75	158.7533	0.5357	10.7197
14.87	12490.0000	10.1908	203.6566	14.86	39144.0000	122.8188	2456.9365	14.87	157.5833	0.5243	10.4874
15.03	12357.0000	10.3867	207.7667	15.02	38386.0000	119.9695	2399.3908	15.04	156.3067	0.5232	10.4672
15.21	12147.3333	8.6685	173.3705	15.20	37368.3333	107.9418	2158.8352	15.22	154.8867	0.5163	10.3269
15.41	11915.3333	9.2344	184.9928	15.40	36189.3333	105.7556	2115.1126	15.42	153.0833	0.5034	10.0732
15.61	11700.0000	9.5424	190.8193	15.60	35066.6667	106.6653	2133.3067	15.62	151.2000	0.4997	9.9940
15.82	11453.3333	8.1526	163.1850	15.81	33856.0000	96.5730	1931.5670	15.83	149.5333	0.4933	9.8676
16.04	11167.0000	8.1472	163.2207	16.03	32548.6667	90.1880	1803.8870	16.05	147.1433	0.4768	9.5374
16.25	10931.0000	9.0586	181.3643	16.25	31335.3333	91.1869	1823.1984	16.27	145.1533	0.4731	9.4613
16.48	10683.0000	7.9863	159.6152	16.47	30178.3333	86.0936	1721.8715	16.49	143.0900	0.4680	9.3637
16.70	10440.6667	8.1481	162.9305	16.69	28921.3333	78.3108	1565.6757	16.71	141.2167	0.4550	9.1032
16.92	10221.0000	8.3167	166.2318	16.91	27727.0000	77.5534	1550.6105	16.93	138.6633	0.4474	8.9508
17.14	9995.5333	7.9172	158.6192	17.13	26667.3333	74.4979	1489.5235	17.15	136.8300	0.4425	8.8464
17.36	9743.4667	6.8998	137.9399	17.35	25566.3333	67.6797	1353.5942	17.37	134.7133	0.4335	8.6721
17.58	9525.9333	7.5101	150.1232	17.57	24481.3333	65.2209	1304.4870	17.59	132.5400	0.4201	8.4040
17.80	9322.9333	7.8521	157.0427	17.79	23519.3333	64.5092	1290.6674	17.81	130.6533	0.4185	8.3693
18.02	9095.3333	6.4821	129.6987	18.01	22598.3333	59.1901	1184.3210	18.03	128.7867	0.4095	8.1904
18.24	8882.4000	7.0963	141.9638	18.23	21631.6667	56.0728	1121.4055	18.25	126.9767	0.3995	7.9895
18.46	8696.6000	7.4009	148.0737	18.44	20778.6667	56.2141	1124.7686	18.46	125.1333	0.3946	7.8937
18.67	8498.5667	6.3107	126.2532	18.66	19988.3333	51.7431	1035.2707	18.68	123.6633	0.3899	7.7993
18.89	8292.9000	6.1518	123.0552	18.88	19175.6667	47.9828	959.6355	18.89	121.5500	0.3781	7.5622
19.10	8125.8333	7.2610	145.1638	19.09	18403.3333	48.6107	972.0094	19.11	119.8833	0.3724	7.4485
19.31	7960.5000	6.1972	123.9638	19.30	17732.3333	46.1514	923.0305	19.32	118.1867	0.3693	7.3834
19.53	7773.7000	5.5018	110.0364	19.52	17035.6667	41.7632	835.1565	19.53	116.6600	0.3597	7.1988

19.74	7607.2000	6.2662	125.2513	19.73	16349.3333	41.9009	837.9119	19.75	114.7367	0.3537	7.0756
19.95	7459.9667	6.1967	123.9349	19.94	15757.3333	41.1061	822.1267	19.96	112.9267	0.3479	6.9601
20.16	7292.5667	5.1310	102.6757	20.15	15182.0000	36.0863	721.5255	20.17	111.6500	0.3419	6.8367
20.37	7133.7333	5.6128	112.2177	20.36	14595.6667	33.8280	676.4631	20.38	110.1800	0.3336	6.6731
20.58	6999.1000	5.8510	117.0582	20.57	14096.3333	35.2995	706.1851	20.59	108.8533	0.3309	6.6190
20.79	6851.9000	4.9352	98.7191	20.78	13620.6667	32.7581	655.2605	20.80	107.3267	0.3265	6.5316
21.00	6695.5333	5.0156	100.3118	20.99	13103.3333	30.4224	608.1450	21.01	105.9967	0.3175	6.3486
21.21	6570.6333	5.5745	111.4900	21.20	12629.3333	31.2114	624.0267	21.22	104.3883	0.3138	6.2764
21.42	6441.0333	4.8472	96.9217	21.40	12221.0000	29.7305	594.7294	21.42	103.1303	0.3097	6.1927
21.62	6307.4333	4.7835	95.6703	21.61	11772.0000	26.9052	538.0028	21.63	101.8917	0.3039	6.0781
21.83	6200.3667	5.1920	103.8201	21.82	11340.3333	27.3650	547.5001	21.84	100.2370	0.2960	5.9195
22.04	6088.6667	4.8493	97.0815	22.02	10974.6667	26.3520	526.7393	22.05	99.1637	0.2945	5.8890
22.24	5957.2333	4.1245	82.4903	22.23	10600.3333	23.6663	473.5254	22.25	97.6983	0.2877	5.7537
22.45	5843.4667	4.8272	96.6008	22.44	10211.8333	23.8164	476.2768	22.46	96.5110	0.2815	5.6286
22.66	5741.8333	4.6606	93.2129	22.64	9879.6333	23.5038	470.2230	22.67	95.2240	0.2804	5.6077
22.86	5623.7333	3.8643	77.2855	22.85	9545.0333	21.5394	430.6882	22.87	94.1250	0.2752	5.5048
23.07	5506.9000	4.0990	81.9807	23.06	9199.3667	20.8586	417.1223	23.08	93.0000	0.2690	5.3809
23.27	5412.9000	4.5722	91.3860	23.26	8881.1667	21.3707	427.4150	23.28	91.8467	0.2660	5.3215
23.48	5312.7000	3.8339	76.6961	23.47	8599.8333	19.4473	388.8968	23.48	90.9257	0.2639	5.2778
23.68	5200.3333	3.7706	75.3934	23.67	8291.7000	18.0548	361.0477	23.69	89.5603	0.2570	5.1398
23.89	5105.8333	4.2359	84.6608	23.88	8008.8000	18.7352	374.8040	23.90	88.5163	0.2536	5.0726
24.09	5015.4667	3.9179	78.4528	24.08	7754.7333	17.8620	357.1905	24.10	87.4770	0.2520	5.0408
24.30	4910.9000	3.2395	64.8306	24.29	7496.1000	16.0021	320.0422	24.31	86.4750	0.2464	4.9268
24.50	4815.3333	3.9819	79.6185	24.49	7234.4667	16.0915	321.8326	24.51	85.4053	0.2428	4.8563
24.71	4731.1000	3.9165	78.3678	24.70	7012.5333	16.1127	322.2033	24.72	84.1473	0.2392	4.7829
24.91	4636.6000	3.3797	67.5758	24.90	6784.4667	14.3353	286.7056	24.92	83.3123	0.2359	4.7187
25.11	4546.6667	3.5481	70.9260	25.10	6548.0333	14.0280	280.6069	25.12	82.0030	0.2305	4.6093
25.32	4472.0667	3.6479	73.0158	25.30	6341.4000	14.1800	283.6000	25.33	81.0893	0.2276	4.5512
25.52	4386.2333	3.0654	61.3265	25.51	6147.1667	12.9409	258.7671	25.53	80.1703	0.2255	4.5109

25.72	4298.0667	3.1335	62.6326	25.71	5937.8000	12.2079	244.0599	25.73	79.2087	0.2200	4.4006
25.93	4223.7000	3.3406	66.7755	25.92	5743.1667	12.6218	252.4352	25.93	78.3297	0.2184	4.3688
26.13	4148.0667	3.0200	60.3815	26.12	5576.5000	11.4469	228.9893	26.14	77.2760	0.2148	4.2952
26.34	4064.1333	2.8140	56.3748	26.32	5396.8667	10.2795	205.5383	26.34	76.5020	0.2112	4.2226
26.54	3991.8333	3.1965	63.9656	26.53	5228.9333	10.9758	219.5187	26.54	75.5873	0.2074	4.1484
26.74	3924.8333	2.9991	59.9454	26.73	5074.8000	10.8055	216.1100	26.75	74.8270	0.2058	4.1158
26.95	3847.9000	2.5969	51.9936	26.93	4919.5667	9.4367	188.7348	26.95	73.9990	0.2028	4.0568
27.15	3776.2000	2.8748	57.5141	27.14	4757.6667	9.4852	189.7525	27.16	73.0707	0.1980	3.9599
27.36	3714.7333	2.9663	59.3456	27.34	4614.7333	9.6520	192.9882	27.36	72.3367	0.1971	3.9410
27.56	3652.0333	2.6538	53.0965	27.54	4476.3667	8.7225	174.4006	27.56	71.1487	0.1930	3.8596
27.76	3586.2333	2.6063	52.1072	27.75	4328.6667	8.4530	169.0648	27.77	70.4087	0.1891	3.7820
27.96	3527.8333	2.8252	56.5453	27.95	4195.7333	8.8571	177.1435	27.97	69.6760	0.1871	3.7412
28.17	3465.1333	2.4492	48.9842	28.16	4070.7333	8.2896	165.7888	28.17	69.0367	0.1853	3.7063
28.37	3400.1667	2.4060	48.1768	28.36	3940.1333	7.4966	149.9885	28.37	68.2877	0.1826	3.6528
28.57	3342.1667	2.6757	53.4794	28.56	3820.3333	7.4880	149.7171	28.58	67.4043	0.1788	3.5764
28.77	3286.2000	2.4239	48.5142	28.76	3715.5667	6.9673	139.2983	28.78	66.7853	0.1782	3.5632
28.98	3223.2667	2.1697	43.4143	28.97	3611.9667	6.6165	132.2790	28.98	65.8783	0.1747	3.4934
29.18	3165.9000	2.3372	46.6865	29.17	3500.9000	6.8387	136.7642	29.19	65.1847	0.1715	3.4301
29.39	3110.9333	2.3508	47.0730	29.37	3403.0667	6.6970	133.9406	29.39	64.3703	0.1706	3.4124
29.59	3052.7000	1.9920	39.8772	29.58	3304.6667	6.0417	120.8886	29.59	63.6960	0.1676	3.3506
29.79	2997.8333	2.1486	43.0443	29.78	3203.6333	6.0265	120.4822	29.80	63.0077	0.1649	3.2978
30.00	2955.1000	2.4693	49.4118	29.98	3111.3667	6.1163	122.3305	30.00	62.1597	0.1618	3.2357

**[EMIM][DEP]**	**[EMIM][FSI]**	**[EMIM][MeSO3]**

T/°C	Shear viscosity η/mPa·sec	Deviation between measure-ments ±	u_{95}	*T*/°C	Shear viscosity η/mPa·sec	Deviation between measure-ments ±	u_{95}	*T*/°C	Shear viscosity η/mPa·sec	Deviation between measure-ments ±	u_{95}
14.75	340.9333	12.4000	7.1591	14.76	22.5207	0.0980	0.0549	14.81	217.7367	6.4400	3.2854
15.05	339.1667	11.9000	6.8705	14.87	22.4437	0.1450	0.0727	14.93	215.8333	6.0800	3.1441
15.35	337.4000	11.7000	6.7550	15.03	22.3407	0.1170	0.0587	15.09	214.0700	5.3600	2.9136
15.65	335.2000	11.4000	6.5818	15.21	22.2823	0.0940	0.0512	15.27	212.1500	6.0400	3.1465
15.95	333.0667	10.7000	6.1776	15.41	22.1667	0.1420	0.0716	15.47	209.7433	5.6700	2.9635
16.25	330.1667	10.7000	6.1776	15.61	22.0293	0.1210	0.0609	15.67	207.0500	4.3600	2.3522
16.55	327.8000	10.5000	6.0622	15.82	21.9130	0.0860	0.0452	15.88	204.3933	4.8300	2.6418
16.85	326.2000	9.3000	5.3694	16.04	21.8023	0.1260	0.0648	16.09	201.7300	5.2600	2.7744
17.15	323.2333	9.7000	5.6003	16.25	21.6517	0.1290	0.0645	16.30	198.7567	3.8800	2.1742
17.45	320.4667	10.1000	5.8312	16.47	21.4703	0.0980	0.0518	16.52	195.9167	3.4900	1.8722
17.75	318.5000	9.9000	5.7158	16.69	21.3690	0.1240	0.0649	16.74	193.1233	4.3000	2.3400
18.05	315.4333	9.4000	5.4271	16.91	21.1773	0.1590	0.0805	16.96	190.3600	3.6600	2.0473
18.35	312.5667	9.5000	5.4848	17.13	21.0317	0.1240	0.0641	17.18	187.4833	3.0000	1.6621
18.65	310.7000	9.6000	5.5426	17.35	20.8890	0.1030	0.0546	17.40	184.7800	3.3700	1.7608
18.95	307.9333	8.8000	5.0807	17.57	20.7670	0.1300	0.0665	17.62	181.8267	3.5500	1.9839
19.25	305.4000	8.1000	4.6765	17.79	20.6030	0.1170	0.0588	17.84	179.0933	2.7700	1.5794
19.55	303.6000	9.3000	5.3694	18.01	20.4923	0.0860	0.0483	18.06	176.5133	2.8900	1.5529
19.85	301.0667	8.6000	4.9652	18.23	20.3623	0.1310	0.0667	18.27	173.9733	2.6800	1.4731
20.15	298.0667	8.6000	4.9652	18.45	20.2020	0.0860	0.0478	18.49	171.0733	2.5500	1.4665
20.45	296.1000	9.0000	5.1962	18.66	20.0637	0.0860	0.0473	18.70	168.6933	2.7000	1.4250
20.75	294.0333	8.5000	4.9075	18.88	19.9360	0.1340	0.0697	18.92	166.3500	2.8300	1.4589
21.05	291.1000	7.8000	4.5033	19.09	19.8027	0.1430	0.0716	19.13	164.0000	1.9700	1.1177
21.35	288.9000	8.1000	4.6765	19.30	19.5930	0.1100	0.0572	19.34	161.5800	2.1600	1.2301
21.65	287.6000	7.8000	4.5033	19.52	19.5063	0.1160	0.0633	19.55	159.4133	2.3800	1.2744

21.95	285.0333	7.6000	4.3879	19.73	19.3887	0.1540	0.0772	19.77	157.1633	1.7900	1.0166
22.25	282.6000	7.8000	4.5033	19.94	19.2580	0.1140	0.0579	19.98	155.0067	1.4600	0.7674
22.55	280.9000	7.8000	4.5033	20.15	19.1530	0.0920	0.0512	20.19	152.9800	1.6900	0.9166
22.85	278.5667	7.1000	4.0992	20.36	19.0437	0.1220	0.0641	20.40	150.9267	1.3800	0.7939
23.15	276.0667	7.1000	4.0992	20.57	18.9030	0.1230	0.0618	20.61	148.8900	1.0500	0.5285
23.45	274.3667	7.4000	4.2724	20.78	18.7600	0.0950	0.0529	20.82	147.0633	1.1300	0.5853
23.75	272.8000	6.3000	3.6373	20.99	18.6817	0.1360	0.0695	21.02	145.1800	1.8400	1.0025
24.05	270.0333	7.0000	4.0415	21.20	18.5103	0.1360	0.0683	21.23	143.3167	1.5300	0.7850
24.35	267.7000	7.5000	4.3301	21.41	18.4067	0.0980	0.0528	21.44	141.5000	1.2100	0.6426
24.65	265.8667	6.8000	3.9260	21.61	18.3160	0.1190	0.0633	21.64	139.8133	1.2700	0.7139
24.95	263.4333	6.4000	3.6950	21.82	18.1937	0.1510	0.0758	21.85	137.9400	1.6000	0.8224
25.25	261.4333	6.7000	3.8682	22.03	18.0800	0.0980	0.0520	22.06	136.2033	1.3000	0.6592
25.55	260.0000	6.6000	3.8105	22.23	17.9577	0.1070	0.0593	22.26	134.5633	1.1700	0.6536
25.85	257.8333	6.1000	3.5218	22.44	17.8673	0.1550	0.0782	22.47	132.9000	1.1400	0.5742
26.15	255.6667	6.2000	3.5796	22.65	17.7417	0.1260	0.0637	22.68	130.7967	1.2800	0.7094
26.45	255.0333	5.5000	3.1754	22.85	17.6630	0.0950	0.0529	22.88	129.0867	1.1000	0.5988
26.75	253.4333	5.8000	3.3486	23.06	17.5667	0.1400	0.0718	23.09	127.5033	1.0500	0.5519
27.05	251.3000	5.7000	3.2909	23.26	17.4353	0.1350	0.0677	23.29	125.9600	1.0400	0.5538
27.35	249.7000	6.0000	3.4641	23.47	17.3493	0.0840	0.0474	23.50	124.5867	1.1800	0.6035
27.65	248.1667	5.6000	3.2332	23.67	17.2540	0.1270	0.0666	23.70	123.2600	1.1400	0.6067
27.95	245.9667	5.3000	3.0600	23.88	17.1470	0.1460	0.0732	23.91	121.6933	0.9000	0.4545
28.25	244.3333	5.5000	3.1754	24.08	17.0017	0.1030	0.0533	24.11	120.2467	0.7300	0.3837
28.55	242.9000	5.4000	3.1177	24.29	16.9357	0.1120	0.0620	24.32	118.7300	1.1100	0.5742
28.85	241.2333	4.6000	2.6558	24.49	16.8280	0.1490	0.0749	24.52	117.2967	0.9900	0.4950
29.15	239.3667	5.0000	2.8868	24.69	16.7090	0.1140	0.0587	24.73	115.8767	0.7600	0.4065
30.00	238.0333	5.2000	3.0022	24.90	16.6457	0.0960	0.0540	24.93	114.5500	0.6400	0.3205
0.00	0.0000	0.0000	0.0000	25.10	16.5250	0.1530	0.0776	25.13	113.0533	1.0600	0.5315
0.00	0.0000	0.0000	0.0000	25.31	16.4277	0.1040	0.0540	25.34	111.6633	0.8300	0.4443
0.00	0.0000	0.0000	0.0000	25.51	16.3407	0.0860	0.0488	25.54	110.3867	0.6800	0.3449

0.00	0.0000	0.0000	0.0000	25.72	16.2770	0.1350	0.0695	25.74	109.1367	0.6400	0.3252
0.00	0.0000	0.0000	0.0000	25.92	16.1650	0.1100	0.0560	25.95	107.6800	0.8500	0.4557
0.00	0.0000	0.0000	0.0000	26.12	16.0543	0.1000	0.0539	26.15	106.4233	0.7700	0.4045
0.00	0.0000	0.0000	0.0000	26.32	16.0017	0.1170	0.0632	26.35	105.2400	0.6900	0.3460
0.00	0.0000	0.0000	0.0000	26.53	15.8797	0.1500	0.0752	26.56	104.0000	0.6700	0.3534
0.00	0.0000	0.0000	0.0000	26.73	15.7923	0.1070	0.0553	26.76	102.7967	0.8900	0.4829
0.00	0.0000	0.0000	0.0000	26.94	15.6973	0.1090	0.0613	26.96	101.7200	0.7100	0.3576
0.00	0.0000	0.0000	0.0000	27.14	15.6203	0.1570	0.0798	27.17	100.5667	0.6300	0.3581
0.00	0.0000	0.0000	0.0000	27.34	15.5160	0.1150	0.0583	27.37	99.4257	0.8150	0.4093
0.00	0.0000	0.0000	0.0000	27.55	15.4403	0.0950	0.0527	27.57	98.2087	0.5870	0.3064
0.00	0.0000	0.0000	0.0000	27.75	15.3833	0.1410	0.0723	27.78	97.0530	0.6830	0.3567
0.00	0.0000	0.0000	0.0000	27.95	15.2507	0.1360	0.0682	27.98	95.9333	0.8050	0.4091
0.00	0.0000	0.0000	0.0000	28.16	15.1847	0.0900	0.0498	28.18	94.8990	0.6070	0.3060
0.00	0.0000	0.0000	0.0000	28.36	15.1093	0.1280	0.0671	28.39	93.9780	0.7780	0.4147
0.00	0.0000	0.0000	0.0000	28.56	15.0070	0.1520	0.0762	28.59	93.0163	0.8150	0.4278
0.00	0.0000	0.0000	0.0000	28.77	14.9240	0.0880	0.0479	28.79	92.0107	0.7070	0.3589
0.00	0.0000	0.0000	0.0000	28.97	14.8430	0.1180	0.0632	29.00	91.0553	0.5040	0.2636
0.00	0.0000	0.0000	0.0000	29.17	14.7703	0.1520	0.0763	29.20	90.0410	0.7870	0.3978
0.00	0.0000	0.0000	0.0000	29.37	14.6570	0.1100	0.0572	29.40	88.9943	0.7830	0.3983
0.00	0.0000	0.0000	0.0000	29.58	14.6140	0.1030	0.0546	29.61	88.1047	0.5600	0.2933
0.00	0.0000	0.0000	0.0000	29.78	14.5247	0.1520	0.0771	29.81	87.1710	0.6350	0.3182
0.00	0.0000	0.0000	0.0000	29.98	14.4157	0.1290	0.0653	30.01	86.2390	0.9080	0.4583

[EMIM][OAc]				[EMIM][OOc]				[EMIM][OPr]			

T/°C	Shear viscosity η/mPa·sec	Deviation between measure-ments ±	u_{95}	*T*/°C	Shear viscosity η/mPa·sec	Deviation between measure-ments ±	u_{95}	*T*/°C	Shear viscosity η/mPa·sec	Deviation between measure-ments ±	u_{95}
14.75	213.8167	0.1573	3.1522	14.75	872.4433	43.4900	23.9401	14.75	224.6100	0.3350	6.6967
14.87	212.4767	0.1443	2.8801	14.87	861.3900	41.6700	22.7156	14.86	222.9633	0.3234	6.4747
15.03	210.6367	0.1459	2.9199	15.03	852.6233	41.4100	22.6080	15.02	221.2933	0.3063	6.1297
15.21	208.2633	0.1611	3.2259	15.21	844.4033	42.5700	22.9674	15.20	218.9233	0.3084	6.1735
15.40	205.7867	0.1491	2.9837	15.41	833.7967	42.8300	23.2917	15.40	216.3033	0.3140	6.2708
15.61	203.0333	0.1454	2.9074	15.61	821.3567	43.2400	23.4972	15.61	213.9533	0.3032	6.0660
15.82	200.2067	0.1593	3.1853	15.83	808.1467	41.7000	22.5726	15.82	211.1733	0.2962	5.9215
16.04	196.8000	0.1525	3.0478	16.04	797.0600	41.3000	22.6551	16.03	208.0400	0.3029	6.0558
16.25	194.0200	0.1427	2.8566	16.26	783.6200	44.3800	24.1896	16.25	205.2267	0.2919	5.8415
16.47	190.6467	0.1499	2.9975	16.48	769.7967	45.3500	24.6139	16.47	202.4267	0.2842	5.6789
16.70	187.7800	0.1495	2.9904	16.70	757.1067	43.6300	23.5737	16.69	199.2300	0.2941	5.8898
16.92	184.3133	0.1361	2.7212	16.92	746.9500	42.0100	22.7567	16.91	196.3733	0.2897	5.7921
17.14	181.3400	0.1417	2.8353	17.14	733.8133	42.7700	23.1853	17.14	193.4267	0.2730	5.4599
17.36	178.0800	0.1418	2.8343	17.36	721.5033	43.1500	23.3210	17.36	190.6100	0.2749	5.5020
17.58	175.1000	0.1353	2.7050	17.59	709.5433	39.8800	21.4643	17.58	187.8700	0.2741	5.4834
17.80	172.2933	0.1327	2.6551	17.81	697.3200	40.2700	21.7938	17.80	185.1433	0.2589	5.1770
18.02	169.3533	0.1377	2.7583	18.02	685.3833	40.7100	21.9754	18.02	182.2067	0.2569	5.1389
18.24	166.8267	0.1321	2.6400	18.24	675.1933	40.6500	22.0003	18.24	179.4933	0.2611	5.2206
18.45	164.2233	0.1275	2.5460	18.46	663.3333	37.2200	20.2144	18.45	176.9067	0.2507	5.0182
18.67	161.8333	0.1322	2.6439	18.68	651.1400	38.9300	21.0292	18.67	174.1967	0.2441	4.8794
18.88	158.8367	0.1289	2.5751	18.89	641.4133	39.4400	21.2472	18.88	171.5533	0.2489	4.9790
19.10	156.5567	0.1229	2.4533	19.10	631.3200	39.7100	21.5287	19.10	169.8433	0.2437	4.8751
19.31	153.9867	0.1234	2.4700	19.32	619.8067	38.5700	20.6972	19.31	167.4033	0.2317	4.6317
19.52	151.6433	0.1264	2.5259	19.53	610.3700	38.1200	20.8082	19.52	164.8667	0.2349	4.6982

19.74	149.1100	0.1174	2.3464	19.74	601.4233	38.2200	21.0142	19.74	162.6267	0.2342	4.6817
19.95	146.4067	0.1177	2.3559	19.96	591.1100	38.6000	21.3128	19.95	160.5800	0.2218	4.4357
20.16	144.3967	0.1204	2.4053	20.17	582.0200	37.9800	20.7302	20.16	158.2300	0.2232	4.4615
20.37	142.4067	0.1162	2.3245	20.38	573.9567	38.3300	20.8688	20.37	156.0733	0.2258	4.5208
20.58	140.5533	0.1130	2.2562	20.59	564.6933	38.6300	21.1406	20.58	154.1233	0.2156	4.3169
20.79	138.2367	0.1139	2.2807	20.80	555.5967	38.9100	21.2523	20.79	152.1967	0.2119	4.2397
21.00	136.3467	0.1131	2.2636	21.00	547.3433	36.9600	20.1084	21.00	150.1133	0.2160	4.3219
21.21	134.2200	0.1071	2.1391	21.21	538.8233	37.5600	20.5497	21.21	148.2300	0.2103	4.2043
21.41	132.2567	0.1096	2.1873	21.42	530.0700	38.0300	20.8195	21.41	146.2867	0.2030	4.0601
21.62	130.4267	0.1069	2.1407	21.63	522.7800	38.1400	20.8452	21.62	144.2300	0.2057	4.1165
21.83	128.2833	0.1043	2.0877	21.84	515.5000	35.8900	19.5337	21.83	142.4333	0.2036	4.0721
22.04	126.6833	0.1046	2.0924	22.04	507.9733	35.0700	19.1904	22.03	140.6233	0.1936	3.8729
22.24	124.5533	0.1038	2.0743	22.25	500.6767	35.3500	19.2882	22.24	138.7167	0.1956	3.9115
22.45	122.9800	0.1014	2.0290	22.46	494.0100	35.3900	19.3460	22.45	137.0467	0.2025	4.0517
22.66	121.1900	0.0990	1.9784	22.66	485.4667	32.0100	17.3375	22.65	135.3567	0.1938	3.8797
22.86	119.4900	0.1008	2.0148	22.87	478.2200	32.2000	17.4866	22.86	133.5500	0.1922	3.8396
23.07	117.9733	0.0988	1.9764	23.07	472.0000	32.3400	17.5849	23.07	131.8300	0.1948	3.8933
23.27	116.4267	0.0961	1.9236	23.28	465.0433	32.4700	17.7422	23.27	130.4000	0.1816	3.6339
23.48	114.9900	0.0975	1.9477	23.48	458.0900	32.4400	17.6377	23.48	128.7100	0.1766	3.5291
23.68	113.1200	0.0953	1.9034	23.69	452.2600	31.9300	17.4000	23.68	127.0300	0.1795	3.5894
23.89	111.7600	0.0932	1.8628	23.89	446.0167	32.0800	17.5511	23.89	125.5267	0.1762	3.5238
24.09	110.2400	0.0924	1.8484	24.10	439.1000	32.4500	17.7706	24.09	124.0433	0.1686	3.3698
24.30	108.7700	0.0928	1.8557	24.30	432.8800	30.7800	16.7315	24.29	122.4133	0.1705	3.4119
24.50	107.4167	0.0900	1.8020	24.51	426.7933	31.1300	17.0034	24.50	120.9633	0.1701	3.4048
24.70	105.6567	0.0886	1.7727	24.71	420.3200	31.4000	17.2100	24.70	119.5133	0.1621	3.2413
24.91	104.3900	0.0893	1.7822	24.92	414.3433	31.6800	17.3005	24.91	118.3467	0.1629	3.2531
25.11	102.7167	0.0860	1.7197	25.12	408.6433	29.9400	16.2412	25.11	116.9267	0.1637	3.2708
25.32	101.4820	0.0854	1.7093	25.32	402.7333	29.4600	16.2608	25.32	115.5633	0.1572	3.1454
25.52	100.1227	0.0853	1.7055	25.53	396.8200	29.7300	16.4039	25.52	114.1133	0.1542	3.0827

25.72	98.8120	0.0845	1.6909	25.73	391.7533	29.7000	16.3769	25.73	112.7867	0.1560	3.1223
25.93	97.6900	0.0829	1.6583	25.93	385.9767	28.2000	15.5338	25.93	111.4667	0.1522	3.0463
26.13	96.1713	0.0821	1.6415	26.14	380.4033	28.4900	15.5441	26.13	110.1067	0.1467	2.9332
26.34	95.0677	0.0818	1.6371	26.34	375.4633	28.3800	15.4626	26.33	108.7467	0.1486	2.9666
26.54	93.9183	0.0799	1.5979	26.55	370.6600	28.3200	15.4791	26.54	107.4300	0.1475	2.9478
26.74	92.8357	0.0794	1.5877	26.75	365.0333	27.7800	15.1738	26.74	106.0933	0.1407	2.8157
26.94	91.6497	0.0788	1.5766	26.95	360.1900	27.4400	14.9648	26.94	104.7900	0.1413	2.8254
27.15	90.4453	0.0776	1.5515	27.15	355.7500	27.5900	15.0648	27.15	103.5933	0.1415	2.8325
27.35	89.4543	0.0768	1.5351	27.36	350.7600	27.7300	15.1708	27.35	102.7100	0.1371	2.7439
27.56	87.7953	0.0753	1.5052	27.56	345.5000	26.6900	14.5484	27.55	101.6327	0.1346	2.6929
27.76	86.8280	0.0746	1.4918	27.77	341.7567	24.9600	13.5543	27.76	100.4467	0.1364	2.7247
27.96	85.8877	0.0732	1.4642	27.97	337.2300	25.0600	13.6525	27.96	99.3547	0.1325	2.6504
28.16	84.9350	0.0731	1.4629	28.17	332.4867	25.3200	13.8017	28.16	98.2523	0.1286	2.5728
28.37	83.9030	0.0728	1.4565	28.37	328.3500	25.2500	13.7295	28.37	97.1210	0.1305	2.6077
28.57	82.7913	0.0715	1.4293	28.58	324.3267	24.6200	13.4884	28.57	96.0743	0.1287	2.5740
28.77	81.9040	0.0712	1.4242	28.78	319.6467	25.1300	13.7180	28.77	94.9800	0.1237	2.4729
28.98	80.6657	0.0695	1.3891	28.99	315.5967	25.2400	13.7443	28.98	93.9207	0.1253	2.5055
29.18	79.7970	0.0695	1.3902	29.19	311.6767	24.7700	13.5046	29.18	92.8733	0.1244	2.4883
29.38	78.7207	0.0682	1.3635	29.39	307.2433	23.8300	13.1569	29.38	91.8487	0.1193	2.3857
29.59	77.7350	0.0675	1.3507	29.59	302.8767	25.0100	13.6353	29.59	90.7933	0.1182	2.3649
29.79	76.8820	0.0668	1.3364	29.80	299.3300	25.0300	13.6560	29.79	89.8237	0.1193	2.3860
29.99	75.7900	0.0656	1.3129	30.00	295.3933	24.9700	13.6401	29.99	88.8457	0.1147	2.2941

[EMIM][OTf]	[EMIM][SCN]	[EMIM][TFSI]

T/°C	Shear viscosity η/mPa·sec	Deviation between measure-ments ±	u_{95}	*T*/°C	Shear viscosity η/mPa·sec	Deviation between measure-ments ±	u_{95}	*T*/°C	Shear viscosity η/mPa·sec	Deviation between measure-ments ±	u_{95}
14.75	48.9593	0.0656	1.3138	14.75	26.4250	0.0224	0.4486	14.75	39.7393	0.0792	1.5844
14.88	48.7477	0.0644	1.2891	14.88	26.3060	0.0225	0.4507	14.88	39.6203	0.0803	1.6076
15.04	48.5240	0.0647	1.2941	15.04	26.2410	0.0191	0.3826	15.04	39.4797	0.0788	1.5763
15.23	48.3363	0.0653	1.3067	15.23	26.0973	0.0214	0.4292	15.23	39.3010	0.0785	1.5708
15.42	47.9937	0.0635	1.2696	15.42	25.9050	0.0222	0.4446	15.42	39.0537	0.0793	1.5867
15.62	47.6267	0.0615	1.2290	15.63	25.7597	0.0201	0.4018	15.63	38.8093	0.0782	1.5635
15.84	47.2627	0.0621	1.2423	15.84	25.5723	0.0196	0.3924	15.84	38.5243	0.0771	1.5415
16.05	46.9357	0.0619	1.2371	16.05	25.3607	0.0221	0.4424	16.06	38.2897	0.0782	1.5642
16.27	46.5300	0.0601	1.2034	16.27	25.1847	0.0204	0.4085	16.28	38.0103	0.0773	1.5473
16.49	46.1183	0.0595	1.1901	16.50	25.0057	0.0189	0.3768	16.50	37.7057	0.0761	1.5211
16.71	45.7783	0.0630	1.2592	16.72	24.7953	0.0192	0.3832	16.72	37.4093	0.0772	1.5427
16.93	45.4840	0.0588	1.1761	16.94	24.6337	0.0195	0.3891	16.94	37.1790	0.0752	1.5043
17.15	45.0713	0.0569	1.1379	17.16	24.4630	0.0173	0.3469	17.16	36.8907	0.0740	1.4812
17.38	44.6740	0.0580	1.1591	17.38	24.2320	0.0185	0.3710	17.38	36.5640	0.0733	1.4651
17.59	44.3163	0.0572	1.1437	17.60	24.0307	0.0187	0.3744	17.60	36.2710	0.0739	1.4781
17.82	43.9237	0.0546	1.0926	17.82	23.8820	0.0155	0.3110	17.82	35.9953	0.0724	1.4473
18.03	43.5430	0.0554	1.1066	18.04	23.6783	0.0158	0.3147	18.04	35.6877	0.0716	1.4319
18.25	43.1613	0.0549	1.0971	18.26	23.4353	0.0182	0.3647	18.25	35.3893	0.0724	1.4479
18.47	42.8413	0.0534	1.0680	18.47	23.3070	0.0171	0.3414	18.47	35.1540	0.0721	1.4423
18.68	42.4763	0.0529	1.0574	18.69	23.1423	0.0153	0.3059	18.69	34.8587	0.0700	1.3991
18.90	42.1150	0.0533	1.0659	18.91	22.9157	0.0162	0.3243	18.90	34.5603	0.0703	1.4067
19.11	41.7457	0.0516	1.0333	19.12	22.7343	0.0158	0.3166	19.12	34.2830	0.0699	1.3968
19.33	41.3043	0.0496	0.9921	19.33	22.5743	0.0164	0.3273	19.33	33.9497	0.0678	1.3557
19.54	40.9530	0.0512	1.0239	19.55	22.3853	0.0181	0.3626	19.54	33.6483	0.0679	1.3574

19.75	40.6080	0.0500	0.9996	19.76	22.1857	0.0186	0.3716	19.75	33.3870	0.0672	1.3442
19.96	40.2587	0.0484	0.9674	19.97	22.0693	0.0161	0.3212	19.97	33.1227	0.0663	1.3274
20.17	40.1190	0.0497	0.9943	20.18	21.9503	0.0143	0.2851	20.18	32.9953	0.0673	1.3449
20.38	39.7997	0.0489	0.9777	20.39	21.7537	0.0159	0.3172	20.39	32.7413	0.0670	1.3400
20.60	39.4767	0.0473	0.9469	20.60	21.6230	0.0145	0.2891	20.60	32.5033	0.0657	1.3141
20.80	39.1703	0.0476	0.9511	20.81	21.4860	0.0137	0.2739	20.81	32.2543	0.0645	1.2912
21.01	38.9097	0.0473	0.9480	21.02	21.3267	0.0165	0.3307	21.01	32.0307	0.0652	1.3040
21.22	38.5960	0.0461	0.9210	21.23	21.2007	0.0166	0.3319	21.22	31.8033	0.0648	1.2956
21.43	38.2983	0.0457	0.9136	21.44	21.0813	0.0148	0.2950	21.43	31.5767	0.0639	1.2784
21.64	38.0180	0.0464	0.9276	21.64	20.9033	0.0160	0.3199	21.64	31.3383	0.0639	1.2761
21.84	37.7343	0.0451	0.9013	21.85	20.7633	0.0170	0.3415	21.85	31.1187	0.0626	1.2521
22.05	37.4447	0.0442	0.8844	22.06	20.6540	0.0157	0.3137	22.05	30.8937	0.0623	1.2455
22.26	37.1710	0.0449	0.8985	22.26	20.4920	0.0165	0.3298	22.26	30.6687	0.0617	1.2355
22.47	36.8903	0.0442	0.8849	22.47	20.3317	0.0174	0.3483	22.47	30.4413	0.0621	1.2431
22.67	36.6053	0.0429	0.8583	22.68	20.2190	0.0149	0.2972	22.67	30.2283	0.0611	1.2215
22.88	36.3387	0.0436	0.8719	22.88	20.0663	0.0143	0.2862	22.88	30.0100	0.0602	1.2047
23.08	36.0720	0.0432	0.8631	23.09	19.9010	0.0156	0.3130	23.09	29.7847	0.0608	1.2163
23.29	35.7940	0.0419	0.8381	23.29	19.7990	0.0140	0.2793	23.29	29.5827	0.0602	1.2033
23.49	35.5910	0.0424	0.8477	23.50	19.7173	0.0143	0.2864	23.50	29.4183	0.0597	1.1937
23.70	35.3397	0.0425	0.8508	23.70	19.5557	0.0164	0.3288	23.70	29.2057	0.0596	1.1925
23.90	35.0750	0.0415	0.8315	23.91	19.4450	0.0156	0.3128	23.90	29.0023	0.0594	1.1879
24.11	34.8110	0.0410	0.8187	24.11	19.3467	0.0141	0.2819	24.11	28.8130	0.0574	1.1473
24.31	34.5400	0.0412	0.8248	24.32	19.1843	0.0160	0.3187	24.32	28.5773	0.0576	1.1508
24.52	34.2757	0.0406	0.8123	24.52	19.0437	0.0168	0.3365	24.52	28.3630	0.0581	1.1618
24.72	34.0170	0.0396	0.7910	24.73	18.9540	0.0147	0.2944	24.72	28.1737	0.0572	1.1430
24.93	33.7810	0.0401	0.8014	24.93	18.8333	0.0158	0.3159	24.93	27.9807	0.0562	1.1246
25.13	33.6360	0.0404	0.8090	25.13	18.6930	0.0155	0.3094	25.13	27.8517	0.0583	1.1659
25.33	33.4100	0.0384	0.7690	25.34	18.5823	0.0147	0.2931	25.34	27.6897	0.0566	1.1320
25.54	33.1740	0.0386	0.7724	25.54	18.4677	0.0143	0.2870	25.54	27.5093	0.0551	1.1020

25.74	32.9447	0.0390	0.7807	25.75	18.3267	0.0158	0.3158	25.74	27.3047	0.0556	1.1113
25.94	32.7297	0.0381	0.7617	25.95	18.2207	0.0146	0.2916	25.95	27.1393	0.0559	1.1180
26.15	32.5047	0.0376	0.7501	26.15	18.1180	0.0133	0.2667	26.15	26.9710	0.0546	1.0928
26.35	32.2850	0.0381	0.7607	26.36	17.9817	0.0145	0.2905	26.35	26.7833	0.0545	1.0906
26.56	32.0583	0.0372	0.7442	26.56	17.8733	0.0144	0.2873	26.56	26.5973	0.0545	1.0903
26.76	31.8583	0.0369	0.7367	26.77	17.8043	0.0134	0.2679	26.76	26.4590	0.0539	1.0786
26.96	31.6600	0.0369	0.7380	26.97	17.6747	0.0141	0.2814	26.97	26.2953	0.0529	1.0595
27.17	31.4450	0.0363	0.7268	27.17	17.5567	0.0140	0.2803	27.17	26.1033	0.0536	1.0731
27.37	31.2213	0.0352	0.7053	27.38	17.4697	0.0130	0.2610	27.37	25.9490	0.0523	1.0472
27.57	31.0113	0.0357	0.7139	27.58	17.3443	0.0136	0.2714	27.57	25.7813	0.0516	1.0316
27.77	30.7897	0.0356	0.7125	27.78	17.2213	0.0135	0.2708	27.78	25.5807	0.0523	1.0456
27.98	30.5733	0.0346	0.6924	27.99	17.1437	0.0120	0.2406	27.98	25.4193	0.0517	1.0349
28.18	30.3647	0.0346	0.6910	28.19	17.0233	0.0127	0.2541	28.18	25.2597	0.0510	1.0191
28.38	30.1713	0.0349	0.6976	28.39	16.9047	0.0137	0.2726	28.39	25.0907	0.0508	1.0170
28.59	29.9610	0.0340	0.6794	28.59	16.8520	0.0140	0.2792	28.59	24.9220	0.0508	1.0154
28.79	29.7567	0.0334	0.6682	28.80	16.7370	0.0145	0.2900	28.80	24.7760	0.0499	0.9983
28.99	29.5613	0.0340	0.6796	29.00	16.6143	0.0151	0.3021	29.00	24.6170	0.0499	0.9974
29.20	29.3707	0.0334	0.6694	29.20	16.5490	0.0135	0.2706	29.20	24.4507	0.0501	1.0026
29.40	29.2153	0.0328	0.6555	29.41	16.4653	0.0136	0.2730	29.40	24.3487	0.0486	0.9715
29.60	29.0290	0.0333	0.6647	29.61	16.3400	0.0145	0.2902	29.61	24.1947	0.0489	0.9768
29.81	28.8453	0.0324	0.6492	29.81	16.2613	0.0134	0.2682	29.81	24.0303	0.0488	0.9757
30.01	28.6533	0.0317	0.6335	30.02	16.1847	0.0133	0.2664	30.01	23.8893	0.0481	0.9627

**[HEXMIM][ACR]**				**[Pyrr][OAc]**				**[Pyrr][OFm]**			

T/°C	Shear viscosity η/mPa·sec	Deviation between measure-ments ±	u_{95}	*T*/°C	Shear viscosity η/mPa·sec	Deviation between measure-ments ±	u_{95}	*T*/°C	Shear viscosity η/mPa·sec	Deviation between measure-ments ±	u_{95}
14.75	1000.9067	1.4245	28.4903	14.75	44.1543	0.6460	0.3701	14.75	5.8207	0.3870	0.1982
14.86	990.0867	1.4141	28.2795	14.86	43.9647	0.6570	0.3652	14.88	5.7934	0.4171	0.2142
15.03	979.3200	1.3904	27.8145	15.02	43.6840	0.7310	0.3991	15.05	5.7819	0.3963	0.2050
15.21	966.4200	1.3492	26.9918	15.21	43.4373	0.6560	0.3720	15.23	5.7565	0.3925	0.2020
15.40	953.2167	1.3428	26.8631	15.40	43.1040	0.7030	0.4019	15.43	5.7127	0.4053	0.2082
15.61	940.1867	1.3292	26.5838	15.60	42.7213	0.7640	0.4256	15.63	5.6944	0.3815	0.1969
15.82	923.8100	1.2952	25.9061	15.81	42.3507	0.6890	0.3913	15.84	5.6648	0.3813	0.1966
16.04	908.7600	1.2901	25.7964	16.03	41.9250	0.6690	0.3709	16.06	5.6182	0.3892	0.1999
16.25	893.8400	1.2434	24.8646	16.25	41.5210	0.6860	0.3775	16.28	5.5808	0.3786	0.1953
16.47	876.7833	1.1858	23.7198	16.47	41.0330	0.6830	0.3915	16.50	5.5558	0.3639	0.1871
16.69	861.0367	1.2093	24.1816	16.69	40.6700	0.6930	0.3917	16.72	5.5106	0.3737	0.1919
16.92	846.9300	1.1847	23.6997	16.91	40.1313	0.7660	0.4377	16.94	5.4698	0.3716	0.1901
17.14	830.1967	1.1215	22.4374	17.13	39.7113	0.8030	0.4443	17.16	5.4349	0.3623	0.1859
17.36	814.5233	1.1409	22.8168	17.35	39.2647	0.7300	0.4147	17.39	5.4040	0.3691	0.1900
17.58	801.4600	1.1161	22.3177	17.57	38.8710	0.7510	0.4069	17.61	5.3586	0.3698	0.1903
17.80	785.9067	1.0625	21.2549	17.79	38.4460	0.7690	0.4159	17.83	5.3220	0.3631	0.1865
18.02	770.5733	1.0469	20.9399	18.01	38.0493	0.7020	0.3808	18.04	5.2943	0.3597	0.1846
18.23	757.9433	1.0376	20.7578	18.23	37.6597	0.6810	0.3746	18.26	5.2554	0.3462	0.1772
18.45	744.3600	1.0182	20.3635	18.44	37.2390	0.7730	0.4191	18.48	5.2163	0.3421	0.1754
18.67	729.1700	0.9841	19.6718	18.66	36.8693	0.6910	0.3887	18.69	5.1913	0.3330	0.1703
18.88	717.2800	1.0024	20.0415	18.88	36.4633	0.6730	0.3729	18.91	5.1506	0.3419	0.1750
19.10	706.0133	0.9630	19.2636	19.09	36.1127	0.7270	0.3873	19.12	5.1153	0.3643	0.1871
19.31	692.0733	0.9243	18.4872	19.30	35.6337	0.7680	0.4355	19.34	5.0903	0.3492	0.1798
19.53	679.9567	0.9207	18.4189	19.52	35.3057	0.7090	0.4059	19.55	5.0555	0.3483	0.1787

19.74	669.5867	0.9218	18.4435	19.73	34.9610	0.7160	0.3891	19.76	5.0225	0.3472	0.1773
19.95	656.7033	0.8625	17.2493	19.94	34.5877	0.6800	0.3487	19.97	5.0019	0.3266	0.1675
20.16	644.6733	0.8557	17.1162	20.15	34.2560	0.6810	0.3600	20.19	4.9750	0.3229	0.1656
20.37	634.9767	0.8570	17.1352	20.36	33.9463	0.7250	0.3833	20.40	4.9348	0.3304	0.1688
20.58	625.4333	0.8130	16.2531	20.57	33.5990	0.6950	0.3695	20.61	4.9087	0.3187	0.1639
20.79	614.8400	0.8083	16.1747	20.78	33.2070	0.6680	0.3716	20.81	4.8891	0.3216	0.1647
21.00	605.6400	0.8099	16.1987	20.99	32.9427	0.6330	0.3463	21.02	4.8538	0.3315	0.1695
21.20	596.1000	0.7912	15.8261	21.20	32.5763	0.7490	0.3952	21.23	4.8204	0.3209	0.1651
21.41	585.9667	0.7649	15.2946	21.41	32.2453	0.7250	0.3838	21.44	4.8055	0.3058	0.1559
21.62	576.9167	0.7613	15.2251	21.61	31.9720	0.6150	0.3428	21.65	4.7757	0.3063	0.1561
21.83	568.8833	0.7433	14.8718	21.82	31.6553	0.6720	0.3435	21.85	4.7411	0.3093	0.1581
22.03	558.5600	0.7223	14.4455	22.03	31.3447	0.6590	0.3441	22.06	4.7179	0.2966	0.1512
22.24	550.1033	0.7119	14.2494	22.23	30.9947	0.6080	0.3264	22.27	4.6944	0.3006	0.1532
22.45	542.6567	0.7074	14.1441	22.44	30.7483	0.6430	0.3343	22.48	4.6576	0.3106	0.1591
22.65	533.5433	0.6873	13.7440	22.65	30.4413	0.6660	0.3421	22.68	4.6364	0.3099	0.1583
22.86	524.4400	0.6691	13.3917	22.85	30.1747	0.5950	0.3059	22.89	4.6128	0.2979	0.1523
23.06	517.4667	0.6807	13.6058	23.06	29.9100	0.5980	0.3113	23.09	4.5771	0.3009	0.1538
23.27	509.0467	0.6416	12.8378	23.26	29.6310	0.6820	0.3494	23.30	4.5508	0.3038	0.1551
23.47	500.1633	0.6203	12.4021	23.47	29.3700	0.5700	0.2989	23.50	4.5329	0.2913	0.1492
23.68	492.7967	0.6219	12.4407	23.67	29.0937	0.5320	0.2747	23.71	4.5018	0.3027	0.1547
23.88	486.0333	0.6175	12.3474	23.88	28.8537	0.6610	0.3334	23.91	4.4718	0.2985	0.1522
24.09	477.4033	0.5840	11.6872	24.08	28.5270	0.6270	0.3358	24.12	4.4582	0.2810	0.1445
24.29	470.0767	0.5765	11.5280	24.29	28.2790	0.5420	0.2915	24.32	4.4335	0.2960	0.1513
24.50	463.5967	0.5688	11.3731	24.49	28.0313	0.5780	0.3079	24.53	4.3990	0.2963	0.1511
24.70	456.3700	0.5619	11.2341	24.69	27.7423	0.6580	0.3337	24.73	4.3863	0.2553	0.1327
24.91	449.0867	0.5570	11.1404	24.90	27.5110	0.5200	0.2714	24.94	4.3644	0.2952	0.1516
25.11	442.9833	0.5568	11.1299	25.10	27.2227	0.5940	0.3065	25.14	4.3298	0.2959	0.1513
25.31	436.2867	0.5386	10.7705	25.31	26.9913	0.6630	0.3350	25.34	4.3041	0.2870	0.1474
25.52	429.1767	0.5239	10.4841	25.51	26.7500	0.5620	0.2868	25.55	4.2910	0.2832	0.1444

25.72	423.3133	0.5247	10.4855	25.72	26.5610	0.4930	0.2474	25.75	4.2696	0.2809	0.1438
25.92	417.3367	0.5131	10.2571	25.92	26.3160	0.5920	0.2978	25.96	4.2392	0.2792	0.1438
26.13	410.4667	0.4963	9.9166	26.12	26.0417	0.5870	0.2947	26.16	4.2260	0.2673	0.1371
26.33	404.7233	0.4940	9.8766	26.33	25.8637	0.5030	0.2533	26.36	4.2008	0.2751	0.1405
26.54	400.4167	0.4888	9.7733	26.53	25.6123	0.5840	0.2958	26.56	4.1691	0.2651	0.1352
26.74	394.0633	0.4711	9.4214	26.73	25.3937	0.5620	0.2845	26.77	4.1501	0.2514	0.1282
26.94	388.0833	0.4648	9.2932	26.94	25.1383	0.5360	0.2883	26.97	4.1317	0.2531	0.1288
27.15	383.1833	0.4609	9.2151	27.14	24.9237	0.5410	0.2816	27.18	4.0975	0.2564	0.1307
27.35	377.6867	0.4549	9.0927	27.34	24.7143	0.5790	0.2993	27.38	4.0803	0.2693	0.1380
27.55	371.7167	0.4464	8.9231	27.55	24.4773	0.5440	0.2733	27.58	4.0709	0.2635	0.1367
27.76	366.8567	0.4471	8.9366	27.75	24.3143	0.5010	0.2508	27.79	4.0333	0.2749	0.1412
27.96	361.7900	0.4339	8.6704	27.96	24.0683	0.5720	0.2905	27.99	4.0112	0.2681	0.1373
28.16	355.3300	0.4099	8.1940	28.16	23.8790	0.5220	0.2665	28.19	4.0252	0.1968	0.1056
28.37	350.3100	0.4169	8.3329	28.36	23.6740	0.4850	0.2561	28.39	3.9840	0.2520	0.1281
28.57	345.6933	0.4111	8.2217	28.56	23.4610	0.5470	0.2800	28.60	3.9577	0.2535	0.1293
28.77	340.3733	0.3965	7.9332	28.77	23.2677	0.5020	0.2581	28.80	3.9454	0.2425	0.1242
28.98	336.8600	0.3936	7.8728	28.97	23.0577	0.4770	0.2449	29.00	3.9250	0.2402	0.1227
29.18	332.7333	0.3922	7.8536	29.17	22.8817	0.5210	0.2641	29.21	3.8958	0.2446	0.1245
29.38	327.9300	0.3823	7.6500	29.37	22.6403	0.5440	0.2777	29.41	3.8802	0.2332	0.1192
29.59	323.0833	0.3755	7.5072	29.58	22.4753	0.4620	0.2351	29.61	3.8654	0.2275	0.1168
29.79	319.4200	0.3719	7.4302	29.78	22.2980	0.4540	0.2345	29.82	3.8391	0.2307	0.1174
29.99	314.9633	0.3671	7.3450	29.98	22.0613	0.5490	0.2771	30.02	3.8149	0.2210	0.1133

[TEAH][MeSO3]

T/°C	Shear viscosity η/mPa·sec	Deviation between measure-ments ±	u_{95}
14.75	155.0133	0.2673	5.3467
14.86	154.0567	0.2672	5.3444
15.03	153.1233	0.2693	5.3902
15.21	151.9533	0.2673	5.3471
15.40	150.4367	0.2627	5.2548
15.61	148.8267	0.2584	5.1694
15.82	147.2200	0.2568	5.1366
16.04	145.5000	0.2503	5.0054
16.25	143.6867	0.2451	4.9050
16.47	141.8367	0.2426	4.8519
16.69	140.1100	0.2415	4.8266
16.92	138.4133	0.2344	4.6875
17.14	136.5800	0.2310	4.6244
17.36	134.7067	0.2276	4.5536
17.58	132.9867	0.2242	4.4836
17.80	131.2467	0.2198	4.3949
18.02	129.4667	0.2188	4.3771
18.24	127.7467	0.2124	4.2466
18.45	126.1867	0.2099	4.1995
18.67	124.5400	0.2104	4.2086
18.89	122.8833	0.2056	4.1104
19.10	121.3900	0.1990	3.9804
19.31	120.1000	0.2060	4.1210
19.53	118.5933	0.2021	4.0378
19.74	117.1333	0.1984	3.9691

19.95	115.7200	0.1946	3.8903
20.16	114.3933	0.1965	3.9333
20.37	113.0033	0.1903	3.8092
20.58	111.6567	0.1890	3.7768
20.79	110.3167	0.1884	3.7663
21.00	108.8967	0.1750	3.5006
21.21	107.6733	0.1679	3.3550
21.42	106.4400	0.1689	3.3753
21.62	105.2733	0.1645	3.2883
21.83	104.3300	0.1694	3.3885
22.04	103.1567	0.1681	3.3658
22.24	101.9647	0.1667	3.3361
22.45	100.8193	0.1622	3.2441
22.66	99.7207	0.1638	3.2778
22.86	98.5427	0.1670	3.3389
23.07	97.4310	0.1645	3.2909
23.27	96.3363	0.1598	3.1960
23.48	95.3017	0.1571	3.1413
23.68	94.2923	0.1508	3.0165
23.89	93.2490	0.1464	2.9289
24.09	92.2273	0.1460	2.9204
24.30	91.1927	0.1442	2.8827
24.50	90.1947	0.1396	2.7928
24.71	89.2063	0.1384	2.7679
24.91	88.2410	0.1379	2.7574
25.12	87.3877	0.1383	2.7671
25.32	86.4557	0.1350	2.7003
25.52	85.5307	0.1366	2.7333
25.72	84.6097	0.1338	2.6768

25.93	83.6683	0.1293	2.5868
26.13	82.7633	0.1285	2.5704
26.33	81.8753	0.1275	2.5493
26.54	81.0270	0.1250	2.4998
26.74	80.3543	0.1265	2.5304
26.95	79.5627	0.1268	2.5361
27.15	78.7277	0.1231	2.4624
27.35	77.9223	0.1216	2.4325
27.56	77.1473	0.1221	2.4415
27.76	76.3260	0.1218	2.4364
27.96	75.5533	0.1188	2.3756
28.17	74.7840	0.1195	2.3917
28.37	74.0673	0.1190	2.3798
28.57	73.4443	0.1131	2.2614
28.77	72.7047	0.1134	2.2686
28.98	71.9663	0.1128	2.2567
29.18	71.2753	0.1101	2.2005
29.38	70.5310	0.1079	2.1585
29.59	69.8363	0.1091	2.1826
29.79	69.1503	0.1068	2.1350
30.00	68.4820	0.1035	2.0694

T…Temperature

Table S9. Experimental rheology data at constant temperature measurements (T = 25°C) and the deviations of the three independent measurements (±). Measurements were conducted in an ambient atmosphere at 101.3 kPa. u_{95} – standard deviation with a confidence interval of 0.95.

[BMIM][ACR]				[BMIM][OAC]				[BMPyrr][FSI]			
Shear rate $\dot{\gamma}$ /s^{-1}	Shear viscosity η/mPa·sec	Deviation between measure-ments ±	u_{95}	Shear rate $\dot{\gamma}$ /s^{-1}	Shear viscosity η/mPa·sec	Deviation between measure-ments ±	u_{95}	Shear rate $\dot{\gamma}$ /s^{-1}	Shear viscosity η/mPa·sec	Deviation between measure-ments ±	u_{95}
1	286.6033	0.0128	12.8191	1	313.0467	0.0056	5.6176	1	50.303	0.0013	1.2989
3.01	285.9900	0.0377	12.5283	3.01	310.7167	0.0164	5.4444	3.01	49.4857	0.0016	0.5262
5.02	285.8067	0.0586	11.6702	5.02	311.2567	0.0267	5.3058	5.02	49.494	0.0014	0.2842
7.03	285.5200	0.0787	11.2009	7.03	311.0400	0.0374	5.3174	7.03	49.4083	0.0008	0.1123
9.04	285.3400	0.1007	11.1413	9.04	310.9167	0.0495	5.4791	9.04	49.3633	0.0003	0.0325
11.1	285.6433	0.1293	11.6971	11.1	310.9867	0.0607	5.4964	11.1	49.397	0.0015	0.1355
13.1	285.6433	0.1498	11.4719	13.1	310.7567	0.0701	5.3751	13.1	49.4007	0.0018	0.1374
15.1	284.9800	0.1563	10.3673	15.1	310.7067	0.0835	5.5483	15.1	49.3697	0.0005	0.0300
17.1	285.2733	0.1938	11.3450	17.1	310.8233	0.0931	5.4502	17.1	49.387	0.0021	0.1215
19.1	284.6733	0.2028	10.6230	19.1	310.4367	0.1052	5.5137	19.1	49.389	0.0009	0.0457
21.1	284.9900	0.2367	11.2166	21.1	310.6200	0.1162	5.5079	21.1	49.392	0.0021	0.0988
23.1	284.4700	0.2430	10.5166	23.1	310.3767	0.1304	5.6349	23.1	49.379	0.001	0.0444
25.1	284.6433	0.2780	11.0693	25.1	310.4833	0.1408	5.6061	25.1	49.3953	0.0025	0.0979
27.1	284.5333	0.2938	10.8245	27.1	310.5767	0.1599	5.8947	27.1	49.3867	0.0017	0.0631
29.1	284.1600	0.3085	10.5865	29.1	310.1233	0.1684	5.7793	29.1	49.3967	0.0012	0.0424
31.2	284.1867	0.3378	10.8409	31.2	310.0567	0.1755	5.6349	31.2	49.3907	0.0029	0.0941
33.2	284.2233	0.3632	10.9507	33.2	310.0933	0.1894	5.7100	33.2	49.3927	0.0027	0.0821
35.2	284.0700	0.3808	10.8360	35.2	309.9767	0.2073	5.8947	35.2	49.3953	0.002	0.0562
37.2	283.8167	0.3981	10.7024	37.2	309.6933	0.2205	5.9352	37.2	49.3933	0.002	0.0526
39.2	283.6633	0.4150	10.5931	39.2	309.4667	0.2315	5.9121	39.2	49.3907	0.0028	0.0716
41.2	283.5967	0.4336	10.5176	41.2	309.2400	0.2419	5.8717	41.2	49.3847	0.0028	0.0688
43.2	283.4433	0.4544	10.5222	43.2	309.0567	0.2529	5.8428	43.2	49.384	0.0033	0.0757
45.2	283.2900	0.4758	10.5245	45.2	309.0300	0.2685	5.9409	45.2	49.3817	0.0033	0.0742

47.2	283.1733	0.4974	10.5314	47.2	308.7633	0.2766	5.8659	47.2	49.3803	0.0035	0.0745
49.2	283.0500	0.5208	10.5749	49.2	308.6333	0.2904	5.9005	49.2	49.3757	0.0036	0.0731
51.3	282.9800	0.5446	10.6197	51.3	308.5733	0.3048	5.9525	51.3	49.3737	0.0037	0.0723
53.3	282.9400	0.5731	10.7551	53.3	308.4900	0.3152	5.9236	53.3	49.375	0.0037	0.0701
55.3	282.8233	0.6006	10.8617	55.3	308.0133	0.3060	5.5368	55.3	49.373	0.004	0.0713
57.3	282.5567	0.6206	10.8308	57.3	307.7033	0.3083	5.3809	57.3	49.3703	0.0044	0.0765
59.3	282.1367	0.6395	10.7906	59.3	307.3767	0.3187	5.3751	59.3	49.3663	0.0047	0.0798
61.3	281.9533	0.6639	10.8301	61.3	307.5067	0.3562	5.8081	61.3	49.3617	0.0048	0.0791
63.3	282.0000	0.6954	10.9794	63.3	307.5367	0.3666	5.7908	63.3	49.3573	0.0047	0.0746
65.3	281.7733	0.7216	11.0488	65.3	307.2800	0.3666	5.6118	65.3	49.351	0.0047	0.0715
67.3	281.4333	0.7351	10.9135	67.3	306.8900	0.3793	5.6292	67.3	49.3613	0.006	0.0898
69.3	281.4667	0.7646	11.0233	69.3	306.9100	0.4007	5.7850	69.3	49.3493	0.0051	0.0734
71.4	281.3333	0.7934	11.1188	71.4	306.7333	0.3995	5.6061	71.4	49.349	0.0064	0.0892
73.4	281.0000	0.8068	10.9949	73.4	306.3467	0.4122	5.6176	73.4	49.3387	0.006	0.0819
75.4	281.0567	0.8399	11.1463	75.4	306.3667	0.4295	5.7042	75.4	49.3353	0.0059	0.0785
77.4	280.7667	0.8610	11.1294	77.4	306.0600	0.4278	5.5252	77.4	49.3283	0.0064	0.0820
79.4	280.6500	0.8848	11.1386	79.4	305.8700	0.4538	5.7158	79.4	49.329	0.0067	0.0840
81.4	280.5600	0.9150	11.2327	81.4	305.7867	0.4521	5.5483	81.4	49.3123	0.0053	0.0649
83.4	280.2933	0.9269	11.1120	83.4	305.4500	0.4711	5.6465	83.4	49.3147	0.0058	0.0692
85.4	280.2900	0.9629	11.2714	85.4	305.4500	0.4769	5.5772	85.4	49.305	0.0057	0.0664
87.4	279.9733	0.9749	11.1520	87.4	305.0400	0.4879	5.5772	87.4	49.3057	0.0061	0.0702
89.4	280.0100	1.0099	11.2886	89.4	305.0567	0.4965	5.5483	89.4	49.3037	0.0057	0.0634
91.5	279.7000	1.0219	11.1760	91.5	304.6833	0.5081	5.5541	91.5	49.2953	0.0068	0.0748
93.5	279.7333	1.0576	11.3152	93.5	304.7000	0.5185	5.5426	93.5	49.2893	0.0071	0.0758
95.5	279.4400	1.0713	11.2207	95.5	304.3500	0.5306	5.5599	95.5	49.287	0.0082	0.0856
97.5	279.4567	1.1060	11.3456	97.5	304.3733	0.5364	5.5021	97.5	49.277	0.0074	0.0757
99.5	279.2233	1.1200	11.2609	99.5	304.0567	0.5537	5.5657	99.5	49.276	0.0084	0.0841
102	279.1500	1.1534	11.3618	102	303.9900	0.5537	5.4560	102	49.2643	0.0075	0.0734
104	278.9833	1.1700	11.3045	104	303.7667	0.5774	5.5830	104	49.2643	0.0083	0.0809

106	278.8467	1.1950	11.3270	106	303.6300	0.5722	5.4213	106	49.257	0.0083	0.0793
108	278.8000	1.2195	11.3402	108	303.5067	0.5970	5.5483	108	49.2543	0.0087	0.0813
110	278.5400	1.2420	11.3393	110	303.2100	0.5901	5.3867	110	49.2507	0.0087	0.0791
112	278.5700	1.2693	11.3752	112	303.2467	0.6143	5.5137	112	49.246	0.0093	0.0832
114	278.2833	1.2849	11.3173	114	302.9100	0.6137	5.4040	114	49.2427	0.0101	0.0888
116	278.3333	1.3239	11.4507	116	302.9300	0.6287	5.4386	116	49.2353	0.009	0.0779
118	278.0467	1.3332	11.3338	118	302.5867	0.6403	5.4444	118	49.2323	0.01	0.0856
120	278.0767	1.3669	11.4302	120	302.6233	0.6437	5.3809	120	49.228	0.0101	0.0845
122	277.8633	1.3821	11.3680	122	302.3367	0.6640	5.4617	122	49.225	0.0105	0.0868
124	277.8000	1.4181	11.4758	124	302.2867	0.6605	5.3405	124	49.216	0.0101	0.0811
126	277.6467	1.4330	11.4093	126	302.0800	0.6899	5.4906	126	49.215	0.0111	0.0885
128	277.5500	1.4606	11.4443	128	301.9800	0.6790	5.3174	128	49.2073	0.0109	0.0854
130	277.4567	1.4771	11.3946	130	301.8100	0.7096	5.4733	130	49.206	0.012	0.0927
132	277.5667	1.4874	11.3002	132	301.6967	0.6957	5.2885	132	49.201	0.0125	0.0948
134	277.4633	1.5055	11.2639	134	301.5367	0.7327	5.4791	134	49.1993	0.0128	0.0955
136	277.3567	1.5404	11.3495	136	301.4200	0.7171	5.2828	136	49.1917	0.0121	0.0896
138	277.2267	1.5513	11.2680	138	301.2500	0.7534	5.4733	138	49.192	0.0133	0.0959
140	277.2367	1.5870	11.3589	140	301.3200	0.7454	5.3347	140	49.1847	0.0128	0.0910
142	276.9667	1.5934	11.2433	142	300.9233	0.7627	5.3809	142	49.1857	0.0142	0.1007
144	277.0867	1.6332	11.3623	144	301.0633	0.7731	5.3809	144	49.1757	0.0134	0.0930
146	276.7400	1.6426	11.2741	146	300.6500	0.7592	5.2135	146	49.171	0.0139	0.0948
148	276.8067	1.6709	11.3069	148	300.7400	0.8089	5.4733	148	49.17	0.0149	0.1005
150	276.7333	1.7053	11.3868	150	300.7400	0.7991	5.3347	150	49.1597	0.0138	0.0920
152	276.4500	1.7058	11.2357	152	300.3067	0.8025	5.2885	152	49.1593	0.0148	0.0981
154	276.5500	1.7434	11.3407	154	300.3833	0.8325	5.4155	154	49.1563	0.0154	0.0999
156	276.4767	1.7734	11.3827	156	300.3967	0.8216	5.2712	156	49.147	0.0146	0.0935
158	276.1733	1.7773	11.2663	158	300.0200	0.8273	5.2481	158	49.1443	0.0155	0.0982
160	276.2033	1.7963	11.2438	160	300.0167	0.8787	5.4964	160	49.1457	0.0173	0.1080
162	276.3100	1.8438	11.3988	162	300.1833	0.8926	5.5195	162	49.1407	0.0156	0.0967

164	276.1667	1.8730	11.4374	164	300.0133	0.8672	5.2943	164	49.1313	0.0155	0.0946
166	275.8667	1.8823	11.3486	166	299.7233	0.8631	5.2077	166	49.125	0.0161	0.0972
168	275.7100	1.8899	11.2613	168	299.4600	0.8805	5.2481	168	49.127	0.0168	0.1002
170	275.6833	1.9106	11.2484	170	299.4167	0.9186	5.4098	170	49.126	0.0179	0.1054
172	275.7133	1.9347	11.2562	172	299.4167	0.9411	5.4791	172	49.121	0.019	0.1105
174	275.7333	1.9673	11.3139	174	299.4100	0.9601	5.5252	174	49.119	0.0185	0.1065
176	275.7233	1.9894	11.3152	176	299.3767	0.9757	5.5483	176	49.113	0.0185	0.1051
178	275.6167	2.0160	11.3367	178	299.3133	0.9884	5.5541	178	49.1103	0.0196	0.1103
180	275.5267	2.0302	11.2883	180	299.2167	1.0040	5.5830	180	49.1063	0.0201	0.1118
182	275.3667	2.0486	11.2682	182	299.0067	1.0029	5.5137	182	49.103	0.0206	0.1136
184	275.1733	2.0600	11.1990	184	298.8133	0.9861	5.3636	184	49.0973	0.021	0.1140
186	275.1200	2.0883	11.2351	186	298.7933	0.9642	5.1904	186	49.0833	0.0201	0.1083
188	275.2867	2.1409	11.3869	188	299.0233	0.9659	5.1384	188	49.074	0.0185	0.0986
190	275.5100	2.1838	11.4964	190	299.2133	1.0317	5.4329	190	49.0777	0.019	0.0999
192	275.2400	2.1632	11.2661	192	298.8233	1.0733	5.5888	192	49.0847	0.0229	0.1196
194	274.8800	2.1734	11.2013	194	298.4867	1.0092	5.2019	194	49.0673	0.0211	0.1089
196	275.2800	2.2585	11.5223	196	299.0233	1.0340	5.2770	196	49.054	0.0183	0.0936
198	275.0367	2.2297	11.2593	198	298.5833	1.1039	5.5714	198	49.0687	0.0225	0.1138
200	274.8800	2.2841	11.4225	200	298.5567	1.0156	5.0807	200	49.0513	0.0171	0.0856
**[Chol][Lys]**				**[DBUH][OAC]**				**[EMIM][ACR]**			
Shear rate $\dot{\gamma}$ /s^{-1}	Shear viscosity η/mPa·sec	Deviation between measure-ments ±	u_{95}	Shear rate $\dot{\gamma}$ /s^{-1}	Shear viscosity η/mPa·sec	Deviation between measure-ments ±	u_{95}	Shear rate $\dot{\gamma}$ /s^{-1}	Shear viscosity η/mPa·sec	Deviation between measure-ments ±	u_{95}
1	4864.2333	0.0825	82.6246	1	6784.0333	0.4225	422.6244	1	88.1423	0.0055	5.5397
3.01	4863.1333	0.2362	78.4458	3.01	6786.8667	1.2552	417.3877	3.01	88.1350	0.0160	5.3287
5.02	4864.8667	0.4083	81.3686	5.02	6789.7000	2.1159	421.4103	5.02	87.9733	0.0268	5.3395
7.03	4872.8667	0.6077	86.3630	7.03	6781.4000	3.0495	433.8009	7.03	87.6643	0.0372	5.2975
9.04	4867.8667	0.7641	84.5131	9.04	6766.9667	3.9942	441.8671	9.04	87.5860	0.0472	5.2206

11.1	4854.9333	0.8848	80.1178	11.1	6767.2667	4.7272	427.7896	11.1	87.6210	0.0574	5.1992
13.1	4854.8333	1.0439	79.9247	13.1	6776.0667	5.5683	426.3720	13.1	87.7163	0.0689	5.2720
15.1	4851.3667	1.2702	84.2791	15.1	6764.1333	6.5401	433.7429	15.1	87.5063	0.0791	5.2479
17.1	4843.7333	1.2769	74.7635	17.1	6775.2667	7.0964	415.3572	17.1	87.5120	0.0893	5.2282
19.1	4843.4667	1.6743	87.7055	19.1	6769.2333	8.1823	428.4174	19.1	87.4400	0.1000	5.2402
21.1	4834.6000	1.5859	75.1840	21.1	6778.7333	8.6048	407.8447	21.1	87.4310	0.1105	5.2366
23.1	4829.7000	1.8961	82.1005	23.1	6762.4667	9.6425	417.0798	23.1	87.4103	0.1206	5.2200
25.1	4821.0000	1.8631	74.2608	25.1	6769.0333	10.2031	406.0733	25.1	87.4083	0.1316	5.2377
27.1	4807.1333	1.9935	73.4646	27.1	6751.3333	10.7500	396.1528	27.1	87.2890	0.1412	5.2033
29.1	4806.0000	2.3811	81.6897	29.1	6740.1000	11.7924	404.7072	29.1	87.2923	0.1518	5.2113
31.2	4804.2333	2.4582	79.0449	31.2	6744.5000	12.4200	398.6674	31.2	87.2713	0.1626	5.2193
33.2	4792.1667	2.3353	70.4475	33.2	6738.2000	12.7488	384.4577	33.2	87.1630	0.1719	5.1851
35.2	4782.1000	2.6140	74.2610	35.2	6723.9000	13.3824	380.5699	35.2	87.0900	0.1823	5.1824
37.2	4777.6333	3.0000	80.7451	37.2	6711.8000	14.2458	383.1734	37.2	87.0610	0.1931	5.1921
39.2	4776.1333	3.2635	83.2674	39.2	6705.1667	15.1018	385.3409	39.2	87.0330	0.2035	5.1921
41.2	4774.9667	3.5131	85.2204	41.2	6701.2333	15.9240	386.4089	41.2	87.0037	0.2138	5.1885
43.2	4773.7333	3.6731	85.0295	43.2	6698.3333	16.7317	387.1733	43.2	86.9643	0.2239	5.1815
45.2	4770.5667	3.8255	84.6367	45.2	6694.7000	17.4504	385.8143	45.2	86.9143	0.2341	5.1783
47.2	4767.5000	4.0184	84.9809	47.2	6690.6667	18.1087	383.4202	47.2	86.8573	0.2440	5.1666
49.2	4763.2667	4.1266	83.8938	49.2	6686.8000	18.7041	379.8558	49.2	86.7837	0.2540	5.1576
51.3	4759.5000	4.1968	81.9120	51.3	6683.5333	19.1539	373.8105	51.3	86.7227	0.2641	5.1522
53.3	4756.2000	4.1612	78.1156	53.3	6684.2000	19.5838	367.6514	53.3	86.6380	0.2737	5.1389
55.3	4755.6000	4.1642	75.3729	55.3	6687.6667	20.1611	364.7666	55.3	86.5843	0.2829	5.1193
57.3	4760.2000	4.2167	73.6279	57.3	6689.1000	21.1340	368.9285	57.3	86.5640	0.2929	5.1130
59.3	4759.4333	4.5848	77.3391	59.3	6682.7333	22.2813	375.8105	59.3	86.5470	0.3032	5.1149
61.3	4754.3333	4.8233	78.5996	61.3	6674.3333	22.8783	373.2397	61.3	86.4780	0.3140	5.1229
63.3	4749.4667	4.6237	73.0746	63.3	6675.0333	22.9716	362.8643	63.3	86.3777	0.3231	5.1036
65.3	4750.8667	4.6638	71.3934	65.3	6679.6000	23.7930	364.2558	65.3	86.3447	0.3323	5.0875
67.3	4749.7667	5.1286	76.1069	67.3	6670.9667	25.0453	371.9830	67.3	86.3363	0.3434	5.0992

69.3	4742.0667	5.0453	72.7211	69.3	6665.7667	25.1351	362.4632	69.3	86.2313	0.3532	5.0936
71.4	4743.5333	5.0304	70.5365	71.4	6671.1667	25.7650	361.0850	71.4	86.1857	0.3618	5.0706
73.4	4741.4667	5.4947	74.8695	73.4	6661.8333	26.9892	367.9512	73.4	86.1733	0.3730	5.0848
75.4	4735.3000	5.3195	70.5685	75.4	6659.6667	26.9190	357.1779	75.4	86.0667	0.3824	5.0727
77.4	4737.3667	5.5501	71.7782	77.4	6659.8667	28.0492	362.4678	77.4	86.0527	0.3911	5.0546
79.4	4731.3667	5.8431	73.6033	79.4	6650.6000	28.5307	359.3245	79.4	85.9850	0.4022	5.0659
81.4	4730.2000	5.5982	68.7919	81.4	6654.2667	29.0149	356.4363	81.4	85.9243	0.4098	5.0336
83.4	4727.2000	6.1394	73.5959	83.4	6645.0333	30.1025	360.9061	83.4	85.9067	0.4212	5.0497
85.4	4722.0000	6.1866	72.4429	85.4	6647.6333	30.1923	353.4274	85.4	85.8183	0.4290	5.0222
87.4	4718.1333	6.6455	75.9542	87.4	6638.8333	31.4296	359.4881	87.4	85.8180	0.4403	5.0358
89.4	4715.7000	6.2096	69.4328	89.4	6640.5667	31.3767	350.8084	89.4	85.7233	0.4483	5.0120
91.5	4714.1667	6.9472	75.9759	91.5	6633.3333	32.6794	357.3466	91.5	85.7197	0.4589	5.0175
93.5	4709.6000	6.6986	71.6471	93.5	6634.4333	32.6517	349.3835	93.5	85.6303	0.4668	4.9953
95.5	4708.7000	7.2307	75.7192	95.5	6626.4333	33.8725	354.7523	95.5	85.6270	0.4781	5.0081
97.5	4706.3000	6.9270	71.0724	97.5	6628.6333	33.9456	348.2064	97.5	85.5447	0.4853	4.9782
99.5	4703.9000	7.4751	75.1114	99.5	6620.3333	35.0241	352.0030	99.5	85.5243	0.4969	4.9951
102	4702.0000	7.2061	71.0211	102	6623.3000	35.3080	347.8228	102	85.4623	0.5044	4.9692
104	4698.2000	7.6355	73.7342	104	6614.2000	36.0884	348.6100	104	85.4260	0.5161	4.9862
106	4697.9667	7.5474	71.4658	106	6616.4000	36.7985	348.6852	106	85.3903	0.5232	4.9578
108	4693.6333	7.7867	72.4593	108	6608.7000	37.0276	344.2586	108	85.3220	0.5344	4.9698
110	4695.3667	7.8878	72.0115	110	6608.2333	38.1457	348.2569	110	85.3127	0.5428	4.9546
112	4689.4000	7.9712	71.4330	112	6603.9667	38.0971	341.5256	112	85.2327	0.5526	4.9541
114	4689.8333	8.3777	73.7638	114	6600.3667	39.4736	347.5778	114	85.2303	0.5624	4.9535
116	4686.3333	8.0777	69.9037	116	6599.3000	39.2511	339.6322	116	85.1407	0.5708	4.9392
118	4685.8000	8.7657	74.5378	118	6593.0000	40.6308	345.5148	118	85.1497	0.5820	4.9507
120	4683.1333	8.4145	70.4004	120	6594.8000	40.6004	339.4731	120	85.0670	0.5891	4.9262
122	4680.8333	9.0042	74.0247	122	6585.9000	41.5723	341.8681	122	85.0513	0.6015	4.9478
124	4680.5333	8.7348	70.6635	124	6588.6667	41.8021	338.1458	124	84.9920	0.6077	4.9159
126	4676.1667	9.0888	72.3606	126	6579.4000	42.5494	338.7322	126	84.9627	0.6206	4.9401

128	4677.2667	9.0966	71.2027	128	6582.2667	43.0158	337.0236	128	84.9243	0.6266	4.9076
130	4672.6000	9.3817	72.3770	130	6573.5667	43.5593	335.9777	130	84.8847	0.6393	4.9335
132	4673.6333	9.4405	71.7159	132	6576.5000	44.2668	336.2924	132	84.8523	0.6458	4.9046
134	4669.6333	9.4872	70.9768	134	6567.6667	44.7228	334.5600	134	84.8087	0.6584	4.9276
136	4669.7667	9.5838	70.6375	136	6570.5667	45.2323	333.3729	136	84.7677	0.6639	4.8944
138	4667.0000	10.2352	74.3745	138	6561.0333	46.0036	334.1574	138	84.7373	0.6780	4.9234
140	4666.7000	9.8202	70.3543	140	6565.1333	46.0764	329.8013	140	84.6713	0.6830	4.8892
142	4665.7667	10.7822	76.0465	142	6556.2333	47.5730	335.6629	142	84.6843	0.6962	4.9144
144	4661.2667	10.0821	70.1998	144	6557.6667	46.9203	326.4895	144	84.5770	0.7032	4.8935
146	4664.2333	10.9470	75.1064	146	6553.8333	48.6495	333.8903	146	84.6157	0.7127	4.8892
148	4657.3333	10.7529	72.8133	148	6547.1333	48.3443	327.4407	148	84.5320	0.7250	4.9052
150	4657.9667	10.6281	70.9742	150	6551.9000	48.7411	325.4995	150	84.4887	0.7281	4.8644
152	4656.9667	11.4988	75.7756	152	6545.0667	50.0985	330.0867	152	84.5150	0.7422	4.8915
154	4651.4667	11.1097	72.2662	154	6541.6333	49.4109	321.3930	154	84.4130	0.7525	4.8955
156	4652.9667	11.1445	71.5739	156	6545.7333	50.0708	321.5564	156	84.3763	0.7559	4.8532
158	4653.5000	11.9282	75.6389	158	6539.4333	51.7981	328.2981	158	84.4133	0.7690	4.8738
160	4648.3333	12.1730	76.2025	160	6531.2000	51.9740	325.2094	160	84.3550	0.7825	4.8955
162	4644.5667	11.5592	71.4080	162	6532.8000	51.1885	316.1426	162	84.2537	0.7882	4.8722
164	4646.6667	11.5548	70.5163	164	6536.1333	51.8222	316.2748	164	84.2333	0.7927	4.8391
166	4648.0333	12.2918	74.1421	166	6532.3667	53.5975	323.2694	166	84.2653	0.8031	4.8428
168	4645.9667	13.0525	77.7712	168	6525.2000	54.8036	326.4709	168	84.2610	0.8165	4.8664
170	4642.7000	13.3379	78.5183	170	6518.8333	55.3507	325.7478	170	84.2123	0.8297	4.8844
172	4639.5000	13.3448	77.6307	172	6514.9333	55.4807	322.7160	172	84.1623	0.8401	4.8905
174	4636.9333	13.2963	76.4660	174	6512.8333	55.3712	318.4999	174	84.1037	0.8495	4.8859
176	4634.8667	13.3095	75.6659	176	6510.4333	55.7127	316.5600	176	84.0640	0.8588	4.8815
178	4633.8000	13.5553	76.1948	178	6508.0333	56.1741	315.8590	178	84.0343	0.8677	4.8787
180	4632.6333	13.8635	77.0492	180	6504.6333	57.0845	317.3275	180	84.0230	0.8784	4.8823
182	4633.1667	14.4319	79.3499	182	6502.0333	58.2505	320.3172	182	84.0200	0.8886	4.8843
184	4634.3667	14.7452	80.1614	184	6501.5333	59.7428	324.6317	184	84.0390	0.8953	4.8701

186	4635.6333	14.6221	78.6575	186	6505.5667	60.4293	325.0149	186	84.0143	0.8986	4.8327
188	4633.1667	13.7200	72.9921	188	6509.7667	59.3152	315.5148	188	83.9200	0.9006	4.7934
190	4626.9667	13.5493	71.2814	190	6503.5333	57.9711	305.3134	190	83.8023	0.9143	4.8119
192	4624.6000	14.8755	77.4711	192	6491.4667	60.3187	314.1675	192	83.8633	0.9363	4.8764
194	4631.4667	15.7052	80.9598	194	6496.6000	62.8000	323.8897	194	83.9293	0.9380	4.8342
196	4625.6333	13.8375	70.5842	196	6502.7667	59.6795	304.6771	196	83.7567	0.9370	4.7819
198	4622.9333	15.8086	79.8615	198	6484.7667	62.4377	315.3065	198	83.8087	0.9656	4.8768
200	4629.1667	15.1408	75.7101	200	6499.6333	63.0496	315.3145	200	83.8117	0.9564	4.7814
**[EMIM][DEP]**				**[EMIM][FSI]**				**[EMIM][MeSO3]**			
Shear rate $\dot{\gamma}$ /s^{-1}	Shear viscosity η/mPa·sec	Deviation between measure-ments ±	u_{95}	Shear rate $\dot{\gamma}$ /s^{-1}	Shear viscosity η/mPa·sec	Deviation between measure-ments ±	u_{95}	Shear rate $\dot{\gamma}$ /s^{-1}	Shear viscosity η/mPa·sec	Deviation between measure-ments ±	u_{95}
1	259.7667	16.1000	9.2953	1	17.1500	2.6990	1.4805	1	112.9263	0.0120	1.8914
3.01	258.6333	15.4000	8.8912	3.01	16.3570	0.9100	0.5189	3.01	112.7897	0.0366	1.4084
5.02	258.5667	15.5000	8.9489	5.02	16.3783	0.3000	0.1511	5.02	112.9883	0.0617	1.3048
7.03	258.3333	16.0000	9.2376	7.03	16.4040	0.1740	0.1005	7.03	112.9913	0.0869	1.1325
9.04	258.2333	16.0000	9.2376	9.04	16.5877	0.3490	0.1998	9.04	112.9817	0.1118	1.1151
11.1	258.1333	15.4000	8.8912	11.1	16.6047	0.1850	0.0944	11.1	112.7873	0.1362	1.2454
13.1	258.2667	15.5000	8.9489	13.1	16.4627	0.0790	0.0440	13.1	112.8117	0.1613	1.3292
15.1	258.0667	15.2000	8.7757	15.1	16.5610	0.2640	0.1425	15.1	112.7993	0.1851	1.2033
17.1	258.4667	14.9000	8.6025	17.1	16.5260	0.0980	0.0526	17.1	112.7877	0.2106	1.3035
19.1	258.1667	14.9000	8.6025	19.1	16.5120	0.1960	0.1079	19.1	112.7393	0.2347	1.1791
21.1	258.3667	14.9000	8.6025	21.1	16.5337	0.0830	0.0419	21.1	112.7513	0.2588	1.2602
23.1	258.0667	14.6000	8.4293	23.1	16.5500	0.2050	0.1074	23.1	112.6900	0.2820	1.1529
25.1	258.2000	14.7000	8.4870	25.1	16.5060	0.0660	0.0373	25.1	112.6867	0.3094	1.2286
27.1	258.0667	14.3000	8.2561	27.1	16.5680	0.1350	0.0733	27.1	112.6030	0.3316	1.0998
29.1	257.8000	14.1000	8.1406	29.1	16.5347	0.1840	0.0938	29.1	112.5443	0.3560	1.0741
31.2	257.8333	14.2000	8.1984	31.2	16.5147	0.0920	0.0462	31.2	112.5860	0.3827	1.1752

33.2	257.8333	14.2000	8.1984	33.2	16.5370	0.0770	0.0433	33.2	112.5297	0.4055	1.1272
35.2	257.6000	13.8000	7.9674	35.2	16.5597	0.1200	0.0621	35.2	112.4483	0.4278	1.0481
37.2	257.3667	13.7000	7.9097	37.2	16.5603	0.1570	0.0785	37.2	112.3810	0.4512	0.9942
39.2	257.1000	13.5000	7.7942	39.2	16.5500	0.1560	0.0785	39.2	112.3313	0.4754	1.0075
41.2	256.9667	13.4000	7.7365	41.2	16.5450	0.1530	0.0769	41.2	112.2953	0.5004	1.0546
43.2	256.7667	13.4000	7.7365	43.2	16.5417	0.1420	0.0717	43.2	112.2507	0.5243	1.0055
45.2	256.5667	13.4000	7.7365	45.2	16.5407	0.1410	0.0711	45.2	112.2150	0.5483	0.9778
47.2	256.4000	13.2000	7.6210	47.2	16.5430	0.1390	0.0698	47.2	112.1550	0.5713	0.9031
49.2	256.1667	13.1000	7.5633	49.2	16.5473	0.1420	0.0710	49.2	112.1043	0.5946	0.8501
51.3	256.1333	13.0000	7.5056	51.3	16.5520	0.1330	0.0665	51.3	112.0520	0.6167	0.8363
53.3	256.0000	12.9000	7.4478	53.3	16.5530	0.1190	0.0600	53.3	112.0270	0.6405	0.8184
55.3	255.9667	12.8000	7.3901	55.3	16.5493	0.1090	0.0555	55.3	112.0287	0.6633	0.8796
57.3	255.7333	13.0000	7.5056	57.3	16.5397	0.1000	0.0506	57.3	111.9747	0.6901	0.9101
59.3	255.3000	12.9000	7.4478	59.3	16.5377	0.1090	0.0545	59.3	111.8970	0.7134	0.8751
61.3	255.0000	12.6000	7.2746	61.3	16.5437	0.1320	0.0660	61.3	111.7977	0.7339	0.7617
63.3	254.9667	12.5000	7.2169	63.3	16.5493	0.1200	0.0602	63.3	111.7870	0.7562	0.7355
65.3	254.9000	12.6000	7.2746	65.3	16.5443	0.1060	0.0532	65.3	111.7763	0.7830	0.8260
67.3	254.4000	12.6000	7.2746	67.3	16.5407	0.1170	0.0585	67.3	111.6747	0.8052	0.7681
69.3	254.3000	12.3000	7.1014	69.3	16.5473	0.1190	0.0595	69.3	111.6080	0.8248	0.6689
71.4	254.2333	12.4000	7.1591	71.4	16.5433	0.1060	0.0534	71.4	111.6197	0.8513	0.7562
73.4	253.7333	12.4000	7.1591	73.4	16.5387	0.1150	0.0577	73.4	111.5073	0.8736	0.7049
75.4	253.7667	12.2000	7.0437	75.4	16.5437	0.1170	0.0589	75.4	111.4733	0.8938	0.6345
77.4	253.5000	12.3000	7.1014	77.4	16.5403	0.1030	0.0516	77.4	111.4340	0.9206	0.7200
79.4	253.2000	12.0000	6.9282	79.4	16.5403	0.1200	0.0602	79.4	111.3360	0.9396	0.5941
81.4	253.1667	12.2000	7.0437	81.4	16.5417	0.1080	0.0543	81.4	111.3430	0.9657	0.6736
83.4	252.7000	12.0000	6.9282	83.4	16.5407	0.1110	0.0557	83.4	111.2437	0.9865	0.5771
85.4	252.7667	11.9000	6.8705	85.4	16.5433	0.1120	0.0562	85.4	111.2343	1.0097	0.6385
87.4	252.2667	11.9000	6.8705	87.4	16.5390	0.1130	0.0568	87.4	111.1317	1.0323	0.6061
89.4	252.3333	11.8000	6.8127	89.4	16.5430	0.1150	0.0577	89.4	111.1247	1.0551	0.6001

91.5	251.8333	11.8000	6.8127	91.5	16.5370	0.1140	0.0572	91.5	111.0240	1.0771	0.5654
93.5	251.8333	11.8000	6.8127	93.5	16.5420	0.1160	0.0581	93.5	111.0203	1.0996	0.5776
95.5	251.4000	11.7000	6.7550	95.5	16.5393	0.1100	0.0552	95.5	110.9300	1.1222	0.5499
97.5	251.4000	11.7000	6.7550	97.5	16.5430	0.1170	0.0587	97.5	110.9247	1.1454	0.5718
99.5	250.9667	11.6000	6.6973	99.5	16.5390	0.1110	0.0558	99.5	110.8247	1.1651	0.5009
102	251.0000	11.7000	6.7550	102	16.5420	0.1070	0.0536	102	110.8270	1.1913	0.5597
104	250.6333	11.5000	6.6395	104	16.5387	0.1120	0.0560	104	110.7397	1.2090	0.4762
106	250.5667	11.6000	6.6973	106	16.5400	0.1070	0.0538	106	110.7317	1.2337	0.5616
108	250.3000	11.4000	6.5818	108	16.5393	0.1160	0.0580	108	110.6537	1.2520	0.4866
110	250.0667	11.6000	6.6973	110	16.5387	0.1030	0.0516	110	110.6363	1.2750	0.5424
112	249.9000	11.4000	6.5818	112	16.5400	0.1180	0.0590	112	110.5737	1.2948	0.4864
114	249.5667	11.6000	6.6973	114	16.5363	0.1060	0.0532	114	110.5223	1.3184	0.5300
116	249.6000	11.4000	6.5818	116	16.5397	0.1140	0.0570	116	110.4893	1.3431	0.4933
118	249.1333	11.5000	6.6395	118	16.5333	0.1070	0.0538	118	110.4017	1.3655	0.4795
120	249.2333	11.5000	6.6395	120	16.5390	0.1150	0.0577	120	110.4060	1.3888	0.5014
122	248.8000	11.4000	6.5818	122	16.5313	0.1080	0.0542	122	110.3090	1.4073	0.4620
124	248.8333	11.5000	6.6395	124	16.5407	0.1020	0.0513	124	110.3210	1.4350	0.4952
126	248.4667	11.3000	6.5241	126	16.5307	0.1080	0.0543	126	110.2240	1.4519	0.4359
128	248.5333	11.5000	6.6395	128	16.5360	0.1080	0.0544	128	110.2363	1.4787	0.5009
130	248.1333	11.2000	6.4663	130	16.5307	0.1120	0.0563	130	110.1407	1.4946	0.4274
132	248.1000	11.4000	6.5818	132	16.5350	0.1110	0.0560	132	110.1540	1.5211	0.5029
134	247.8333	11.2000	6.4663	134	16.5280	0.1140	0.0572	134	110.0383	1.5374	0.4419
136	247.8000	11.4000	6.5818	136	16.5350	0.1080	0.0542	136	110.0530	1.5649	0.5265
138	247.4333	11.2000	6.4663	138	16.5253	0.1050	0.0528	138	109.9530	1.5819	0.4186
140	247.5333	11.2000	6.4663	140	16.5353	0.1160	0.0581	140	109.9890	1.6079	0.4795
142	247.0333	11.2000	6.4663	142	16.5233	0.1020	0.0516	142	109.8737	1.6271	0.4590
144	247.2000	11.1000	6.4086	144	16.5343	0.1210	0.0605	144	109.9017	1.6484	0.4251
146	246.8667	11.3000	6.5241	146	16.5267	0.0960	0.0489	146	109.8130	1.6724	0.5426
148	246.7000	11.1000	6.4086	148	16.5237	0.1140	0.0570	148	109.7613	1.6877	0.3989

150	246.8333	11.2000	6.4663	150	16.5347	0.1160	0.0580	150	109.8023	1.7166	0.4829
152	246.3333	11.2000	6.4663	152	16.5207	0.0930	0.0476	152	109.6987	1.7404	0.4416
154	246.4000	11.1000	6.4086	154	16.5237	0.1200	0.0600	154	109.6797	1.7560	0.3672
156	246.4000	11.1000	6.4086	156	16.5350	0.1180	0.0590	156	109.6890	1.7811	0.4652
158	245.9667	11.3000	6.5241	158	16.5220	0.0890	0.0459	158	109.5960	1.8062	0.4981
160	245.7667	11.0000	6.3509	160	16.5140	0.1040	0.0523	160	109.5270	1.8213	0.3466
162	245.9333	10.9000	6.2931	162	16.5257	0.1290	0.0649	162	109.5403	1.8402	0.3753
164	245.9000	11.1000	6.4086	164	16.5343	0.1220	0.0610	164	109.5453	1.8680	0.4800
166	245.6333	11.2000	6.4663	166	16.5303	0.0970	0.0501	166	109.4733	1.8922	0.5382
168	245.3000	11.1000	6.4086	168	16.5163	0.0810	0.0424	168	109.3823	1.9125	0.5384
170	245.0667	11.0000	6.3509	170	16.5113	0.0820	0.0426	170	109.3143	1.9295	0.4159
172	245.0333	10.9000	6.2931	172	16.5080	0.1030	0.0517	172	109.2857	1.9473	0.4143
174	244.9333	10.9000	6.2931	174	16.5080	0.1140	0.0570	174	109.2563	1.9668	0.3496
176	244.9000	10.8000	6.2354	176	16.5123	0.1140	0.0570	176	109.2420	1.9890	0.3233
178	244.8000	10.8000	6.2354	178	16.5073	0.1210	0.0607	178	109.1960	2.0079	0.3969
180	244.6667	10.7000	6.1776	180	16.5040	0.1120	0.0560	180	109.1450	2.0293	0.3430
182	244.4333	10.9000	6.2931	182	16.5013	0.0910	0.0458	182	109.1000	2.0532	0.4283
184	244.3333	10.9000	6.2931	184	16.5023	0.0750	0.0401	184	109.0727	2.0793	0.5014
186	244.3667	11.0000	6.3509	186	16.5140	0.0710	0.0407	186	109.1017	2.1068	0.5819
188	244.6667	11.0000	6.3509	188	16.5347	0.1100	0.0553	188	109.1783	2.1307	0.6158
190	244.6667	10.7000	6.1776	190	16.5323	0.1560	0.0797	190	109.1687	2.1435	0.3828
192	244.0667	10.7000	6.1776	192	16.4980	0.1190	0.0598	192	108.9913	2.1579	0.3292
194	243.9667	11.0000	6.3509	194	16.5080	0.0700	0.0376	194	108.9790	2.1932	0.6067
196	244.5333	10.9000	6.2931	196	16.5390	0.1470	0.0744	196	109.1240	2.2127	0.4854
198	243.8000	10.8000	6.2354	198	16.4923	0.1020	0.0511	198	108.8970	2.2238	0.4011
200	244.1667	11.0000	6.3509	200	16.5320	0.0920	0.0480	200	109.0177	2.2648	0.6652

[EMIM][OAc]				[EMIM][OOc]				[EMIM][OPr]			
Shear rate $\dot{\gamma}$ /s^{-1}	Shear viscosity η/mPa·sec	Deviation between measure-ments ±	u_{95}	Shear rate $\dot{\gamma}$ /s^{-1}	Shear viscosity η/mPa·sec	Deviation between measure-ments ±	u_{95}	Shear rate $\dot{\gamma}$ /s^{-1}	Shear viscosity η/mPa·sec	Deviation between measure-ments ±	u_{95}
1	109.8233	0.0011	1.1039	1	427.1533	23.4300	12.9910	1	122.3500	0.0022	2.2269
3.01	108.9800	0.0033	1.0835	3.01	427.3633	23.6600	13.4598	3.01	122.4133	0.0108	3.5913
5.02	108.7600	0.0056	1.1221	5.02	427.1733	23.6900	13.6544	5.02	122.3100	0.0187	3.7235
7.03	108.6367	0.0073	1.0365	7.03	427.9767	25.5700	14.6657	7.03	122.2200	0.0271	3.8540
9.04	108.5500	0.0082	0.9102	9.04	427.9400	25.0800	14.3771	9.04	122.1233	0.0343	3.7913
11.1	108.6867	0.0096	0.8686	11.1	427.5800	24.9000	14.2311	11.1	122.1900	0.0409	3.7015
13.1	108.6533	0.0134	1.0189	13.1	427.6800	26.2400	15.0102	13.1	122.1867	0.0491	3.7624
15.1	108.5133	0.0133	0.8796	15.1	427.4600	24.8200	14.1709	15.1	122.1933	0.0559	3.7076
17.1	108.6233	0.0150	0.8765	17.1	427.6233	26.5800	15.1897	17.1	122.1767	0.0637	3.7273
19.1	108.5200	0.0165	0.8642	19.1	427.3367	25.9400	14.8063	19.1	122.1933	0.0714	3.7418
21.1	108.5467	0.0177	0.8424	21.1	427.2267	26.9600	15.4118	21.1	122.1733	0.0785	3.7224
23.1	108.4733	0.0196	0.8474	23.1	427.1600	26.8500	15.3232	23.1	122.1467	0.0861	3.7299
25.1	108.4633	0.0224	0.8903	25.1	426.6900	27.6800	15.8163	25.1	122.0800	0.0940	3.7398
27.1	108.4233	0.0208	0.7671	27.1	426.6833	27.7000	15.7557	27.1	122.0900	0.1014	3.7398
29.1	108.3733	0.0238	0.8175	29.1	426.4000	27.5100	15.6792	29.1	122.0633	0.1075	3.6902
31.2	108.3167	0.0262	0.8429	31.2	425.8200	28.3800	16.1980	31.2	122.0000	0.1151	3.6897
33.2	108.2833	0.0255	0.7711	33.2	425.5733	28.5000	16.1572	33.2	121.9567	0.1237	3.7297
35.2	108.2467	0.0251	0.7145	35.2	425.2833	27.5900	15.5969	35.2	121.9467	0.1313	3.7297
37.2	108.1967	0.0264	0.7114	37.2	425.2967	28.0400	15.8945	37.2	121.9267	0.1373	3.6937
39.2	108.1633	0.0278	0.7081	39.2	424.9267	28.1700	15.9968	39.2	121.8933	0.1436	3.6639
41.2	108.0900	0.0305	0.7375	41.2	424.6933	28.6300	16.2595	41.2	121.8467	0.1501	3.6436
43.2	108.0433	0.0320	0.7420	43.2	424.1000	27.8500	15.8039	43.2	121.8033	0.1570	3.6336
45.2	107.9933	0.0332	0.7333	45.2	424.3533	29.4400	16.7187	45.2	121.7567	0.1642	3.6297
47.2	107.9533	0.0337	0.7165	47.2	423.6533	28.5900	16.4007	47.2	121.7300	0.1708	3.6133
49.2	107.9033	0.0335	0.6831	49.2	424.0200	30.0300	17.2009	49.2	121.6967	0.1775	3.6054

51.3	107.8567	0.0332	0.6470	51.3	423.7233	29.4200	16.8516	51.3	121.6533	0.1852	3.6146
53.3	107.8033	0.0327	0.6160	53.3	423.8567	30.4600	17.4127	53.3	121.6000	0.1927	3.6185
55.3	107.7433	0.0343	0.6201	55.3	423.3833	30.2100	17.2432	55.3	121.4767	0.2023	3.6625
57.3	107.6900	0.0380	0.6655	57.3	423.2300	31.1600	17.8084	57.3	121.4233	0.2077	3.6303
59.3	107.6433	0.0413	0.6976	59.3	422.7567	30.3800	17.3777	59.3	121.3867	0.2120	3.5737
61.3	107.6033	0.0391	0.6379	61.3	423.0000	30.7800	17.5918	61.3	121.3767	0.2184	3.5642
63.3	107.5500	0.0370	0.5859	63.3	422.9033	30.8900	17.6246	63.3	121.3167	0.2276	3.6001
65.3	107.4833	0.0415	0.6352	65.3	422.2600	30.6300	17.4634	65.3	121.2233	0.2347	3.5948
67.3	107.4400	0.0452	0.6696	67.3	422.2100	30.7400	17.5659	67.3	121.1933	0.2368	3.5172
69.3	107.3933	0.0411	0.5918	69.3	422.3467	30.7800	17.5695	69.3	121.1600	0.2457	3.5398
71.4	107.3367	0.0454	0.6352	71.4	421.9100	31.0500	17.6892	71.4	121.0600	0.2542	3.5609
73.4	107.2833	0.0489	0.6650	73.4	421.8867	31.1800	17.8031	73.4	121.0400	0.2557	3.4816
75.4	107.2367	0.0445	0.5907	75.4	421.9167	31.2500	17.8130	75.4	120.9867	0.2659	3.5288
77.4	107.1800	0.0521	0.6720	77.4	421.5500	31.7200	18.1037	77.4	120.8967	0.2712	3.5050
79.4	107.1333	0.0487	0.6086	79.4	421.6000	31.2900	17.8611	79.4	120.8967	0.2764	3.4802
81.4	107.0767	0.0525	0.6439	81.4	421.2033	31.5600	17.9725	81.4	120.7933	0.2867	3.5225
83.4	107.0267	0.0544	0.6503	83.4	421.1333	31.1500	17.7970	83.4	120.7800	0.2881	3.4562
85.4	106.9667	0.0544	0.6379	85.4	420.9967	31.5300	17.9030	85.4	120.7000	0.2981	3.4936
87.4	106.9233	0.0586	0.6698	87.4	421.1533	31.5900	17.7549	87.4	120.6767	0.2989	3.4179
89.4	106.8633	0.0575	0.6408	89.4	421.0267	31.7900	17.8177	89.4	120.5867	0.3119	3.4894
91.5	106.8233	0.0628	0.6863	91.5	420.9133	31.8300	17.8696	91.5	120.5533	0.3125	3.4200
93.5	106.7667	0.0622	0.6637	93.5	420.7033	31.6900	17.7679	93.5	120.4800	0.3241	3.4736
95.5	106.7167	0.0667	0.6995	95.5	420.5200	31.5000	17.6819	95.5	120.4633	0.3238	3.3893
97.5	106.6700	0.0683	0.6974	97.5	420.3033	31.6800	17.7439	97.5	120.3700	0.3363	3.4536
99.5	106.6267	0.0705	0.7112	99.5	420.3067	31.7200	17.7905	99.5	120.3533	0.3381	3.3986
102	106.5700	0.0740	0.7286	102	420.1133	32.1400	17.9961	102	120.2667	0.3494	3.4386
104	106.5233	0.0737	0.7139	104	420.0533	31.7200	17.7774	104	120.2300	0.3525	3.4060
106	106.4767	0.0815	0.7685	106	419.7000	31.8800	17.8592	106	120.1533	0.3590	3.4038
108	106.4300	0.0779	0.7275	108	419.7133	31.5900	17.6765	108	120.1467	0.3653	3.4030

110	106.3833	0.0878	0.7996	110	419.5133	32.0900	17.9777	110	120.0533	0.3697	3.3774
112	106.3333	0.0833	0.7490	112	419.4333	31.7100	17.7534	112	120.0300	0.3793	3.3994
114	106.2900	0.0924	0.8146	114	419.0800	31.5700	17.6623	114	119.9700	0.3789	3.3348
116	106.2367	0.0902	0.7801	116	418.9967	31.5500	17.6173	116	119.9100	0.3927	3.3968
118	106.1967	0.0967	0.8220	118	418.9600	31.7300	17.7649	118	119.8767	0.3893	3.3119
120	106.1467	0.0989	0.8259	120	418.6567	31.5200	17.6077	120	119.8000	0.4038	3.3784
122	106.1067	0.1000	0.8259	122	418.5800	31.1800	17.4298	122	119.7867	0.4026	3.3106
124	106.0533	0.1065	0.8607	124	418.4300	31.7300	17.7184	124	119.6933	0.4151	3.3552
126	106.0067	0.1046	0.8301	126	418.3700	31.1800	17.4221	126	119.7033	0.4142	3.2957
128	105.9633	0.1142	0.8965	128	417.9300	31.1400	17.4119	128	119.5933	0.4257	3.3380
130	105.9200	0.1104	0.8542	130	418.0767	31.1000	17.4122	130	119.6033	0.4272	3.2969
132	105.8767	0.1213	0.9205	132	417.6300	30.9500	17.2998	132	119.4933	0.4369	3.3197
134	105.8367	0.1177	0.8807	134	417.6167	30.3700	16.9888	134	119.5133	0.4381	3.2785
136	105.7867	0.1276	0.9431	136	417.5067	31.2600	17.4604	136	119.4033	0.4506	3.3197
138	105.7467	0.1252	0.9113	138	417.3300	30.2800	16.9034	138	119.4333	0.4479	3.2497
140	105.6967	0.1327	0.9520	140	417.1267	30.7800	17.1762	140	119.3300	0.4654	3.3311
142	105.6567	0.1353	0.9526	142	417.0567	30.3600	16.9805	142	119.3300	0.4562	3.2187
144	105.6100	0.1340	0.9374	144	416.8867	30.1800	16.7999	144	119.2667	0.4772	3.3266
146	105.5667	0.1465	1.0076	146	416.7200	30.5800	17.0763	146	119.2033	0.4708	3.2313
148	105.5267	0.1381	0.9333	148	416.6967	29.8200	16.6230	148	119.2267	0.4810	3.2566
150	105.4800	0.1492	0.9971	150	416.5767	30.8500	17.2037	150	119.1167	0.4962	3.3151
152	105.4400	0.1534	1.0137	152	416.3467	30.0100	16.7427	152	119.1067	0.4850	3.1935
154	105.3933	0.1459	0.9474	154	416.6367	30.4700	16.9873	154	119.1100	0.5022	3.2623
156	105.3500	0.1585	1.0221	156	416.0433	30.0900	16.7735	156	118.9900	0.5160	3.3116
158	105.3167	0.1665	1.0577	158	416.1667	30.6200	17.0891	158	118.9633	0.5039	3.1947
160	105.2700	0.1584	0.9902	160	416.0867	29.6300	16.5264	160	119.0033	0.5090	3.1889
162	105.2200	0.1575	0.9755	162	416.1900	30.2800	16.8423	162	118.9500	0.5311	3.2875
164	105.1767	0.1708	1.0433	164	415.7967	30.2400	16.8497	164	118.8433	0.5419	3.3093
166	105.1400	0.1820	1.1001	166	415.6600	30.3600	16.9239	166	118.7867	0.5365	3.2359

168	105.1067	0.1824	1.0841	168	415.7233	30.4600	17.0278	168	118.8033	0.5280	3.1441
170	105.0633	0.1774	1.0443	170	415.6400	29.8600	16.6538	170	118.7867	0.5364	3.1537
172	105.0233	0.1745	1.0137	172	415.8933	30.3500	16.9284	172	118.8067	0.5421	3.1510
174	104.9867	0.1727	0.9908	174	415.6400	29.7100	16.5444	174	118.7833	0.5540	3.1877
176	104.9433	0.1738	0.9897	176	415.7067	29.9300	16.6762	176	118.7667	0.5615	3.1958
178	104.9033	0.1759	0.9897	178	415.7233	30.1100	16.7672	178	118.7433	0.5670	3.1901
180	104.8667	0.1790	0.9975	180	415.4733	29.5700	16.4790	180	118.7067	0.5691	3.1614
182	104.8367	0.1854	1.0179	182	415.5600	30.2600	16.8638	182	118.6733	0.5675	3.1178
184	104.8000	0.1985	1.0785	184	415.2567	30.0400	16.7677	184	118.5933	0.5677	3.0914
186	104.7700	0.2138	1.1533	186	415.0000	30.3500	16.9386	186	118.5033	0.5828	3.1339
188	104.7233	0.2152	1.1455	188	415.0267	30.9500	17.2485	188	118.4367	0.6131	3.2634
190	104.6867	0.1961	1.0310	190	415.0567	30.0400	16.6688	190	118.5067	0.6335	3.3334
192	104.6567	0.1881	0.9810	192	415.3567	30.0400	16.7370	192	118.5700	0.6025	3.1420
194	104.6433	0.2223	1.1476	194	414.8933	30.6000	17.0520	194	118.4233	0.6026	3.1064
196	104.6000	0.2137	1.0933	196	414.7667	30.2900	16.8153	196	118.3867	0.6556	3.3414
198	104.5733	0.1953	0.9835	198	415.2667	30.2500	16.8580	198	118.5167	0.6145	3.1086
200	104.5567	0.2338	1.1682	200	414.4933	30.4500	16.9579	200	118.2900	0.6454	3.2223
**[EMIM][OTf]**				**[EMIM][SCN]**				**[EMIM][TFSI]**			
Shear rate $\dot{\gamma}$ /s^{-1}	Shear viscosity η/mPa·sec	Deviation between measure-ments ±	u_{95}	Shear rate $\dot{\gamma}$ /s^{-1}	Shear viscosity η/mPa·sec	Deviation between measure-ments ±	u_{95}	Shear rate $\dot{\gamma}$ /s^{-1}	Shear viscosity η/mPa·sec	Deviation between measure-ments ±	u_{95}
1	34.6833	0.0012	1.2422	1	21.1520	0.0032	3.2554	1	28.2710	0.0008	0.7691
3.01	34.6577	0.0031	1.0272	3.01	19.7943	0.0015	0.4814	3.01	28.0847	0.0026	0.8731
5.02	34.7053	0.0049	0.9754	5.02	19.6713	0.0012	0.2395	5.02	28.0083	0.0051	1.0220
7.03	34.6870	0.0064	0.9102	7.03	19.5197	0.0007	0.0926	7.03	27.9313	0.0078	1.1060
9.04	34.7060	0.0084	0.9311	9.04	19.5550	0.0015	0.1639	9.04	27.9517	0.0098	1.0868
11.1	34.7243	0.0107	0.9646	11.1	19.5493	0.0021	0.1932	11.1	27.9673	0.0119	1.0772
13.1	34.7053	0.0123	0.9398	13.1	19.5083	0.0017	0.1263	13.1	27.9570	0.0140	1.0725

15.1	34.7177	0.0142	0.9437	15.1	19.4903	0.0020	0.1318	15.1	27.9443	0.0164	1.0861
17.1	34.7370	0.0167	0.9753	17.1	19.4440	0.0029	0.1702	17.1	27.9540	0.0183	1.0702
19.1	34.7443	0.0185	0.9699	19.1	19.4210	0.0032	0.1697	19.1	27.9527	0.0208	1.0875
21.1	34.7597	0.0210	0.9937	21.1	19.4690	0.0030	0.1420	21.1	27.9643	0.0229	1.0864
23.1	34.7427	0.0225	0.9744	23.1	19.4580	0.0031	0.1331	23.1	27.9610	0.0253	1.0937
25.1	34.7310	0.0242	0.9637	25.1	19.4160	0.0045	0.1775	25.1	27.9663	0.0272	1.0837
27.1	34.7313	0.0262	0.9648	27.1	19.4593	0.0041	0.1508	27.1	27.9647	0.0300	1.1043
29.1	34.7397	0.0284	0.9738	29.1	19.5093	0.0035	0.1193	29.1	27.9693	0.0322	1.1032
31.2	34.7297	0.0301	0.9645	31.2	19.4310	0.0052	0.1671	31.2	27.9700	0.0343	1.1006
33.2	34.7293	0.0321	0.9667	33.2	19.4837	0.0044	0.1315	33.2	27.9737	0.0365	1.0993
35.2	34.7313	0.0343	0.9744	35.2	19.5140	0.0053	0.1506	35.2	27.9750	0.0390	1.1100
37.2	34.7223	0.0358	0.9650	37.2	19.4027	0.0070	0.1870	37.2	27.9767	0.0414	1.1116
39.2	34.7167	0.0378	0.9643	39.2	19.4303	0.0060	0.1530	39.2	27.9773	0.0433	1.1049
41.2	34.7157	0.0394	0.9573	41.2	19.4270	0.0063	0.1519	41.2	27.9803	0.0457	1.1089
43.2	34.7137	0.0415	0.9619	43.2	19.3680	0.0093	0.2148	43.2	27.9853	0.0478	1.1044
45.2	34.7090	0.0433	0.9575	45.2	19.4277	0.0068	0.1497	45.2	27.9853	0.0500	1.1058
47.2	34.7090	0.0455	0.9636	47.2	19.3537	0.0109	0.2300	47.2	27.9870	0.0523	1.1064
49.2	34.7117	0.0477	0.9694	49.2	19.3813	0.0094	0.1903	49.2	27.9860	0.0547	1.1105
51.3	34.7127	0.0495	0.9655	51.3	19.3660	0.0106	0.2076	51.3	27.9843	0.0570	1.1120
53.3	34.6997	0.0515	0.9661	53.3	19.3527	0.0118	0.2222	53.3	27.9823	0.0592	1.1125
55.3	34.6950	0.0532	0.9625	55.3	19.3667	0.0113	0.2044	55.3	27.9833	0.0613	1.1099
57.3	34.6877	0.0544	0.9498	57.3	19.3370	0.0139	0.2429	57.3	27.9887	0.0635	1.1077
59.3	34.6817	0.0559	0.9421	59.3	19.3497	0.0131	0.2205	59.3	27.9973	0.0656	1.1075
61.3	34.6813	0.0584	0.9529	61.3	19.3207	0.0154	0.2509	61.3	27.9950	0.0683	1.1148
63.3	34.6787	0.0605	0.9547	63.3	19.3357	0.0145	0.2300	63.3	27.9887	0.0706	1.1161
65.3	34.6640	0.0611	0.9346	65.3	19.3267	0.0159	0.2437	65.3	27.9920	0.0728	1.1145
67.3	34.6560	0.0623	0.9248	67.3	19.3287	0.0165	0.2454	67.3	28.0007	0.0751	1.1155
69.3	34.6530	0.0648	0.9339	69.3	19.3187	0.0168	0.2417	69.3	27.9920	0.0775	1.1187
71.4	34.6493	0.0662	0.9274	71.4	19.3087	0.0185	0.2586	71.4	27.9893	0.0795	1.1145

73.4	34.6487	0.0678	0.9248	73.4	19.3260	0.0173	0.2354	73.4	28.0007	0.0820	1.1186
75.4	34.6447	0.0702	0.9318	75.4	19.2913	0.0205	0.2716	75.4	27.9927	0.0844	1.1196
77.4	34.6417	0.0711	0.9185	77.4	19.3130	0.0193	0.2502	77.4	28.0003	0.0863	1.1157
79.4	34.6417	0.0738	0.9294	79.4	19.2787	0.0225	0.2831	79.4	27.9947	0.0891	1.1219
81.4	34.6357	0.0750	0.9208	81.4	19.2863	0.0227	0.2791	81.4	27.9970	0.0911	1.1196
83.4	34.6387	0.0770	0.9231	83.4	19.2840	0.0225	0.2692	83.4	27.9883	0.0931	1.1165
85.4	34.6330	0.0790	0.9253	85.4	19.2737	0.0249	0.2916	85.4	27.9830	0.0951	1.1137
87.4	34.6313	0.0806	0.9213	87.4	19.2720	0.0245	0.2801	87.4	27.9910	0.0974	1.1134
89.4	34.6220	0.0823	0.9203	89.4	19.2763	0.0247	0.2757	89.4	27.9823	0.0994	1.1116
91.5	34.6283	0.0844	0.9230	91.5	19.2580	0.0272	0.2968	91.5	27.9920	0.1019	1.1134
93.5	34.6157	0.0859	0.9190	93.5	19.2650	0.0267	0.2860	93.5	27.9833	0.1042	1.1150
95.5	34.6160	0.0873	0.9140	95.5	19.2617	0.0269	0.2817	95.5	27.9940	0.1067	1.1175
97.5	34.6080	0.0891	0.9141	97.5	19.2520	0.0289	0.2967	97.5	27.9867	0.1088	1.1163
99.5	34.6070	0.0910	0.9144	99.5	19.2547	0.0283	0.2846	99.5	27.9893	0.1116	1.1223
102	34.5997	0.0925	0.9118	102	19.2607	0.0286	0.2820	102	27.9863	0.1132	1.1153
104	34.5987	0.0949	0.9169	104	19.2397	0.0312	0.3016	104	27.9873	0.1159	1.1199
106	34.5947	0.0959	0.9084	106	19.2447	0.0310	0.2942	106	27.9917	0.1176	1.1139
108	34.5917	0.0987	0.9181	108	19.2400	0.0308	0.2868	108	27.9830	0.1204	1.1193
110	34.5900	0.0991	0.9047	110	19.2320	0.0333	0.3036	110	27.9940	0.1219	1.1129
112	34.5833	0.1019	0.9134	112	19.2250	0.0335	0.3005	112	27.9843	0.1250	1.1203
114	34.5830	0.1028	0.9051	114	19.2313	0.0329	0.2897	114	27.9927	0.1267	1.1157
116	34.5743	0.1055	0.9125	116	19.2217	0.0349	0.3018	116	27.9780	0.1291	1.1174
118	34.5723	0.1069	0.9090	118	19.2123	0.0364	0.3088	118	27.9890	0.1316	1.1191
120	34.5623	0.1093	0.9135	120	19.2193	0.0352	0.2943	120	27.9803	0.1333	1.1147
122	34.5637	0.1111	0.9140	122	19.2063	0.0366	0.3005	122	27.9853	0.1361	1.1196
124	34.5567	0.1121	0.9074	124	19.2037	0.0388	0.3136	124	27.9820	0.1375	1.1126
126	34.5523	0.1146	0.9124	126	19.1997	0.0376	0.2999	126	27.9813	0.1408	1.1207
128	34.5463	0.1156	0.9051	128	19.2020	0.0390	0.3057	128	27.9850	0.1421	1.1138
130	34.5397	0.1189	0.9176	130	19.1837	0.0403	0.3111	130	27.9800	0.1452	1.1203

132	34.5343	0.1194	0.9068	132	19.1993	0.0394	0.2989	132	27.9830	0.1465	1.1134
134	34.5273	0.1228	0.9184	134	19.1770	0.0413	0.3088	134	27.9793	0.1502	1.1233
136	34.5230	0.1230	0.9069	136	19.1793	0.0423	0.3118	136	27.9810	0.1510	1.1131
138	34.5200	0.1261	0.9159	138	19.1783	0.0408	0.2957	138	27.9793	0.1544	1.1218
140	34.5097	0.1270	0.9094	140	19.1690	0.0439	0.3137	140	27.9740	0.1556	1.1140
142	34.5107	0.1288	0.9085	142	19.1690	0.0454	0.3207	142	27.9843	0.1585	1.1188
144	34.4957	0.1320	0.9184	144	19.1633	0.0451	0.3133	144	27.9660	0.1608	1.1185
146	34.4983	0.1308	0.8981	146	19.1513	0.0477	0.3266	146	27.9883	0.1618	1.1109
148	34.4870	0.1355	0.9174	148	19.1477	0.0453	0.3067	148	27.9710	0.1661	1.1241
150	34.4803	0.1350	0.9018	150	19.1423	0.0485	0.3240	150	27.9713	0.1665	1.1123
152	34.4810	0.1361	0.8969	152	19.1367	0.0492	0.3245	152	27.9863	0.1691	1.1145
154	34.4680	0.1407	0.9150	154	19.1267	0.0489	0.3179	154	27.9557	0.1720	1.1186
156	34.4600	0.1402	0.8999	156	19.1267	0.0511	0.3283	156	27.9600	0.1726	1.1078
158	34.4640	0.1403	0.8892	158	19.1307	0.0505	0.3199	158	27.9817	0.1744	1.1060
160	34.4527	0.1452	0.9085	160	19.1003	0.0530	0.3326	160	27.9657	0.1789	1.1195
162	34.4380	0.1479	0.9147	162	19.1103	0.0514	0.3179	162	27.9463	0.1806	1.1164
164	34.4333	0.1471	0.8982	164	19.1017	0.0551	0.3359	164	27.9553	0.1816	1.1085
166	34.4370	0.1465	0.8831	166	19.1173	0.0544	0.3280	166	27.9737	0.1824	1.0997
168	34.4340	0.1483	0.8842	168	19.0883	0.0578	0.3440	168	27.9813	0.1852	1.1039
170	34.4260	0.1525	0.8977	170	19.0880	0.0546	0.3219	170	27.9717	0.1887	1.1107
172	34.4147	0.1556	0.9055	172	19.0623	0.0587	0.3414	172	27.9603	0.1924	1.1203
174	34.4027	0.1588	0.9132	174	19.0667	0.0565	0.3253	174	27.9510	0.1951	1.1226
176	34.3943	0.1610	0.9153	176	19.0543	0.0590	0.3353	176	27.9473	0.1975	1.1235
178	34.3870	0.1629	0.9161	178	19.0487	0.0598	0.3363	178	27.9463	0.1999	1.1239
180	34.3857	0.1643	0.9138	180	19.0510	0.0588	0.3270	180	27.9500	0.2024	1.1253
182	34.3833	0.1649	0.9067	182	19.0333	0.0636	0.3492	182	27.9613	0.2032	1.1172
184	34.3840	0.1633	0.8877	184	19.0527	0.0610	0.3314	184	27.9753	0.2043	1.1109
186	34.3783	0.1620	0.8713	186	19.0517	0.0653	0.3515	186	27.9820	0.2042	1.0983
188	34.3630	0.1644	0.8750	188	19.0530	0.0672	0.3578	188	27.9617	0.2055	1.0937

190	34.3440	0.1732	0.9118	190	19.0443	0.0627	0.3294	190	27.9257	0.2111	1.1117
192	34.3473	0.1758	0.9161	192	19.0147	0.0650	0.3389	192	27.9400	0.2162	1.1263
194	34.3610	0.1691	0.8715	194	19.0273	0.0716	0.3692	194	27.9840	0.2125	1.0960
196	34.3290	0.1753	0.8946	196	19.0383	0.0678	0.3460	196	27.9327	0.2156	1.0998
198	34.3353	0.1811	0.9152	198	19.0107	0.0669	0.3382	198	27.9423	0.2223	1.1226
200	34.3370	0.1724	0.8621	200	19.0467	0.0757	0.3787	200	27.9673	0.2178	1.0890
**[HEXMIM][ACR]**				**[Pyrr][OAc]**				**[Pyrr][OFm]**			
Shear rate $\dot{\gamma}$ /s^{-1}	Shear viscosity η/mPa·sec	Deviation between measure-ments ±	u_{95}	Shear rate $\dot{\gamma}$ /s^{-1}	Shear viscosity η/mPa·sec	Deviation between measure-ments ±	u_{95}	Shear rate $\dot{\gamma}$ /s^{-1}	Shear viscosity η/mPa·sec	Deviation between measure-ments ±	u_{95}
1	465.6633	0.0084	8.4208	1	28.3387	3.9770	2.1746	1	4.1956	1.2108	0.6460
3.01	466.2233	0.0259	8.6200	3.01	27.5143	1.0710	0.5764	3.01	4.3480	0.5165	0.2583
5.02	464.9933	0.0430	8.5635	5.02	27.3807	0.7610	0.4085	5.02	4.3869	0.1912	0.0978
7.03	465.2900	0.0630	8.9543	7.03	27.3577	0.5580	0.3013	7.03	4.3855	0.0457	0.0233
9.04	465.6067	0.0822	9.0864	9.04	27.5177	0.3400	0.1827	9.04	4.3466	0.1543	0.0845
11.1	465.2233	0.1002	9.0677	11.1	27.4443	0.7450	0.3726	11.1	4.3203	0.3742	0.2003
13.1	464.7067	0.1165	8.9165	13.1	27.4223	0.5600	0.2815	13.1	4.3620	0.2489	0.1268
15.1	465.2467	0.1375	9.1219	15.1	27.3647	0.7360	0.4080	15.1	4.3483	0.2245	0.1171
17.1	464.5100	0.1528	8.9474	17.1	27.4110	0.7730	0.3865	17.1	4.3480	0.3465	0.1757
19.1	464.7633	0.1744	9.1308	19.1	27.3467	0.7490	0.4085	19.1	4.3626	0.2172	0.1088
21.1	464.0667	0.1891	8.9592	21.1	27.3973	0.7710	0.3877	21.1	4.3609	0.3239	0.1620
23.1	464.4467	0.2117	9.1585	23.1	27.3330	0.8030	0.4349	23.1	4.3538	0.2521	0.1273
25.1	463.3167	0.2254	8.9762	25.1	27.3507	0.8160	0.4140	25.1	4.3524	0.2719	0.1390
27.1	463.5333	0.2495	9.1969	27.1	27.3803	0.7590	0.3908	27.1	4.3413	0.3067	0.1598
29.1	463.4967	0.2681	9.2060	29.1	27.3563	0.7580	0.4074	29.1	4.3488	0.2594	0.1339
31.2	462.4800	0.2822	9.0640	31.2	27.3330	0.8280	0.4286	31.2	4.3689	0.2274	0.1178
33.2	462.1033	0.3046	9.1810	33.2	27.3583	0.7800	0.3989	33.2	4.3545	0.3032	0.1553
35.2	462.2333	0.3302	9.3913	35.2	27.3810	0.7220	0.3743	35.2	4.3483	0.3062	0.1565

37.2	462.2533	0.3494	9.4043	37.2	27.3460	0.7620	0.4037	37.2	4.4070	0.2100	0.1062
39.2	462.0800	0.3669	9.3613	39.2	27.3803	0.6530	0.3431	39.2	4.3652	0.2461	0.1321
41.2	461.6400	0.3864	9.3782	41.2	27.3753	0.6730	0.3658	41.2	4.3519	0.2838	0.1454
43.2	461.4133	0.4015	9.2988	43.2	27.3580	0.6860	0.3627	43.2	4.4603	0.2427	0.1290
45.2	461.0333	0.4228	9.3519	45.2	27.3680	0.6420	0.3353	45.2	4.4313	0.2057	0.1051
47.2	460.9100	0.4416	9.3532	47.2	27.3597	0.6610	0.3492	47.2	4.3504	0.2835	0.1455
49.2	460.7067	0.4656	9.4532	49.2	27.3640	0.6440	0.3405	49.2	4.5043	0.3752	0.1877
51.3	460.6233	0.4827	9.4187	51.3	27.3683	0.6300	0.3324	51.3	4.3517	0.2846	0.1467
53.3	460.0867	0.5076	9.5277	53.3	27.3893	0.5590	0.2910	53.3	4.3566	0.2858	0.1455
55.3	459.5633	0.5242	9.4788	55.3	27.3663	0.6250	0.3251	55.3	4.4568	0.1705	0.0980
57.3	459.2967	0.5306	9.2633	57.3	27.3663	0.6220	0.3225	57.3	4.3538	0.2799	0.1428
59.3	459.3367	0.5382	9.0752	59.3	27.4240	0.4160	0.2109	59.3	4.3518	0.2805	0.1444
61.3	459.1867	0.5840	9.5261	61.3	27.3383	0.6630	0.3529	61.3	4.3494	0.2906	0.1495
63.3	458.9500	0.6037	9.5380	63.3	27.3630	0.6030	0.3186	63.3	4.3543	0.2844	0.1448
65.3	458.1400	0.6225	9.5275	65.3	27.3910	0.5560	0.2968	65.3	4.3523	0.2779	0.1427
67.3	458.2667	0.6421	9.5378	67.3	27.3530	0.6250	0.3349	67.3	4.3590	0.2913	0.1470
69.3	458.3333	0.6707	9.6758	69.3	27.3513	0.6180	0.3328	69.3	4.3514	0.2877	0.1471
71.4	457.6533	0.6833	9.5799	71.4	27.3350	0.6650	0.3567	71.4	4.3524	0.2786	0.1430
73.4	457.7033	0.7049	9.6051	73.4	27.3267	0.6820	0.3647	73.4	4.3500	0.2913	0.1498
75.4	457.5967	0.7283	9.6597	75.4	27.3657	0.5750	0.3089	75.4	4.3522	0.2812	0.1438
77.4	457.0467	0.7409	9.5781	77.4	27.3163	0.6920	0.3668	77.4	4.3521	0.2895	0.1492
79.4	457.2333	0.7723	9.7253	79.4	27.3157	0.6910	0.3711	79.4	4.3520	0.2892	0.1481
81.4	456.5833	0.7867	9.6632	81.4	27.3050	0.7000	0.3764	81.4	4.3518	0.2771	0.1425
83.4	456.7467	0.8113	9.7250	83.4	27.3023	0.7010	0.3781	83.4	4.3514	0.2938	0.1507
85.4	456.1567	0.8320	9.7412	85.4	27.3260	0.6330	0.3399	85.4	4.3511	0.2760	0.1420
87.4	456.3000	0.8467	9.6855	87.4	27.2927	0.7140	0.3834	87.4	4.3493	0.2914	0.1498
89.4	455.8367	0.8745	9.7771	89.4	27.3067	0.6710	0.3618	89.4	4.3517	0.2755	0.1414
91.5	455.8300	0.8945	9.7781	91.5	27.3020	0.6700	0.3625	91.5	4.3489	0.2945	0.1516
93.5	455.3633	0.9198	9.8422	93.5	27.2960	0.6700	0.3606	93.5	4.3510	0.2721	0.1400

95.5	455.4833	0.9355	9.7987	95.5	27.2927	0.6830	0.3667	95.5	4.3492	0.2923	0.1497
97.5	454.9300	0.9620	9.8655	97.5	27.3033	0.6620	0.3604	97.5	4.3500	0.2707	0.1394
99.5	455.1533	0.9831	9.8820	99.5	27.2833	0.6730	0.3625	99.5	4.3493	0.2894	0.1488
102	454.6700	0.9986	9.8385	102	27.2973	0.6740	0.3701	102	4.3468	0.2748	0.1419
104	454.7233	1.0279	9.9293	104	27.2797	0.6810	0.3711	104	4.3493	0.2844	0.1463
106	454.1700	1.0501	9.9486	106	27.2943	0.6290	0.3389	106	4.3478	0.2788	0.1435
108	454.4133	1.0746	9.9923	108	27.2730	0.6810	0.3699	108	4.3501	0.2808	0.1438
110	454.0067	1.0860	9.9164	110	27.2833	0.6780	0.3691	110	4.3472	0.2874	0.1480
112	453.9533	1.1159	10.0083	112	27.2797	0.6610	0.3619	112	4.3512	0.2743	0.1406
114	453.8233	1.1334	9.9762	114	27.2793	0.6520	0.3538	114	4.3472	0.2950	0.1520
116	453.5500	1.1591	10.0257	116	27.2733	0.6630	0.3619	116	4.3500	0.2685	0.1381
118	453.5367	1.1807	10.0410	118	27.2717	0.6670	0.3606	118	4.3481	0.2959	0.1522
120	453.1500	1.1992	10.0216	120	27.2693	0.6590	0.3580	120	4.3513	0.2765	0.1430
122	453.3000	1.2282	10.1044	122	27.2577	0.6590	0.3562	122	4.3487	0.2963	0.1520
124	452.7900	1.2442	10.0623	124	27.2657	0.6550	0.3585	124	4.3475	0.2757	0.1425
126	452.9633	1.2745	10.1450	126	27.2593	0.6350	0.3456	126	4.3493	0.2875	0.1474
128	452.4500	1.2873	10.0915	128	27.2650	0.6170	0.3312	128	4.3472	0.2792	0.1445
130	452.6300	1.3216	10.1969	130	27.2597	0.6370	0.3510	130	4.3500	0.2840	0.1456
132	452.2233	1.3245	10.0643	132	27.2590	0.6400	0.3422	132	4.3464	0.2792	0.1445
134	452.4533	1.3591	10.1700	134	27.2493	0.6310	0.3452	134	4.3505	0.2854	0.1462
136	451.9400	1.3685	10.0822	136	27.2637	0.6300	0.3467	136	4.3461	0.2747	0.1425
138	452.1700	1.4082	10.2248	138	27.2613	0.6010	0.3266	138	4.3495	0.2919	0.1496
140	451.6867	1.4176	10.1476	140	27.2667	0.6020	0.3311	140	4.3483	0.2674	0.1383
142	451.8800	1.4419	10.1745	142	27.2497	0.6200	0.3366	142	4.3478	0.3026	0.1557
144	451.5300	1.4692	10.2261	144	27.2523	0.5990	0.3328	144	4.3527	0.2579	0.1323
146	451.4267	1.4784	10.1465	146	27.2520	0.6110	0.3290	146	4.3443	0.3009	0.1562
148	451.5967	1.5152	10.2601	148	27.2517	0.5860	0.3269	148	4.3533	0.2782	0.1417
150	451.0200	1.5244	10.1829	150	27.2553	0.5750	0.3168	150	4.3473	0.2655	0.1378
152	451.2400	1.5528	10.2320	152	27.2533	0.5890	0.3218	152	4.3471	0.3128	0.1607

154	451.1900	1.5902	10.3389	154	27.2463	0.5860	0.3363	154	4.3552	0.2708	0.1376
156	450.6367	1.5921	10.2183	156	27.2477	0.5840	0.3260	156	4.3485	0.2633	0.1365
158	450.7300	1.6165	10.2426	158	27.2457	0.5900	0.3207	158	4.3434	0.3125	0.1620
160	450.9733	1.6522	10.3399	160	27.2367	0.5770	0.3232	160	4.3521	0.3025	0.1545
162	450.6600	1.6780	10.3697	162	27.2360	0.5870	0.3369	162	4.3551	0.2500	0.1271
164	450.1467	1.6857	10.2907	164	27.2443	0.5700	0.3243	164	4.3491	0.2545	0.1321
166	450.0533	1.7032	10.2665	166	27.2497	0.5730	0.3176	166	4.3406	0.2938	0.1535
168	450.2433	1.7326	10.3216	168	27.2437	0.5690	0.3129	168	4.3415	0.3233	0.1676
170	450.3633	1.7752	10.4517	170	27.2377	0.5610	0.3138	170	4.3474	0.3258	0.1672
172	450.4500	1.7928	10.4312	172	27.2237	0.5670	0.3212	172	4.3536	0.3069	0.1563
174	450.3167	1.8258	10.4989	174	27.2260	0.5650	0.3214	174	4.3559	0.2921	0.1482
176	450.2400	1.8471	10.4992	176	27.2197	0.5730	0.3280	176	4.3567	0.2751	0.1391
178	450.1633	1.8713	10.5204	178	27.2167	0.5430	0.3132	178	4.3576	0.2738	0.1386
180	450.1400	1.8914	10.5137	180	27.2143	0.5580	0.3216	180	4.3564	0.2869	0.1450
182	450.1400	1.9080	10.4901	182	27.2147	0.5480	0.3084	182	4.3512	0.3122	0.1586
184	449.8800	1.9158	10.4131	184	27.2237	0.5370	0.2947	184	4.3433	0.3410	0.1749
186	449.4067	1.9227	10.3400	186	27.2387	0.5260	0.2865	186	4.3353	0.3363	0.1759
188	448.9400	1.9407	10.3243	188	27.2420	0.5410	0.3092	188	4.3391	0.2702	0.1433
190	449.1967	1.9940	10.4954	190	27.2140	0.5760	0.3234	190	4.3562	0.2099	0.1069
192	449.8867	2.0081	10.4613	192	27.2043	0.5560	0.3148	192	4.3598	0.2789	0.1408
194	449.4067	1.9923	10.2676	194	27.2357	0.5260	0.2879	194	4.3356	0.3572	0.1855
196	448.7133	2.0544	10.4806	196	27.2237	0.5600	0.3145	196	4.3491	0.2110	0.1102
198	449.6767	2.1043	10.6299	198	27.2030	0.5320	0.3040	198	4.3579	0.3023	0.1527
200	448.5467	2.0790	10.3949	200	27.2367	0.5300	0.3004	200	4.3306	0.2971	0.1592

[TEAH][MeSO3]			
Shear rate $\dot{\gamma}$ /s^{-1}	Shear viscosity η/mPa·sec	Deviation between measure-ments ±	u_{95}
1	89.1357	0.0029	2.8650
3.01	89.3910	0.0079	2.6404
5.02	89.3670	0.0133	2.6569
7.03	89.2640	0.0187	2.6666
9.04	89.1823	0.0242	2.6761
11.1	89.2193	0.0293	2.6516
13.1	89.3210	0.0352	2.6944
15.1	89.1940	0.0389	2.5766
17.1	89.1943	0.0436	2.5515
19.1	89.2443	0.0502	2.6291
21.1	89.1947	0.0540	2.5575
23.1	89.2003	0.0604	2.6145
25.1	89.2417	0.0662	2.6372
27.1	89.2063	0.0713	2.6276
29.1	89.2190	0.0781	2.6820
31.2	89.2067	0.0836	2.6841
33.2	89.2087	0.0877	2.6449
35.2	89.1870	0.0934	2.6547
37.2	89.1780	0.0999	2.6854
39.2	89.1647	0.1062	2.7095
41.2	89.1617	0.1123	2.7252
43.2	89.1543	0.1182	2.7360
45.2	89.1377	0.1236	2.7320
47.2	89.1287	0.1291	2.7341
49.2	89.1197	0.1348	2.7373

51.3	89.1117	0.1397	2.7259
53.3	89.0933	0.1449	2.7207
55.3	89.0747	0.1507	2.7266
57.3	89.0570	0.1581	2.7604
59.3	88.9733	0.1580	2.6640
61.3	88.9640	0.1629	2.6579
63.3	88.9453	0.1671	2.6388
65.3	88.9313	0.1736	2.6577
67.3	88.9030	0.1800	2.6726
69.3	88.8900	0.1841	2.6554
71.4	88.8677	0.1896	2.6574
73.4	88.8463	0.1967	2.6814
75.4	88.8357	0.2006	2.6616
77.4	88.8103	0.2075	2.6814
79.4	88.7997	0.2130	2.6834
81.4	88.7813	0.2189	2.6887
83.4	88.7550	0.2258	2.7077
85.4	88.7333	0.2302	2.6952
87.4	88.7097	0.2375	2.7165
89.4	88.6893	0.2412	2.6963
91.5	88.6620	0.2485	2.7169
93.5	88.6470	0.2529	2.7069
95.5	88.6187	0.2595	2.7173
97.5	88.6013	0.2635	2.7022
99.5	88.5780	0.2700	2.7133
102	88.5550	0.2742	2.7008
104	88.5403	0.2811	2.7149
106	88.5077	0.2863	2.7133
108	88.4873	0.2907	2.7032

110	88.4430	0.2964	2.7084
112	88.4307	0.3010	2.6978
114	88.3993	0.3080	2.7096
116	88.3737	0.3109	2.6918
118	88.3407	0.3175	2.7007
120	88.3210	0.3213	2.6850
122	88.2960	0.3279	2.6942
124	88.2543	0.3319	2.6820
126	88.2393	0.3370	2.6818
128	88.1993	0.3415	2.6740
130	88.1890	0.3461	2.6692
132	88.1463	0.3510	2.6659
134	88.1347	0.3555	2.6597
136	88.0927	0.3597	2.6504
138	88.0870	0.3649	2.6489
140	88.0510	0.3680	2.6318
142	88.0227	0.3754	2.6513
144	88.0157	0.3777	2.6256
146	87.9627	0.3841	2.6356
148	87.9737	0.3869	2.6162
150	87.9273	0.3896	2.5998
152	87.8987	0.3982	2.6233
154	87.9037	0.4000	2.6020
156	87.8567	0.4032	2.5872
158	87.8283	0.4109	2.6038
160	87.8433	0.4146	2.5947
162	87.8220	0.4155	2.5674
164	87.7690	0.4204	2.5650
166	87.7323	0.4283	2.5810

168	87.7233	0.4343	2.5867
170	87.7250	0.4404	2.5908
172	87.7353	0.4423	2.5734
174	87.7230	0.4455	2.5641
176	87.7030	0.4506	2.5634
178	87.6853	0.4550	2.5587
180	87.6663	0.4608	2.5616
182	87.6443	0.4659	2.5617
184	87.6033	0.4731	2.5715
186	87.5690	0.4750	2.5564
188	87.5407	0.4749	2.5274
190	87.5783	0.4768	2.5082
192	87.5937	0.4884	2.5455
194	87.5320	0.4940	2.5459
196	87.5027	0.4908	2.5062
198	87.5697	0.5033	2.5429
200	87.4607	0.5018	2.5102

Determination of ion pair volumes

The method was adapted from Slattery et al.[197] It is not necessary to know the precise crystal structure of the liquid under investigation and we can use the following approach:

We can determine the sizes of certain ions by looking at crystals containing similar ions of known sizes (Eq S2). These known sizes can be accessed via databases, e.g., the CCDC database. For instance, we can look at the crystal size of sodium formate[198] (V_{SF}=229.53 Å³, Z=4, Z'=1). By knowing the size of the sodium cation[199] (V_{Na+}=3.94 Å³) we can now get the information about the formate anion [OFm]⁻. Be aware of Z, which refers to the number of sodium formate molecules in one crystal lattice. Therefore 229.53 Å³ (V_{SF}) needs to be divided by 4 (Z) and then simply subtracted by 3.94 Å³ (V_{Na+}).

$$V_{OFm^-} = \frac{V_{SF}}{Z} - V_{Na^+} \qquad \text{Eq S2}$$

This leads to the size of 53.44 Å³ [OFm]⁻ anion. By doing this for multiple known crystal lattices, the end result gets more precise e.g., using additionally ammonium formate. Even if we lack the structure of a crystal with a specific ion, we can estimate its size by looking at similar ions in a series. By comparing different alkyl chain lengths, for instance, we can make an educated guess about the ion's size. This estimation is quicker and simpler than determining the crystal structure. However, we need to be cautious about where we start this estimation in the series.

Table S10. Determination of crystal volumina

Ionic liquids	**$V_{IonPair}$ [Å³]**	**References**
[BMIM][ACR]	287.57	197, 200
[BMIM][OAc]	268.36	197, 201
[Chol][Lys]	560.66	202-204
[DBUH][OAc]	404.49	201, 205
[EMIM][ACR]	247.57	197, 200
[EMIM][DEP]	359.41	197, 199, 206
[EMIM][MeSO₃]	282.17	197, 207
[EMIM][OAc]	228.36	197, 201
[EMIM][OOc]	377.15	197, 208
[EMIM][OPr]	256.66	197, 209
[EMIM][OTf]	272.60	197, 210
[EMIM][SCN]	269.76	197, 211-214
[EMIM][TFSI]	388.00	197, 203
[HEXMIM][ACR]	333.57	197, 200
[Pyrr][OAc]	181.24	201, 215
[Pyrr][Ofm]	165.24	198, 199, 215, 216
[TEAH][MeSO₃]	285.13	199, 207, 217

Shear viscosity versus factor C

Table S11. Data of the averaged shear viscosity and its maximum deviation between three independent measurements and Factor C including its deviation stemming from measurement uncertainties during surface tension determination.

	**Shear viscosity *η*** mPa·sec	**Std. deviation (95%)** mPa·sec	**Factor *C*** mN·nm²	**± deviation** mN·nm²
[BMIM][ACR]	279.7714	11.18944	8.3969E-09	2.1000E-10
[BMIM][OAc]	304.5269	5.51814	8.2360E-09	1.7000E-10
[Chol][Lys]	4717.72767	75.66102	1.4898E-08	6.3000E-10
[DBUH][OAc]	6626.00133	356.41877	1.5978E-08	8.3000E-10
[EMIM][ACR]	85.63728	5.02196	8.3850E-09	1.8000E-10
[EMIM][DEP]	251.29667	7.0939	1.0520E-08	2.5000E-10
[EMIM][MeSO3]	110.90722	0.6983	9.5542E-09	3.5000E-10
[EMIM][OAc]	106.6652	0.8502	8.0109E-09	1.0200E-09
[EMIM][OOc]	420.50117	16.80966	1.3230E-08	9.6000E-10
[EMIM][OPr]	120.36097	3.42391	7.7895E-09	4.3000E-10
[EMIM][OTf]	34.57968	0.92943	8.7940E-09	4.3000E-10
[EMIM][SCN]	19.28021	0.29963	7.0865E-09	4.0000E-10
[EMIM][TFSI]	27.97976	1.10375	9.5099E-09	3.7000E-10
[HEXMIM][ACR]	455.98737	9.79848	9.3732E-09	9.3000E-10
[Pyrr][OAc]	27.31367	0.3635	6.2238E-09	5.8000E-10
[Pyrr][OFm]	4.35439	0.149	6.7519E-09	5.4000E-10
[TEAH][MeSO3]	88.49301	2.64987	9.5091E-09	6.5000E-10

NMR spectra

[BMIM][ACR]

^{1}H NMR (300 MHz, D_2O) δ 8.66 (s, 1H), 7.39 (dt, *J* = 14.6, 2.0 Hz, 2H), 6.11 – 5.85 (m, 2H), 5.55 (dd, *J* = 9.8, 2.2 Hz, 1H), 4.12 (t, *J* = 7.2 Hz, 2H), 3.83 (s, 3H), 1.77 (p, *J* = 7.3 Hz, 2H), 1.24 (h, *J* = 7.3 Hz, 2H), 0.85 (t, *J* = 7.4 Hz, 3H). ^{13}C NMR (76 MHz, D_2O) δ 174.93, 135.74, 133.99, 126.16, 123.44, 122.18, 49.20, 35.58, 31.21, 18.71, 12.63.

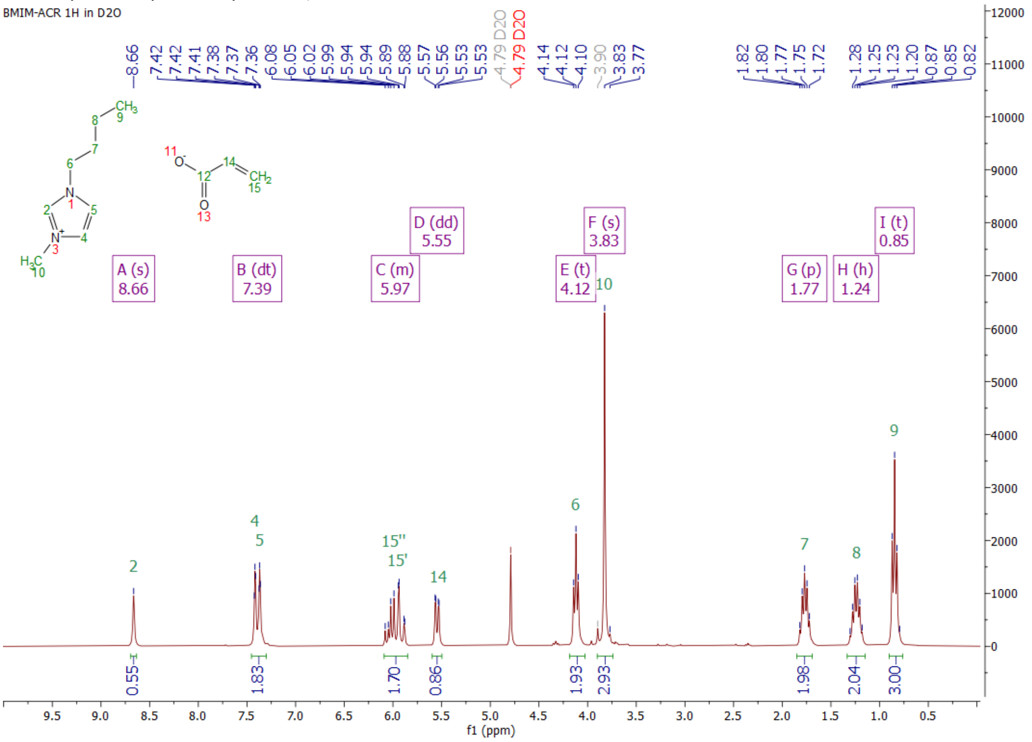

Figure S14. ^{1}H NMR of [BMIM][ACR] in D_2O

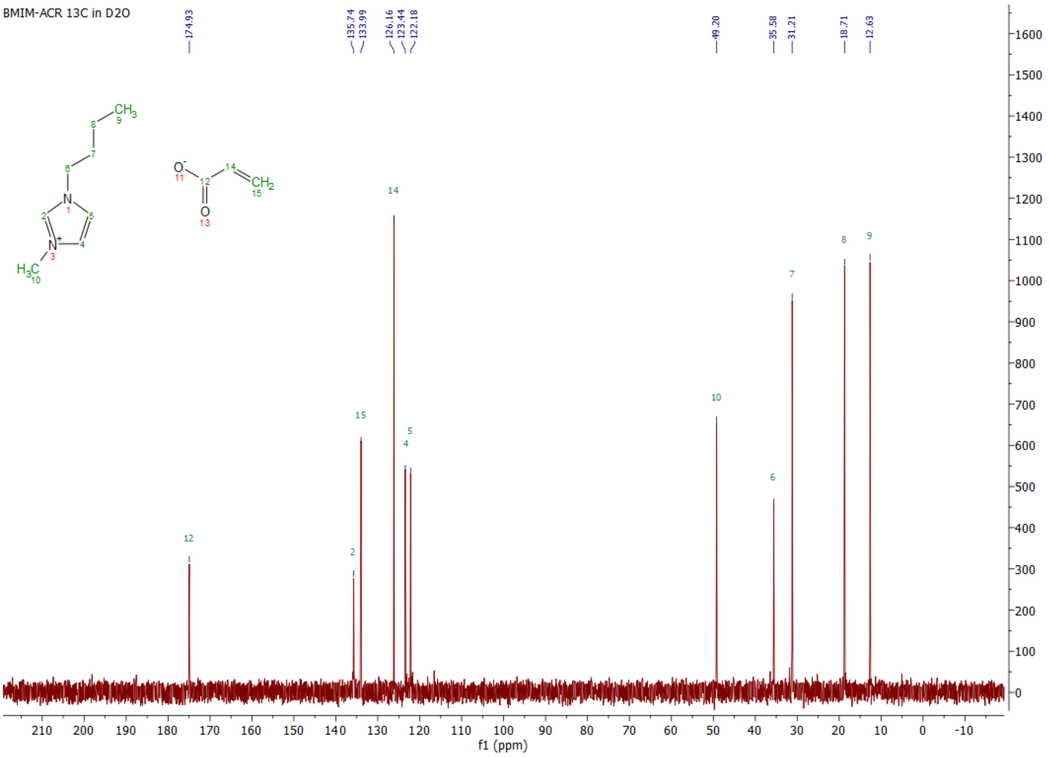

Figure S15. ^{13}C NMR of [BMIM][ACR] in D_2O

[BMIM][OAc]:

^{1}H NMR (300 MHz, Water) δ 8.67 (s, 1H), 7.39 (dd, *J* = 13.1, 1.8 Hz, 2H), 4.12 (t, *J* = 7.1 Hz, 2H), 3.82 (s, 3H), 1.81 (s, 3H), 1.75 (q, *J* = 7.3 Hz, 4H), 1.23 (h, *J* = 7.4 Hz, 2H), 0.83 (t, *J* = 7.4 Hz, 3H). ^{13}C NMR (76 MHz, D_2O) δ 180.62, 135.79, 123.44, 122.17, 121.71, 49.19, 42.13, 35.57, 31.84, 31.21, 23.24, 18.76, 18.70, 12.61, 10.53.

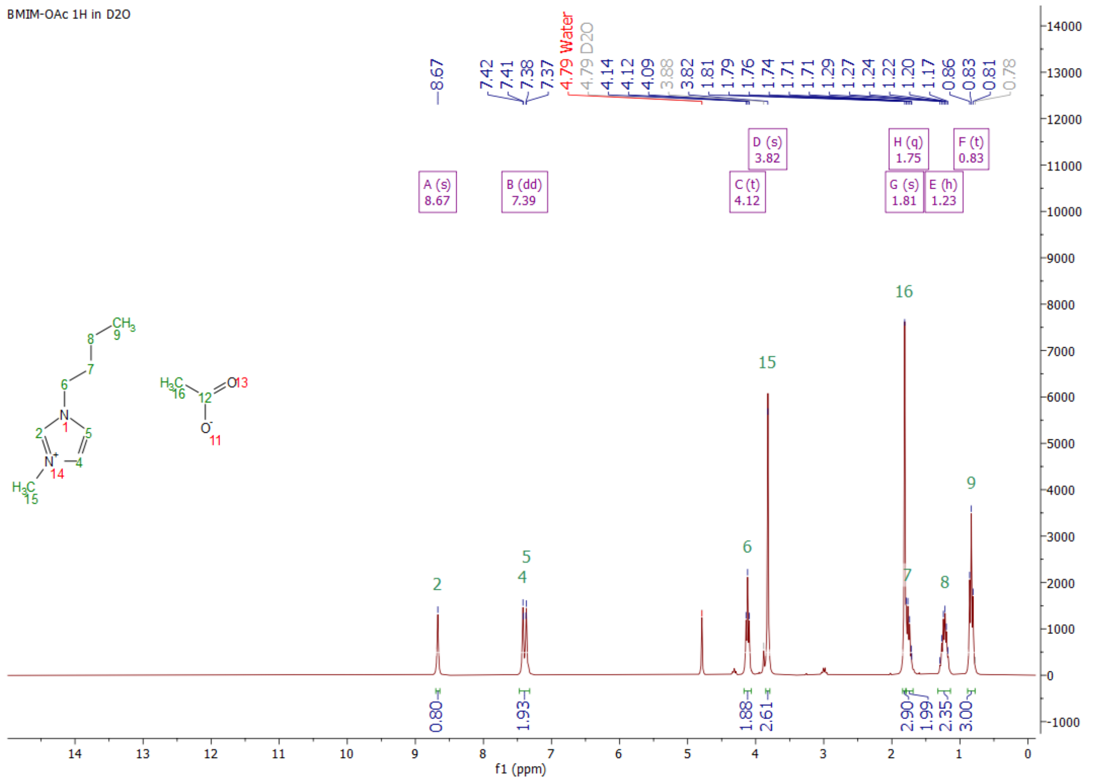

Figure S16. ^{1}H NMR of [BMIM][OAc] in D_2O

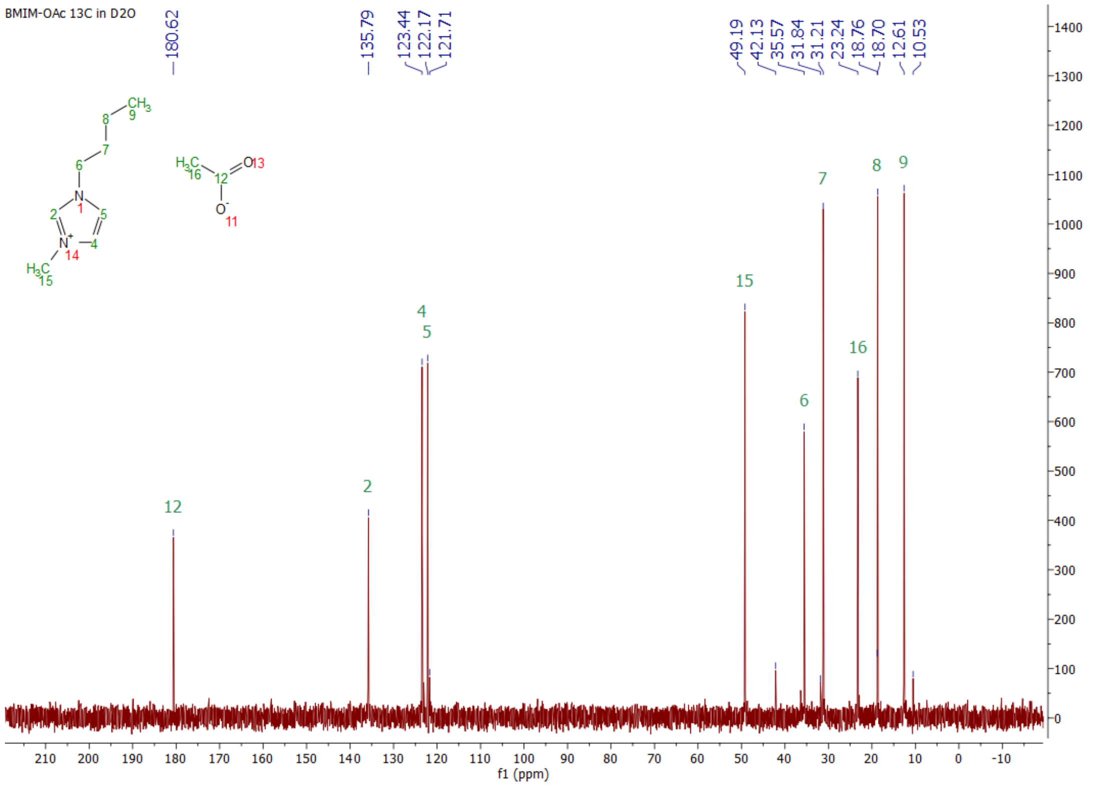

Figure S17. ^{13}C NMR of [BMIM][OAc] in D_2O

[BMPyrr][FSI]

^{1}H NMR (300 MHz, DMSO) δ 3.46 (s, 1H), 3.44 (s, 1H), 3.44 – 3.25 (m, 1H), 2.99 (s, 2H), 2.16 – 2.05 (m, 2H), 1.70 (dtd, J = 16.2, 7.6, 4.8 Hz, 1H), 1.32 (p, J = 7.4 Hz, 1H), 0.94 (t, J = 7.3 Hz, 2H). ^{13}C NMR (76 MHz, DMSO) δ 63.62, 63.58, 63.53, 63.24, 63.20, 63.16, 47.65, 47.60, 47.55, 40.35, 40.08, 39.80, 39.52, 39.24, 38.96, 38.69, 24.95, 21.11, 19.27, 13.25.

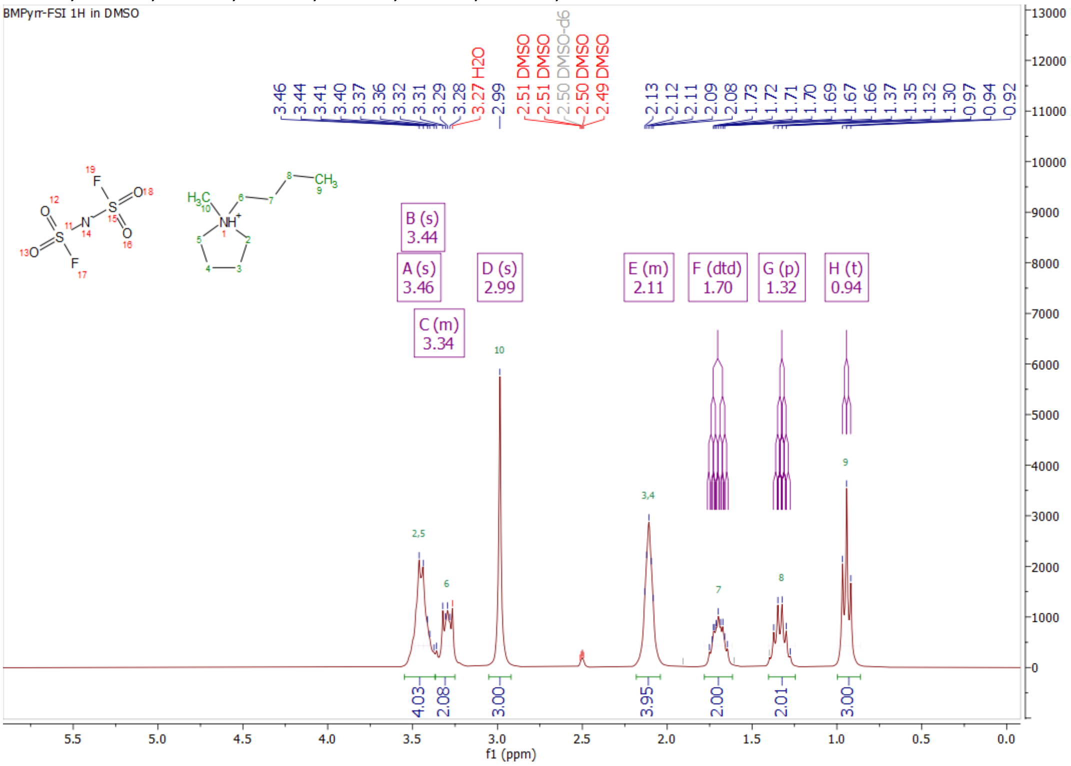

Figure S18. ^{1}H NMR of [BMPyrr][FSI] in d_6-DMSO

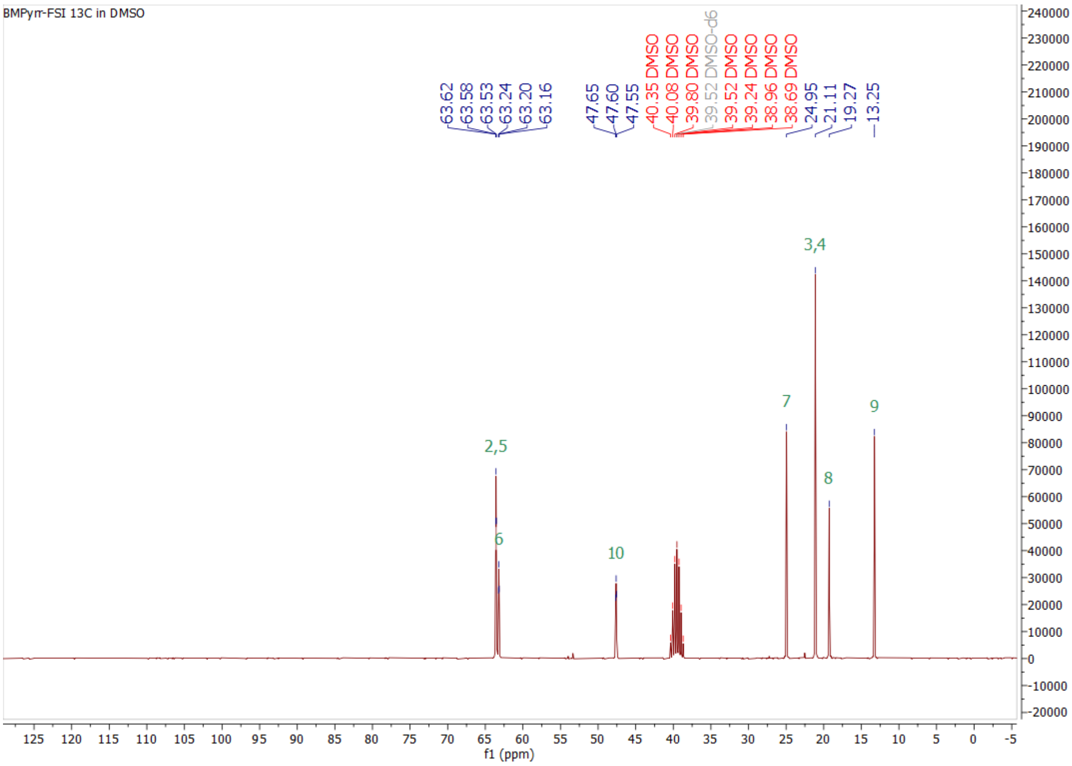

Figure S19. ^{13}C NMR of [BMPyrr][FSI] in d_6-DMSO

[Chol][Lys]

^{1}H NMR (300 MHz, D_2O) δ 4.18 – 3.88 (m, 2H), 3.58 – 3.37 (t, 2H), 3.17 (s, 9H), 2.60 (t, *J* = 7.0 Hz, 2H), 1.66 – 1.20 (m, 6H). ^{13}C NMR (76 MHz, D_2O) δ 183.57, 67.43, 55.92, 55.55, 53.90, 53.84, 53.79, 40.27, 34.42, 31.26, 22.34.

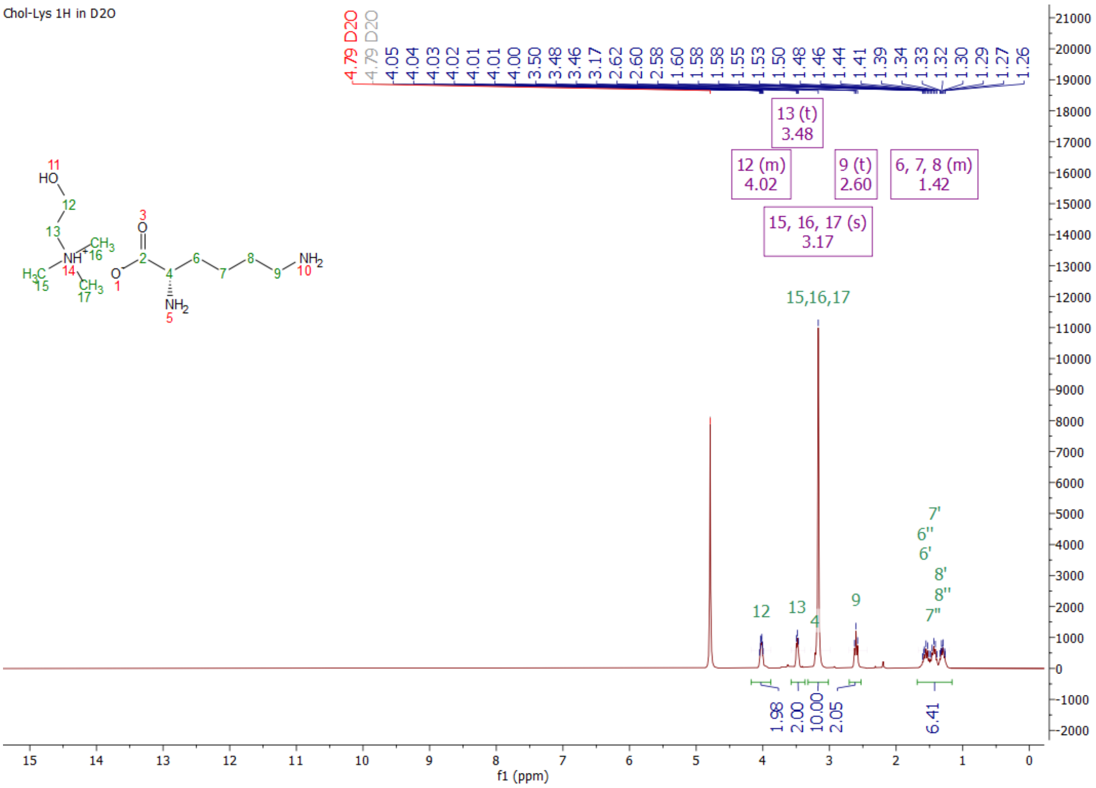

Figure S20. ^{1}H NMR of [Chol][Lys] in D_2O

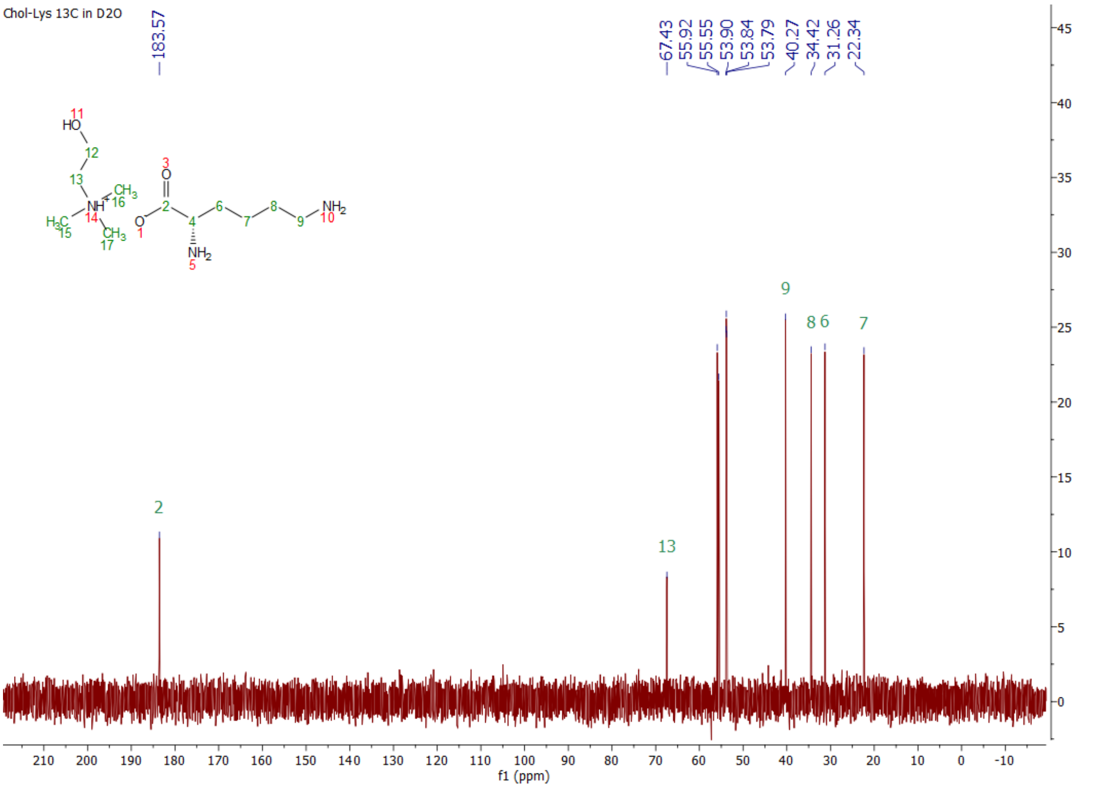

Figure S21. ^{13}C NMR of [Chol][Lys] in D_2O

[DBUH][OAc]

^{1}H NMR (300 MHz, D_2O) δ 3.46 (dq, *J* = 15.0, 7.1 Hz, 4H), 3.25 (t, *J* = 5.8 Hz, 2H), 2.62 – 2.47 (m, 2H), 1.93 (h, *J* = 5.9 Hz, 2H), 1.83 (s, 3H), 1.73 – 1.50 (m, 7H). ^{13}C NMR (76 MHz, D_2O) δ 180.83, 165.86, 54.06, 49.82, 48.15, 37.90, 35.99, 32.67, 29.03, 28.41, 25.84, 24.96, 23.68, 23.29, 22.60, 18.88.

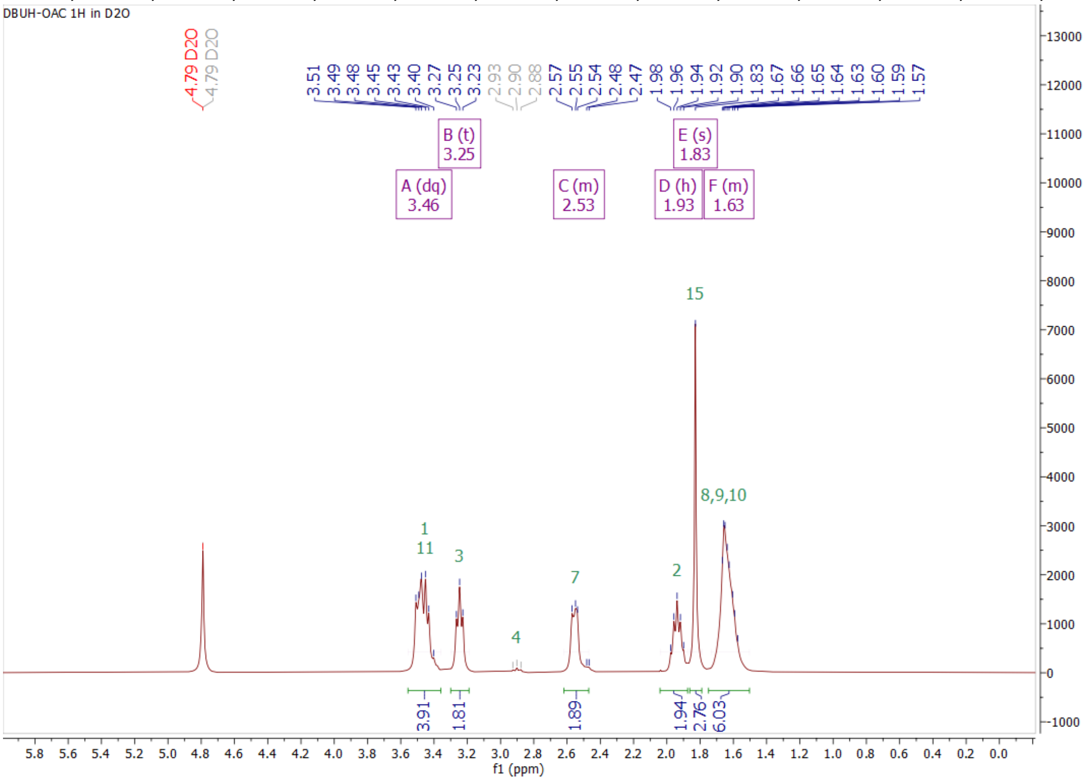

Figure S22. ^{1}H NMR of [DBUH][OAc] in D_2O

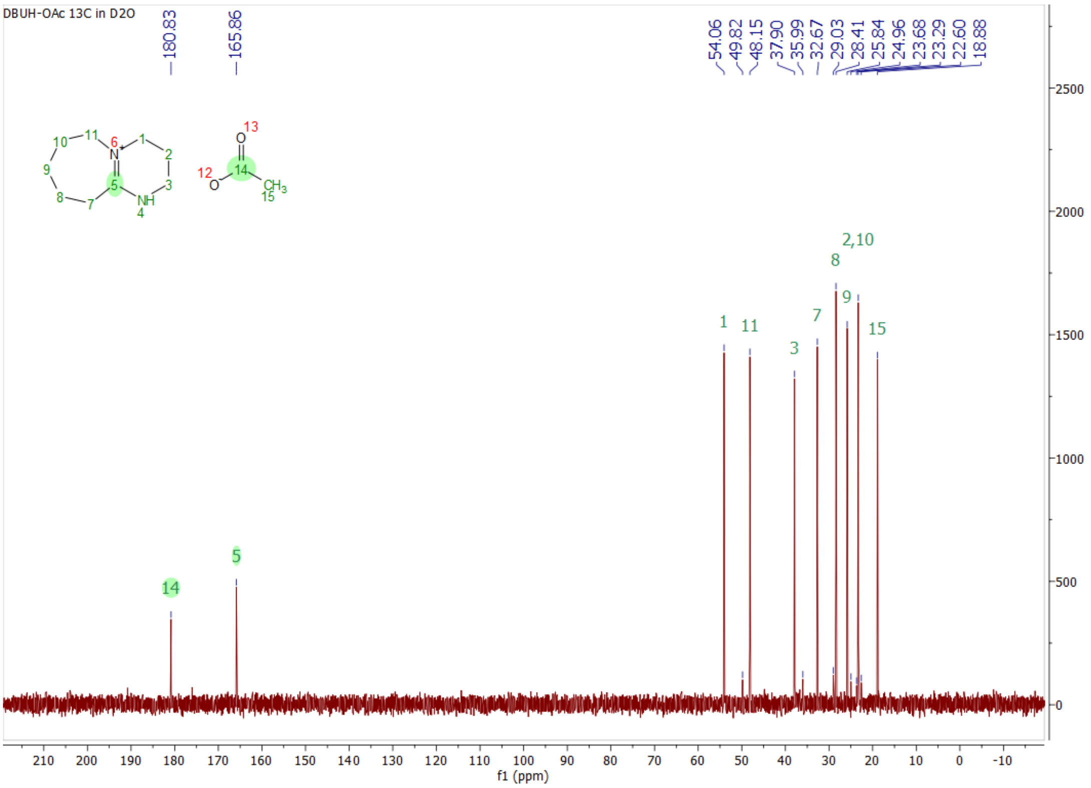

Figure S23. ^{13}C NMR of [DBUH][OAc] in D_2O

[EMIM][ACR]

^{1}H NMR (300 MHz, D_2O) δ 8.63 (s, 1H), 7.36 (d, *J* = 20.1 Hz, 2H), 5.92 (qd, *J* = 17.3, 5.8 Hz, 2H), 5.61 – 5.37 (m, 1H), 4.12 (q, *J* = 7.2 Hz, 2H), 3.79 (s, 3H), 1.38 (q, *J* = 8.9 Hz, 3H). ^{13}C NMR (76 MHz, D_2O) δ 174.74, 135.42, 134.02, 126.07, 123.41, 123.16, 121.82, 44.69, 35.56, 14.46.

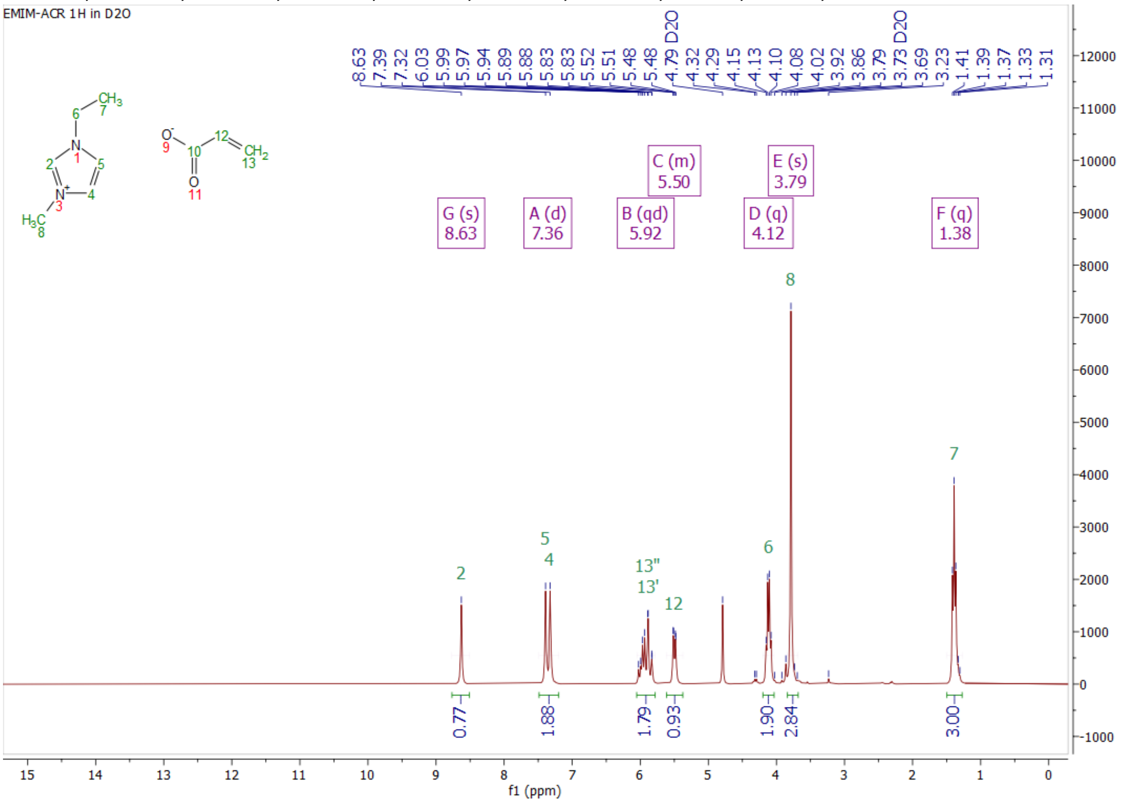

Figure S24. ^{1}H NMR of [EMIM][ACR] in D_2O

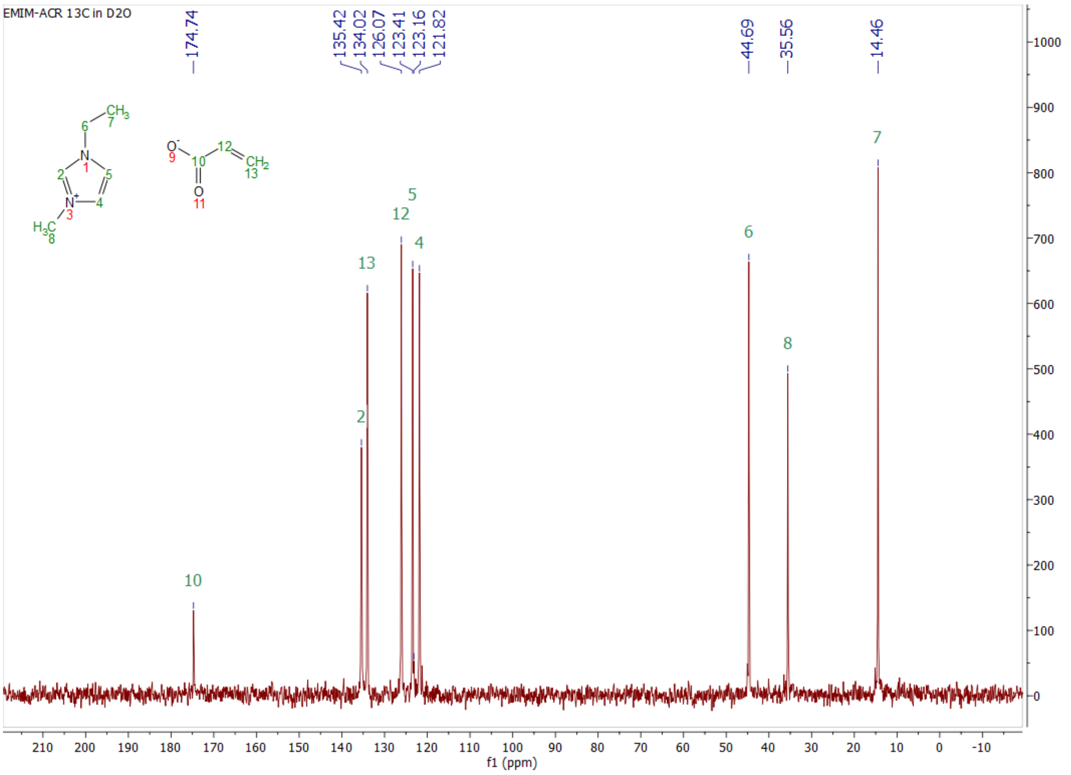

Figure S25. ^{13}C NMR of [EMIM][ACR] in D_2O

[EMIM][DCA]

^{1}H NMR (400 MHz, D_2O) δ 9.07 (s, 1H), 7.72 (t, *J* = 4.5 Hz, 1H), 7.67 – 7.60 (m, 1H), 4.18 (q, *J* = 7.2 Hz, 5H), 3.84 (s, 5H), 1.41 (t, *J* = 7.3 Hz, 7H). ^{13}C NMR (101 MHz, D_2O) δ 136.30, 123.60, 121.97, 119.17, 44.33, 35.78, 15.05.

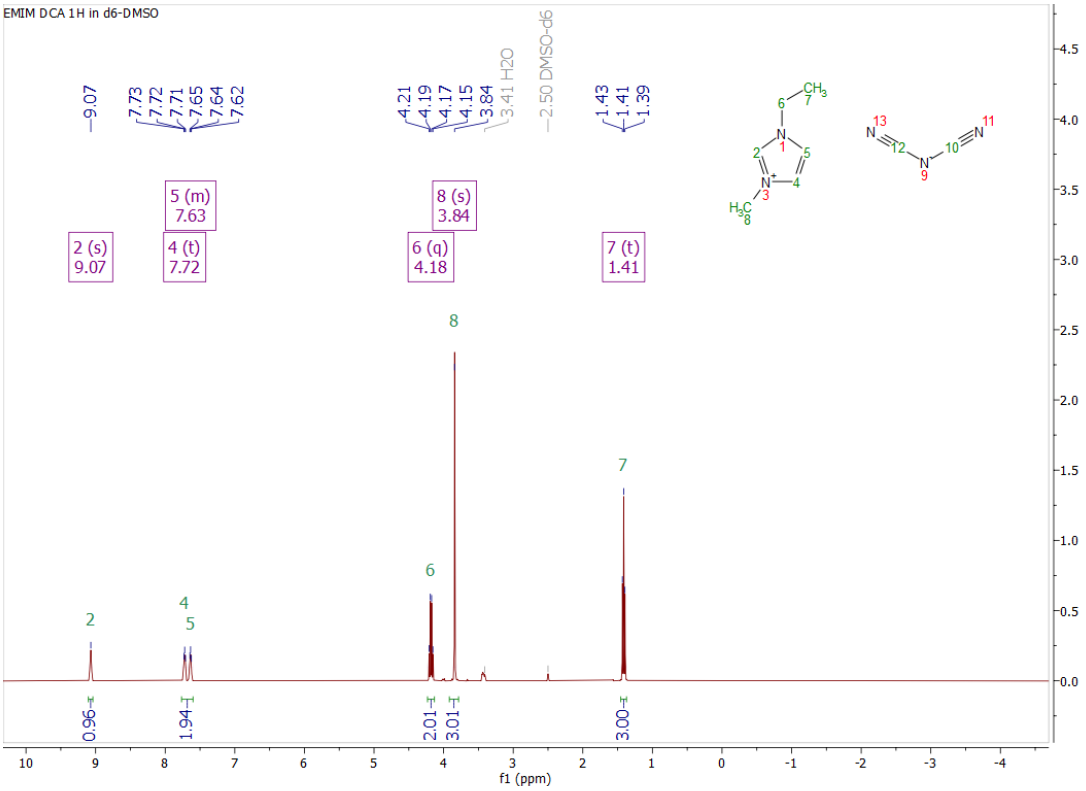

Figure S26. ^{1}H NMR of [EMIM][DCA] in d_6-DMSO.

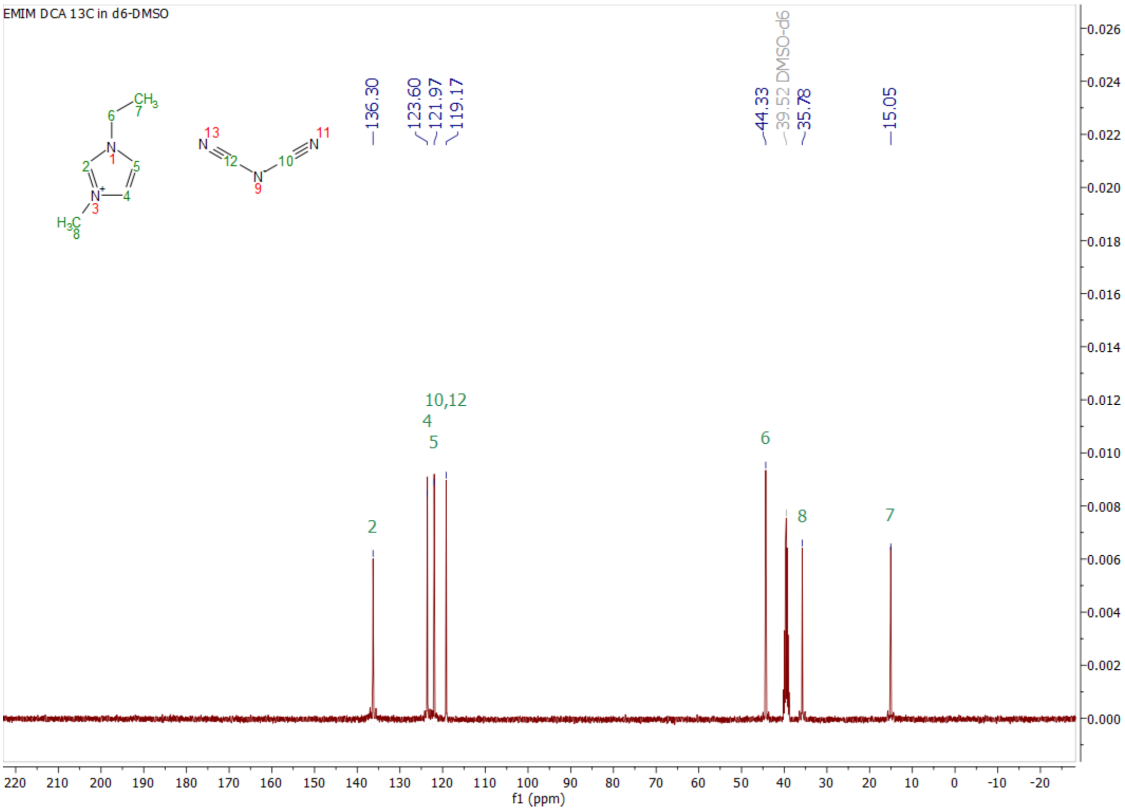

Figure S27. ^{13}C NMR of [EMIM][DCA] in D_2O.

[EMIM][DEP]

^{1}H NMR (300 MHz, D_2O) δ 8.69 (s, 1H), 7.42 (dt, *J* = 20.2, 2.1 Hz, 2H), 4.19 (q, *J* = 7.4 Hz, 2H), 3.94 – 3.78 (m, 8H), 1.45 (t, *J* = 7.4 Hz, 3H), 1.27 – 1.10 (m, 7H). ^{13}C NMR (76 MHz, D_2O) δ 135.51, 123.43, 121.85, 62.12, 62.05, 44.72, 35.56, 15.65, 15.56, 14.47.

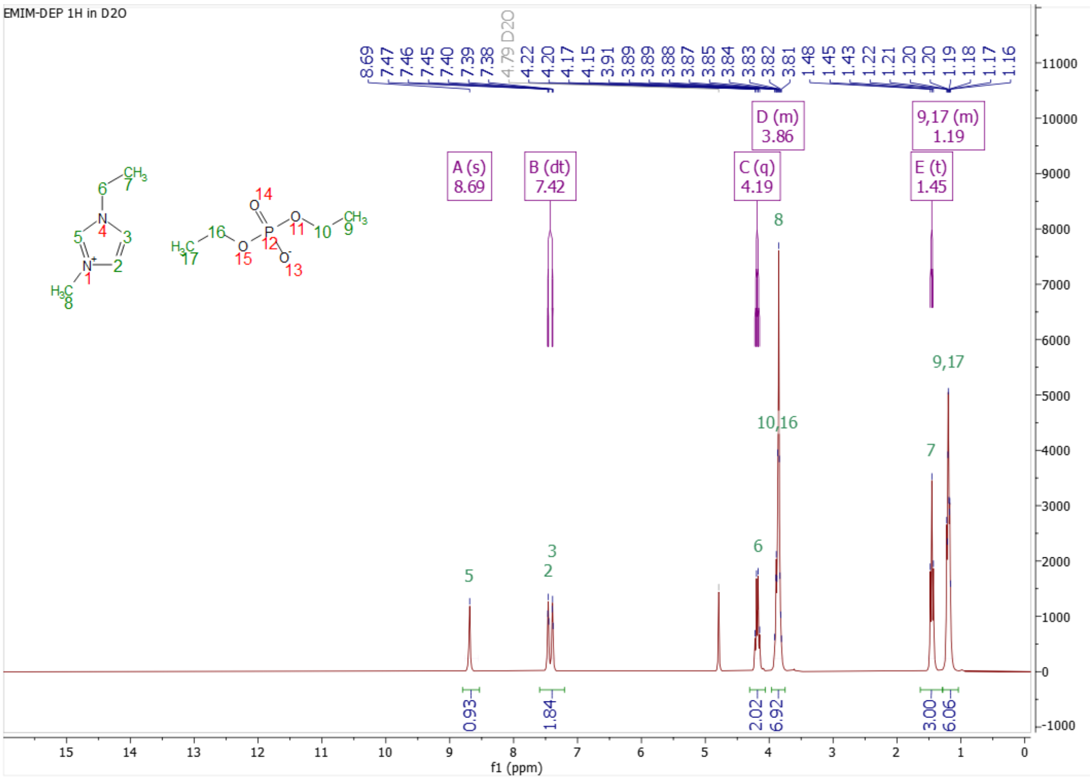

Figure S28. ^{1}H NMR of [EMIM][DEP] in D_2O

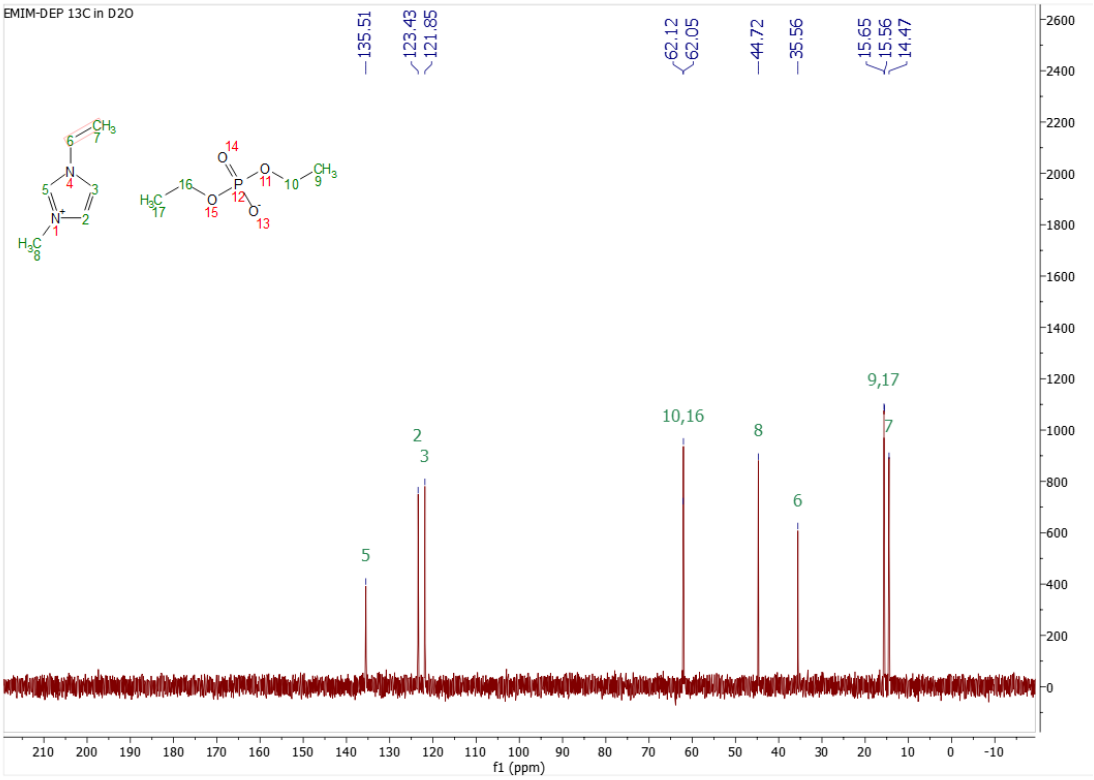

Figure S29. ^{13}C NMR of [EMIM][DEP] in D_2O

[EMIM][FSI]

^{1}H NMR (300 MHz, DMSO) δ 9.05 (s, 1H), 7.64 (dt, *J* = 22.6, 1.8 Hz, 2H), 4.19 (q, *J* = 7.3 Hz, 2H), 3.85 (s, 3H), 1.43 (t, 3H).^{13}C NMR (76 MHz, DMSO) δ 136.10, 123.40, 121.76, 121.73, 44.17, 40.17, 39.89, 39.61, 39.34, 39.06, 38.78, 38.50, 35.50, 14.74, 14.70, 14.66.

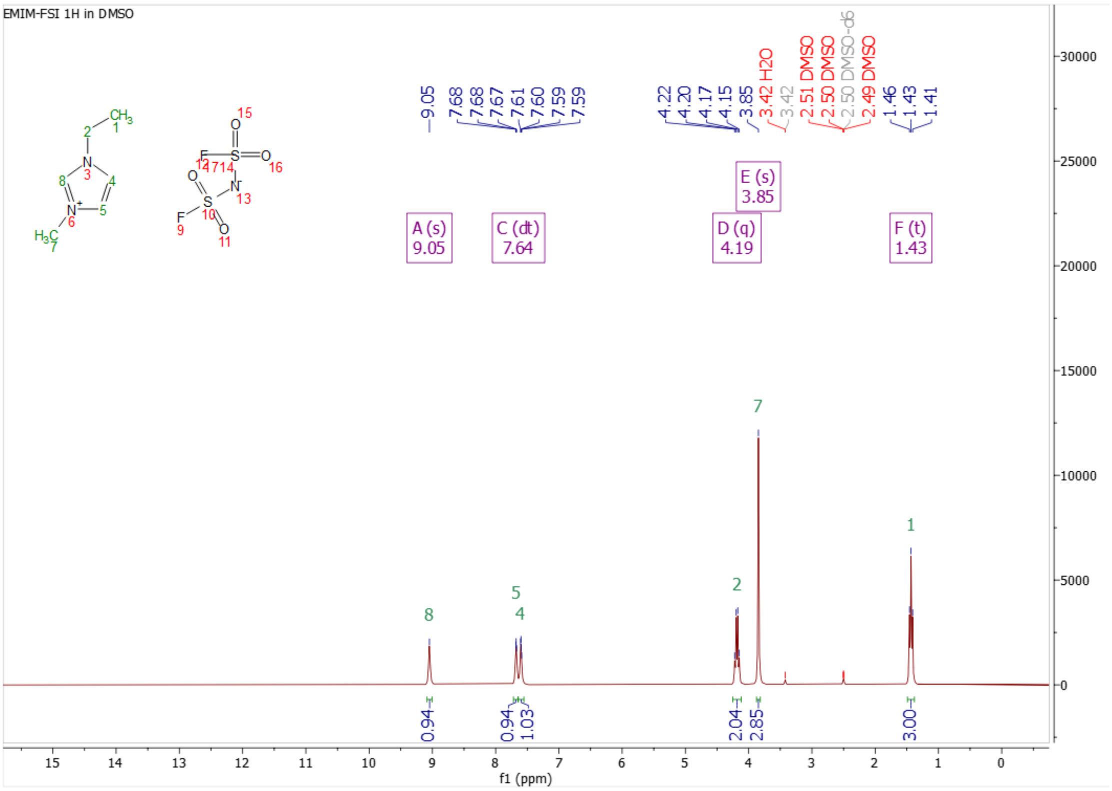

Figure S30. ^{1}H NMR of [EMIM][FSI] in d_6-DMSO

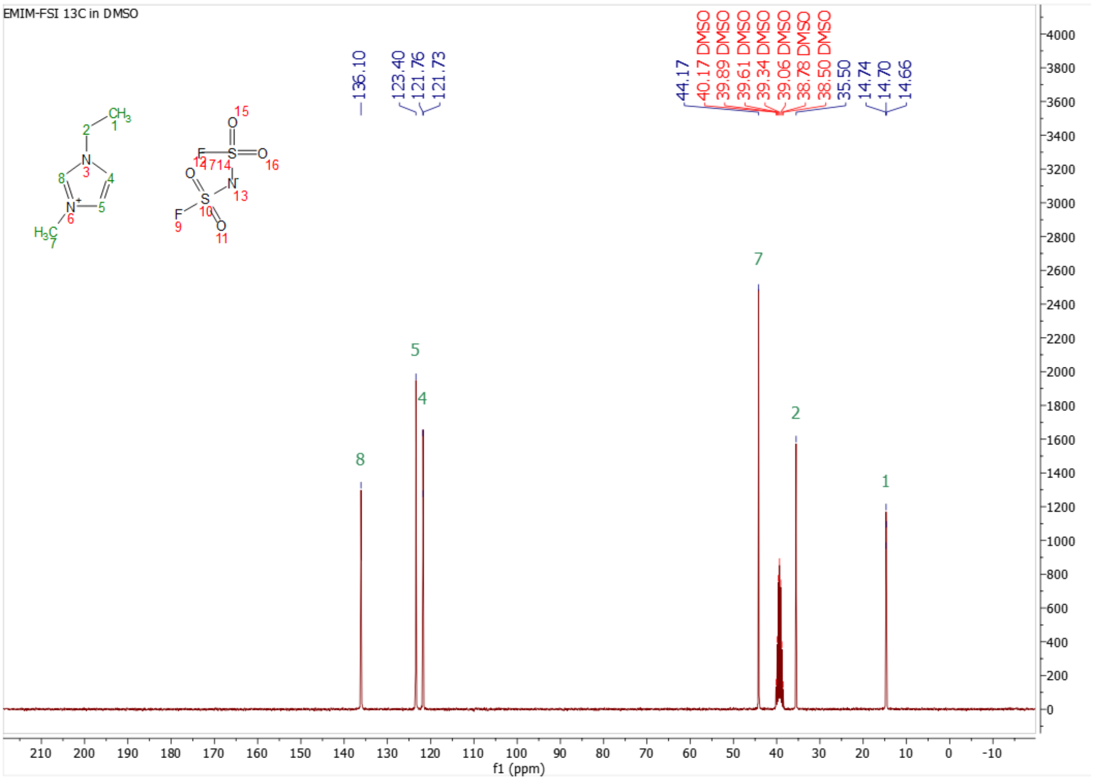

Figure S31. ^{13}C NMR of [EMIM][FSI] in d_6-DMSO

[EMIM][MeSO3]

^{1}H NMR (300 MHz, D_2O) δ 8.74 (s, 1H), 7.49 (dt, 2H), 4.25 (q, J = 7.4 Hz, 2H), 3.92 (d, J = 3.3 Hz, 3H), 2.79 (s, 3H), 1.58 – 1.46 (m, 3H). ^{13}C NMR (76 MHz, D_2O) δ 135.59, 123.49, 121.90, 44.79, 38.58, 35.65, 14.54.

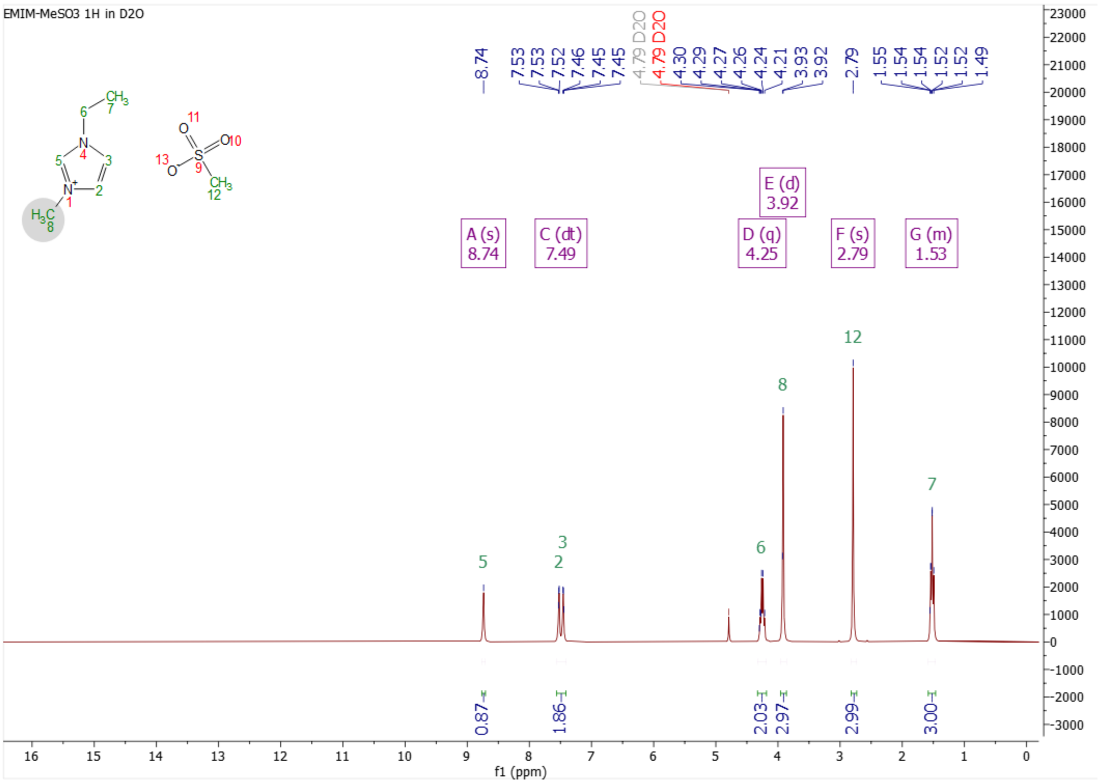

Figure S32. ^{1}H NMR of [EMIM][MeSO3] in D_2O

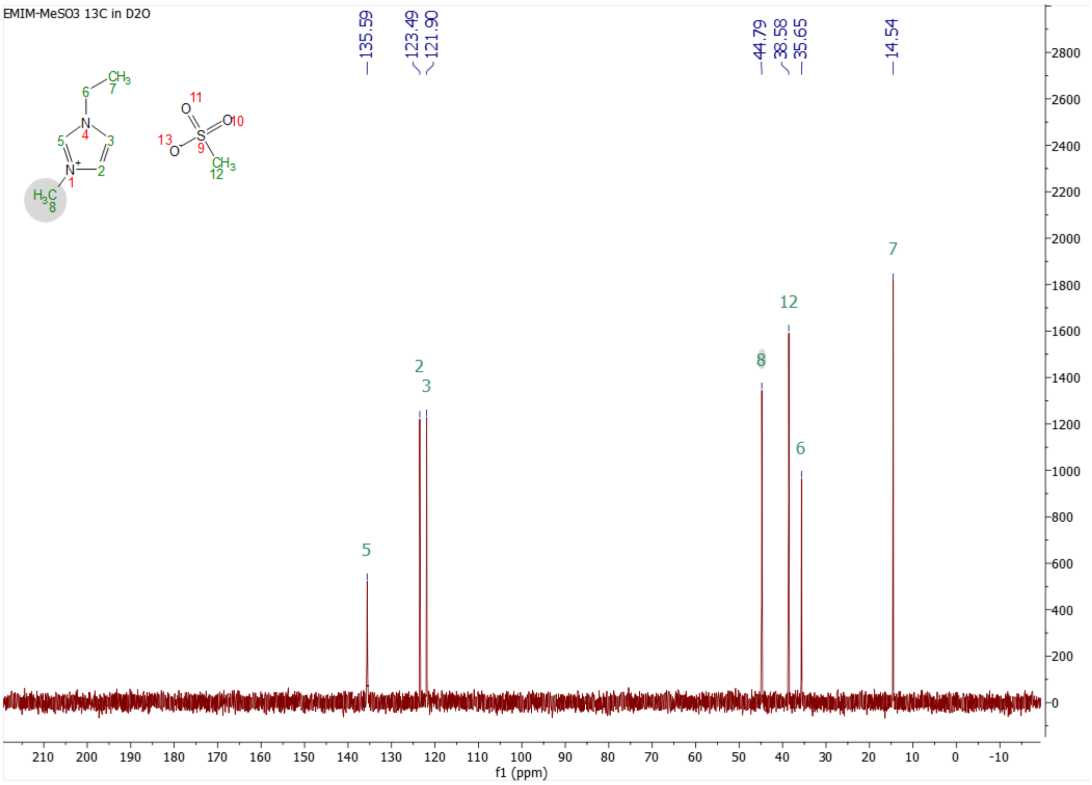

Figure S33. ^{13}C NMR of [EMIM][MeSO3] in D_2O

[EMIM][OAc]

^{1}H NMR (300 MHz, DMSO) δ 10.39 (s, 1H), 8.01 (dt, *J* = 35.6, 1.8 Hz, 2H), 4.23 (q, *J* = 7.3 Hz, 2H), 3.89 (s, 3H), 1.59 (s, 3H), 1.36 (t, *J* = 7.3 Hz, 3H). ^{13}C NMR (76 MHz, DMSO) δ 173.59, 173.57, 137.98, 123.63, 122.15, 43.86, 40.35, 40.07, 39.80, 39.52, 39.24, 38.97, 38.69, 35.35, 26.00, 15.26.

^{1}H NMR (400 MHz, D_2O) δ 8.64 (d, J = 1.8 Hz, 0H), 7.33 (dt, J = 28.2, 1.9 Hz, 1H), 4.03 (q, J = 7.4 Hz, 1H), 3.71 (s, 1H), 1.61 (s, 1H), 1.28 (t, J = 7.4 Hz, 1H). ^{13}C NMR (101 MHz, D_2O) δ 179.56, 135.60, 135.56, 123.44, 121.82, 44.63, 35.59, 23.58, 14.58.

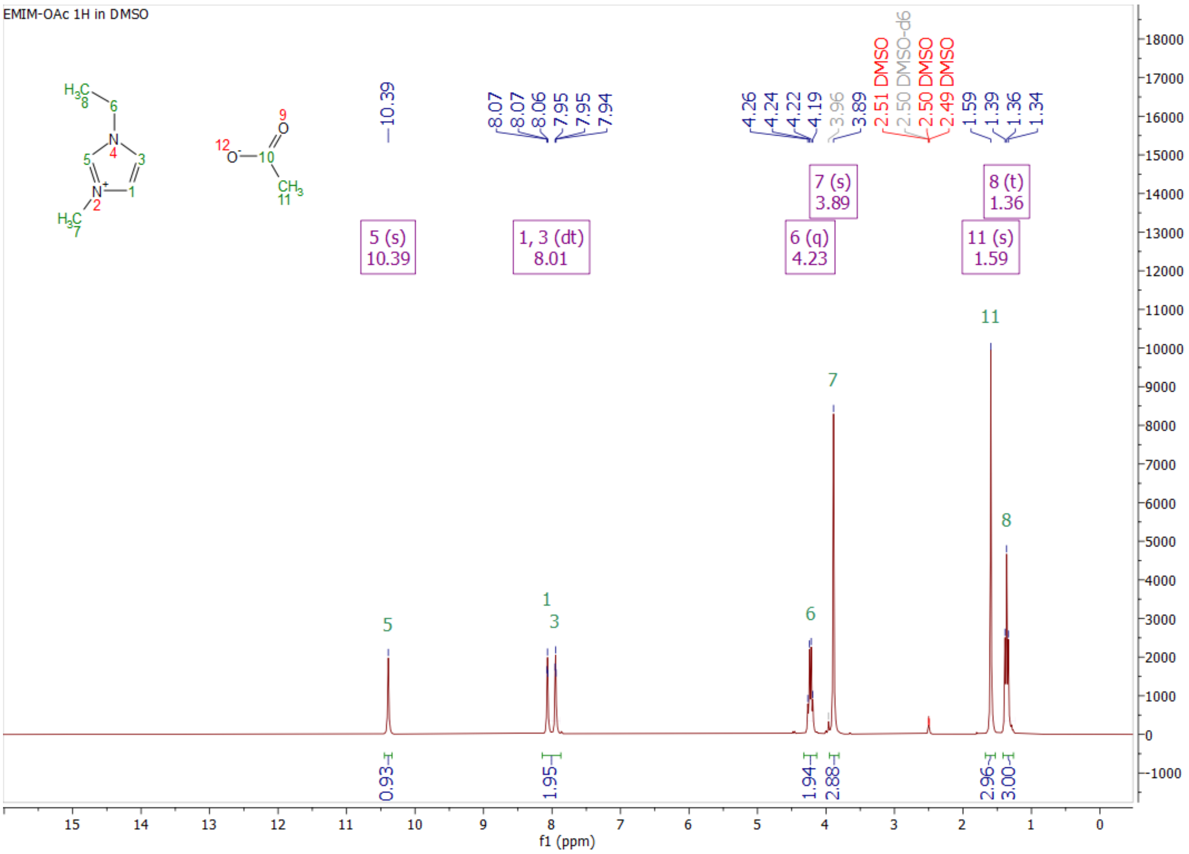

Figure S34. ^{1}H NMR of [EMIM][OAc] in d_6-DMSO

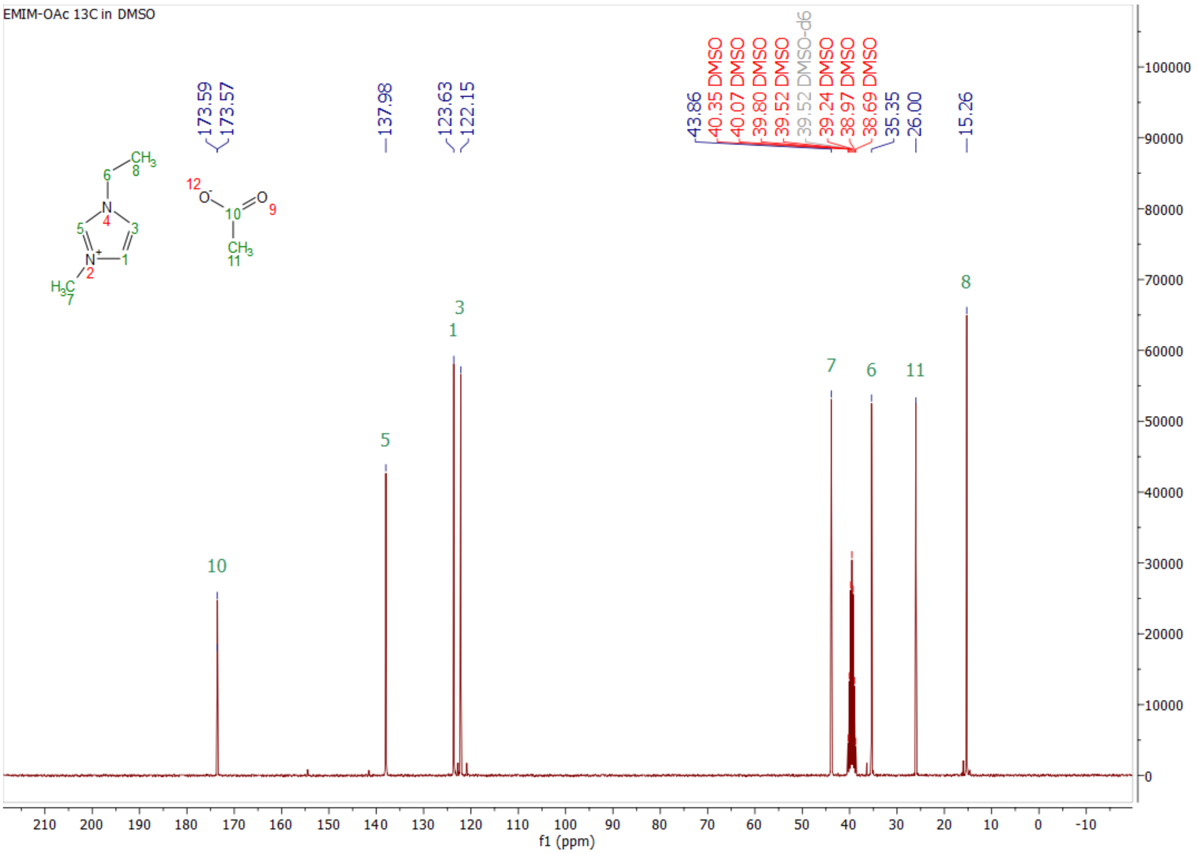

Figure S35. ^{13}C NMR of [EMIM][OAc] in d_6-DMSO

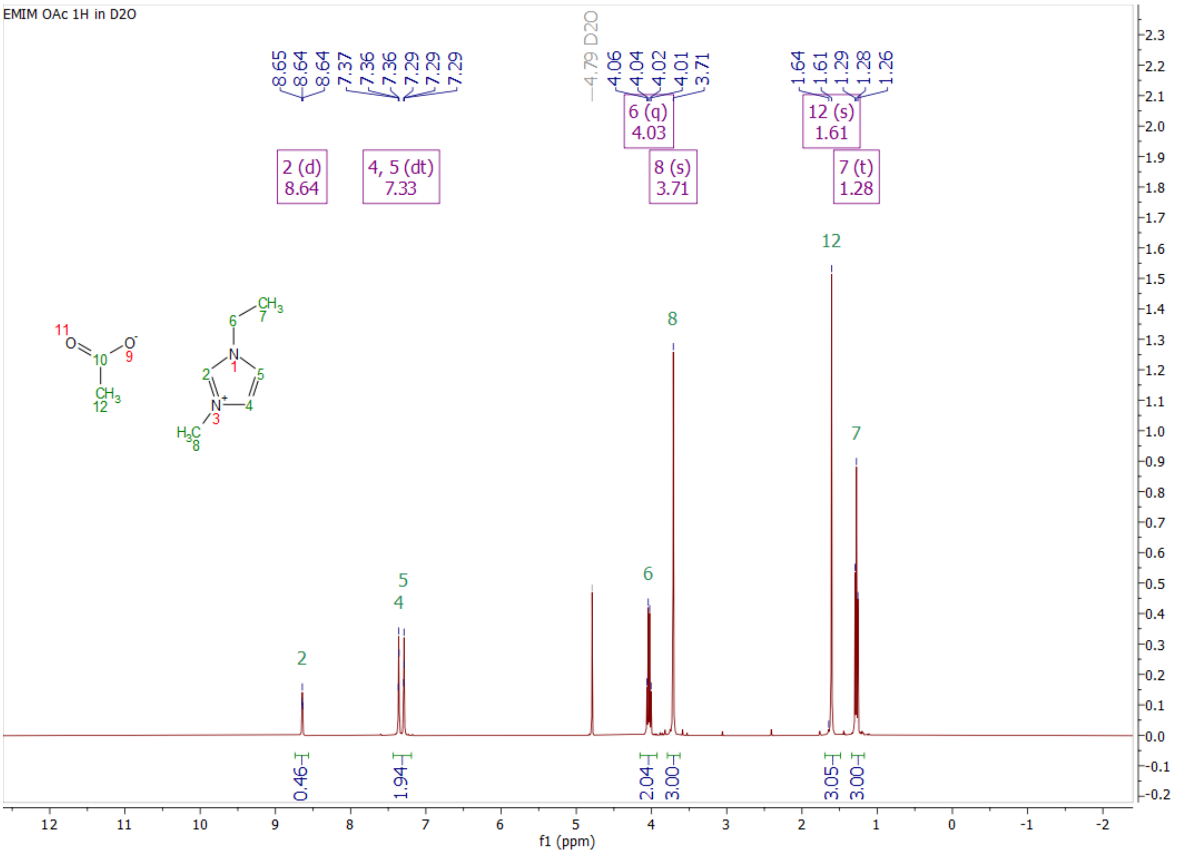

Figure S36. ^{1}H NMR of [EMIM][OAc] in D_2O

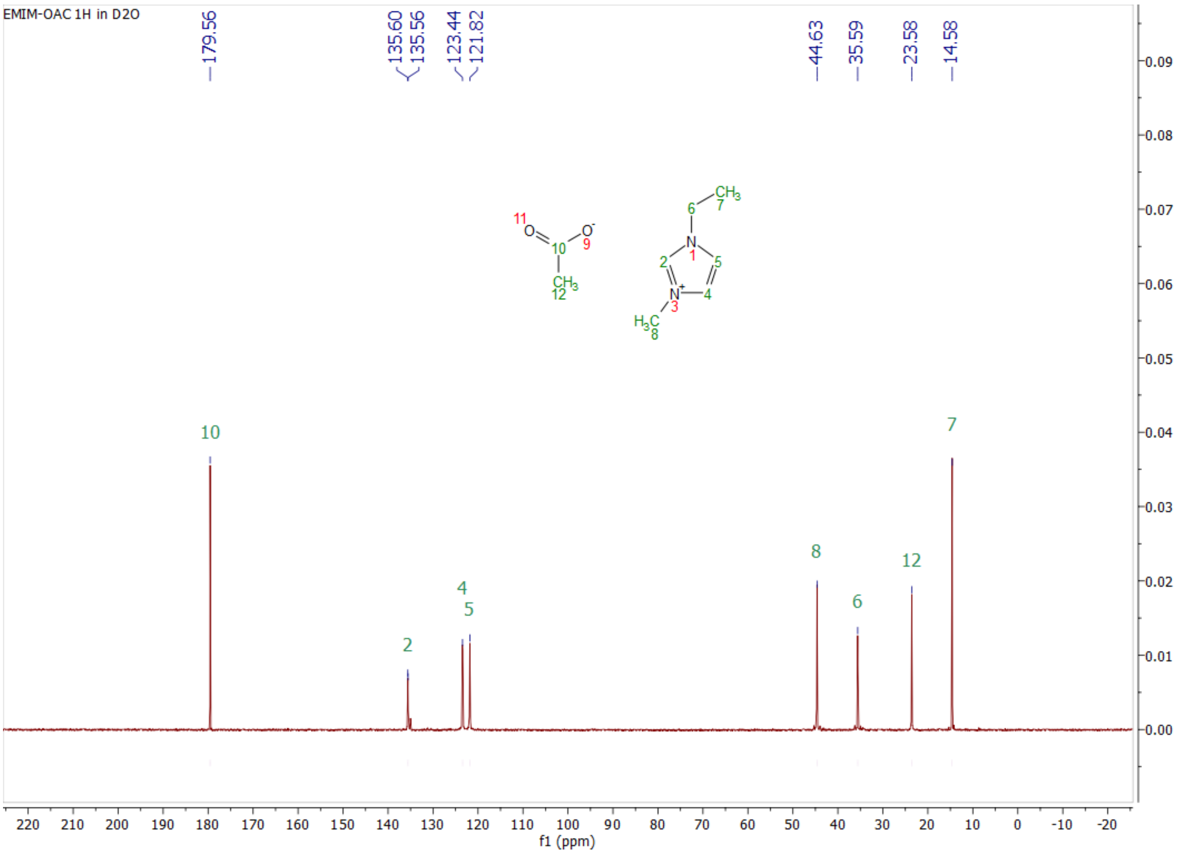

Figure S37. ^{13}C NMR of [EMIM][OAc] in D_2O

[EMIM][OOc]

^{1}H NMR (300 MHz, D_2O) δ 8.72 (s, 1H), 7.44 (dd, J = 19.2, 2.0 Hz, 2H), 4.19 (q, J = 7.4 Hz, 2H), 3.86 (s, 3H), 2.10 (t, J = 7.5 Hz, 2H), 1.55 – 1.37 (m, 5H), 1.30 – 1.11 (m, 8H), 0.80 (t, J = 6.4 Hz, 3H). ^{13}C NMR (76 MHz, D_2O) δ 183.40, 123.46, 121.86, 44.74, 37.66, 35.57, 31.23, 28.93, 28.48, 26.00, 22.12, 14.51, 13.51.

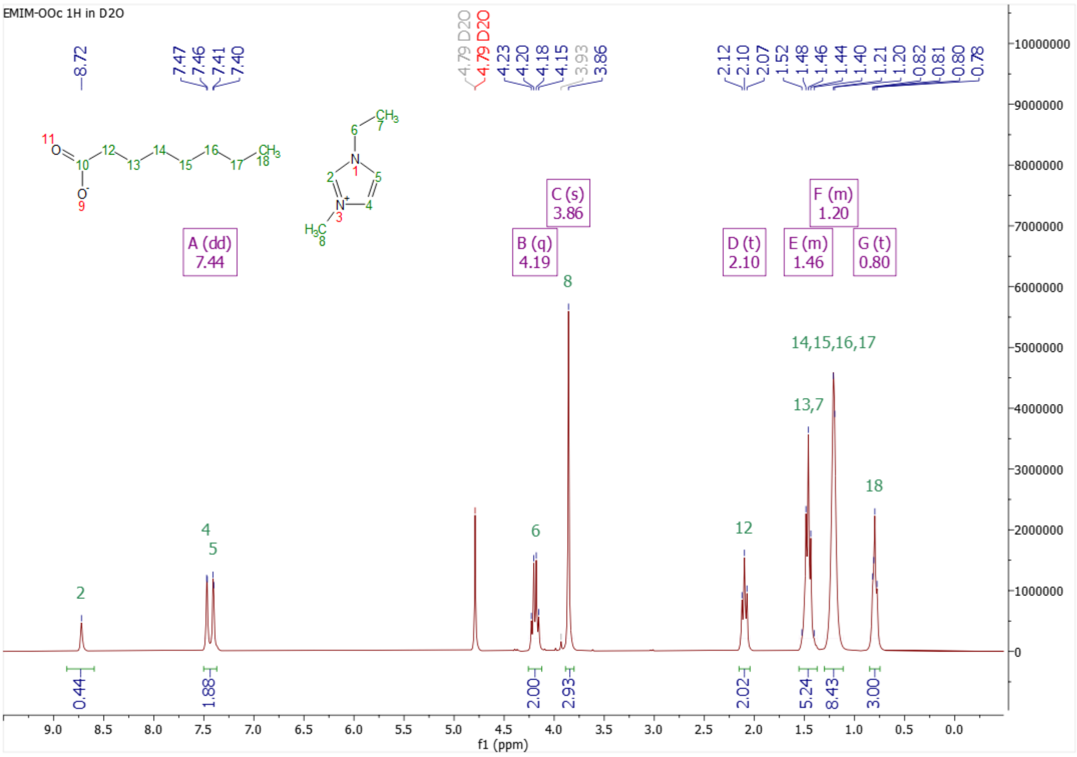

Figure S38. ^{1}H NMR of [EMIM][OOc] in D_2O

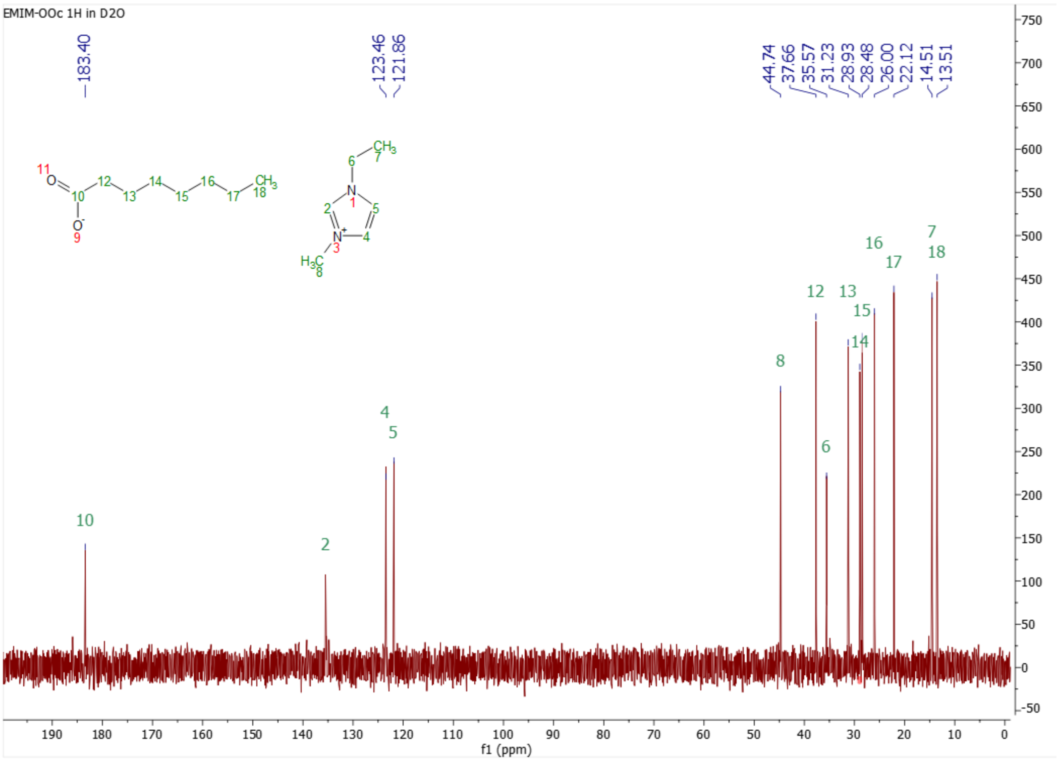

Figure S39. ^{13}C NMR of [EMIM][OOc] in D_2O

[EMIM][OPr]

^{1}H NMR (300 MHz, D_2O) δ 8.65 (s, 1H), 7.38 (dt, *J* = 20.6, 2.1 Hz, 2H), 4.14 (q, *J* = 7.4 Hz, 2H), 3.80 (s, 3H), 2.05 (q, *J* = 7.6 Hz, 2H), 1.40 (t, *J* = 7.3 Hz, 3H), 0.93 (t, *J* = 7.6 Hz, 3H). ^{13}C NMR (76 MHz, D_2O) δ 183.98, 135.49, 123.40, 121.82, 44.70, 35.54, 30.50, 14.45, 10.09.

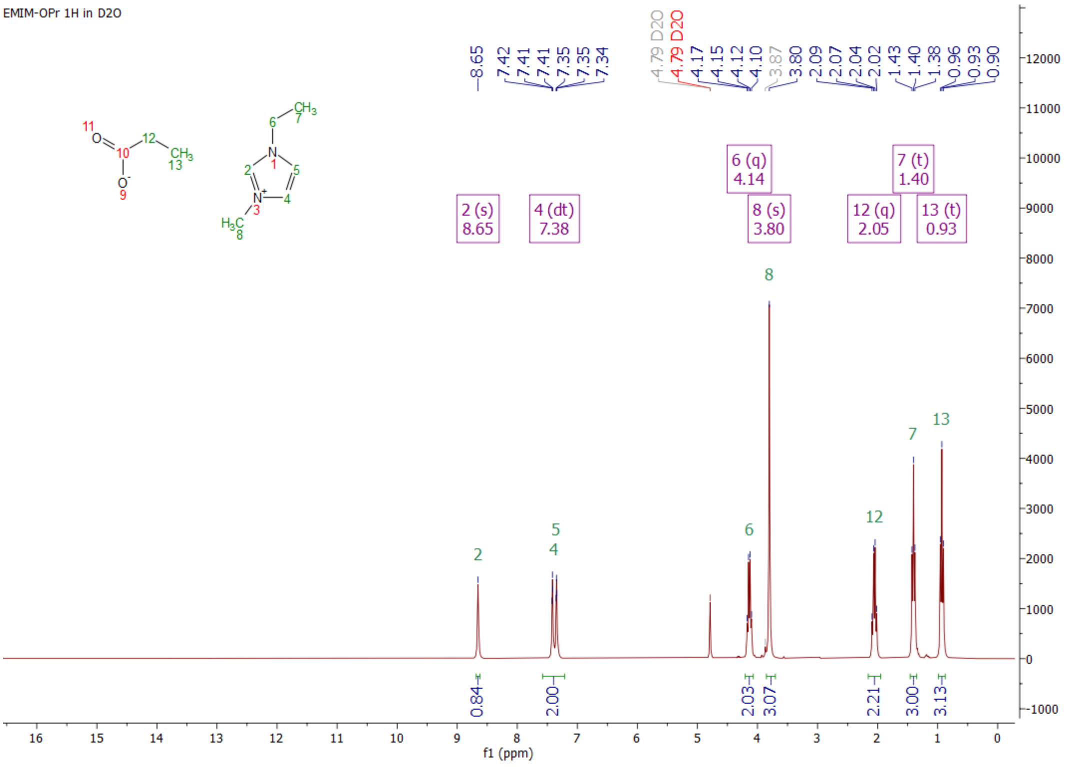

Figure S40. ^{1}H NMR of [EMIM][OPr] in D_2O

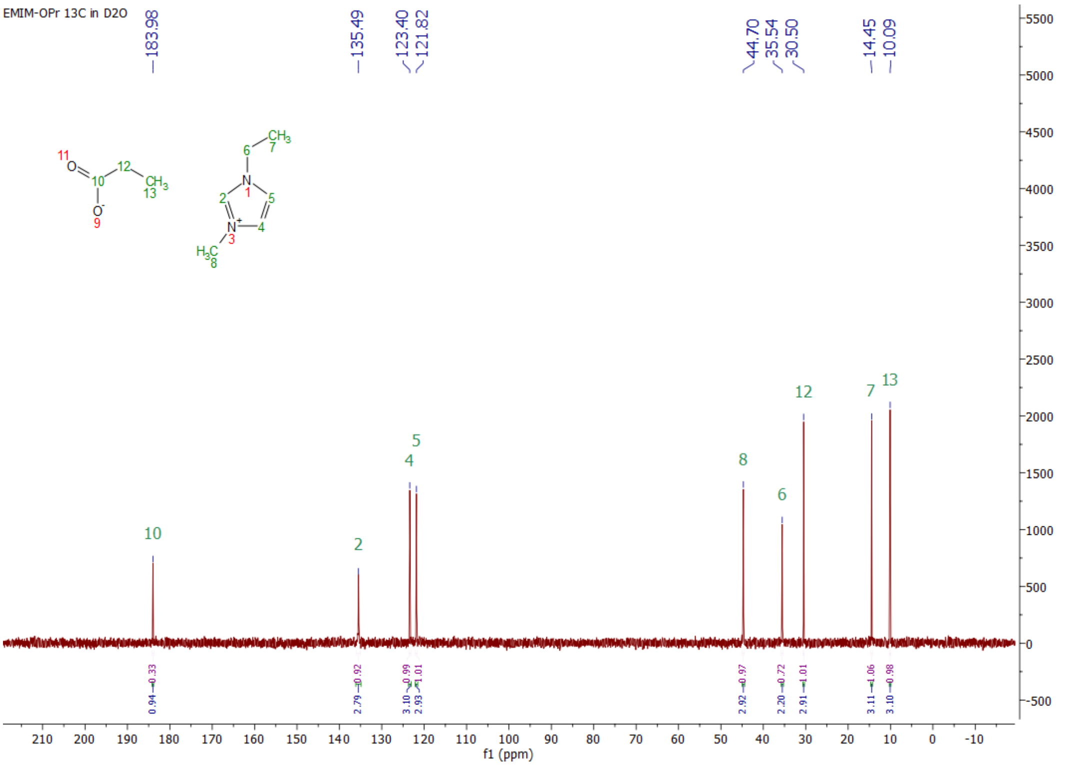

Figure 41. ^{13}C NMR of [EMIM][OPr] in D_2O

[EMIM][OTf]

^{1}H NMR (300 MHz, DMSO) δ 9.05 (s, 1H), 7.69 (dt, J = 25.9, 1.8 Hz, 2H), 4.19 (q, J = 7.3 Hz, 2H), 3.85 (s, 3H), 1.41 (t, J = 7.3 Hz, 3H). ^{13}C NMR (76 MHz, DMSO) δ 136.38, 127.17, 123.59, 123.01, 122.91, 121.99, 118.65, 114.39, 44.32, 42.96, 40.35, 40.07, 39.79, 39.52, 39.24, 38.96, 38.68, 35.68, 35.30, 14.96, 14.94, 14.91.

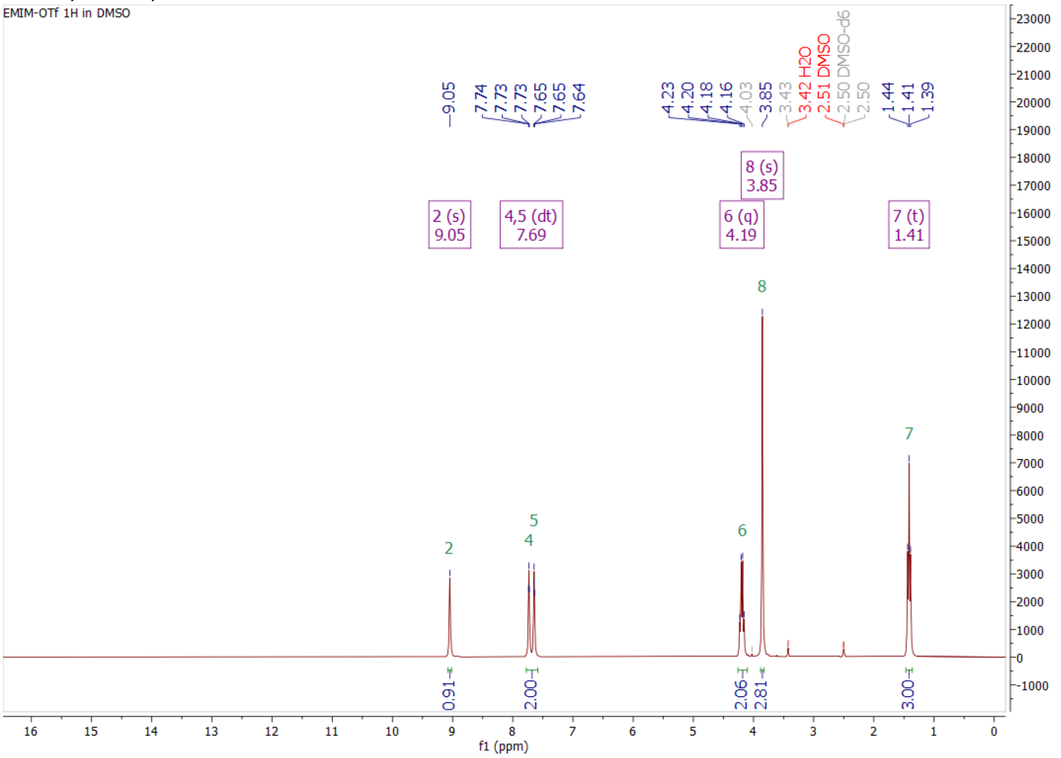

Figure S42. ^{1}H NMR of [EMIM][OTf] in d_6-DMSO

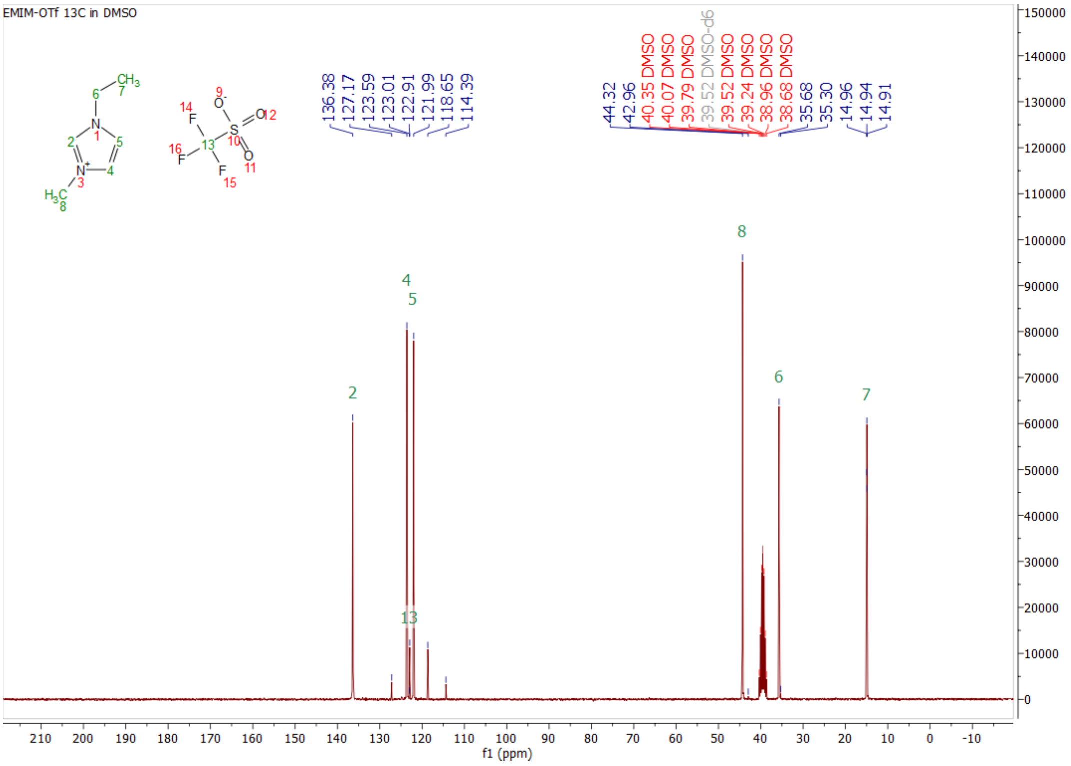

Figure S43. ^{13}C NMR of [EMIM][OTf] in d_6-DMSO

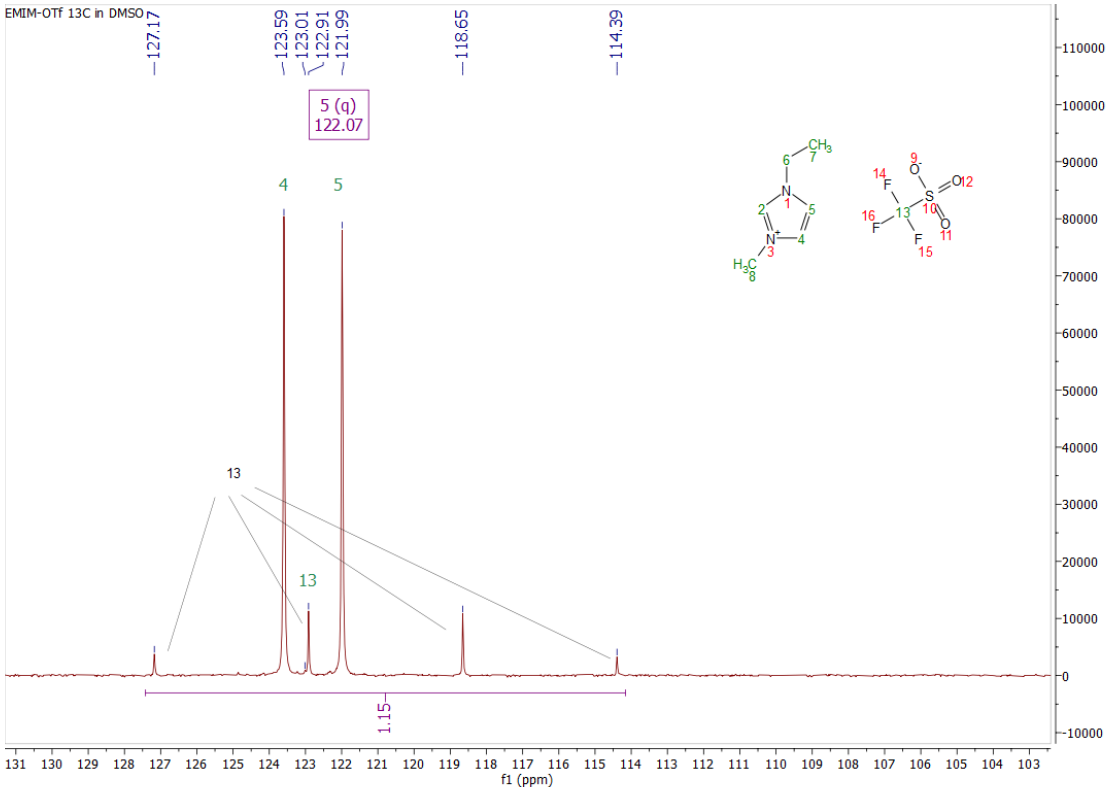

Figure S44. Detailed view of the ^{13}C NMR of [EMIM][OTf] in d_6-DMSO between 131 and 103 ppm. The peaks of carbon 13 are couplings with the fluorine atoms.

[EMIM][SCN]

^{1}H NMR (300 MHz, D_2O) δ 8.82 (s, 1H), 7.57 (dt, J = 20.2, 2.0 Hz, 2H), 4.34 (q, J = 7.4 Hz, 2H), 4.01 (s, 3H), 1.60 (t, J = 7.4 Hz, 3H). ^{13}C NMR (76 MHz, D_2O) δ 135.71, 132.96, 123.61, 122.01, 45.02, 35.86, 14.72.

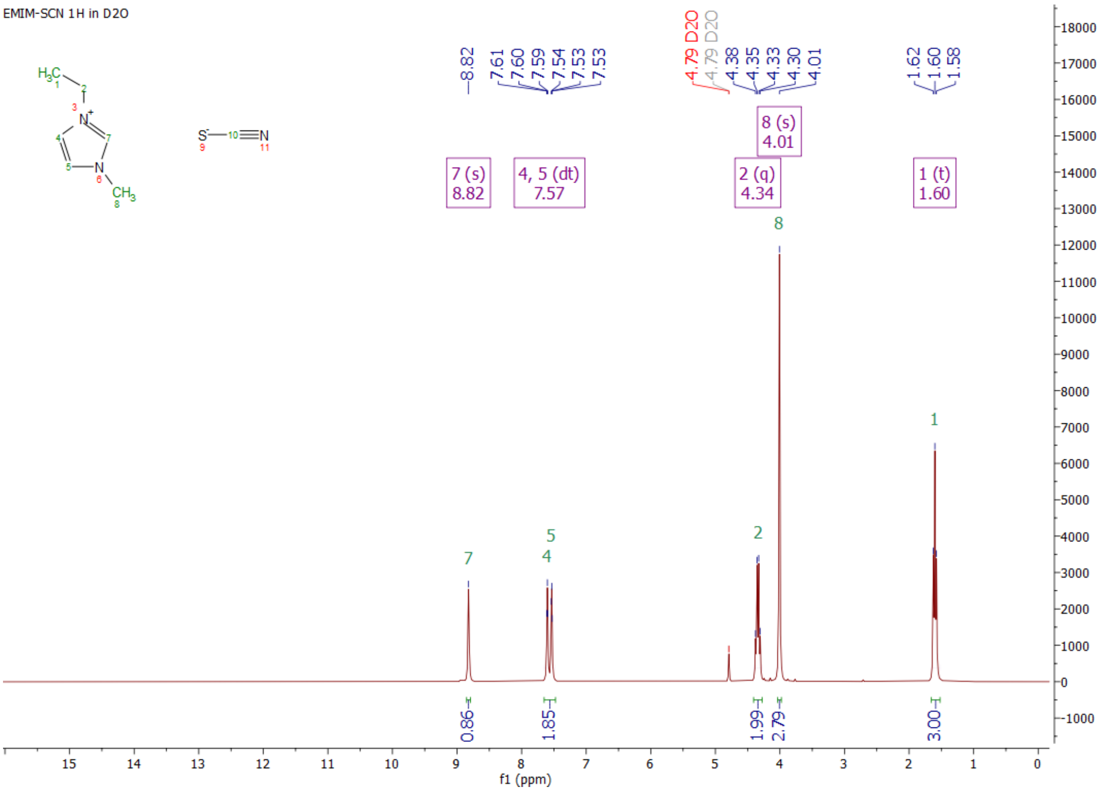

Figure S45. ^{1}H NMR of [EMIM][SCN] in D_2O

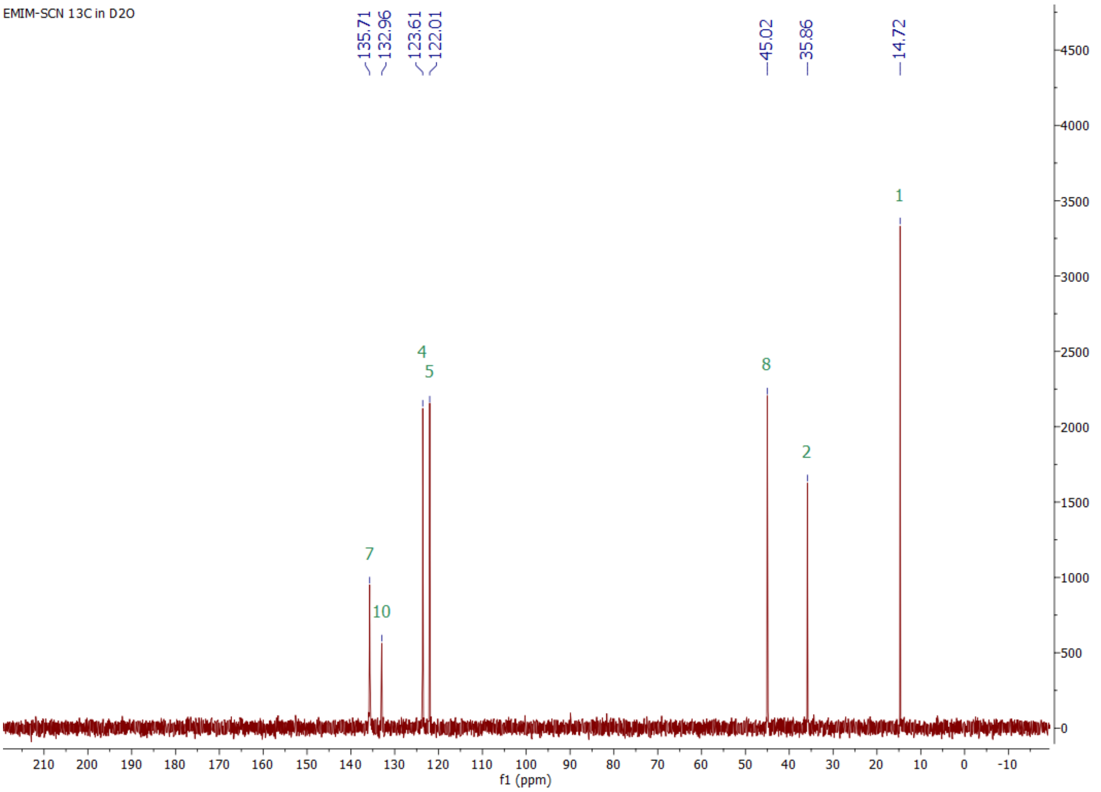

Figure S46. ^{13}C NMR of [EMIM][SCN] in D_2O

[EMIM][TFSI]

^{1}H NMR (300 MHz, DMSO) δ 9.08 (s, 1H), 7.67 (dt, *J* = 24.4, 1.9 Hz, 2H), 4.20 (q, *J* = 7.3 Hz, 2H), 3.85 (s, 3H), 1.43 (t, *J* = 7.3 Hz, 3H). ^{13}C NMR (76 MHz, DMSO) δ 136.33, 123.59, 121.96, 126.02 - 113.25 (q), 44.30, 40.35, 40.07, 39.80, 39.52, 39.24, 38.96, 38.69, 35.63, 14.85, 14.82.

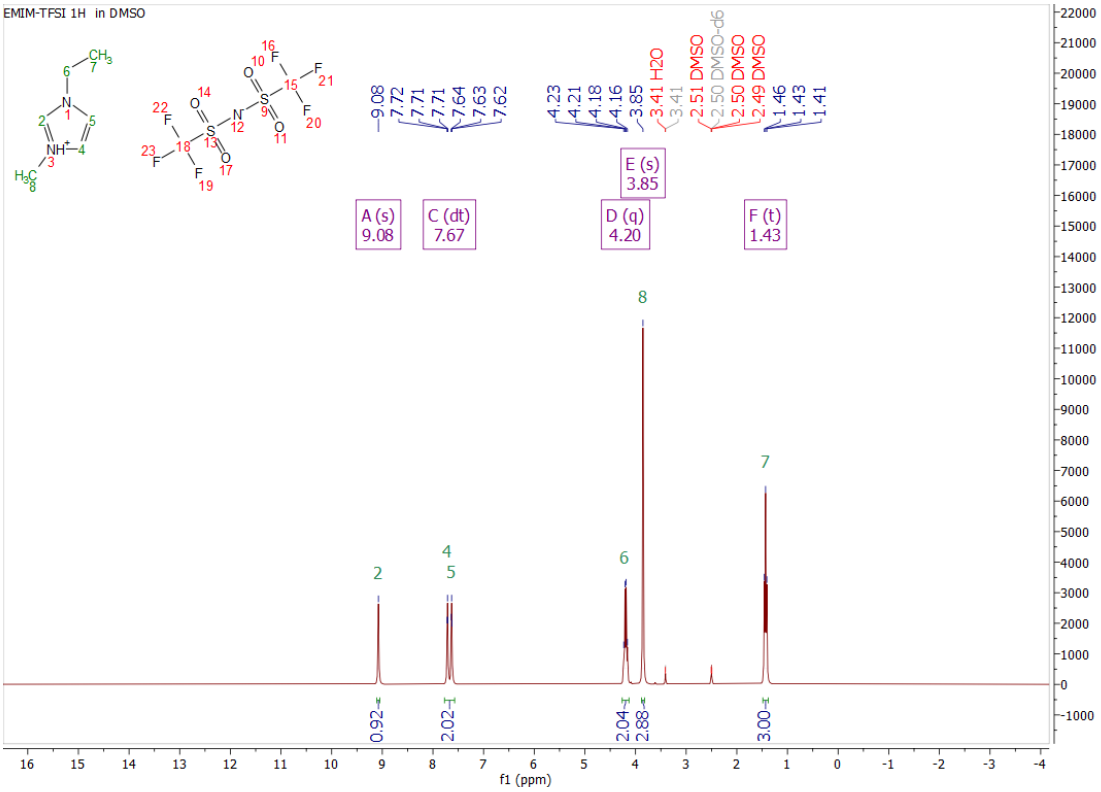

Figure S47. ^{1}H NMR of [EMIM][TFSI] in d_6-DMSO

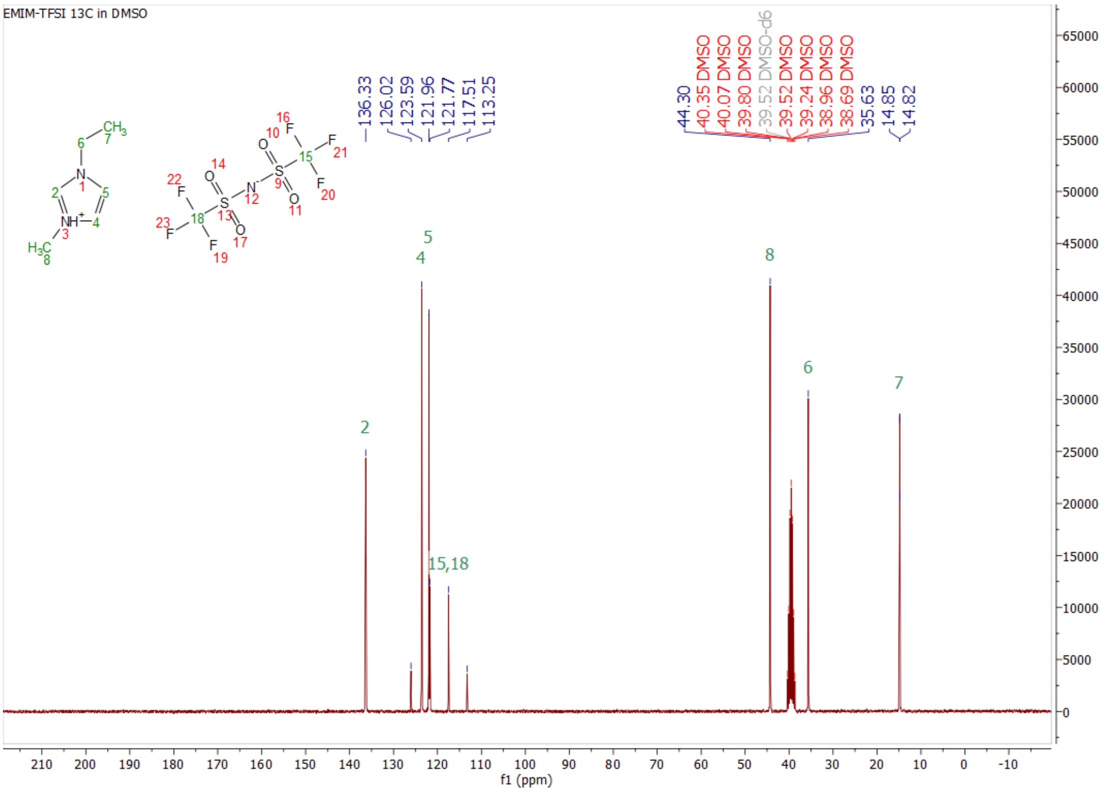

Figure S48. ^{13}C NMR of [EMIM][TFSI] in d_6-DMSO

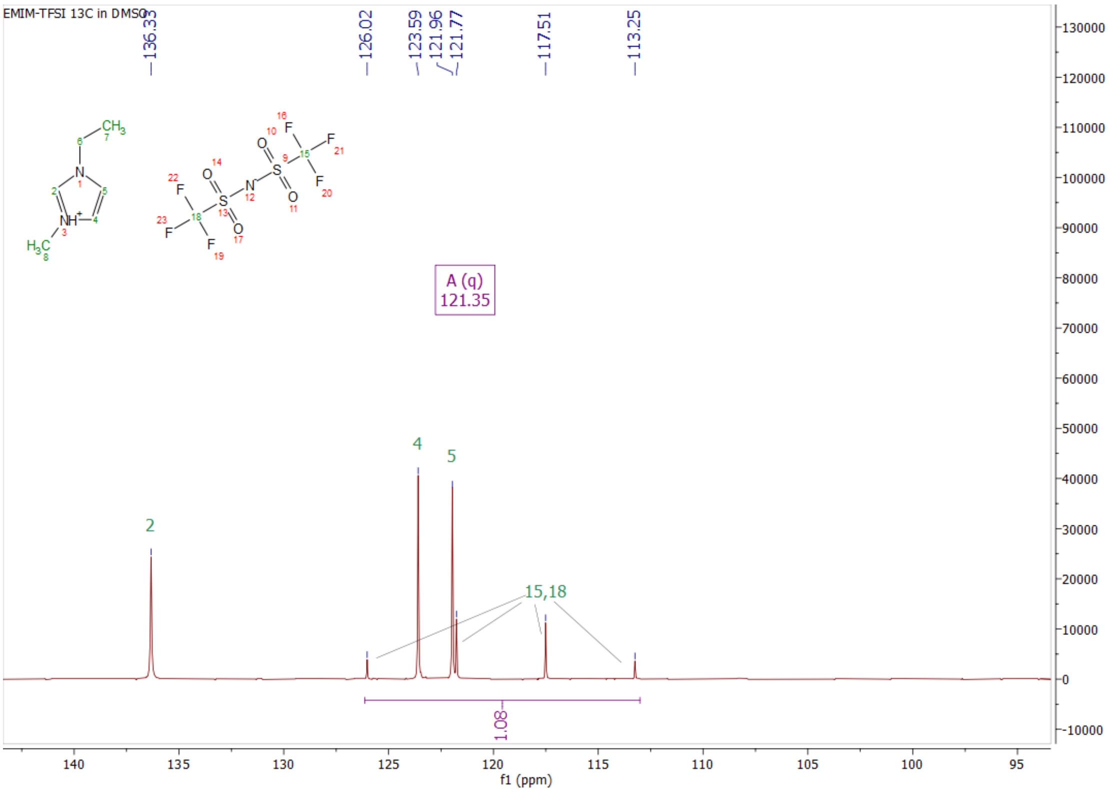

Figure S49. ^{13}C NMR of [EMIM][TFSI] in d_6-DMSO between 140 and 95 ppm. The peaks of carbon 15 and 18 are couplings with the fluorine atoms.

[HEXMIM][OAc]

^{1}H NMR (300 MHz, D_2O) δ 8.69 (s, 1H), 7.42 (dt, *J* = 13.3, 1.9 Hz, 2H), 6.15 – 5.89 (m, 2H), 5.58 (dd, *J* = 9.8, 2.3 Hz, 1H), 4.14 (t, *J* = 7.1 Hz, 2H), 3.85 (s, 3H), 1.80 (q, *J* = 7.1 Hz, 2H), 1.35 – 1.15 (m, 3H), 0.80 (q, *J* = 4.6 Hz, 3H). ^{13}C NMR (76 MHz, D_2O) δ 175.08, 135.74, 133.94, 126.23, 123.48, 122.19, 49.52, 35.60, 30.30, 29.13, 24.96, 21.75, 13.23.

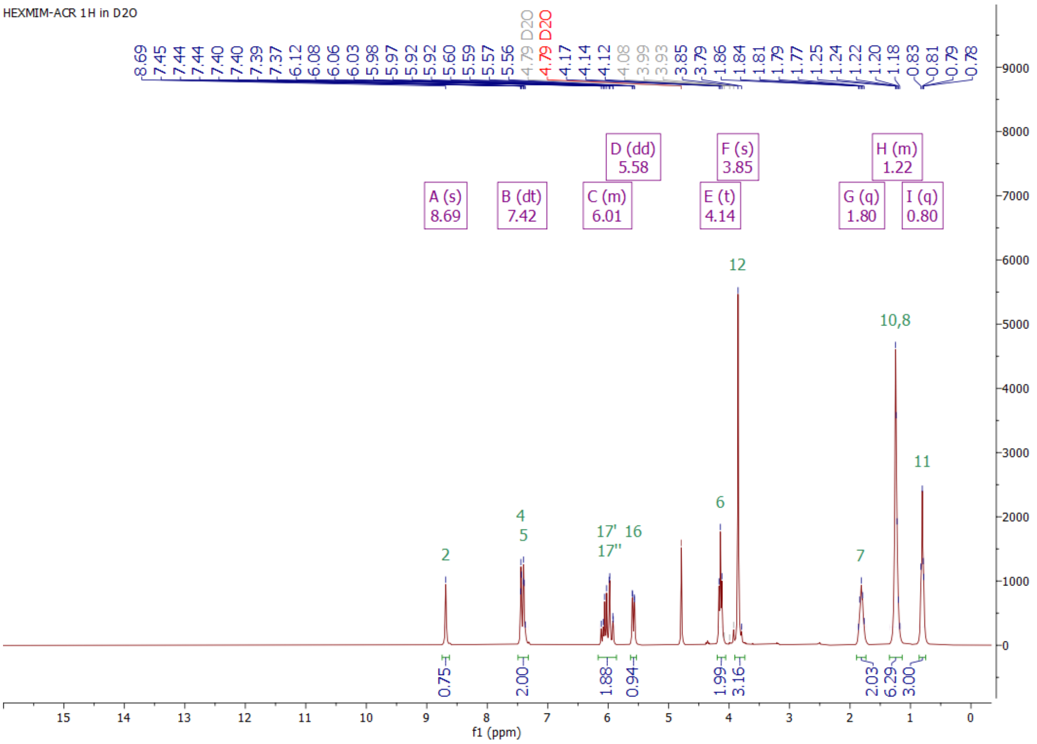

Figure S50. ^{1}H NMR of [HEXMIM][OAc] in D_2O

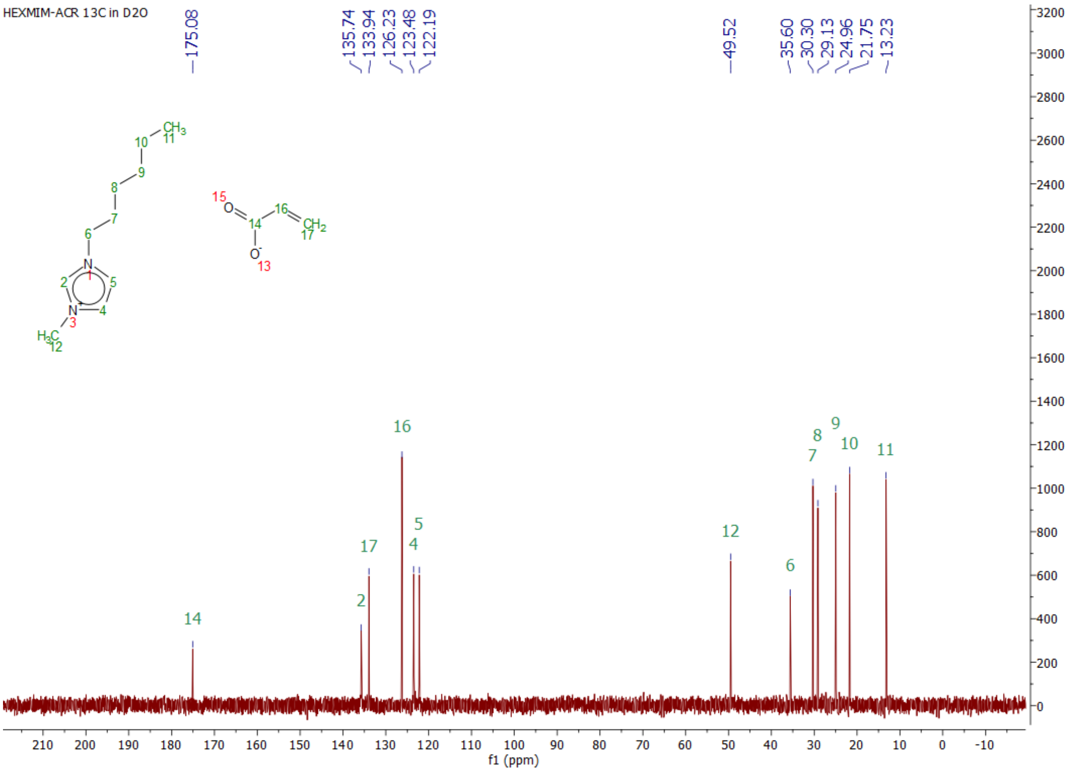

Figure S51. ^{13}C NMR of [HEXMIM][OAc] in D_2O

[Pyrr][OAc]

^{1}H NMR (400 MHz, D_2O) δ 3.31 (t, *J* = 6.8 Hz, 1H), 3.17 (t, *J* = 6.9 Hz, 1H), 3.07 (tt, *J* = 4.8, 2.4 Hz, 8H), 1.96 – 1.66 (m, 6H). ^{13}C NMR (101 MHz, D_2O) δ 180.23, 171.51, 47.69, 45.70, 45.15, 45.09, 45.05, 25.21, 24.03, 23.56, 23.22, 23.19, 23.14, 21.22.

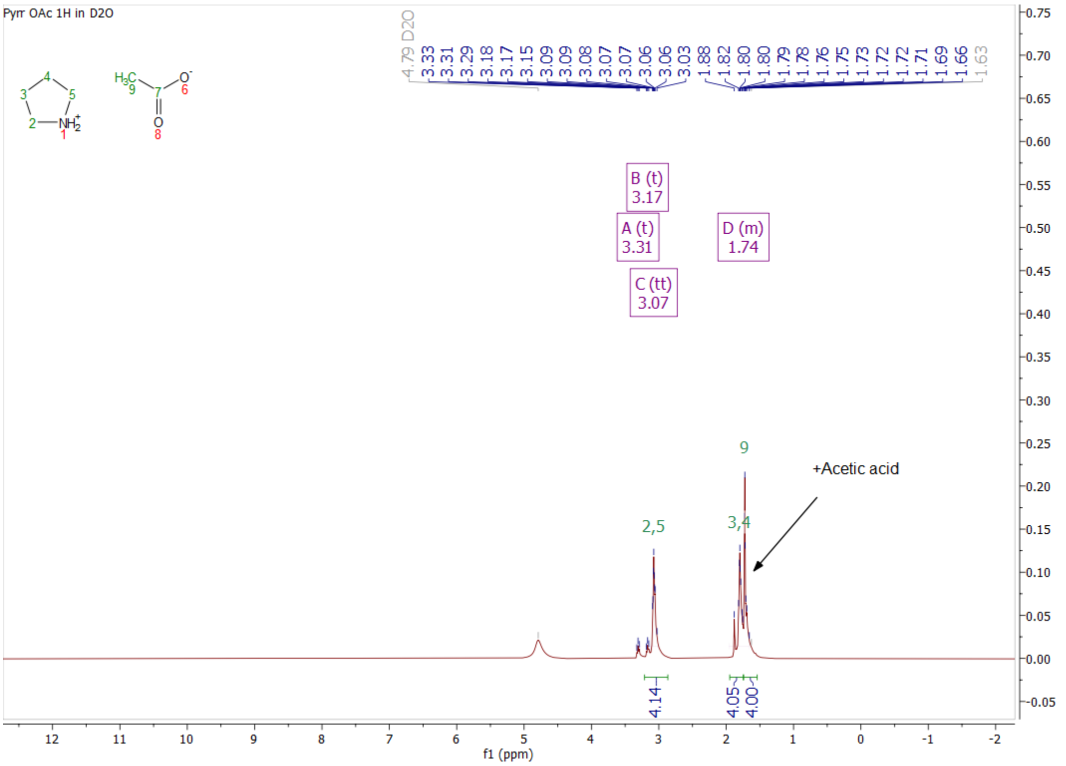

Figure S52. ^{1}H NMR of [Pyrr][OAc] in d_6-DMSO

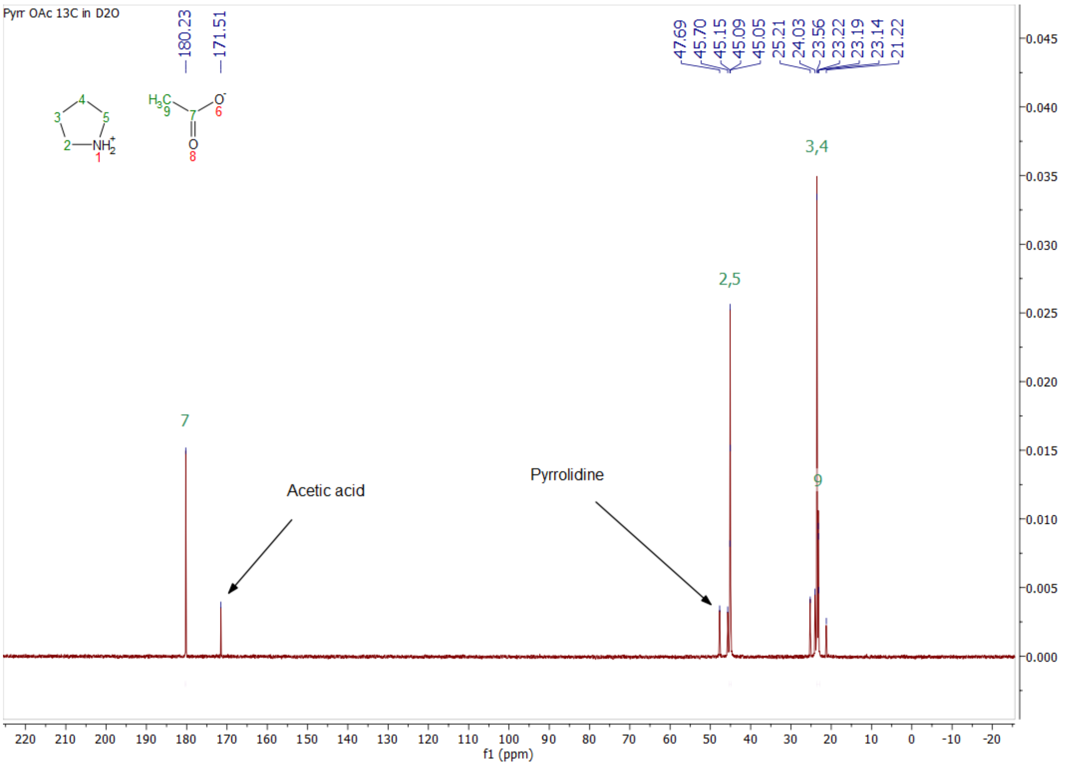

Figure S53. ^{13}C NMR of [Pyrr][OAc] in d_6-DMSO

[Pyrr][OFm]

^{1}H NMR (400 MHz, D_2O) δ 8.11 (s, 1H), 3.57 – 3.18 (m, 5H), 1.89 – 1.79 (m, 3H) ^{13}C NMR (101 MHz, D_2O) δ 167.19, 162.71, 47.02, 46.85, 45.47, 43.46, 43.29, 24.45, 23.84.

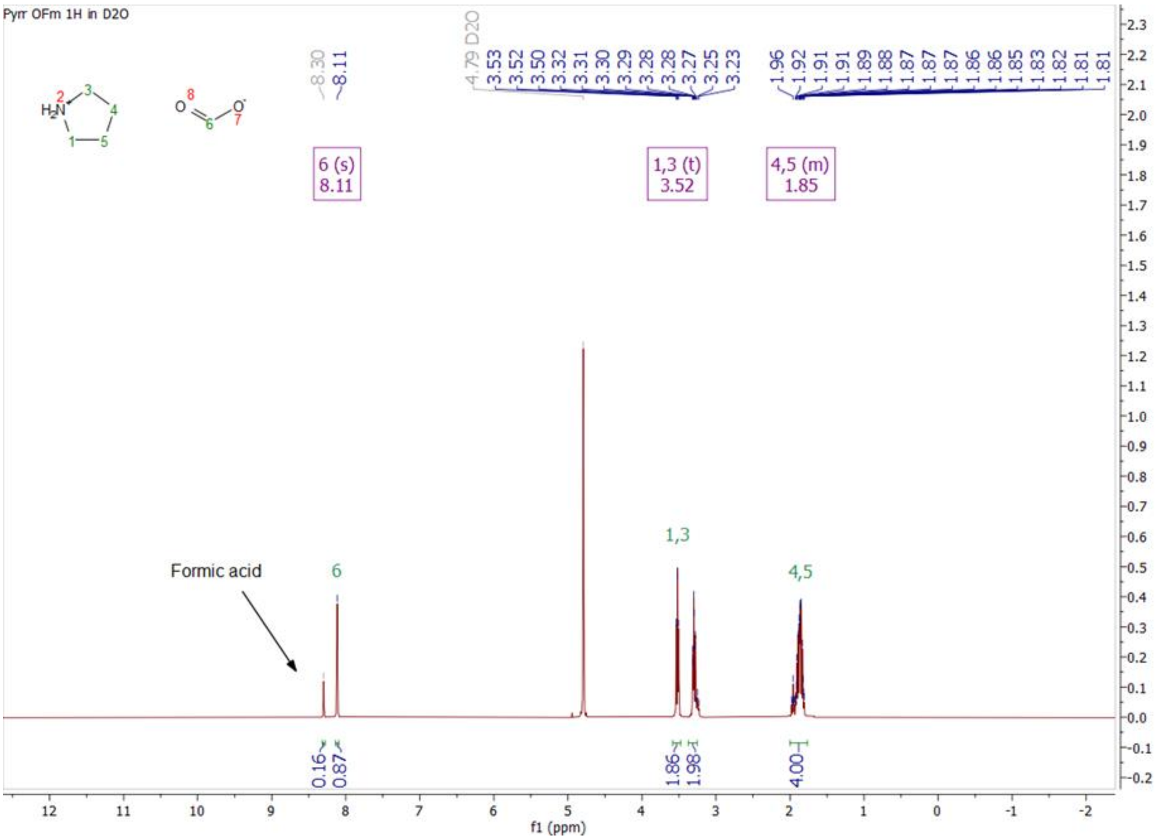

Figure S54. ^{1}H NMR of [Pyrr][OFm] in D_2O

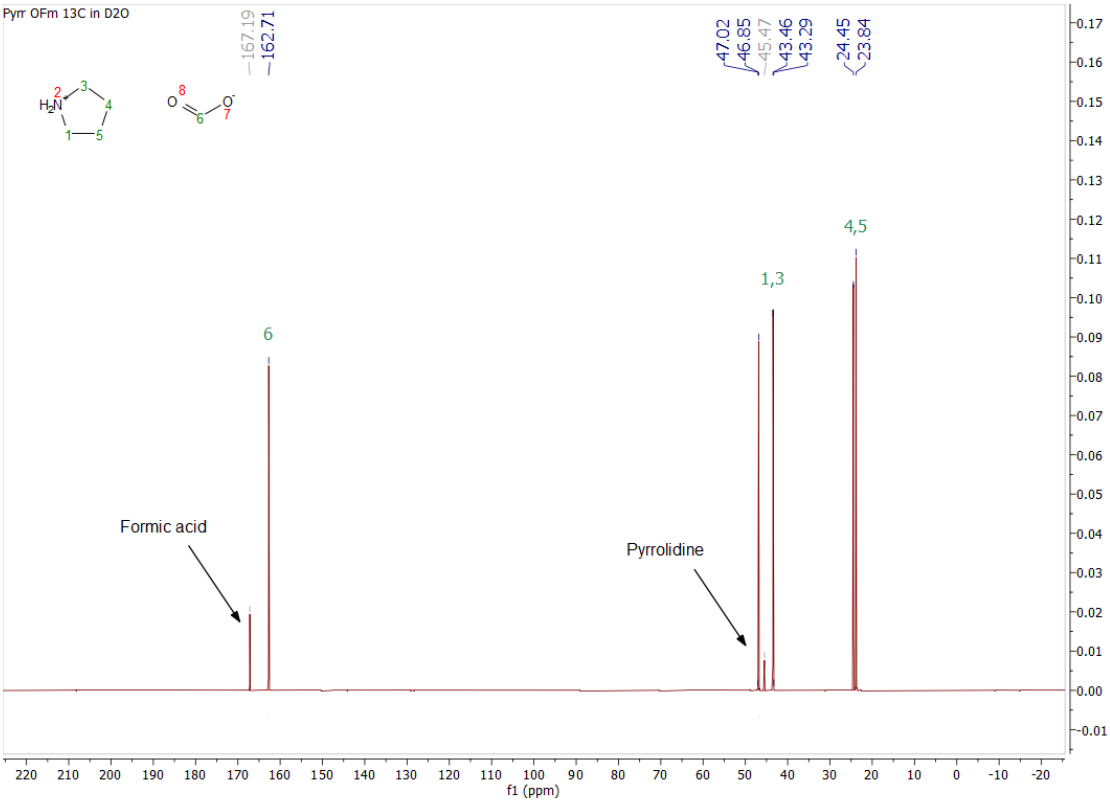

Figure S55. ^{13}C NMR of [Pyrr][OFm] in D_2O

[TEAH][$MeSO_3$]

^{1}H NMR (300 MHz, D_2O) δ 3.18 (q, *J* = 7.3 Hz, 2H), 2.76 (s, 1H), 1.26 (t, *J* = 7.3 Hz, 3H). ^{13}C NMR (76 MHz, D_2O) δ 46.64, 38.55, 8.29.

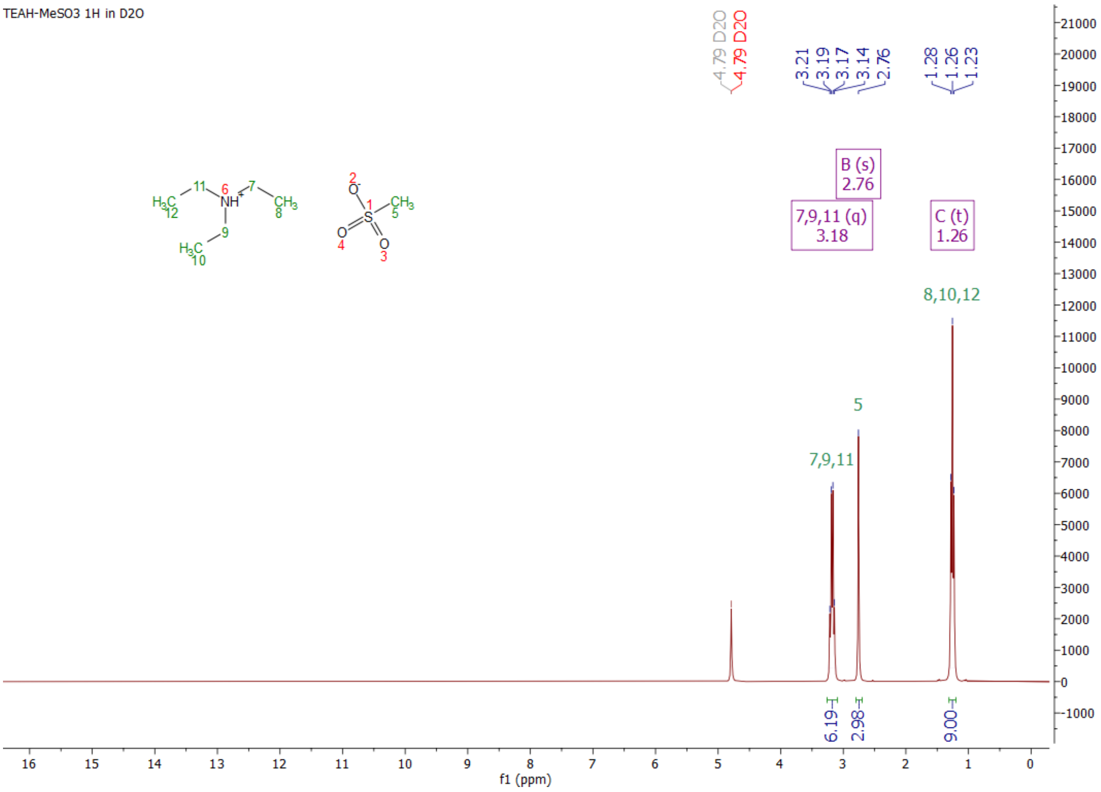

Figure S56. ^{1}H NMR of [TEAH][$MeSO_3$] in D_2O

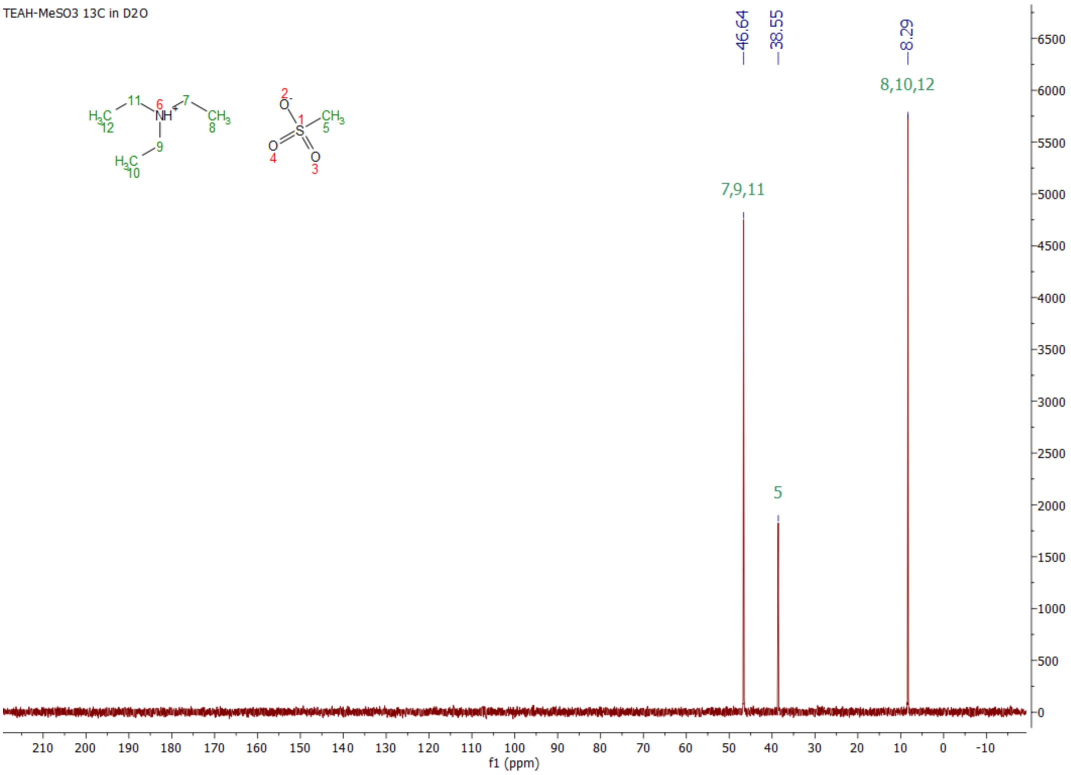

Figure S57. ^{13}C NMR of [TEAH][$MeSO_3$] in D_2O

References

1. Waldner, C.; Hirn, U., Modeling liquid penetration into porous materials based on substrate and liquid surface energies. *J. Colloid Interface Sci.* **2023,** *640*, 445-455.
2. Birdi, K. S., *Handbook of Surface and Colloid Chemistry* 2nd Edition ed.; CRC Press: Boca Raton, **2002**.
3. Fowkes, F. M.; Maruchi, S., Acid-Base Interactions in Polymer Adsorption. *ACS Coat. Plast.* **1977,** (Preprint 37), 605-610.
4. Gebhardt, K. F., *Grundlagen der physikalischen Chemie von Grenzflächen und Methoden zur Bestimmung grenzflächenenergetischer Probleme*. FhG IGB Stuttgart: **1982**.
5. Jańczuk, B.; Białopiotrowicz, T.; Wójcik, W., The components of surface tension of liquids and their usefulness in determinations of surface free energy of solids. *J. Colloid Interface Sci.* **1989,** *127* (1), 59-66.
6. Sell, P.-J.; Renzow, D., Bestimmung des benetzungsverhaltens von pigmenten. *Prog. Org. Coat.* **1975,** *3* (4), 323-348.
7. Ström, G.; Fredriksson, M.; Stenius, P., Contact angles, work of adhesion, and interfacial tensions at a dissolving Hydrocarbon surface. *J. Colloid Interface Sci.* **1987,** *119* (2), 352-361.
8. Busscher, H. J.; van Pelt, A. W. J.; de Boer, P.; de Jong, H. P.; Arends, J., The effect of surface roughening of polymers on measured contact angles of liquids. *Colloids Surf.* **1984,** *9* (4), 319-331.
9. Jie-Rong, C.; Wakida, T., Studies on the surface free energy and surface structure of PTFE film treated with low temperature plasma. *J. Appl. Polym. Sci.* **1997,** *63* (13), 1733-1739.
10. Fowkes, F. M., Attractive Forces at Interfaces. *Ind. Eng. Chem.* **1964,** *56* (12), 40-52.
11. Rabel, W., Einige Aspekte der Benetzungstheorie und ihre Anwendung auf die Untersuchung und Veränderung der Oberflächeneigenschaften von Polymeren. *Farbe und Lack* **1971,** *77*, 997-1006.
12. Van Oss, C. J.; Ju, L.; Chaudhury, M. K.; Good, R. J., Estimation of the polar parameters of the surface tension of liquids by contact angle measurements on gels. *J. Colloid Interface Sci.* **1989,** *128* (2), 313-319.
13. Lechner, M. D.; Wohlfarth, C.; Wohlfarth, B., *Surface tension of pure liquids and binary liquid mixtures*. Springer-Verlag Berlin Heidelberg: **1997**; Vol. 16.
14. Jasper, J. J., The Surface Tension of Pure Liquid Compounds. *J. Phys. Chem. Ref. Data* **1972,** *1* (4), 841-1010.
15. Berry, J. D.; Neeson, M. J.; Dagastine, R. R.; Chan, D. Y.; Tabor, R. F., Measurement of surface and interfacial tension using pendant drop tensiometry. *J. Colloid Interface Sci.* **2015,** *454*, 226-237.
16. Almeida, H. F. D.; Passos, H.; Lopes-da-Silva, J. A.; Fernandes, A. M.; Freire, M. G.; Coutinho, J. A. P., Thermophysical Properties of Five Acetate-Based Ionic Liquids. *J. Chem. Eng. Data* **2012,** *57* (11), 3005-3013.
17. Araújo, J. M. M.; Pereiro, A. B.; Alves, F.; Marrucho, I. M.; Rebelo, L. P. N., Nucleic acid bases in 1-alkyl-3-methylimidazolium acetate ionic liquids: A thermophysical and ionic conductivity analysis. *J. Chem. Thermodyn.* **2013,** *57*, 1-8.
18. Lago, S.; Rodríguez, H.; Arce, A.; Soto, A., Improved concentration of citrus essential oil by solvent extraction with acetate ionic liquids. *Fluid Phase Equilib.* **2014,** *361*, 37-44.
19. Guan, W.; Ma, X. X.; Li, L.; Tong, J.; Fang, D. W.; Yang, J. Z., Ionic parachor and its application in acetic acid ionic liquid homologue 1-alkyl-3-methylimidazolium acetate {[$C_n$mim][OAc](n = 2,3,4,5,6)}. *J. Phys. Chem. B* **2011,** *115* (44), 12915-12920.
20. Tariq, M.; Forte, P. A. S.; Gomes, M. F. C.; Lopes, J. N. C.; Rebelo, L. P. N., Densities and refractive indices of imidazolium- and phosphonium-based ionic liquids: Effect of temperature, alkyl chain length, and anion. *J. Chem. Thermodyn.* **2009,** *41* (6), 790-798.
21. Safarov, J.; Geppert-Rybczynska, M.; Kul, I.; Hassel, E., Thermophysical properties of 1-butyl-3-methylimidazolium acetate over a wide range of temperatures and pressures. *Fluid Phase Equilib.* **2014,** *383*, 144-155.
22. Pinkert, A.; Ang, K. L.; Marsh, K. N.; Pang, S., Density, viscosity and electrical conductivity of protic alkanolammonium ionic liquids. *Phys. Chem. Chem. Phys.* **2011,** *13* (11), 5136-5143.

23. Hiraga, Y.; Kato, A.; Sato, Y.; Smith, R. L., Densities at Pressures up to 200 MPa and Atmospheric Pressure Viscosities of Ionic Liquids 1-Ethyl-3-methylimidazolium Methylphosphate, 1-Ethyl-3-methylimidazolium Diethylphosphate, 1-Butyl-3-methylimidazolium Acetate, and 1-Butyl-3-methylimidazolium Bis(trifluoromethylsulfonyl)imide. *J. Chem. Eng. Data* **2015,** *60* (3), 876-885.
24. Xu, A. R.; Wang, J. J.; Zhang, Y. J.; Chen, Q. T., Effect of Alkyl Chain Length in Anions on Thermodynamic and Surface Properties of 1-Butyl-3-methylimidazolium Carboxylate Ionic Liquids. *Ind. Eng. Chem* **2012,** *51* (8), 3458-3465.
25. Haghtalab, A.; Shojaeian, A., Volumetric and viscometric behaviour of the binary systems of N-methyldiethanolamine and diethanolamine with 1-butyl-3-methylimidazolium acetate at various temperatures. *J. Chem. Thermodyn.* **2014,** *68*, 128-137.
26. Shiflett, M. B.; Kasprzak, D. J.; Junk, C. P.; Yokozeki, A., Phase behavior of {carbon dioxide plus [bmim][Ac]} mixtures. *J. Chem. Thermodyn.* **2008,** *40* (1), 25-31.
27. Shiflett, M. B.; Harmer, M. A.; Junk, C. P.; Yokozeki, A., Solubility and diffusivity of difluoromethane in room-temperature ionic liquids. *J. Chem. Eng. Data* **2006,** *51* (2), 483-495.
28. McHale, G.; Hardacre, C.; Ge, R.; Doy, N.; Allen, R. W.; MacInnes, J. M.; Bown, M. R.; Newton, M. I., Density-viscosity product of small-volume ionic liquid samples using quartz crystal impedance analysis. *Anal. Chem.* **2008,** *80* (15), 5806-5811.
29. Losetty, V.; Matheswaran, P.; Wilfred, C. D., Synthesis, thermophysical properties and COSMO-RS study of DBU based protic ionic liquids. *J. Chem. Thermodyn.* **2017,** *105*, 151-158.
30. Freire, M. G.; Teles, A. R. R.; Rocha, M. A. A.; Schröder, B.; Neves, C. M. S. S.; Carvalho, P. J.; Evtuguin, D. V.; Santos, L. M. N. B. F.; Coutinho, J. A. P., Thermophysical Characterization of Ionic Liquids Able To Dissolve Biomass. *J. Chem. Eng. Data* **2011,** *56* (12), 4813-4822.
31. Seki, S.; Tsuzuki, S.; Hayamizu, K.; Umebayashi, Y.; Serizawa, N.; Takei, K.; Miyashiro, H., Comprehensive Refractive Index Property for Room-Temperature Ionic Liquids. *J. Chem. Eng. Data* **2012,** *57* (8), 2211-2216.
32. Larriba, M.; Navarro, P.; García, J.; Rodríguez, F., Liquid-liquid extraction of toluene from n-heptane by {[emim][TCM] plus [emim][DCA]} binary ionic liquid mixtures. *Fluid Phase Equilib.* **2014,** *364*, 48-54.
33. França, J. M. P.; Reis, F.; Vieira, S. I. C.; Lourenço, M. J. V.; Santos, F. J. V.; Nieto de Castro, C. A.; Pádua, A. A. H., Thermophysical properties of ionic liquid dicyanamide (DCA) nanosystems. *J. Chem. Thermodyn.* **2014,** *79*, 248-257.
34. Quijada-Maldonado, E.; van der Boogaart, S.; Lijbers, J. H.; Meindersma, G. W.; de Haan, A. B., Experimental densities, dynamic viscosities and surface tensions of the ionic liquids series 1-ethyl-3-methylimidazolium acetate and dicyanamide and their binary and ternary mixtures with water and ethanol at T=(298.15 to 343.15K). *J. Chem. Thermodyn.* **2012,** *51*, 51-58.
35. Schreiner, C.; Zugmann, S.; Hartl, R.; Gores, H. J., Fractional Walden Rule for Ionic Liquids: Examples from Recent Measurements and a Critique of the So-Called Ideal KCl Line for the Walden Plot. *J. Chem. Eng. Data* **2010,** *55* (5), 1784-1788.
36. Wong, C. L.; Soriano, A. N.; Li, M. H., Diffusion coefficients and molar conductivities in aqueous solutions of 1-ethyl-3-methylimidazolium-based ionic liquids. *Fluid Phase Equilib.* **2008,** *271* (1-2), 43-52.
37. Stoppa, A.; Buchner, R.; Hefter, G., How ideal are binary mixtures of room-temperature ionic liquids? *J. Mol. Liq.* **2010,** *153* (1), 46-51.
38. Klomfar, J.; Součková, M.; Pátek, J., P–ρ–T Measurements for 1-Ethyl and 1-Butyl-3-methylimidazolium Dicyanamides from Their Melting Temperature to 353 K and up to 60 MPa in Pressure. *J. Chem. Eng. Data* **2012,** *57* (4), 1213-1221.
39. Rodríguez-Cabo, B.; Arce, A.; Soto, A., Desulfurization of fuels by liquid–liquid extraction with 1-ethyl-3-methylimidazolium ionic liquids. *Fluid Phase Equilib.* **2013,** *356*, 126-135.
40. Wang, J. Y.; Zhao, F. Y.; Liu, R. J.; Hu, Y. Q., Thermophysical properties of 1-methyl-3-methylimidazolium dimethylphosphate and 1-ethyl-3-methylimidazolium diethylphosphate. *J. Chem. Thermodyn.* **2011,** *43* (1), 47-50.
41. Wan Normazlan, W. M. D.; Sairi, N. A.; Alias, Y.; Udaiyappan, A. F.; Jouyban, A.; Khoubnasabjafari, M., Composition and Temperature Dependence of Density, Surface Tension, and

Viscosity of EMIM DEP/MMIM DMP + Water + 1-Propanol/2-Propanol Ternary Mixtures and Their Mathematical Representation Using the Jouyban–Acree Model. *J. Chem. Eng. Data* **2014,** *59* (8), 2337-2348.
42. Ficke, L. E.; Novak, R. R.; Brennecke, J. F., Thermodynamic and Thermophysical Properties of Ionic Liquid plus Water Systems. *J. Chem. Eng. Data* **2010,** *55* (11), 4946-4950.
43. Wang, J. F.; Li, C. X.; Shen, C.; Wang, Z. H., Towards understanding the effect of electrostatic interactions on the density of ionic liquids. *Fluid Phase Equilib.* **2009,** *279* (2), 87-91.
44. Palgunadi, J.; Kang, J. E.; Nguyen, D. Q.; Kim, J. H.; Min, B. K.; Lee, S. D.; Kim, H.; Kim, H. S., Solubility of CO in dialkylimidazolium dialkylphosphate ionic liquids. *Thermochim. Acta* **2009,** *494* (1-2), 94-98.
45. Cai, F. F.; Ibrahim, J. J.; Gao, L. J.; Wei, R. P.; Xiao, G. M., A study on the liquid-liquid equilibrium of 1-alkyl-3-methylimidazolium dialkylphosphate with methanol and dimethyl carbonate. *Fluid Phase Equilib.* **2014,** *382*, 254-259.
46. Shah, M. R.; Anantharaj, R.; Banerjee, T.; Yadav, G. D., Quaternary (liquid+liquid) equilibria for systems of imidazolium based ionic liquid+thiophene+pyridine+cyclohexane at 298.15K: Experiments and quantum chemical predictions. *J. Chem. Thermodyn.* **2013,** *62*, 142-150.
47. Rabari, D.; Patel, N.; Joshipura, M.; Banerjee, T., Densities of Six Commercial Ionic Liquids: Experiments and Prediction Using a Cohesion Based Cubic Equation of State. *J. Chem. Eng. Data* **2014,** *59* (3), 571-578.
48. Hasse, B.; Lehmann, J.; Assenbaum, D.; Wasserscheid, P.; Leipertz, A.; Fröba, A. P., Viscosity, Interfacial Tension, Density, and Refractive Index of Ionic Liquids [EMIM][$MeSO_3$], [EMIM][$MeOHPO_2$], [EMIM][$OcSO_4$], and [BBIM][NTf_2] in Dependence on Temperature at Atmospheric Pressure. *J. Chem. Eng. Data* **2009,** *54* (9), 2576-2583.
49. Anantharaj, R.; Banerjee, T., Thermodynamic properties of 1-ETHYL-3-methylimidazolium methanesulphonate with aromatic sulphur, nitrogen compounds at T = 298.15–323.15 K and P = 1 bar. *Can. J. Chem. Eng.* **2012,** *91* (2), 245-256.
50. Singh, M. P.; Mandal, S. K.; Verma, Y. L.; Gupta, A. K.; Singh, R. K.; Chandra, S., Viscoelastic, Surface, and Volumetric Properties of Ionic Liquids [BMIM][$OcSO_4$], [BMIM][PF_6], and [EMIM][$MeSO_3$]. *J. Chem. Eng. Data* **2014,** *59* (8), 2349-2359.
51. Domańska, U.; Królikowski, M., Measurements of activity coefficients at infinite dilution for organic solutes and water in the ionic liquid 1-ethyl-3-methylimidazolium methanesulfonate. *J. Chem. Thermodyn.* **2012,** *54*, 20-27.
52. Nazet, A.; Sokolov, S.; Sonnleitner, T.; Makino, T.; Kanakubo, M.; Buchner, R., Densities, Viscosities, and Conductivities of the Imidazolium Ionic Liquids [Emim][Ac], [Emim][FAP], [Bmim][BETI], [Bmim][FSI], [Hmim][TFSI], and [Omim][TFSI]. *J. Chem. Eng. Data* **2015,** *60* (8), 2400-2411.
53. Shiflett, M. B.; Yokozeki, A., Phase Behavior of Carbon Dioxide in Ionic Liquids: [emim][Acetate], [emim][Trifluoroacetate], and [emim][Acetate] plus [emim][Trifluoroacetate] Mixtures. *J. Chem. Eng. Data* **2009,** *54* (1), 108-114.
54. Castro, M. C.; Rodríguez, H.; Arce, A.; Soto, A., Mixtures of Ethanol and the Ionic Liquid 1-Ethyl-3-methylimidazolium Acetate for the Fractionated Solubility of Biopolymers of Lignocellulosic Biomass. *Ind. Eng. Chem* **2014,** *53* (29), 11850-11861.
55. Fröba, A. P.; Rausch, M. H.; Krzeminski, K.; Assenbaum, D.; Wasserscheid, P.; Leipertz, A., Thermal Conductivity of Ionic Liquids: Measurement and Prediction. *Int. J. Thermophys.* **2010,** *31* (11-12), 2059-2077.
56. Oliveira, F. S.; Rebelo, L. P. N.; Marrucho, I. M., Influence of Different Inorganic Salts on the Ionicity and Thermophysical Properties of 1-Ethyl-3-methylimidazolium Acetate Ionic Liquid. *J. Chem. Eng. Data* **2015,** *60* (3), 781-789.
57. Corderí, S.; Gómez, E.; Calvar, N.; Domínguez, Á., Measurement and Correlation of Liquid–Liquid Equilibria for Ternary and Quaternary Systems of Heptane, Cyclohexane, Toluene, and [EMim][OAc] at 298.15 K. *Ind. Eng. Chem* **2014,** *53* (22), 9471-9477.
58. Rosenboom, J. G.; Afzal, W.; Prausnitz, J. M., Solubilities of some organic solutes in 1-ethyl-3-methylimidazolium acetate. Chromatographic measurements and predictions from COSMO-RS. *J. Chem. Thermodyn.* **2012,** *47*, 320-327.

59. Pinto, A. M.; Rodríguez, H.; Arce, A.; Soto, A., Combined physical and chemical absorption of carbon dioxide in a mixture of ionic liquids. *J. Chem. Thermodyn.* **2014,** *77*, 197-205.
60. Pereiro, A. B.; Araujo, J. M.; Oliveira, F. S.; Bernardes, C. E.; Esperanca, J. M.; Lopes, J. N.; Marrucho, I. M.; Rebelo, L. P., Inorganic salts in purely ionic liquid media: the development of High Ionicity Ionic Liquids (HIILs). *Chem Commun (Camb)* **2012,** *48* (30), 3656-3658.
61. Vercher, E.; Llopis, F. J.; González-Alfaro, V.; Miguel, P. J.; Martínez-Andreu, A., Refractive Indices and Deviations in Refractive Indices of Trifluoromethanesulfonate-Based Ionic Liquids in Water. *J. Chem. Eng. Data* **2011,** *56* (12), 4499-4504.
62. Montalbán, M. G.; Bolívar, C. L.; Díaz Baños, F. G.; Víllora, G., Effect of Temperature, Anion, and Alkyl Chain Length on the Density and Refractive Index of 1-Alkyl-3-methylimidazolium-Based Ionic Liquids. *J. Chem. Eng. Data* **2015,** *60* (7), 1986-1996.
63. Gardas, R. L.; Costa, H. F.; Freire, M. G.; Carvalho, P. J.; Marrucho, I. M.; Fonseca, I. M. A.; Ferreira, A. G. M.; Coutinho, J. A. P., Densities and Derived Thermodynamic Properties of Imidazolium-, Pyridinium-, Pyrrolidinium-, and Piperidinium-Based Ionic Liquids. *J. Chem. Eng. Data* **2008,** *53* (3), 805-811.
64. Klomfar, J.; Soucková, M.; Pátek, J., Temperature Dependence of the Surface Tension and Density at 0.1 MPa for 1-Ethyl- and 1-Butyl-3-methylimidazolium Dicyanamide. *J. Chem. Eng. Data* **2011,** *56* (8), 3454-3462.
65. Rodríguez, H.; Brennecke, J. F., Temperature and Composition Dependence of the Density and Viscosity of Binary Mixtures of Water + Ionic Liquid. *J. Chem. Eng. Data* **2006,** *51* (6), 2145-2155.
66. Yusoff, R.; Aroua, M. K.; Shamiri, A.; Ahmady, A.; Jusoh, N. S.; Asmuni, N. F.; Bong, L. C.; Thee, S. H., Density and Viscosity of Aqueous Mixtures of N-Methyldiethanolamines (MDEA) and Ionic Liquids. *J. Chem. Eng. Data* **2013,** *58* (2), 240-247.
67. Mbondo Tsamba, B. E.; Sarraute, S.; Traïkia, M.; Husson, P., Transport Properties and Ionic Association in Pure Imidazolium-Based Ionic Liquids as a Function of Temperature. *J. Chem. Eng. Data* **2014,** *59* (6), 1747-1754.
68. Vuksanovic, J. M.; Calado, M. S.; Ivanis, G. R.; Kijevcanin, M. L.; Serbanovic, S. P.; Visak, Z. P., Environmentally friendly solutions of liquid poly(ethylene glycol) and imidazolium based ionic liquids with bistriflamide and triflate anions: Volumetric and viscosity studies. *Fluid Phase Equilib.* **2013,** *352*, 100-109.
69. García-Miaja, G.; Troncoso, J.; Romaní, L., Excess properties for binary systems ionic liquid+ethanol: Experimental results and theoretical description using the ERAS model. *Fluid Phase Equilib.* **2008,** *274* (1-2), 59-67.
70. Larriba, M.; Navarro, P.; García, J.; Rodríguez, F., Selective extraction of toluene from n-heptane using [emim][SCN] and [bmim][SCN] ionic liquids as solvents. *J. Chem. Thermodyn.* **2014,** *79*, 266-271.
71. Klomfar, J.; Součková, M.; Pátek, J., Low-Temperature and High-Pressure p–ρ–T Relation for 1-(2-Methoxyethyl)-1-methylpyrrolidinium Bis(trifluoromethylsulfonyl)imide and 1-C_n-3-methylimidazolium Thiocyanate with n = 2 and 4. *J. Chem. Eng. Data* **2015,** *60* (6), 1855-1867.
72. Królikowska, M.; Hofman, T., Densities, isobaric expansivities and isothermal compressibilities of the thiocyanate-based ionic liquids at temperatures (298.15–338.15K) and pressures up to 10MPa. *Thermochim. Acta* **2012,** *530*, 1-6.
73. Froba, A. P.; Kremer, H.; Leipertz, A., Density, refractive index, interfacial tension, and viscosity of ionic liquids [EMIM][$EtSO_4$], [EMIM][NTf_2], [EMIM][$N(CN)_2$], and [OMA][NTf_2] in dependence on temperature at atmospheric pressure. *J. Phys. Chem. B* **2008,** *112* (39), 12420-12430.
74. Wandschneider, A.; Lehmann, J. K.; Heintz, A., Surface tension and density of pure ionic liquids and some binary mixtures with 1-propanol and 1-butanol. *J. Chem. Eng. Data* **2008,** *53* (2), 596-599.
75. Miran Beigi, A. A.; Abdouss, M.; Yousefi, M.; Pourmortazavi, S. M.; Vahid, A., Investigation on physical and electrochemical properties of three imidazolium based ionic liquids (1-hexyl-3-methylimidazolium tetrafluoroborate, 1-ethyl-3-methylimidazolium bis(trifluoromethylsulfonyl) imide and 1-butyl-3-methylimidazolium methylsulfate). *J. Mol. Liq.* **2013,** *177*, 361-368.

76. Seoane, R. G.; Corderí, S.; Gómez, E.; Calvar, N.; González, E. J.; Macedo, E. A.; Domínguez, A., Temperature Dependence and Structural Influence on the Thermophysical Properties of Eleven Commercial Ionic Liquids. *Ind. Eng. Chem* **2012,** *51* (5), 2492-2504.
77. Bansal, S.; Kaur, N.; Chaudhary, G. R.; Mehta, S. K.; Ahluwalia, A. S., Physiochemical Properties of New Formulations of 1-Ethyl-3-methylimidazolium Bis(trifluoromethylsulfonyl)imide with Tritons. *J. Chem. Eng. Data* **2014,** *59* (12), 3988-3999.
78. Seoane, R. G.; González, E. J.; González, B., 1-Alkyl-3-methylimidazolium bis(trifluoromethylsulfonyl)imide ionic liquids as solvents in the separation of azeotropic mixtures. *J. Chem. Thermodyn.* **2012,** *53*, 152-157.
79. Choi, Y.-Y.; Hwang, I.-C.; Shin, S.-H.; Park, S.-J., Liquid–liquid equilibria, excess molar volume and deviations of the refractive indices at 298.15K for mixtures of solvents used in the molybdenum extraction process. *Fluid Phase Equilib.* **2013,** *354*, 59-65.
80. Corderí, S.; González, B., Ethanol extraction from its azeotropic mixture with hexane employing different ionic liquids as solvents. *J. Chem. Thermodyn.* **2012,** *55*, 138-143.
81. Lago, S.; Rodríguez, H.; Soto, A.; Arce, A., Deterpenation of Citrus Essential Oil by Liquid-Liquid Extraction with 1-Alkyl-3-methylimidazolium Bis(trifluoromethylsulfonyl)amide Ionic Liquids. *J. Chem. Eng. Data* **2011,** *56* (4), 1273-1281.
82. Tariq, M.; Serro, A. P.; Mata, J. L.; Saramago, B.; Esperança, J. M. S. S.; Lopes, J. N. C.; Rebelo, L. P. N., High-temperature surface tension and density measurements of 1-alkyl-3-methylimidazolium bistriflamide ionic liquids. *Fluid Phase Equilib.* **2010,** *294* (1-2), 131-138.
83. Jacquemin, J.; Husson, P.; Mayer, V.; Cibulka, I., High-pressure volumetric properties of imidazolium-based ionic liquids: Effect of the anion. *J. Chem. Eng. Data* **2007,** *52* (6), 2204-2211.
84. Součková, M.; Klomfar, J.; Pátek, J., Measurements and group contribution analysis of 0.1MPa densities for still poorly studied ionic liquids with the [PF_6] and [NTf_2] anions. *J. Chem. Thermodyn.* **2014,** *77*, 31-39.
85. Jacquemin, J.; Husson, P.; Padua, A. A. H.; Majer, V., Density and viscosity of several pure and water-saturated ionic liquids. *Green Chem.* **2006,** *8* (2), 172-180.
86. Gardas, R. L.; Freire, M. G.; Carvalho, P. J.; Marrucho, I. M.; Fonseca, I. M. A.; Ferreira, A. G. M.; Coutinho, J. A. P., measurements of imidazolium-based ionic liquids. *J. Chem. Eng. Data* **2007,** *52* (5), 1881-1888.
87. Dzida, M.; Chorazewski, M.; Geppert-Rybczynska, M.; Zorebski, E.; Zorebski, M.; Zarska, M.; Czech, B., Speed of Sound and Adiabatic Compressibility of 1-Ethyl-3-methylimidazolium Bis(trifluoromethylsulfonyl)imide under Pressures up to 100 MPa. *J. Chem. Eng. Data* **2013,** *58* (6), 1571-1576.
88. Safarov, J.; El-Awady, W. A.; Shahverdiyev, A.; Hassel, E., Thermodynamic Properties of 1-Ethyl-3-methylimidazolium Bis(trifluoromethylsulfonyl)imide. *J. Chem. Eng. Data* **2011,** *56* (1), 106-112.
89. Krummen, M.; Wasserscheid, P.; Gmehling, J., Measurement of activity coefficients at infinite dilution in ionic liquids using the dilutor technique. *J. Chem. Eng. Data* **2002,** *47* (6), 1411-1417.
90. Salinas, R.; Pla-Franco, J.; Lladosa, E.; Montón, J. B., Density, Speed of Sound, Viscosity, and Excess Properties of Binary Mixtures Formed by Ethanol and Bis(trifluorosulfonyl)imide-Based Ionic Liquids. *J. Chem. Eng. Data* **2015,** *60* (3), 525-540.
91. Fredlake, C. P.; Crosthwaite, J. M.; Hert, D. G.; Aki, S. N. V. K.; Brennecke, J. F., Thermophysical properties of imidazolium-based ionic liquids. *J. Chem. Eng. Data* **2004,** *49* (4), 954-964.
92. Yao, H.; Zhang, S.; Wang, J.; Zhou, Q.; Dong, H.; Zhang, X., Densities and Viscosities of the Binary Mixtures of 1-Ethyl-3-methylimidazolium Bis(trifluoromethylsulfonyl)imide with N-Methyl-2-pyrrolidone or Ethanol at T = (293.15 to 323.15) K. *J. Chem. Eng. Data* **2012,** *57* (3), 875-881.
93. Tokuda, H.; Tsuzuki, S.; Susan, M. A.; Hayamizu, K.; Watanabe, M., How ionic are room-temperature ionic liquids? An indicator of the physicochemical properties. *J. Phys. Chem. B* **2006,** *110* (39), 19593-19600.
94. Burrell, G. L.; Burgar, I. M.; Separovic, F.; Dunlop, N. F., Preparation of protic ionic liquids with minimal water content and ^{15}N NMR study of proton transfer. *Phys. Chem. Chem. Phys.* **2010,** *12* (7), 1571-1577.

95. Iojoiu, C.; Martinez, M.; Hanna, M.; Molmeret, Y.; Cointeaux, L.; Leprêtre, J. C.; El Kissi, N.; Guindet, J.; Judeinstein, P.; Sanchez, J. Y., PILs-based Nafion membranes: a route to high-temperature PEFMCs dedicated to electric and hybrid vehicles. *Polym. Adv. Technol.* **2008,** *19* (10), 1406-1414.
96. de Oliveira, L. H.; Aznar, M. n., Liquid−Liquid Equilibria for {1-Ethyl-3-methylimidazolium Diethylphosphate or 1-Ethyl-3-methylimidazolium Ethylsulfate} + 4,6-Dimethyldibenzothiophene + Dodecane Systems at 298.2 K and 313.2 K. *J. Chem. Eng. Data* **2011,** *56* (5), 2005-2012.
97. Castro, M. C.; Arce, A.; Soto, A.; Rodríguez, H., Thermophysical Characterization of the Mixtures of the Ionic Liquid 1-Ethyl-3-Methylimidazolium Acetate with 1-Propanol or 2-Propanol. *J. Chem. Eng. Data* **2016,** *61* (7), 2299-2310.
98. Wang, M. J.; Yin, T. X.; Xu, C.; Chen, Z. Y.; Shen, W. G., Liquid Liquid Phase Equilibrium and Heat Capacity of Binary Mixture 1-Ethyl-3-methylimidazolium Bis(trifluoromethylsulfonyl)imide+1-Propanol. *J. Chem. Eng. Data* **2014,** *59* (11), 3389-3396.
99. Navarro, P.; Larriba, M.; García, J.; Rodríguez, F., Thermal stability, specific heats, and surface tensions of ([emim][DCA]+[4empy][Tf2N]) ionic liquid mixtures. *J. Chem. Thermodyn.* **2014,** *76*, 152-160.
100. Fletcher, S. I.; Sillars, F. B.; Hudson, N. E.; Hall, P. J., Physical Properties of Selected Ionic Liquids for Use as Electrolytes and Other Industrial Applications. *J. Chem. Eng. Data* **2010,** *55* (2), 778-782.
101. Martino, W.; de la Mora, J. F.; Yoshida, Y.; Saito, G.; Wilkes, J., Surface tension measurements of highly conducting ionic liquids. *Green Chem.* **2006,** *8* (4), 390-397.
102. Ren, N. N.; Gong, Y. H.; Lu, Y. Z.; Meng, H.; Li, C. X., Surface Tension Measurements for Seven Imidazolium-Based Dialkylphosphate Ionic Liquids and Their Binary Mixtures with Water (Methanol or Ethanol) at 298.15 K and 1 atm. *J. Chem. Eng. Data* **2014,** *59* (2), 189-196.
103. Ghani, N. A.; Sairi, N. A.; Aroua, M. K.; Alias, Y.; Yusoff, R., Density, Surface Tension, and Viscosity of Ionic Liquids (1-Ethyl-3-methylimidazolium diethylphosphate and 1,3-Dimethylimidazolium dimethylphosphate) Aqueous Ternary Mixtures with MDEA. *J. Chem. Eng. Data* **2014,** *59* (6), 1737-1746.
104. Almeida, H. F. D.; Teles, A. R. R.; Lopes-da-Silva, J. A.; Freire, M. G.; Coutinho, J. A. P., Influence of the anion on the surface tension of 1-ethyl-3-methylimidazolium-based ionic liquids. *J. Chem. Thermodyn.* **2012,** *54*, 49-54.
105. Schuermann, J.; Huber, T.; LeCorre, D.; Mortha, G.; Sellier, M.; Duchemin, B.; Staiger, M. P., Surface tension of concentrated cellulose solutions in 1-ethyl-3-methylimidazolium acetate. *Cellulose* **2016,** *23* (2), 1043-1050.
106. Tong, J.; Ma, X.; Kong, Y. X.; Chen, Y.; Guan, W.; Yang, J. Z., Ionic parachor and its application II. Ionic liquid homologues of 1-alkyl-3-methylimidazolium propionate [C(n)mim][Pro] (n = 2-6). *J. Phys. Chem. B* **2012,** *116* (20), 5971-5976.
107. Kilaru, P.; Baker, G. A.; Scovazzo, P., Density and surface tension measurements of imidazolium-, quaternary phosphonium-, and ammonium-based room-temperature ionic liquids: Data and correlations. *J. Chem. Eng. Data* **2007,** *52* (6), 2306-2314.
108. Součková, M.; Klomfar, J.; Pátek, J., Surface tension of 1-alkyl-3-methylimidazolium based ionic liquids with trifluoromethanesulfonate and tetrafluoroborate anion. *Fluid Phase Equilib.* **2011,** *303* (2), 184-190.
109. Domańska, U.; Królikowska, M.; Królikowski, M., Phase behaviour and physico-chemical properties of the binary systems {1-ethyl-3-methylimidazolium thiocyanate, or 1-ethyl-3-methylimidazolium tosylate+water, or+an alcohol}. *Fluid Phase Equilib.* **2010,** *294* (1-2), 72-83.
110. Zhang, Q.; Li, M.; Zhang, X.; Wu, X., The Thermodynamic Estimation and Viscosity, Electrical Conductivity Characteristics of 1-Alkyl-3-Methylimidazolium Thiocyanate Ionic Liquids. *Z. Phys. Chem.* **2014,** *228* (8), 851-867.
111. Anantharaj, R.; Banerjee, T., Phase behavior of catalytic deactivated compounds and water with 1-ethyl-3-methylimidazolium acetate [EMIM][OAc] ionic liquid at T=298.15–323.15K and p=1bar. *J. Ind. Eng. Chem.* **2012,** *18* (1), 331-343.
112. Chen, Z.; Morales-Collazo, O.; Brennecke, J. F., Protic Imidazolium Cation-Based Ionic Liquids Show Unexpected Interfacial Properties. *Langmuir* **2020,** *36* (30), 8904-8913.

113. Carvalho, P. J.; Freire, M. G.; Marrucho, I. M.; Queimada, A. J.; Coutinho, J. A. P., Surface tensions for the 1-alkyl-3-methylimidazolium bis(trifluoromethylsulfonyl)imide ionic liquids. *J. Chem. Eng. Data* **2008,** *53* (6), 1346-1350.
114. Klomfar, J.; Soucková, M.; Pátek, J., Surface tension measurements with validated accuracy for four 1-alkyl-3-methylimidazolium based ionic liquids. *J. Chem. Thermodyn.* **2010,** *42* (3), 323-329.
115. Shirota, H.; Mandai, T.; Fukazawa, H.; Kato, T., Comparison between Dicationic and Monocationic Ionic Liquids: Liquid Density, Thermal Properties, Surface Tension, and Shear Viscosity. *J. Chem. Eng. Data* **2011,** *56* (5), 2453-2459.
116. Součková, M.; Klomfar, J.; Pátek, J., Surface tension and 0.1MPa densities of imidazolium-, pyridinium-, pyrrolidinium-, and piperidinium-based tris(pentafluoroethyl)trifluorophosphate ionic liquids. *Fluid Phase Equilib.* **2012,** *333*, 38-46.
117. Geppert-Rybczyńska, M.; Lehmann, J. K.; Heintz, A., Surface Tensions and the Gibbs Excess Surface Concentration of Binary Mixtures of the Ionic Liquid 1-Ethyl-3-methylimidazolium Bis[(trifluoromethyl)sulfonyl]imide with Tetrahydrofuran and Acetonitrile. *J. Chem. Eng. Data* **2011,** *56* (4), 1443-1448.
118. Harris, K. R., Temperature and Pressure Dependence of the Viscosity of the Ionic Liquid 1-Butyl-3-methylimidazolium Acetate. *J. Chem. Eng. Data* **2020,** *65* (2), 804-813.
119. Almeida, H. F. D.; Lopes, J. N. C.; Rebelo, L. P. N.; Coutinho, J. A. P.; Freire, M. G.; Marrucho, I. M., Densities and Viscosities of Mixtures of Two Ionic Liquids Containing a Common Cation. *J. Chem. Eng. Data* **2016,** *61* (8), 2828-2843.
120. Bai, Y. G.; Zeng, S. J.; Bai, L.; Gao, H. S.; Zhou, Z. M.; Zhang, X. P., Highly Efficient Dehydration of Ethyl Acetate using Strong Hydrophilic Ionic Liquids. *Ind. Eng. Chem* **2020,** *59* (38), 16751-16761.
121. Yang, F. X.; Feng, P.; Chen, L.; Wang, X. P.; Tan, H. Z., Influences of Organic Solvents on the Properties of 1-Butyl-3-methylimidazolium Acetate. *J. Chem. Eng. Data* **2020,** *65* (4), 1911-1918.
122. Stevanovic, S.; Podgorsek, A.; Padua, A. A.; Costa Gomes, M. F., Effect of water on the carbon dioxide absorption by 1-alkyl-3-methylimidazolium acetate ionic liquids. *J. Phys. Chem. B* **2012,** *116* (49), 14416-25.
123. Balchandani, S.; Mandal, B.; Dharaskar, S., Measurements and modeling of vapor liquid equilibrium of CO_2 in amine activated imidazolium ionic liquid solvents. *Fluid Phase Equilib.* **2020,** *521*, 112643-112658.
124. Crosthwaite, J. M.; Muldoon, M. J.; Dixon, J. K.; Anderson, J. L.; Brennecke, J. F., Phase transition and decomposition temperatures, heat capacities and viscosities of pyridinium ionic liquids. *J. Chem. Thermodyn.* **2005,** *37* (6), 559-568.
125. Maginn, E. *Design and Evaluation of Ionic Liquids as Novel CO2 Absorbents*; Office of Scientific and Technical Information (OSTI): **2007**, DOI: 10.2172/969140 (accessed 2023-08-14).
126. Pandit, S. A.; Rather, M. A.; Bhat, S. A.; Rather, G. M.; Bhat, M. A., Influence of the Anion on the Equilibrium and Transport Properties of 1-Butyl-3-methylimidazolium Based Room Temperature Ionic Liquids. *J. Solution Chem.* **2016,** *45* (12), 1641-1658.
127. Bogolitsyn, K. G.; Skrebets, T. E.; Makhova, T. A., Physicochemical properties of 1-butyl-3-methylimidazolium acetate. *Russ. J. Gen. Chem.* **2009,** *79* (1), 125-128.
128. Kanakubo, M.; Makino, T.; Umecky, T., CO_2 solubility in and physical properties for ionic liquid mixtures of 1-butyl-3-bethylimidazolium acetate and 1-butyl-3-methylimidazolium bis(trifluoromethanesulfonyl)amide. *J. Mol. Liq.* **2016,** *217*, 112-119.
129. Fendt, S.; Padmanabhan, S.; Blanch, H. W.; Prausnitz, J. M., Viscosities of Acetate or Chloride-Based Ionic Liquids and Some of Their Mixtures with Water or Other Common Solvents. *J. Chem. Eng. Data* **2011,** *56* (1), 31-34.
130. Kakinuma, S.; Shirota, H., Femtosecond Raman-Induced Kerr Effect Study of Temperature-Dependent Intermolecular Dynamics in Pyrrolidinium-Based Ionic Liquids: Effects of Anion Species. *J. Phys. Chem. B* **2019,** *123* (6), 1307-1323.
131. Seki, S.; Serizawa, N.; Ono, S.; Takei, K.; Hayamizu, K.; Tsuzuki, S.; Umebayashi, Y., Densities, Viscosities, and Refractive Indices of Binary Room-Temperature Ionic Liquids with Common Cations/Anions. *J. Chem. Eng. Data* **2019,** *64* (2), 433-441.

132. Makino, T.; Kanakubo, M.; Umecky, T.; Suzuki, A.; Nishida, T.; Takano, J., Electrical Conductivities, Viscosities, and Densities of N-Methoxymethyl- and N-Butyl-N-methylpyrrolidinium Ionic Liquids with the Bis(fluorosulfonyl)amide Anion. *J. Chem. Eng. Data* **2012,** *57* (3), 751-755.
133. Sánchez-Ramírez, N.; Assresahegn, B. D.; Bélanger, D.; Torresi, R. M., A Comparison among Viscosity, Density, Conductivity, and Electrochemical Windows of N-n-Butyl-N-methylpyrrolidinium and Triethyl-n-pentylphosphonium Bis(fluorosulfonyl imide) Ionic Liquids and Their Analogues Containing Bis(trifluoromethylsulfonyl) Imide Anion. *J. Chem. Eng. Data* **2017,** *62* (10), 3437-3444.
134. Domanska, U.; Okuniewska, P.; Paduszynski, K.; Krolikowska, M.; Zawadzki, M.; Wieckowski, M., Extraction of 2-Phenylethanol (PEA) from Aqueous Solution Using Ionic Liquids: Synthesis, Phase Equilibrium Investigation, Selectivity in Separation, and Thermodynamic Models. *J. Phys. Chem. B* **2017,** *121* (32), 7689-7698.
135. Havlová, M.; Dohnal, V., Phase equilibria and thermophysical properties of aqueous solutions of two bis(fluorosulfonyl)imide-based ionic liquids. *Fluid Phase Equilib.* **2021,** *547*, 113137-113151.
136. Sayah, S.; Ghamouss, F.; Santos-Peña, J.; Tran-Van, F.; Lemordant, D., The Intriguing Properties of 1-Ethyl-3-methylimidazolium bis(fluorosulfonyl)imide Ionic Liquid. *J. Solution Chem.* **2019,** *48* (7), 992-1008.
137. Skonieczny, M.; Królikowska, M., Thermodynamic Properties of {Diethyl Phosphate-Based Ionic Liquid (1) + Ethanol (2)} Systems, Experimental Data and Correlation. *J. Chem. Eng. Data* **2022,** *67* (4), 869-885.
138. Cheng, S.; Musial, M.; Wojnarowska, Z.; Ngai, K. L.; Jacquemin, J.; Paluch, M., Universal scaling behavior of entropy and conductivity in ionic liquids. *J. Mol. Liq.* **2020,** *316*, 113824-113830.
139. de Pablo, L.; Segovia Puras, J. J.; Martín, C.; Bermejo, M. D., Determination of Density and Viscosity of Binary Mixtures of Water and Dimethyl Sulfoxide with 1-Ethyl-3-methylimidazolium Diethylphosphate $[EtMeIm]^+[Et_2PO_4]^-$ at Atmospheric Pressure. *J. Chem. Eng. Data* **2018,** *63* (4), 1053-1064.
140. Tenney, C. M.; Massel, M.; Mayes, J. M.; Sen, M.; Brennecke, J. F.; Maginn, E. J., A Computational and Experimental Study of the Heat Transfer Properties of Nine Different Ionic Liquids. *J. Chem. Eng. Data* **2014,** *59* (2), 391-399.
141. Yamamoto, T.; Matsubara, R.; Nohira, T., Highly Conductive Ionic Liquid Electrolytes for Potassium-Ion Batteries. *J. Chem. Eng. Data* **2021,** *66* (2), 1081-1088.
142. Gouveia, A. S. L.; Bernardes, C. E. S.; Tome, L. C.; Lozinskaya, E. I.; Vygodskii, Y. S.; Shaplov, A. S.; Lopes, J. N. C.; Marrucho, I. M., Ionic liquids with anions based on fluorosulfonyl derivatives: from asymmetrical substitutions to a consistent force field model. *Phys. Chem. Chem. Phys.* **2017,** *19* (43), 29617-29624.
143. Matsumoto, K.; Nishiwaki, E.; Hosokawa, T.; Tawa, S.; Nohira, T.; Hagiwara, R., Thermal, Physical, and Electrochemical Properties of $Li[N(SO_2F)_2]$-[1-Ethyl-3-methylimidazolium]$[N(SO_2F)_2]$ Ionic Liquid Electrolytes for Li Secondary Batteries Operated at Room and Intermediate Temperatures. *J. Phys. Chem. A* **2017,** *121* (17), 9209-9219.
144. Seki, S.; Kobayashi, T.; Kobayashi, Y.; Takei, K.; Miyashiro, H.; Hayamizu, K.; Tsuzuki, S.; Mitsugi, T.; Umebayashi, Y., Effects of cation and anion on physical properties of room-temperature ionic liquids. *J. Mol. Liq.* **2010,** *152* (1-3), 9-13.
145. Harris, K. R.; Kanakubo, M., Self-Diffusion Coefficients and Related Transport Properties for a Number of Fragile Ionic Liquids. *J. Chem. Eng. Data* **2016,** *61* (7), 2399-2411.
146. Vallejo, J. P.; del Río, J. M. L.; Fernández, J.; Lugo, L., Tribological performance of silicon nitride and carbon black Ionanofluids based on 1-ethyl-3-methylimidazolium methanesulfonate. *J. Mol. Liq.* **2020,** *319*, 114335-114346.
147. Vataščin, E.; Havlová, M.; Dohnal, V., Phase equilibria and volumetric and viscosity behavior of the aqueous double salt ionic liquid $[EMIM]_x[SCN]$ $[MeSO3]_{(1-x)}$. *Fluid Phase Equilib.* **2021,** *548*, 113199-113210.
148. Bioucas, F. E. B.; Queirós, C. S. G. P.; Lozano-Martín, D.; Ferreira, M. S.; Paredes, X.; Santos, Â. F.; Santos, F. J. V.; Lopes, M. L. M.; Lampreia, I. M. S.; Lourenço, M. J. V.; de Castro, C. A. N.; Massonne, K., $[C_2mim][CH_3SO_3]$–A Suitable New Heat Transfer Fluid? Part 2: Thermophysical Properties of Its Mixtures with Water. *Ind. Eng. Chem* **2022,** *61* (5), 2280-2305.

149. Bioucas, F. E. B.; Vieira, S. I. C.; Lourenço, M. J. V.; Santos, F. J. V.; Nieto de Castro, C. A.; Massonne, K., [C2mim][CH3SO3] – A Suitable New Heat Transfer Fluid? Part 1. Thermophysical and Toxicological Properties. *Ind. Eng. Chem* **2018,** *57* (25), 8541-8551.
150. Cooper, E. I., New, Stable, Ambient-Temperature Molten Salts. *ECS Proceedings Volumes* **1992,** *1992-16* (1), 386-396.
151. Yang, F. X.; Wang, X. P.; Chen, Q.; Tan, H. Z., Improvement of the properties of 1-ethyl-3-methylimidazolium acetate using organic solvents for biofuel process. *J. Mol. Liq.* **2019,** *284*, 82-91.
152. Sescousse, R.; Le, K. A.; Ries, M. E.; Budtova, T., Viscosity of cellulose-imidazolium-based ionic liquid solutions. *J. Phys. Chem. B* **2010,** *114* (21), 7222-7228.
153. Zareiekordshouli, F.; Lashanizadehgan, A.; Darvishi, P., Experimental and theoretical study of CO_2 solubility under high pressure conditions in the ionic liquid 1-ethyl-3-methylimidazolium acetate. *J. Supercrit. Fluids* **2018,** *133*, 195-210.
154. Zhang, Q. G.; Cai, S. Y.; Zhang, W. B.; Lan, Y. L.; Zhang, X. Y., Density, viscosity, conductivity, refractive index and interaction study of binary mixtures of the ionic liquid 1-ethyl-3-methylimidazolium acetate with methyldiethanolamine. *J. Mol. Liq.* **2017,** *233*, 471-478.
155. Ries, M. E.; Radhi, A.; Green, S. M.; Moffat, J.; Budtova, T., Microscopic and Macroscopic Properties of Carbohydrate Solutions in the Ionic Liquid 1-Ethyl-3-methyl-imidazolium Acetate. *J. Phys. Chem. B* **2018,** *122* (37), 8763-8771.
156. Brehm, M.; Pulst, M.; Kressler, J.; Sebastiani, D., Triazolium-Based Ionic Liquids: A Novel Class of Cellulose Solvents. *J. Phys. Chem. B* **2019,** *123* (18), 3994-4003.
157. Shojaeian, A.; Hanifehei, M.; Fatoorehchi, H., Density, Viscosity, and Refractive Index Measurements for Binary Mixtures of N-Methyldiethanolamine (MDEA), Diethanolamine (DEA), and 2-Amino-2-methyl-1-propanol (AMP) with 1-Ethyl-3-methylimidazolium Acetate ([Emim][Ac]). *J. Chem. Eng. Data* **2021,** *66* (9), 3520-3530.
158. Chen, Z.; Huo, Y.; Long, P.; Shen, H.; Lee, J. M., Effects of electrostatic interaction on the properties of ionic liquids correlated with the change of free volume. *Phys. Chem. Chem. Phys.* **2017,** *19* (7), 5389-5395.
159. Ghoshdastidar, D.; Ghosh, D.; Senapati, S., High Nucleobase-Solubilizing Ability of Low-Viscous Ionic Liquid/Water Mixtures: Measurements and Mechanism. *J. Phys. Chem. B* **2016,** *120* (3), 492-503.
160. Renda, C. M.; Patel, Y. K.; Henshaw, L. R.; Munson, K. T.; Fiebig, O. C.; Tran, A. T.; Shriver, J.; Cruz, J.; Yu, L.; Vaden, T. D., Thermodynamic and conductivity properties of acetic acid EMIMOAc ionic liquid solutions. *J. Mol. Liq.* **2016,** *216*, 710-715.
161. Wei, Y.; Zhang, W. B.; Zhang, X. Y.; Yang, H. G.; Zhang, Q. G., The Volumetric and Transport Properties of 1-Ethyl-3-Methylimidazolium Trifluoromethanesulfonate Ionic Liquid and Propylene Carbonate Binary System. *J. Solution Chem.* **2019,** *48* (2), 125-141.
162. Seddon, K. R.; Stark, A.; Torres, M.-J., Viscosity and Density of 1-Alkyl-3-methylimidazolium Ionic Liquids. In *Clean Solvents*, American Chemical Society: **2002**; Vol. 819, pp 34-49.
163. Anwar, N.; Riyazuddeen, Excess Molar Volumes, Excess Molar Isentropic Compressibilities, Viscosity Deviations, and Activation Parameters for 1-Ethyl-3-methyl-imidazolium Trifluoro-methanesulfonate + Dimethyl Sulfoxide and/or Acetonitrile at T = 298.15 to 323.15 K and P = 0.1 MPa. *J. Chem. Eng. Data* **2018,** *63* (2), 269-289.
164. Fatima, U.; Riyazuddeen; Alam, M. J.; Ahmad, S., Experimental thermophysical properties and DFT calculations of imidazolium ionic liquids and 2-butanol mixtures. *Fluid Phase Equilib.* **2020,** *508*, 112447-112458.
165. Bonhote, P.; Dias, A. P.; Papageorgiou, N.; Kalyanasundaram, K.; Gratzel, M., Hydrophobic, Highly Conductive Ambient-Temperature Molten Salts. *Inorg Chem* **1996,** *35* (5), 1168-1178.
166. Foo, C. K.; Leo, C. Y.; Aramesh, R.; Aroua, M. K.; Aghamohammadi, N.; Shafeeyan, M. S.; Shamiri, A., Density and viscosity of aqueous mixtures of N-methyldiethanolamines (MDEA), piperazine (PZ) and ionic liquids. *J. Mol. Liq.* **2015,** *209*, 596-602.
167. Nordness, O.; Simoni, L. D.; Stadtherr, M. A.; Brennecke, J. F., Characterization of Aqueous 1-Ethyl-3-Methylimidazolium Ionic Liquids for Calculation of Ion Dissociation. *J. Phys. Chem. B* **2019,** *123* (6), 1348-1358.

168. Aranowski, R.; Cichowska-Kopczyńska, I.; Dębski, B.; Jasiński, P., Conductivity and viscosity changes of imidazolium ionic liquids induced by H_2o and Co_2. *J. Mol. Liq.* **2016,** *221*, 541-546.
169. Gouveia, A. S. L.; Tome, L. C.; Lozinskaya, E. I.; Shaplov, A. S.; Vygodskii, Y. S.; Marrucho, I. M., Exploring the effect of fluorinated anions on the CO_2/N_2 separation of supported ionic liquid membranes. *Phys. Chem. Chem. Phys.* **2017,** *19* (42), 28876-28884.
170. Morgan, D.; Ferguson, L.; Scovazzo, P., Diffusivities of gases in room-temperature ionic liquids: Data and correlations obtained using a lag-time technique. *Ind. Eng. Chem* **2005,** *44* (13), 4815-4823.
171. Wang, G. X.; Xing, Z.; Zhang, X. Y.; Liu, F. J.; Zhang, Q. G., Thermodynamic, excess Properties and Intermolecular interactions of ionic liquid 1-Ethyl-3-Methylimidazolium thiocyanate and propylene carbonate mixtures. *J. Solution Chem.* **2022,** *51* (5), 594-608.
172. Mohan, M.; Banerjee, T.; Goud, V. V., Solid Liquid Equilibrium of Cellobiose, Sucrose, and Maltose Monohydrate in Ionic Liquids: Experimental and Quantum Chemical Insights. *J. Chem. Eng. Data* **2016,** *61* (9), 2923-2932.
173. Vataščin, E.; Dohnal, V., Thermodynamic properties of aqueous solutions of [EMIM] thiocyanate and [EMIM] dicyanamide. *J. Chem. Thermodyn.* **2017,** *106*, 262-275.
174. Lladosa, E.; Loras, S.; Poy, H.; Caballero, L., Thermophysical Properties of Mixtures of 1-Ethyl-3-methylimidazolium Methylsulfate or 1-Ethyl-3-methylimidazolium Thiocyanate with Alcohols. *J. Chem. Eng. Data* **2021,** *66* (2), 968-978.
175. Harris, K. R.; Kanakubo, M., Temperature and Pressure Dependence of the Viscosity of the Ionic Liquids 1-Hexyl-3-methylimidazolium Tetrafluoroborate and 1-Ethyl- and 1-Hexyl-3-methylimidazolium Bis(trifluoromethylsulfonyl)amides. *J. Chem. Eng. Data* **2021,** *66* (12), 4618-4628.
176. Atilhan, M.; Jacquemin, J.; Rooney, D.; Khraisheh, M.; Aparicio, S., Viscous Behavior of Imidazolium-Based Ionic Liquids. *Ind. Eng. Chem* **2013,** *52* (47), 16774-16785.
177. Ahosseini, A.; Scurto, A. M., Viscosity of imidazolium-based ionic liquids at elevated pressures: Cation and anion effects. *Int. J. Thermophys.* **2008,** *29* (4), 1222-1243.
178. Tariq, M.; Carvalho, P. J.; Coutinho, J. A. P.; Marrucho, I. M.; Lopes, J. N. C.; Rebelo, L. P. N., Viscosity of (C2–C14) 1-alkyl-3-methylimidazolium bis(trifluoromethylsulfonyl)amide ionic liquids in an extended temperature range. *Fluid Phase Equilib.* **2011,** *301* (1), 22-32.
179. Liu, Q.; Ma, L.; Wang, S.; Ni, Z.; Fu, X.; Wang, J.; Zheng, Q., Study on the properties of density, viscosity, excess molar volume, and viscosity deviation of [C_2mim][NTf_2], [C_2mmim][NTf_2], [C_4mim][NTf_2], and [C_4mmim][NTf_2] with PC binary mixtures. *J. Mol. Liq.* **2021,** *325*, 114573-114584.
180. Makino, T.; Kanakubo, M.; Masuda, Y.; Umecky, T.; Suzuki, A., CO2 absorption properties, densities, viscosities, and electrical conductivities of ethylimidazolium and 1-ethyl-3-methylimidazolium ionic liquids. *Fluid Phase Equilib.* **2014,** *362*, 300-306.
181. Hofmann, A.; Migeot, M.; Hanemann, T., Investigation of Binary Mixtures Containing 1-Ethyl-3-methylimidazolium Bis(trifluoromethanesulfonyl)azanide and Ethylene Carbonate. *J. Chem. Eng. Data* **2016,** *61* (1), 114-123.
182. Aljasmi, A.; AlJimaz, A. S.; AlKhaldi, K. H. A. E.; AlTuwaim, M. S., Dependency of Physicochemical Properties of Imidazolium Bis(Trifluoromethylsulfonyl)Imide-Based Ionic Liquids on Temperature and Alkyl Chain. *J. Chem. Eng. Data* **2022,** *67* (4), 858-868.
183. Bruce, D. W.; Cabry, C. P.; Lopes, J. N. C.; Costen, M. L.; D'Andrea, L.; Grillo, I.; Marshall, B. C.; McKendrick, K. G.; Minton, T. K.; Purcell, S. M.; Rogers, S.; Slattery, J. M.; Shimizu, K.; Smoll, E.; Tesa-Serrate, M. A., Nanosegregation and Structuring in the Bulk and at the Surface of Ionic-Liquid Mixtures. *J. Phys. Chem. B* **2017,** *121* (24), 6002-6020.
184. Chaabene, N.; Ngo, K.; Turmine, M.; Vivier, V., New hydrophobic deep eutectic solvent for electrochemical applications. *J. Mol. Liq.* **2020,** *319*, 114198.
185. Khalil, R.; Chaabene, N.; Azar, M.; Malham, I. B.; Turmine, M., Effect of the chain lengthening on transport properties of imidazolium-based ionic liquids. *Fluid Phase Equilib.* **2020,** *503*, 112316.
186. Liu, Q. S.; Zhao, L. W.; Zheng, Q. G.; Mou, L.; Zhang, P. F., Excess Molar Volume and Viscosity Deviation of [C2mim][NTf2]/[C4mim][NTf2] + DMC/DEC. *J. Chem. Eng. Data* **2018,** *63* (12), 4484-4496.
187. Noda, A.; Hayamizu, K.; Watanabe, M., Pulsed-Gradient Spin−Echo 1H and 19F NMR Ionic Diffusion Coefficient, Viscosity, and Ionic Conductivity of Non-Chloroaluminate Room-Temperature Ionic Liquids. *J. Phys. Chem. B* **2001,** *105* (20), 4603-4610.

188. Yadav, A.; Guha, A.; Pandey, A.; Pal, M.; Trivedi, S.; Pandey, S., Densities and dynamic viscosities of ionic liquids having 1-butyl-3-methylimidazolium cation with different anions and (trifluoromethylsulfonyl)imide anion with different cations in the temperature range (283.15 to 363.15) K. *J. Chem. Thermodyn.* **2018,** *116*, 67-75.
189. Pameté, E.; Gorska, B.; Béguin, F., Binary mixtures of ionic liquids based on EMIm cation and fluorinated anions: physico-chemical characterization in view of their application as low-temperature electrolytes. *J. Mol. Liq.* **2020,** *298*, 111959-111968.
190. Ramenskaya, L.; Grishina, E. P.; Kudryakova, N. O., Physicochemical features of short-chain 1-alkyl-3-methylimidazolium bis(trifluoromethylsulfonyl)-imide ionic liquids containing equilibrium water absorbed from air. *J. Mol. Liq.* **2018,** *272*, 759-765.
191. Tokuda, H.; Hayamizu, K.; Ishii, K.; Susan, M. A.; Watanabe, M., Physicochemical properties and structures of room temperature ionic liquids. 2. Variation of alkyl chain length in imidazolium cation. *J. Phys. Chem. B* **2005,** *109* (13), 6103-6110.
192. Papović, S.; Bešter-Rogač, M.; Vraneš, M.; Gadžurić, S., The effect of the alkyl chain length on physicochemical features of (ionic liquids +γ-butyrolactone) binary mixtures. *J. Chem. Thermodyn.* **2016,** *99*, 1-10.
193. Anwar, N.; Riyazuddeen; Yasmeen, S., Volumetric, compressibility and viscosity studies of binary mixtures of [EMIM][NTf_2] with ethylacetate/methanol at (298.15-323.15) K. *J. Mol. Liq.* **2016,** *224*, 189-200.
194. Doblinger, S.; Silvester, D. S.; Costa Gomes, M., Functionalized Imidazolium Bis(trifluoromethylsulfonyl)imide Ionic Liquids for Gas Sensors: Solubility of H_2, O_2 and SO_2. *Fluid Phase Equilib.* **2021,** *549*, 113211-113217.
195. Camper, D.; Becker, C.; Koval, C.; Noble, R., Diffusion and solubility measurements in room temperature ionic liquids. *Ind. Eng. Chem* **2006,** *45* (1), 445-450.
196. Kadyan, A.; Pandey, S., Lithium bis(trifluoromethylsulfonyl)imide-added ionic liquid 1-ethyl-3-methylimidazolium bis(trifluoromethylsulfonyl)imide mixture: Densities and dynamic viscosities in the temperature range (298.15–358.15)K. *J. Chem. Thermodyn.* **2018,** *116*, 159-165.
197. Slattery, J. M.; Daguenet, C.; Dyson, P. J.; Schubert, T. J.; Krossing, I., How to predict the physical properties of ionic liquids: a volume-based approach. *Angew Chem Int Ed Engl* **2007,** *46* (28), 5384-5388.
198. Allan, D. R.; Clark, S. J., Impeded dimer formation in the high-pressure crystal structure of formic acid. *Phys. Rev. Lett.* **1999,** *82* (17), 3464-3467.
199. Jenkins, H. D.; Roobottom, H. K.; Passmore, J.; Glasser, L., Relationships among Ionic Lattice Energies, Molecular (Formula Unit) Volumes, and Thermochemical Radii. *Inorg Chem* **1999,** *38* (16), 3609-3620.
200. Oswald, I. D. H.; Urquhart, A. J., Polymorphism and polymerisation of acrylic and methacrylic acid at high pressure. *Crystengcomm* **2011,** *13* (14), 4503-4507.
201. Dawson, A.; Allan, D. R.; Parsons, S.; Ruf, M., Use of a CCD diffractometer in crystal structure determinations at high pressure. *J. Appl. Crystallogr.* **2004,** *37* (3), 410-416.
202. Williams, P. A.; Hughes, C. E.; Harris, K. D., L-Lysine: exploiting powder X-ray diffraction to complete the set of crystal structures of the 20 directly encoded proteinogenic amino acids. *Angew. Chem. Int. Ed.* **2015,** *54* (13), 3973-3977.
203. Nockemann, P.; Binnemans, K.; Thijs, B.; Parac-Vogt, T. N.; Merz, K.; Mudring, A. V.; Menon, P. C.; Rajesh, R. N.; Cordoyiannis, G.; Thoen, J.; Leys, J.; Glorieux, C., Temperature-driven mixing-demixing behavior of binary mixtures of the ionic liquid choline bis(trifluoromethylsulfonyl)imide and water. *J. Phys. Chem. B* **2009,** *113* (5), 1429-1437.
204. Pereira, J. F. B.; Barber, P. S.; Kelley, S. P.; Berton, P.; Rogers, R. D., Double salt ionic liquids based on 1-ethyl-3-methylimidazolium acetate and hydroxyl-functionalized ammonium acetates: strong effects of weak interactions. *Phys. Chem. Chem. Phys.* **2017,** *19* (39), 26934-26943.
205. Mesto, E.; Quaranta, E., Hydrogen-bonded and pi-interaction assembly in two 8-alkoxycarbonyl-1,8-diazabicyclo[5.4.0]undec-7-enium chloride salts. *Acta Crystallogr. Sect. C: Cryst. Struct. Commun.* **2013,** *69* (4), 444-447.

206. Ezra, F. S.; Collin, R. L., The crystal structure of magnesium diethyl phosphate, $Mg[PO_2(OC_2H_5)_2]_2$. *Acta Crystallogr. Sect. B: Struct. Sci.* **1973,** *29* (7), 1398-1403.
207. Santos, C. S.; Rivera-Rubero, S.; Dibrov, S.; Baldelli, S., Ions at the surface of a room-temperature ionic liquid. *J. Phys. Chem. C* **2007,** *111* (21), 7682-7691.
208. Bond, A. D., On the crystal structures and melting point alternation of the n-alkyl carboxylic acids. *New J. Chem.* **2004,** *28* (1), 104-114.
209. Strieter, F. J.; Templeton, D. H.; Scheuerman, R. F.; Sass, R. L., The crystal structure of propionic acid. *Acta Crystallogr.* **1962,** *15* (12), 1233-1239.
210. Jesariew, D.; Ilczyszyn, M. M.; Pietraszko, A., The crystal structure and the phase transitions of pyridinium trifluoromethanesulfonate. *MRX* **2014,** *1* (1), 015705-015724.
211. Wolstenholme, D. J.; Weigand, J. J.; E, M. C.; Cameron, T. S., The progression of strong and weak hydrogen bonds in a series of ethylenediammonium dithiocyanate derivatives--a new bonding protocol for macromolecules? *Phys. Chem. Chem. Phys.* **2008,** *10* (24), 3569-3577.
212. Karoui, S.; Kamoun, S.; Michaud, F., Ethyl-enedi-ammonium chloride thio-cyanate. *Acta Crystallogr. Sect. Sect. E: Struct. Rep. Online* **2013,** *69* (5), o669-o669.
213. Jenkins, H. D.; Liebman, J. F., Volumes of solid state ions and their estimation. *Inorg Chem* **2005,** *44* (18), 6359-6372.
214. Kooijam, H.; Godbole, M. D.; Bouwman, E.; Spek, A. L., Experimental Crystal Structure Determination. CCDC: CCDC Database (Number 617333), **2007**, (accessed 2023-08-20).
215. Giglmeier, H.; Kerscher, T.; Klufers, P.; Mayer, P., Pyrrolidinium chloride. *Acta Crystallogr. Sect. Sect. E: Struct. Rep. Online* **2009,** *65* (Pt 3), o592-o592.
216. Fuess, H.; Bats, J. W.; Dannöhl, H.; Meyer, H.; Schweig, A., Comparison of observed and calculated densities. XII. Deformation density in complex anions. II. Experimental and theoretical densities in sodium formate. *Acta Crystallogr. Sect. B: Struct. Sci.* **1982,** *38* (3), 736-743.
217. Azadbakht, R.; Hadadzadeh, H.; Amiri Rudbari, H., Bis(triethyl-ammonium) tetra-chlorido-cobaltate(II). *Acta Crystallogr. Sect. Sect. E: Struct. Rep. Online* **2012,** *68* (6), m859-m859.
